# RNA-binding proteins mediate the maturation of chromatin topology during differentiation

**DOI:** 10.1038/s41556-025-01735-5

**Published:** 2025-09-08

**Authors:** Bondita Dehingia, Małgorzata Milewska-Puchała, Marcin Janowski, Mahmoud-Reza Rafiee, Misbah Abbas, Aleksandra Piotrowska, Jan Senge, Piotr Blaut, Dietrich Walsh, Jacqueline Severino, Debadeep Chaudhury, Sajjad Iqbal, Rogelio Montiel-Manriquez, Sylwia Jankowska, Peyman Zare, Wolfgang Huber, Jianliang Xu, Rafael Casellas, Timo Zimmermann, Paweł Dłotko, Jeroen Krijgsveld, Aleksandra Pękowska

**Affiliations:** 1 Dioscuri Centre for Chromatin Biology and Epigenomics, Nencki Institute of Experimental Biology, Polish Academy of Sciences, Warsaw, Poland; 2 Genome Biology Unit, European Molecular Biology Laboratory, Heidelberg, Germany; 3 German Cancer Research Center, Heidelberg, Germany; 4 Dioscuri Centre for Topological Data Analysis, Institute of Mathematics, Polish Academy of Sciences, Warsaw, Poland; 5 European Molecular Biology Laboratory Imaging Centre, Heidelberg, Germany; 6 Unidad de Investigación Biomédica en Cáncer, Instituto Nacional de Cancerología-Instituto de Investigaciones Biomédicas, Universidad Nacional Autónoma de México, Mexico City, Mexico; 7 Lymphocyte Nuclear Biology, NIAMS, NIH, Bethesda, MD USA; 8 Division of Proteomics of Stem Cell and Cancer, German Cancer Research Center, Heidelberg, Germany; 9 Heidelberg University, Medical Faculty, Heidelberg, Germany; 10 Present Address: Centre for Genomic Regulation, The Barcelona Institute of Science and Technology, Barcelona, Spain; 11 Present Address: Department of Biology, Georgia State University, Atlanta, GA USA; 12 Present Address: Department of Hematopoietic Biology and Malignancy, Division of Cancer Medicine, The University of Texas MD Anderson Cancer Center, Houston, TX USA

**Keywords:** Nuclear organization, Differentiation, Stem-cell differentiation

## Abstract

Topologically associating domains (TADs) and chromatin architectural loops impact promoter–enhancer interactions, with CCCTC-binding factor (CTCF) defining TAD borders and loop anchors. TAD boundaries and loops progressively strengthen upon embryonic stem (ES) cell differentiation, underscoring the importance of chromatin topology in ontogeny. However, the mechanisms driving this process remain unclear. Here we show a widespread increase in CTCF–RNA-binding protein (RBP) interactions upon ES to neural stem (NS) cell differentiation. While dispensable in ES cells, RBPs reinforce CTCF-anchored chromatin topology in NS cells. We identify Pantr1, a non-coding RNA, as a key facilitator of CTCF–RBP interactions, promoting chromatin maturation. Using acute CTCF degradation, we find that, through its insulator function, CTCF helps maintain neuronal gene silencing in NS cells by acting as a barrier to untimely gene activation during development. Altogether, we reveal a fundamental mechanism driving developmentally linked chromatin structural consolidation and the contribution of this process to the control of gene expression in differentiation.

## Abstract (web summary)

Dehingia, Milewska-Puchała and colleagues find a pervasive lncRNA-mediated increase in interactions between CTCF and RNA-binding proteins during embryonic stem cell differentiation. These interactions reinforce chromatin architecture in neural cells.

## Main

At genomic distances that typically separate cognate promoter–enhancer pairs, mammalian genomes fold into domains of strong self-contacts termed topologically associating domains (TADs)^1–3^. TAD boundaries often interact with each other, forming architectural loops^4,5^. Multiple lines of evidence sustain the view that, by shaping the promoter–enhancer dialogue, TADs and architectural loops constitute functional units of genome organization^6–22^.

CCCTC-binding factor (CTCF) is a ubiquitously expressed, 11-zinc-finger DNA-binding protein^8^ exerting the role of an insulator shielding promoters from inappropriate enhancer activity^23–25^. By blocking the translocation of the loop-extruding cohesin complex (cohesin), which comprises structural maintenance of chromosomes 1 and 3, Rad21 and auxiliary proteins^26–30^, CTCF forms TAD borders and anchors of architectural loops^1,31,32^. CTCF sites making up the TAD borders are oriented towards the TAD centre^4,5,33^. Likewise, architectural loops overwhelmingly connect two convergently oriented CTCF-bound motifs^4,5^.

Consistent with its fundamental role in structuring the genome, CTCF is required for proper development and tissue homeostasis. In mice, deletion of CTCF arrests embryogenesis^8^, while loss of one copy of the CTCF gene predisposes the animals to cancer^34,35^. In humans, heterozygous deleterious mutations in CTCF cause mental retardation^36–38^. Furthermore, the removal or inversion of CTCF-binding sites loosens TAD borders and architectural loops^15,39,40^, leading to aberrant promoter–enhancer interactions, gene misexpression, developmental malformations^12,13,15,41^ or cancer^42,43^. In line with this, CTCF-bound loop anchors often overlap with genetic variants associated with diseases, including neuropsychiatric disorders^44^. Thus, architectural functions of CTCF underlie cell differentiation, embryonic development, organ homeostasis and higher-level brain functions.

Despite the preserved genomic coordinates of TAD borders and architectural loops across cell types^4,6,18,45,46^, and evolution^1,47–52^, their strengths are modulated in development with an associated impact on gene expression^39,53^. While TADs and loops emerge upon zygotic genome activation^8,10,19^, their strengths enhance progressively upon loss of totipotency accompanying the transition of the blastomeres to the pluripotent stem cell state^54–56^. TADs and loop structures develop as the cells exit pluripotency, commit to the neural lineage and subsequently differentiate into mature cell types^9,10,19^. Reprogramming reverts this effect^9^. However, what drives the general strengthening of architectural loops and TAD borders and what role it may fulfil in early embryonic development remains unknown.

Here, we investigate the mechanisms that drive the consolidation of chromatin topology upon mouse embryonic stem (ES) to neural stem (NS) cell differentiation. We find a pervasive increase in interactions between CTCF and RNA-binding proteins (RBPs) upon exit from pluripotency. We identify Pantr1, a long non-coding RNA (lncRNA) partner of CTCF strongly induced in the NS cells, as the mediator of the enhanced interactions between CTCF and RBPs upon ES cell differentiation. Pantr1 fosters chromatin insulation at TAD borders and stabilizes loops in the NS cells. Exploiting a CTCF–degron system and genome editing, we find a more pronounced CTCF insulator role in the lineage-committed than in the pluripotent stem cells. Altogether, we reveal a fundamental mechanism in which CTCF, RBPs and non-coding RNAs (ncRNAs) cooperatively reinforce chromatin architecture, guiding cell fate decisions during differentiation.

## Results

Chromatin conformation assays, including Hi-C, revealed the strengthening of CTCF-anchored architectural loops and TAD boundaries upon exit from pluripotency^9,10^. Here, we set out to address the molecular underpinnings of this process.

**Fig. 1:**
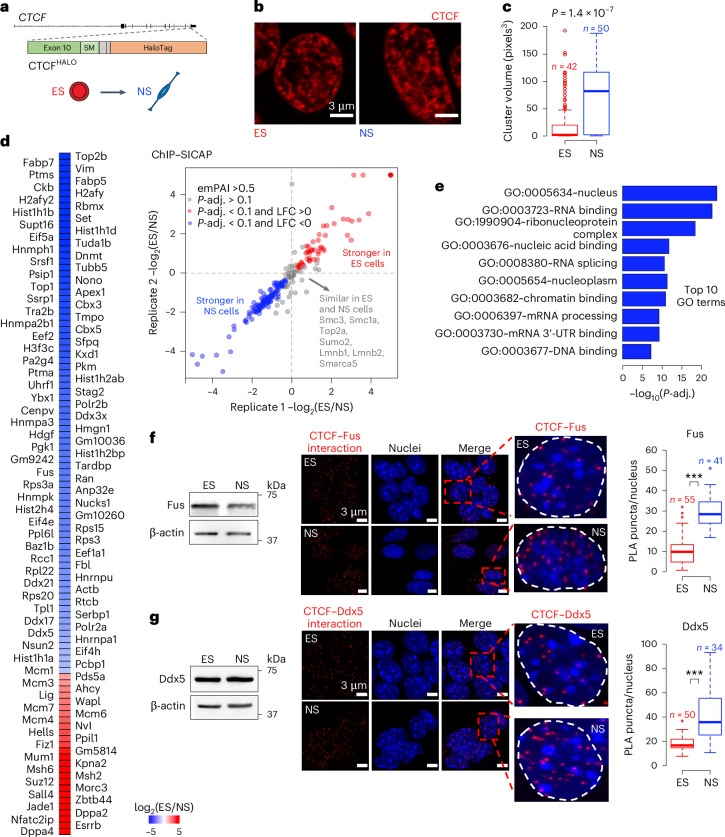
ES-to-NS cell transition is accompanied by enhanced CTCF clustering and increase in CTCF–RBP interactions. **a**, In CTCF^HALO^ ES cells, the HALO domain, along with the linking peptide (SM), is inserted in the C-terminal tail of the CTCF protein. **b**, The distribution of CTCF in ES and NS CTCF^HALO^ cells. Cells stained with 5 μM TMR were preextracted, fixed and imaged using a Zeiss LSM800 confocal microscope in AiryScan mode. **c**, Volumetric analysis of CTCF clusters in ES and NS cells. Box plots depict the distribution of the measured volumes of CTCF clusters in CTCF^HALO^ ES and NS cells (*P* = 1.4 × 10^−7^, two-sided *t*-test, *N*_experiments_ = 3; nuclei from one representative experiment are displayed). **d**, ChIP–SICAP reveals changes in the CTCF–protein interactome upon ES-to-NS cell transition. Proteins with high abundance are considered (emPAI >0.5). Left: heat map of logarithm base 2 of fold change of protein abundances between ES and NS cells (LFC); proteins with *P*-adj. < 0.1 are shown. Right: ChIP–SICAP LFC in two biological replicates. Red: proteins with decreased; blue: proteins with increased association with CTCF upon ES-to-NS transition. **e**, Gene Ontology (GO) analysis of proteins featuring an increased association with CTCF in NS compared with the ES cells. The top ten GO terms are displayed (*P*-adj. = 2.3 × 10^−23^). **f**, The association between CTCF and Fus in ES and NS cells. Left: western blot analysis of Fus expression in ES and NS CTCF^HALO^ cells. Middle: a representative example of a PLA readout in ES and NS cells (Zeiss LSM800 confocal microscope in AiryScan mode, *λ*_ex_ = 594 nm; *λ*_em_ = 624 nm). Right: box plot showing the distribution of the per-nucleus number of PLA puncta in ES and NS cells (***P < 0.01, two-sided *t*-test). **g**, The association between CTCF and DEAD-box RNA helicase Ddx5 in ES and NS cells, analogous to the one presented in **f** (***P < 0.01, two-sided *t*-test). In box plots, the box spans first and third quartile, the line inside the box indicates median, and whiskers indicate smallest (bottom) and largest (top) non-outlier in the data). Source numerical data and unprocessed blots are available in the extended data and source data, as well as in data repositories (see accession codes and the webpage associated with this study).

**Extended Data Fig. 1:**
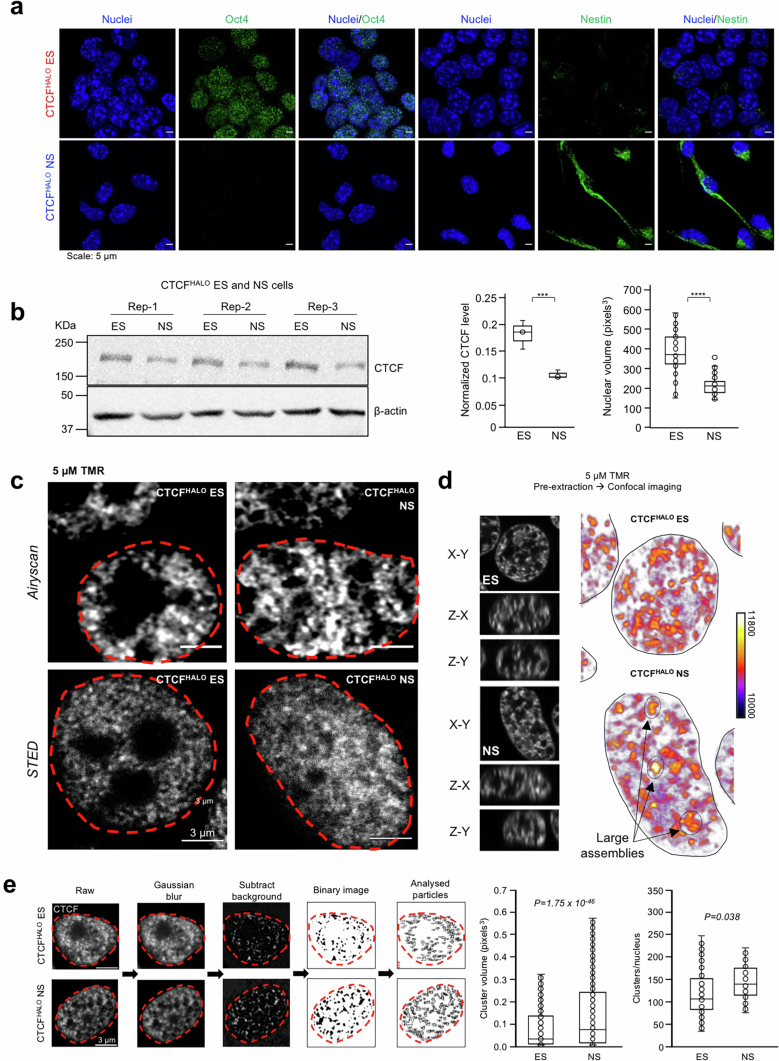
Relates to Fig. 2| The ES-to-NS cell transition is accompanied by changes in the nuclear distribution of CTCF and the gain of interactions between CTCF and RNA binding protein. **a**. Pluripotency and neural stem cell marker protein expression in ES and NS CTCF^HALO^ cells. ES cells express the pluripotency marker Oct4 and feature no signal for Nestin, a neural stem cell marker. On the contrary, NS cells express Nestin but maintain the pluripotency marker Oct4 silenced (secondary antibody coupled with Alexa Fluor was used, Zeiss LSM800 confocal microscope with Airyscan mode, the experiment was performed three times. ES-to-NS cell transition validation was displayed from one experiment). **b**. Western blot analysis of the level of CTCF protein in ES and NS CTCF^HALO^ cells. Beta-actin was used as a loading control. Bottom left panel: densitometry-based quantification of Western blot signal for CTCF referred to the signal of actin beta in ES and NS CTCF^HALO^ cells (***P = *0.0093*, two-sided t-test, N = 3; box spans first and third quartile, line inside the box indicates median, whiskers indicate smallest (bottom) and largest (top) non-outlier in the data)). Bottom right panel: nuclear volume in ES and NS cells (****P = *7.74* × *10*^−*13*^, two-sided t-test, N = 45; box spans first and third quartile, line inside the box indicates median, whiskers indicate smallest (bottom) and largest (top) non-outlier in the data)). **c**. Stimulated emission depletion (STED) microscopy and Confocal (Zeiss LSM800 with Airyscan mode) analyses of paraformaldehyde-fixed ES and NS cells with pre-extraction. The cells were incubated with TMR to visualise CTCF; the dye was then washed off. CTCF not bound to DNA was removed by treating the cells with the extraction buffer. Next, the cells were fixed (4% paraformaldehyde), mounted to the coverslips, and analyzed using STED or Zeiss LSM800 confocal microscope with Airyscan mode, experiment was performed three times, nuclei from one representative experiment were displayed. **d**. A representative heatmap of TMR signal intensity is displayed for fixed ES and NS cells. Image were acquired in Airyscan mode, experiment was performed three times, representative nuclei from one experiment was displayed. **e**. Enhanced clustering of CTCF in the NS cells is visualised by STED. Single plane images were considered. To remove noise, Gaussian blur was applied and background subtracted. Features were then identified and analysed (right). We observe more aggregates of CTCF in the NS than in the ES cells. (Right: N_ESnuclei_ = 92, N_ESnuclei_ = 93; **P = *0.038;* two-sided t-test; box spans first and third quartile, line inside the box indicates median, whiskers indicate smallest (bottom) and largest (top) non-outlier in the data). The CTCF aggregates in the NS cells are of a bigger volume than the aggregates in the ES cells (Left: N_ESnuclei_ = 92, N_ESnuclei_ = 93; ****P = *1.75* × *10*^−*46*^*;* two-sided t-test; box spans first and third quartile, line inside the box indicates median, whiskers indicate smallest (bottom) and largest (top) non-outlier in the data). Source numerical data and unprocessed blots are available in source data as well as in data repositories (see accession codes and the webpage associated with this study).

**Extended Data Fig. 2:**
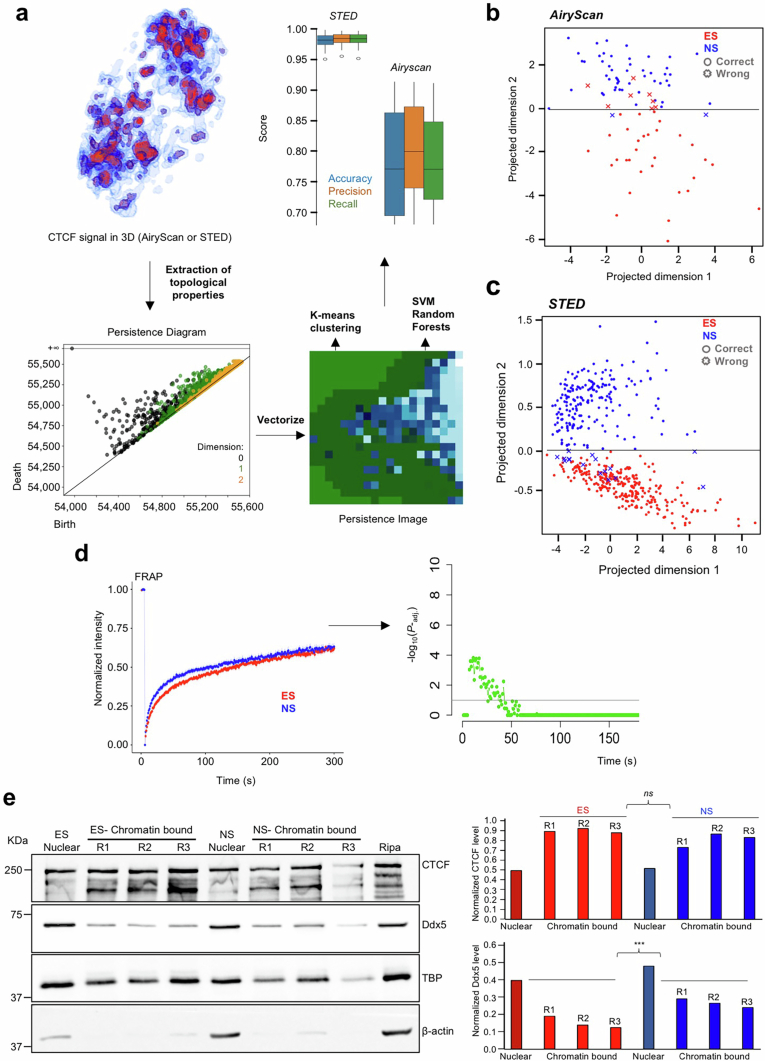
Relates to Fig. 1 Topological data analysis of CTCF signal distribution in the cell nucleus allows distinguishing ES and NS cells. **a**. Topological Data Analysis (TDA) to assess the differences in the pattern of CTCF distribution in ES and NS cells. Images were pre-processed to remove any intensity differences for the structural analysis of the cells. Three-dimensional Zeiss confocal microscope pictures (Zeiss LSM800 with Airyscan mode) as well as three-dimensional super-resolution STED microscope pictures of CTCF distribution were considered and converted into cubical complexes. Persistence images, Betti Curves as well as Persistence Statistics were considered to obtain vectorisation. Machine learning was used to train different classifiers (70–30% train test split) in both cases. Precision, (balanced) accuracy, and recall values for the cell type prediction based on the topological features of CTCF distribution in the cell nucleus are displayed with the boxplots (Airyscan: N_NucleiES_ = 39, N_NucleiNS_ = 52, STED: N_NucleiES_ = 269, N_NucleiNS_ = 209; box spans first and third quartile, line inside the box indicates median, whiskers indicate smallest (bottom) and largest (top) non-outlier in the data) for the best-performing classifier & vectorisation. **b**. 2D projection of a separating hyperplane in 3D after dimensionality reduction using Principal Component Analysis on the Betti curve vectorization of persistence diagrams from Airyscan pictures (explained variance = 53%). The projection shows the hyperplane as the abscissa. Red circles: correctly annotated ES cells; blue circles: correctly annotated NS cells; crosses incorrect annotation of cell type based on the TDA analysis of the CTCF signal. **c**. TDA of STED images of CTCF distribution in ES and NS cells. Analogue projection of a separating hyperplane as in b for persistence image vectorizations of STED pictures (explained variance = 99.9%). Red circles: correctly annotated ES cells; blue circles: correctly annotated NS cells; crosses incorrect annotation of cell type based on the TDA analysis of the CTCF signal. **d**. Fluorescence recovery after photobleaching (FRAP) analysis of TMR (concentration: 5 µM) labelled ES and NS CTCF^HALO^ cells (measurements were performed using Zeiss LSM780 confocal microscope). FRAP curves were normalized following the standard two-step method: first, the background signal was subtracted (background: signal coming outside of the nucleus), then the fluorescence signal was referred to that of the average fluorescence at the FRAP-ed area immediately before the bleach (first 5 frames). Finally, the normalized signal at the bleach cycle (t = 6s) was subtracted from the normalized values at t>6s.). CTCF displays overall similar dynamics of association with chromatin in ES and NS cells. **e**. Despite differences in the nuclear volume and the overall level of CTCF, the amount of chromatin-bound CTCF is similar in the ES and NS cells while Ddx5 interacts with chromatin more in the NS cells. The abundance of CTCF and Ddx5 chromatin-bound protein was determined in ES and NS cells using subcellular fractionation. Actin-beta and Tata binding protein (TBP) was used as a controls testifying successful nuclear fraction isolation (left panel). Right panel: densitometry-based quantification of Western blot signal for CTCF (top) and Ddx5 (bottom) in the nuclear and chromatin bound fractions. Ddx5 binding to chromatin is enhanced in NS cells compared to ES cells (*** *P* = *0.004*, two-sided t-test, N = 3). Source numerical data and unprocessed blot are available in source data as well as in data repositories (see accession codes and the webpage associated with this study).

CTCF can dimerize and form assemblies in the nucleoplasm^57–60^; the self-association and clustering of CTCF correlate with architectural loop formation observed by Hi-C^58,61^. Thus, we sought to compare the pattern of CTCF distribution in the ES and NS cell nucleoplasm. We took advantage of an ES cell line with a homozygous insertion of a HALO domain into the C-terminus of the CTCF protein (henceforth CTCF^HALO^; Fig. 1a). We propagated the CTCF^HALO^ ES cells in the presence of glycogen synthase kinase 3 (GSK3) and MAPK–Erk pathway inhibitors together with leukaemia inhibitory factor (LIF), to emulate the ground-state naive pluripotent stem identity (2i/LIF, henceforth ES cells). In parallel, we obtained NS cells from CTCF^HALO^ ES cells (Extended Data Fig. 1a). As previously reported^62^, CTCF protein levels were lower in NS cells compared with ES cells (Extended Data Fig. 1b, FC_ES/NS_ of 1.9, *P* < 0.01, two-sided *t*-test). Yet, at the same time, we found a similar amount of CTCF bound to chromatin (Extended Data Fig. 2e). Hence, the CTCF–DNA association is similar in ES and NS cells.

Regardless of the preserved association of CTCF in the cell nucleus in both cell types, both the near-super-resolution confocal (AiryScan) and super-resolution (stimulated emission depletion, STED) imaging of fixed cells prepared following an established sample preparation procedure^57,63,64^ revealed more prominent clusters of CTCF in the NS than in the ES cells (Fig. 1b,c and Extended Data Fig. 1b–e). To capture the spatial distribution of CTCF signals unbiasedly, we took advantage of topological data analysis (TDA). TDA combines algebraic topology with computational geometry, allowing the derivation of a set of sensitive measures of the structure under consideration without the need for image thresholding or additional processing (Extended Data Fig. 2a). Clustering, based on the TDA-inferred descriptors to the CTCF signal, revealed a clear separation of ES and NS cell nuclei (Extended Data Fig. 2a–c). Hence, loss of pluripotency is accompanied by a global change in the distribution of CTCF; neural lineage-committed cells feature larger CTCF clusters in the nucleoplasm.

CTCF binds to an extended CG-rich motif and features a particularity stable interaction with chromatin^65^. The removal of zinc finger domains of CTCF affects the dynamics of CTCF–DNA interactions^59,66,67^ and impacts loop formation^59,61,68^. We thus sought to test if loss of pluripotency is linked to altered CTCF–chromatin contacts. Fluorescence recovery after photobleaching (FRAP) in the CTCF^HALO^ ES and NS cells revealed modest differences in CTCF dynamics in the NS cells (Extended Data Fig. 2d). Together with our previous observations, this result suggests a largely preserved interaction dynamics of CTCF with DNA in both cell types (Extended Data Fig. 2e). Differences in the pattern of CTCF distribution in the nucleoplasm hinted at a yet unknown biochemical basis of the gain of CTCF architectural functions upon differentiation. Thus, we sought to determine the protein partners of CTCF in the ES and NS cells.

## Protein partners of CTCF in ES and NS cells

**Extended Data Fig. 3:**
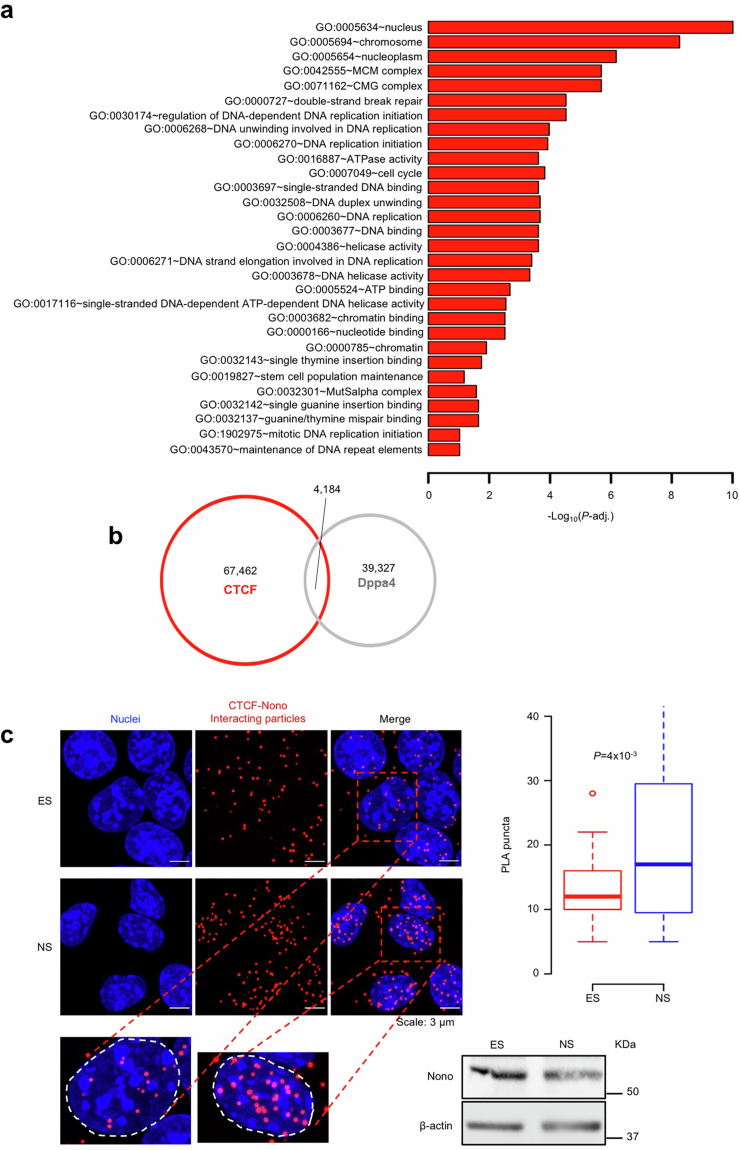
Relates to Fig. 1| The ES-to-NS cell transition is accompanied by changes in the nuclear distribution of CTCF and the gain of interactions between CTCF and RNA binding protein. **a**. Gene ontology (GO) analysis of proteins linked with CTCF in ES or NS cells (*P-adj*. < 2.2 × 10^−16^). **b**. Venn Diagram displaying the overlap between Dppa4 and CTCF binding sites in the ES cells. **c**. PLA analysis of CTCF-Nono interaction in ES and NS CTCF^HALO^ cells. Top: a representative example of proximity ligation assay (PLA) readout in ES and NS cells (CTCF-Nono interaction, Zeiss LSM800 confocal microscope with Airyscan mode, λex 594 nm; λem 624 nm). Bottom left: western blot analysis of expression of Nono in ES and NS CTCF^HALO^ cells. Right: boxplot showing the distribution of the per nucleus number of PLA puncta in ES and NS cells (*** *P* = *4* × *10*^−*3*^, two-sided t-test N_ESnuclei_ = 30, N_NSnuclei_ = 28; box spans first and third quartile, line inside the box indicates median, whiskerers indicate smalest (bottom) and largest (top) non-outlier in the data).). Source numerical data and unprocessed blot are available in source data as well as in data repositories (see accession codes and the webpage associated with this study).

CTCF interacts with various proteins, including cohesins and DNA topoisomerases^8^. To identify the chromatin-bound proteins that colocalize with CTCF, we took advantage of chromatin immunoprecipitation coupled with selective isolation of chromatin-associated proteins^69^ (ChIP–SICAP; Methods). We applied ChIP–SICAP to our ES and NS cells^9^. After data normalization and filtering, we retained 208 proteins with the highest abundance in our samples (exponentially modified protein abundace index (emPAI) >0.5; Fig. 1d and Supplementary Table 1)). As expected, the list was enriched with nuclear proteins (Extended Data Fig. 3a, adjusted *P* value (*P*-adj.) < 2.2 × 10^−16^). We identified the well-known partners of CTCF, including cohesins (Rad21, Smc1 and Smc3)^32,70,71^, multiple HEAT repeat-containing proteins associated with Kleisins (HAWKs), including Stag1, Stag2, Pds5a and Pds5b, as well as topoisomerase 2B^72^ and polycomb group proteins (Suz12)^73–75^. Consistent with the recently discovered targeting of early replication origins to anchors of loops and TAD boundaries^76,77^, ChIP–SICAP revealed colocalization of CTCF with the components of the DNA replication machinery. Likewise, ChIP–SICAP detected SWI/SNF family chromatin remodeller Smarca5, which regulates CTCF binding to DNA^78^. Thus, our ChIP–SICAP data faithfully reflect the protein interactome of CTCF.

## Global increase in CTCF–RBP interactions upon ES-to-NS transition

To identify candidate factors that could mediate the consolidation of CTCF-centred chromatin topology upon the exit from pluripotency, we quantitatively compared the abundance of proteins enriched at CTCF-bound chromatin in ES and NS cells (Fig. 1d). Sequence-specific transcription factors (TFs, including Dppa4, Esrrb, Dppa2, Zbtb44, Sall4 and Fiz1) were more abundant in the ES cell than in the NS cell CTCF interactome (Ybx1 was the only sequence-specific TF interacting with CTCF more in the NS cells). Confirming this result, we found that a fraction of CTCF-binding sites overlap with Dppa4-bound regions (Extended Data Fig. 3b).

Polycomb repressive complex 2 (PRC2) forms the so-called polycomb bodies (PcGs) in the ES cells; PcGs are disassembled upon neural differentiation of ES cells^79^. CTCF impacts the assembly of PRC2^73,74^, and the overlap between CTCF- and H3K27me3-enriched sites decreases upon neuronal induction of pluripotent stem cells^80^. In line with this, ChIP–SICAP revealed a substantial decrease in the interaction between CTCF and PRC2 proteins in NS cells compared with ES cells (Fig. 1d), corroborating our observations.

We found a remarkable overrepresentation of RBPs among targets featuring an increased interaction with CTCF in the NS cells (Fig. 1e, *P*-adj. = 2.3 × 10^−23^). The list of RBPs included DEAD-box RNA-helicase Ddx5 (1.8-fold increase in CTCF binding in the NS cells, *P*-adj. = 0.03), Fused in Sarcoma (Fus, 2.5-fold increase, *P*-adj. = 0.04) and Non-POU Domain Containing Octamer Binding (Nono^81–83^, 3.5-fold increase, *P*-adj. = 0.04).

Proximity ligation assays (PLAs) help to reveal the hotspots of interactions between two proteins^84^. To validate our ChIP–SICAP results orthogonally, we performed PLA in our ES^HALO^ and NS^HALO^ cells. We found a significant gain in the number of Ddx5-CTCF, Fus-CTCF (Fig. 1f,g) and Nono-CTCF puncta in the NS cells, confirming the ChIP–SICAP observations (Extended Data Fig. 3c). Western blot revealed that the protein levels of Ddx5, Fus and Nono were similar in both cell types (Fig. 1f,g and Extended Data Fig. 3c), ruling out the simple explanation that an increase in Ddx5, Fus or Nono levels drives the observed increase in the abundance of these factors within the CTCF interactome in the NS cells. This result, taken together with the observation that CTCF–chromatin association is similar in ES and NS cells (Extended Data Fig. 2e), further indicates a genuine enhancement of CTCF–RBP interactions in ES-to-NS differentiation.

Importantly, ChIP–SICAP revealed that the association between CTCF and the cohesin complex (Rad21, Smc3 and Smc1a) was similar in ES and NS cells, consistent with the notion that an equal fraction of CTCF-binding sites overlap Rad21 peaks in both cell types^9^. Likewise, this result suggests that ES and NS cells do not differ in the amount of cohesins loaded onto chromatin. Finally, chromatin remodeller Smarca5, which modulates CTCF binding and loop formation^78^, featured similar enrichment within the CTCF-associated proteome in ES and NS cells, suggesting that nucleosome positioning is probably not the mechanism underlying loop strengthening upon differentiation.

## RBPs warrant CTCF clustering in lineage-committed cells

**Extended Data Fig. 4:**
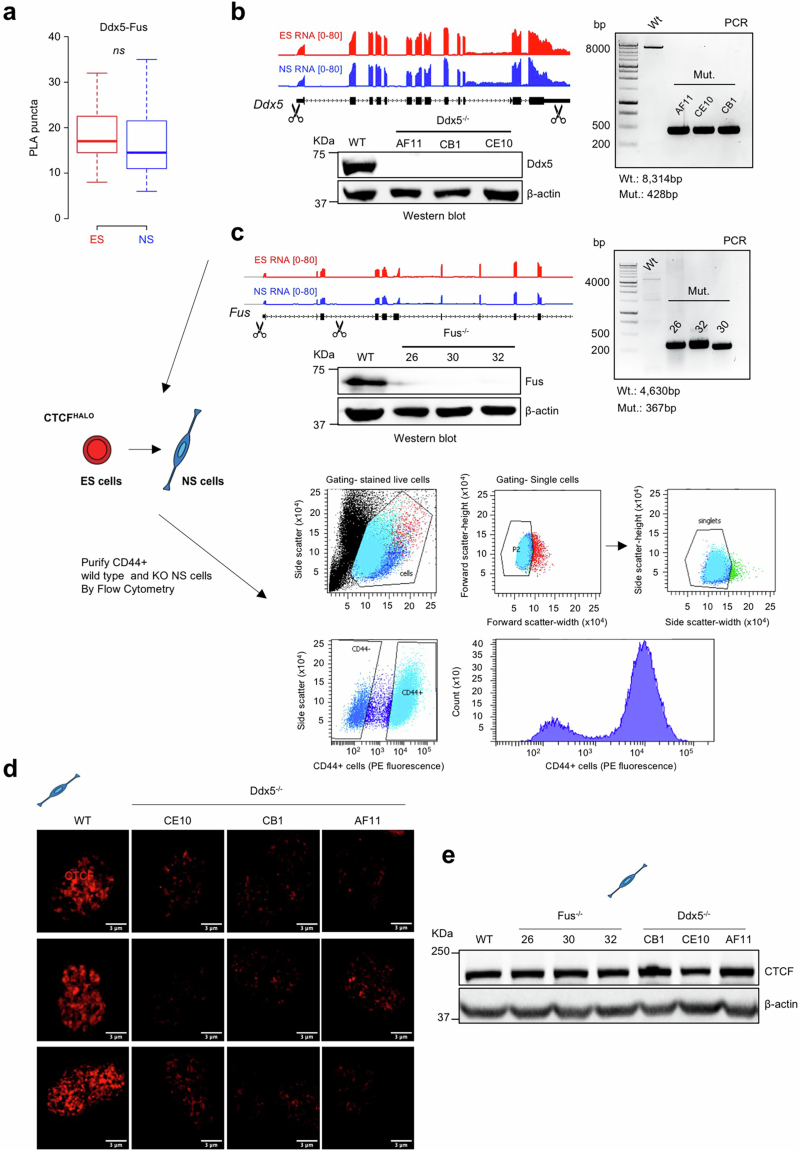
Relates to Fig. 2| Ddx5 and Fus impact CTCF functions in a differentiation-stage-specific manner. **a**. PLA for Ddx5-Fus interaction in ES and NS cells (N_ESnuclei_ = 16, N_NSnuclei_ = 20; box spans first and third quartile, line inside the box indicates median, whiskers indicate smallest (bottom) and largest (top) non-outlier in the data, *P* = *0.47*; *ns* = *non-significant*). **b**. Engineering of the Ddx5^−/−^ CTCF^HALO^ ES cells. Top panel: genome browser view of RNA-seq data tracks at the Ddx5 locus in CTCF^HALO^ ES and NS cells. Scissors indicate sites targeted by sgRNAs used for the CRISPR-Cas9 mediated genome editing. Bottom panel: Ddx5 protein level in wild type and Ddx5 deletion ES cells, experiment was performed two times. **c**. Engineering of the Fus^−/−^ CTCF^HALO^ ES cells. Top panel: genome browser view of RNA-seq data tracks at the Fus locus in CTCF^HALO^ ES and NS cells. Scissors indicate sites targeted by sgRNAs used for the CRISPR-Cas9 mediated genome editing. Bottom panel: Fus protein level in wild type and Fus deletion ES cells, experiment was performed two times. **d**. Loss of Ddx5 leads to loss of CTCF clustering in the nucleoplasm. Imaging of wild type and Ddx5^−/−^ NS CTCF^HALO^ cells. Cells were incubated with 5 µM TMR for 30 minutes at 37 °C. Following this, the cells were incubated with the pre-extraction buffer to remove the unbound fraction of CTCF. An additional Ddx5 deletion clone was obtained to further verify the effect (Zeiss LSM800 confocal microscope with Airyscan mode was used to acquire the images) experiment was performed three times. **e**. Western blot analysis of CTCF protein level in Ddx5^−/−^ and Fus^−/−^ NS CTCF^HALO^ cells, experiment was performed two times. Source numerical data, unprocessed gels and blot are available in source data as well as in data repositories (see accession codes and the webpage associated with this study).

CTCF–RBP–RNA interactions can impact chromatin architecture^58,61,85–88^. To better understand the contribution of RBPs in the maturation of CTCF-centred chromatin topology, we focused on Ddx5 and Fus. Ddx5 unwinds RNA^89,90^ and modulates the insulator functions of CTCF at the H19/IGF2 locus in a human cell line^85^. Fus is essential for the normal development and functions of the nervous system^91^. In haematopoietic cell aging, Fus regulates CTCF binding to DNA exemplifying an important role of this RBP in CTCF biology^92^. Fus does not seem to feature intrinsic enzymatic activity directed towards RNAs. Ddx5 and Fus may interact with each other. Yet, we found that the Ddx5–Fus interaction was similar in ES and NS cells (Extended Data Fig. 4a).

**Fig. 2:**
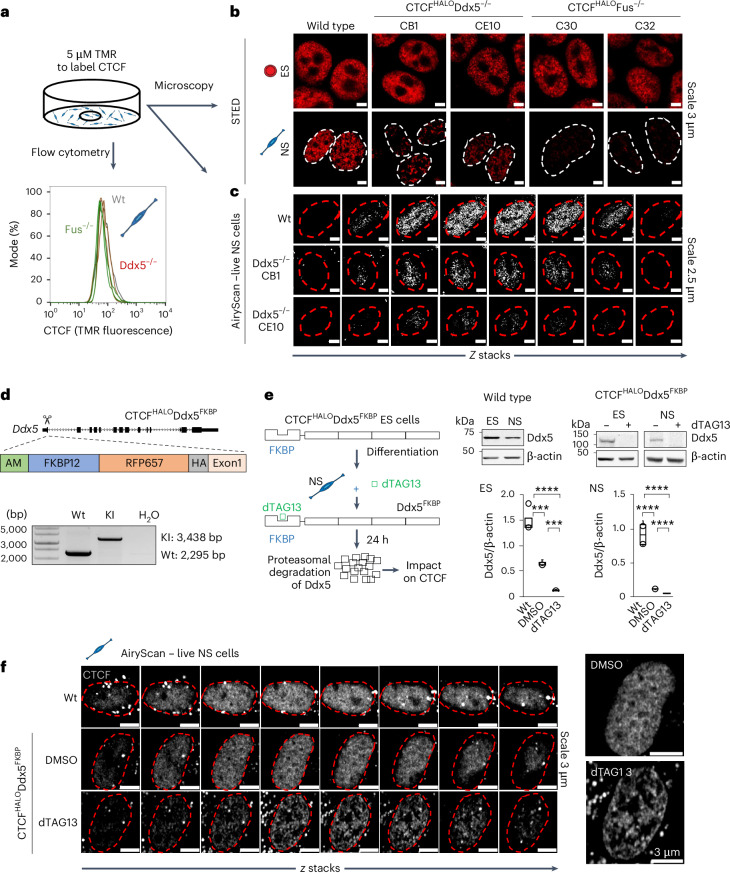
Ddx5 and Fus shape the distribution of CTCF in the nucleoplasm in a differentiation-stage-specific manner. **a**, The experimental design to determine the impact of deletion of Ddx5 and Fus on the distribution of CTCF in the ES and NS cell nucleoplasm. **b**, Loss of Ddx5 or Fus has a differentiation-stage-specific impact on CTCF clustering. STED microscopy of CTCF^HALO^ ES and NS wild-type (Wt) and Ddx5- or Fus-knockout (KO) lines stained by TMR. **c**, Live-cell near super-resolution microscopy of TMR-stained NS wild-type and Ddx5-knockout lines, *N*_experiments_ = 2; nuclei from one representative experiment are displayed. **d**, Genetic engineering of a Ddx5^FKBP^ degron CTCF^HALO^ ES cell line. Top: cassette containing an FKBP domain was inserted into the 5′ end of the Ddx5 coding sequence in the CTCF^HALO^ ES cell line. Bottom: PCR validation of the homozygous KI of the cassette, *N*_experiments_ = 3 in ES and NS cells; genotyping from one representative experiment is displayed. **e**, The addition of dTAG13 results in the removal of Ddx5 regardless of the differentiation state. Left: experimental design. Right: western blot validation of Ddx5 protein removal upon 24-h treatment with dTAG13 (*N*_experiments_ = 3 for DMSO and dTAG13 ES and NS cells, *N*_experiments_ = 4 for wild-type ES and NS cells; ES ***P_Wt vs DMSO_ = 0.001, ***P_DMSO vs dTAG13_ = 0.005 and ****P_Wt vs dTAG13_ = 0.0001; NS ****P_Wt vs DMSO_ = 0.0007, ****P_Wt vs dTAG13_ = 0.0004 and ****P_DMSO vs dTAG13_ = 0.0007 two-sided *t*-test; the box spans the first and third quartiles, the line inside the box indicates the median, and whiskers indicate the smallest (bottom) and largest (top) non-outlier in the data). **f**, Live-cell imaging of CTCF clusters in the nucleus in wild-type and Ddx5^FKBP^CTCF^HALO^ KI cells (Ddx5-KI) treated with either DMSO or dTAG13 for 24 h, *N*_experiments_ = 2; nuclei from one representative experiment are displayed. Source numerical data, unprocessed gels and blots are available in extended and source data as well as in data repositories (see also the webpage associated with this study).

**Extended Data Fig. 5:**
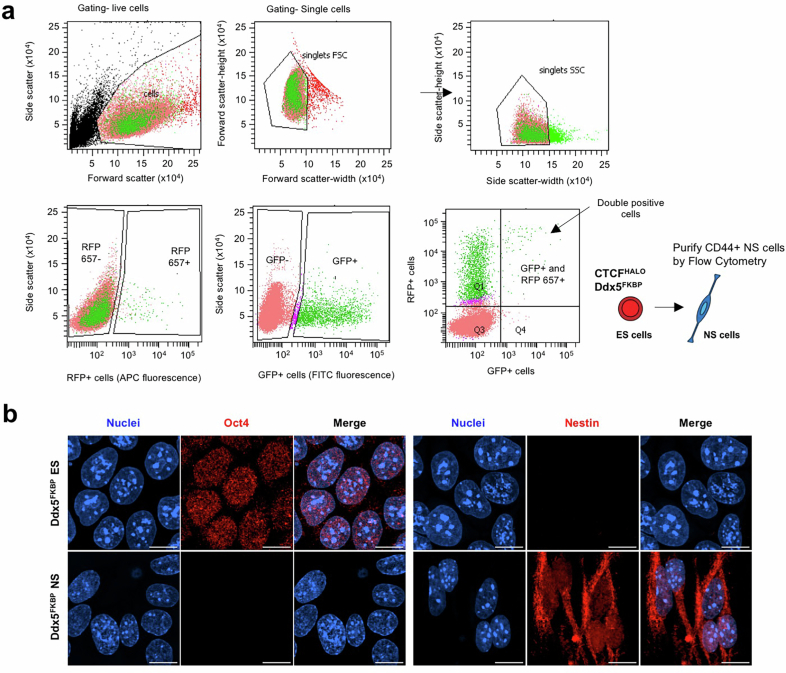
Relates to Fig. 2| Generation and validation of Ddx5 FKBP ES and NS cell lines. **a**. Purification of Ddx5^FKBP^ ES cells. CTCF^HALO^ ES cells were co-transfected with modified pSpCas9(BB)-2A-GFP (PX458) plasmid containing two sgRNA targeting Exon1 of the Ddx5 gene and the donor plasmid with the *knock-in* cassette (AM-FKBP-RFP657-HA). Cells showing both the RFP657 and GFP fluorescent signals were sorted at 36 hours post-transfection. **b**. Pluripotency and neural stem cell marker protein expression in ES and NS CTCF^HALO^ Ddx5^FKBP^ cells. ES cells express pluripotency marker Oct4 and feature no signal for Nestin, a neural stem cell marker. On the contrary, NS cells express Nestin but maintain the pluripotency marker Oct4 silenced (secondary antibody coupled with Alexa Fluor was used, Zeiss LSM800 confocal microscope with Airyscan mode, experiment was performed two times).

To address how Ddx5 and Fus may impact CTCF functions in development, we obtained Ddx5- and Fus-knockout CTCF^HALO^ ES cell lines using clustered regularly interspaced short palindromic repeats (CRISPR)–Cas9 (Extended Data Fig. 4b,c). Next, we differentiated the modified ES cell lines to NS cells (Methods). While the removal of Ddx5 or Fus had no notable impact on the distribution of CTCF in the ES cells, we observed a profound reduction of CTCF clustering in knockout NS cells (Fig. 2a–c and Extended Data Fig. 4d,e). This effect was not simply a consequence of a change in overall level of CTCF; flow cytometry and western blot analysis revealed a similar abundance of CTCF in all NS cell lines (Fig. 2a–c and Extended Data Fig. 4d,e). To test whether this effect was directly mediated by the Ddx5 protein, we obtained Ddx5^FKBP^CTCF^HALO^ ES cells and their NS cell derivatives amenable for acute depletion of Ddx5 upon treatment with dTAG13 (Fig. 2d–f and Extended Data Fig. 5). Despite the overall diminished Ddx5 levels after the genetic modification of the locus (Fig. 2e), further depletion of the protein led to loss of CTCF clustering in the nucleus of the Ddx5^FKBP^CTCF^HALO^ NS cells (Fig. 2f), corroborating our previous observations. Altogether, these results suggest that Ddx5 and Fus control architectural functions of CTCF in lineage-committed cells.

## RBP Ddx5 fosters CTCF binding to high-occupancy sites

**Fig. 3:**
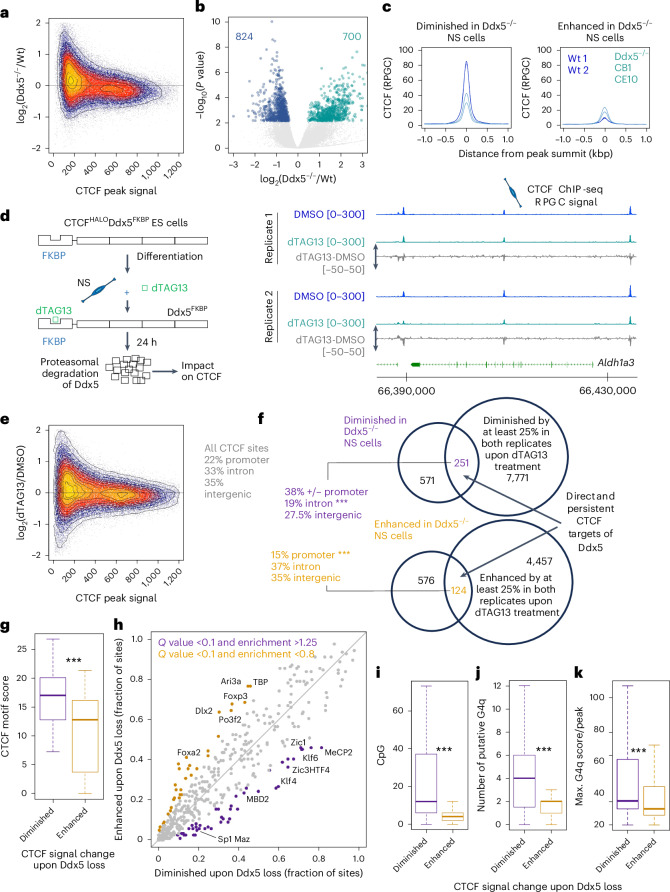
Ddx5 loss weakens CTCF binding at CG-rich locations featuring high propensity to form G4q. **a**, Mean difference and average intensity (MA) plot of the CTCF ChIP-seq peak signal (area under the curve ± 100 bp around the peak summit) change in wild-type and Ddx5^−/−^ NS cells. **b**, Volcano plot of peak signal (in **a**) in wild-type and Ddx5^−/−^ NS cells; blue: FDR <0.25, DESeq2 method. **c**, Average CTCF binding at sites identified as changing CTCF abundance upon Ddx5 loss (in **b**). **d**, Acute loss of Ddx5 leads to diminished CTCF binding at the Aldh1a3 locus. Left: experimental scheme. Ddx5 is removed upon the addition of dTAG13. Right: RPGC-normalized CTCF signal; grey tracks: difference between CTCF signal in dTAG13- and DMSO-treated NS cells. **e**, MA plot of the CTCF ChIP-seq peak signal (see **b**) following acute depletion of Ddx5 in NS cells. **f**, Identification of CTCF peaks affected by Ddx5 depletion. Two biological replicate samples of CTCF ChIP-seq for each genotype were considered (*N* = 2 wild type, *N* = 2 Ddx5^−/−^ clones along with *N* = 2 biological replicate treatments of Ddx5^FKBP^ KI NS cells with vehicle or dTAG13; ***P < 0.01, Fisher’s test). **g**, CTCF motif strength at peaks with altered CTCF signal upon Ddx5 removal (***P < 0.0001; two-sided *t*-test, peaks from **f** were considered; *n*_losing_ = 251, *n*_gaining_ = 124). **h**, TF-binding site (TFBS) enrichment at peaks with altered CTCF signal upon Ddx5 removal (peaks from **f** were considered; *n*_losing_ = 251, *n*_gaining_ = 124). **i**, The number of CpGs at peaks with altered CTCF signal upon Ddx5 removal (***P < 0.0001; two-sided *t*-test, peaks from **f** were considered; *n*_losing_ = 251, *n*_gaining_ = 124). **j**, The number of G4q (score >20) at peaks with altered CTCF signal upon Ddx5 removal (***P < 0.0001; two-sided *t*-test, peaks from **f** were considered; *n*_losing_ = 251, *n*_gaining_ = 124). **k**, G4q score at peaks with altered CTCF signal upon Ddx5 removal (***P < 0.0001; two-sided *t*-test, peaks from **f** were considered; *n*_losing_ = 251, *n*_gaining_ = 124). The box spans the first and third quartiles, the line inside the box indicates the median, and whiskers indicate the smallest (bottom) and largest (top) non-outlier in the data. Source numerical data are available in the extended data and source data, and in data repositories.

**Extended Data Fig. 6:**
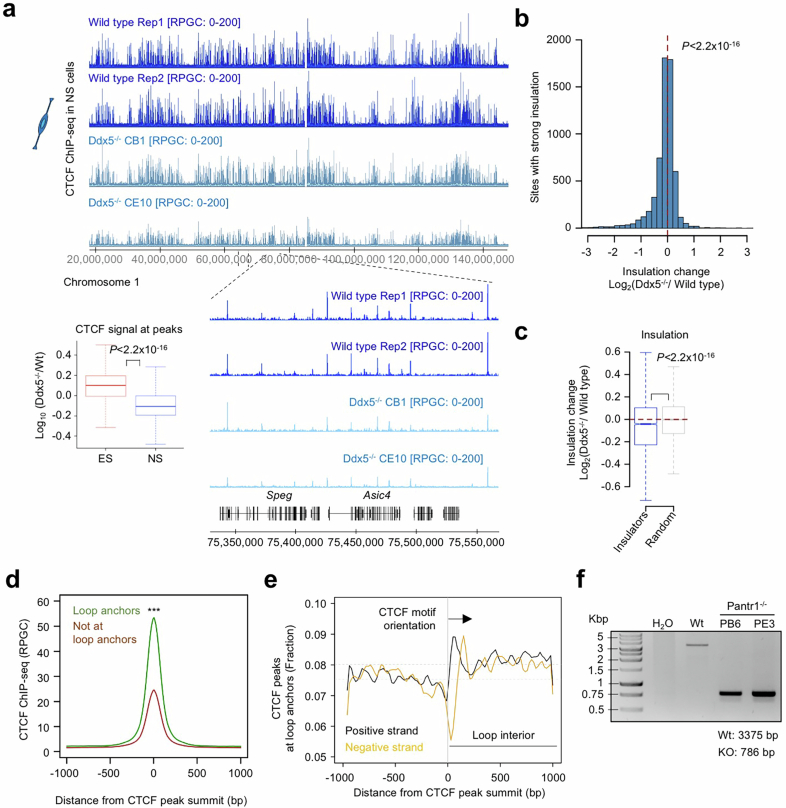
Relates to Figs. 3 & 4| Ddx5 impacts CTCF binding and chromatin insulation in the NS cells. **a**. Loss of Ddx5 diminishes CTCF binding to chromatin in the NS cells genome-wide. The top and bottom right panels are RPGC-normalized CTCF ChIP-seq tracks in two replicates of the wild type and Ddx5^−/−^ cells (two clonal lines were considered). The bottom left panel: boxplot illustrating the distribution of the logarithm base 2 of the fold change of CTCF signal in ES and NS cells (Peaks_ES_ = 12718, Peaks_NS_ = 42232; RPGC normalized signal integrated over regions −/+ 50bp around CTCF peak summits, *P* < *2.2* × *10*^*−16*^, two-sided t-test, merged ChIP-seq files for the two wild type and two knockout clones were considered in this analysis; box spans first and third quartile, line inside the box indicates median, whiskers indicate smallest (bottom) and largest (top) non-outlier in the data). **b**. Loss of Ddx5 impacts inter-domain contacts, leading to diminished insulation score (IS). Histogram of IS change in wild type versus Ddx5^−/−^ NS cells. Insulators identified in the wild type NS cells were considered (*n* = 6,132, *P* < *2.2* × *10*^−*16*^, two-sided t-test). **c**. IS change for insulators (left panel) and randomly picked genomic intervals (n = 6132, *P* < *2.2* × *10*^−*16*^, two-sided t-test; box spans first and third quartile, line inside the box indicates median, whiskers indicate smallest (bottom) and largest (top) non-outlier in the data).). **d**. Average signal of CTCF at peaks intersecting loop anchors (green) versus peaks in the rest of the genome (brown, *** - *P* < *2.2* × *10*^−*16*^, two-sided t-test). **e**. Distribution of putative G4 quadruplexes around the summits of CTCF peaks featuring a CTCF motif (within 20bp of the peak summit). G4q score was computed using R/Bioconductor package *pqsfinder* and G4q with a minimum score 10 were considered for this analysis. The direction of the CTCF motif is indicated with the arrow. **f**. Genotyping of Pantr1^−/−^ clones in CTCF^HALO^ ES cells, experiment was performed three times. Source numerical data and unprocessed gel are available in source data as well as in data repositories (see accession codes and the webpage associated with this study).

We next determined the impact of Ddx5 on CTCF. In contrast to ES cells, the CTCF–DNA association was diminished in Ddx5^−/−^ NS cells (Extended Data Fig. 6a), primarily at high-occupancy sites (Fig. 3a–c). Acute depletion of Ddx5 upon dTAG13 treatment had a similar effect (Fig. 3e). Hence, in the NS cells, the presence of Ddx5 fosters CTCF binding to DNA at high-occupancy sites.

To further discern the mechanisms through which Ddx5 impacts CTCF binding, we identified CTCF peaks affected in both genetic and acute depletion of Ddx5 in NS cells (Methods). More peaks were losing than gaining CTCF signal upon Ddx5 depletion in the NS cells (Fig. 3f). Locations where we scored lower CTCF binding in Ddx5 mutants featured a higher CTCF motif score (Fig. 3g) and were enriched in binding sites for other transcriptional regulators including ‘stripe’ TFs^93^ such as MAZ (Fig. 3h), which was previously shown to favour CTCF binding^94^.

Stripe TFs frequently bind to CG-rich sequences; such sites may form secondary DNA structures, including G4 quadruplexes (G4q), which impact TF binding^95^. We found that CTCF peaks losing signal in Ddx5^−/−^ cells were CpG rich (Fig. 3i) and featured a higher propensity to form G4q than the sites that gain CTCF signal in NS cells depleted for Ddx5 (Fig. 3i,j). Hence, the presence of Ddx5 is important to foster CTCF binding to strong motifs embedded within CpG-rich sequences that feature an enhanced propensity to form G4q.

## Ddx5 loss weakens chromatin architectural loops

**Fig. 4:**
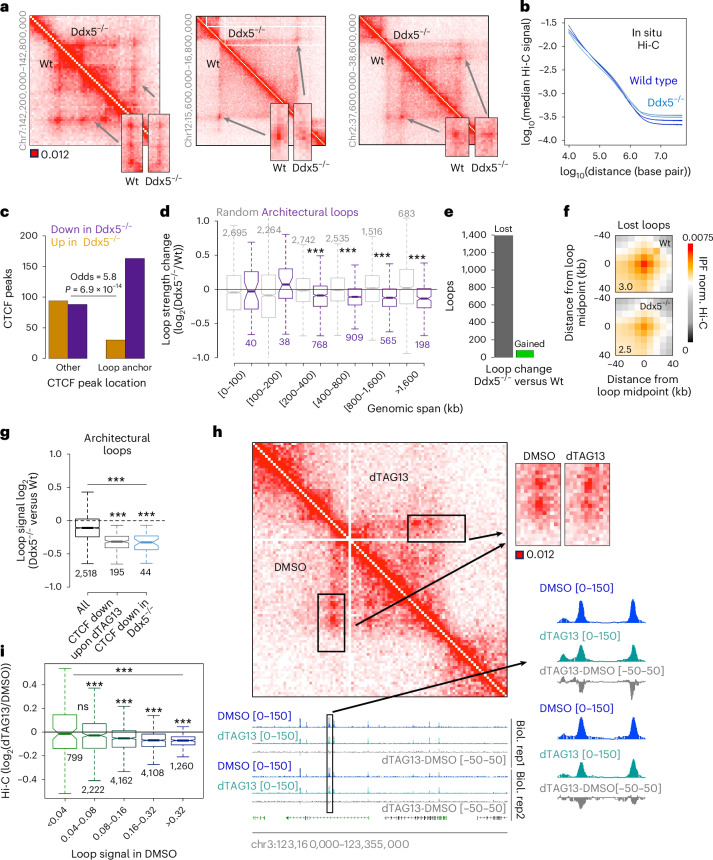
The presence of Ddx5 leads to CTCF–CTCF loop strengthening genome-wide. **a**, In situ Hi-C profiles in wild-type and Ddx5^−/−^ CTCF^HALO^ NS cells (each map is an average of two biological replicate libraries). **b**, Hi-C signal decline as a function of genomic distance in wild-type and Ddx5^−/−^ NS cells. **c**, The fraction of CTCF peaks with altered CTCF abundance upon Ddx5 loss at loop anchors or other locations in the genome (*P* = 6.9 × 10^−14^, Fisher’s exact test, peaks from Fig. 3b). **d**, Architectural loop strength (Methods, purple) is diminished upon loss of Ddx5 in the NS cells. Grey: bin pairs not connected by a loop but separated at equal genomic distance as the loop anchors; ***P < 0.01 two-sided *t*-test; numbers of instances per interaction size range are indicated in the figure. **e**, The number of loops featuring diminished or enhanced Hi-C signal in Ddx5^−/−^ compared with wild-type NS cells (Methods). **f**, APA of loops lost in the Ddx5^−/−^ NS cells (in **e**). Loops with anchors separated by more than 100 kb were considered. **g**, Changes in loop strength in wild-type and Ddx5^−/−^ NS cells. Changes in loop strength are shown for all loops and for loops with anchors overlapping CTCF peaks that decreased upon Ddx5 loss. Numbers of loops in each category are indicated; ***P < 0.01 two-sided *t*-test. **h**, Acute loss of Ddx5 impacts CTCF–CTCF loop formation. Hi-C was obtained in DMSO- and dTAG13-treated Ddx5^FKBP^ NS cells. Biol., biological. **i**, Acute loss of Ddx5 affects primarily strong loops (measured as the summed Hi-C signal in a 5 × 5 square centred at loop centroid at a resolution of 10 kb; numbers of loops in each category are indicated; ***P < 0.01 two-sided *t*-test; ns, nonsignificant). Each box spans the first and third quartiles, the line inside the box indicates the median, and whiskers indicate the smallest (bottom) and largest (top) non-outlier in the data. Source numerical data are available in extended and source data as well as in data repositories (see also the webpage associated with this study).

To address how Ddx5 contributes to chromatin topology, we carried out in situ Hi-C in the wild-type and Ddx5^−/−^ mutant NS cells (Fig. 4a). We identified 12,924 loops in the wild-type NS cells. Notably, CTCF peaks losing CTCF signal upon Ddx5 depletion were frequently at loop anchors (Fig. 4c). Furthermore, while the overall chromatin structure at length scales of up to a megabase was largely preserved in wild-type and Ddx5^−/−^ NS cells (Fig. 4a,b), architectural loops, which bridge convergently oriented CTCF binding sites (Methods), were weakened upon Ddx5 loss (Fig. 4d). In line with this, we found more diminished than enhanced loops in the Ddx5^−/−^ cells (Fig. 4e,f and Methods). The effect on loop signal scaled with the impact on CTCF binding to loop anchors in the Ddx5-knockout NS cells (Fig. 4g).

The differences in looping between the control and genetically modified cells were significant and, as expected^58,61^, subtle (Fig. 4d). Therefore, to further ascertain our observations, we obtained Hi-C libraries in dimethyl sulfoxide (DMSO)- and dTAG13-treated Ddx5^FKBP^CTCF^HALO^ NS cells (Fig. 4h). Architectural loops were significantly weaker upon dTAG13 treatment (Fig. 4i), supporting the conclusions reached using Ddx5^−/−^ NS cells.

At TAD borders, CTCF acts as an insulator, and this function scales with chromatin loop formation. Ddx5 has been previously shown to impact chromatin insulation at the IGF2-H19 imprinted locus^85^, which we also observed (Fig. 4a). Thus, we identified chromatin insulators and assessed the impact of Ddx5 loss on these elements. Loss of Ddx5 led to frequently diminished chromatin contact insulation (Extended Data Fig. 6b,c). Hence, Ddx5 contributes to loop and insulator strengthening in the lineage-committed cells.

CTCF peaks intersecting loop anchors featured higher CTCF signal than peaks at other locations in the genome (Extended Data Fig. 6d). As somewhat anticipated^96–98^, CTCF peak sequences at loop anchors presented a high propensity to form G4q (Extended Data Fig. 6e). The predicted G4q frequently aligned with the location of the CTCF motif (Extended Data Fig. 6e). As CTCF does not bind G4q^95,98^ and Ddx5 can dismantle G4q^99^, it is likely that, by removing G4q overlapping the CTCF motif, Ddx5 allows robust CTCF binding that may promote chromatin structure (Discussion).

## lncRNA Pantr1 regulates CTCF–RBP interactions

**Fig. 5:**
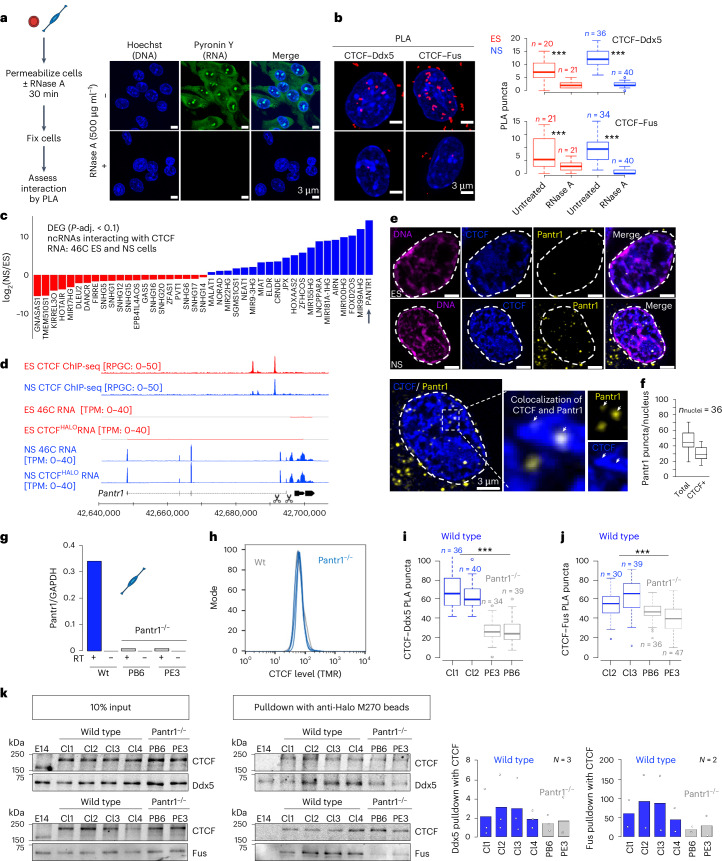
NS cell-specific lncRNA Pantr1 regulates the association between CTCF and RBPs. **a**, Left: acute RNA removal from the cells. Right: confocal microscopy images or Pyronin Y staining in untreated and RNAseA-treated NS CTCF^HALO^ cells. *N*_experiments_ = 3; nuclei from one representative experiment are displayed. **b**, PLA for CTCF–Ddx5 and CTCF–Fus interactions in untreated and RNaseA-treated CTCF^HALO^ cells (***P < 0.001; numbers of nuclei in each category are indicated). **c**, Polyadenylated lncRNAs that interact with CTCF^101^ and feature changes in expression level upon the ES-to-NS transition (*P*-adj. < 0.1 DESeq2 method; 46C ES and NS cell transcriptomes were considered in this analysis). **d**, Pantr1 is transcriptionally activated upon neural induction of distinct ES cell lines (RNA-seq: 46C and CTCF^HALO^ ES and NS cells, CTCF ChIP-seq: 46C ES and NS cells). Scissors: sgRNA locations in CRISPR–Cas9 editing. **e**, RNA-FISH of Pantr1 RNA (yellow) in CTCF^HALO^ ES and NS cell nuclei (CTCF: TMR blue; DNA, DAPI magenta). A single plane is displayed. *N*_experiments_ = 2 for NS and *N*_experiments_ = 1 for ES: nuclei from one representative experiment are displayed. **f**, Over 60% of Pantr1 puncta overlap CTCF-enriched regions in NS cell nuclei. (*N*_experiments_ = 2; representative nuclei from one experiment are shown). **g**, Normalized expression of Pantr1 in wild-type and Pantr1^−/−^ NS cells (quantitative reverse-transcriptase PCR (qRT–PCR); normalized average expression of Pantr1 in two technical replicate qRT–PCR reactions for a representative validation is displayed; validations were performed before each experiment using Pantr1^−/−^ NS cells (*n* > 3). **h**, Flow cytometry-assisted examination of CTCF protein expression in wild-type and Pantr1^−/−^ CTCF^HALO^ NS cells (*n*_Wt_ = 32,263, *n*_Pantr1 PB6_ = 19,544, *n*_Pantr1 PE3_ = 14,769; 5 µM TMR). **i**, Loss of Pantr1 disrupts CTCF–Ddx5 interactions. CTCF–Ddx5 interaction in wild-type and Pantr1^−/−^ NS cells (***P < 0.001; two-sided *t*-test). **j**, Analysis of CTCF–Fus interactions, analogous to the one presented in **i**. **k**, Co-immunoprecipitation (co-IP) assays assessing interactions between CTCF and Ddx5 and CTCF and Fus in wild-type and CTCF^HALO^ Pantr1^−/−^ NS cells (readout: western blot; an exemplary experiment is displayed). Four clones of wild-type and two clones of Pantr1^−/−^ were considered; three independent experiments probing CTCF–Ddx5 interactions and two experiments to probe CTCF–Fus interactions were performed. Each box spans the first and third quartiles, the line inside the box indicates the median, and the whiskers indicate the smallest (bottom) and largest (top) non-outlier in the data. Microscopy images were acquired with a Zeiss LSM800 confocal microscope with an AiryScan detector. Source numerical data are available in extended and source data as well as in data repositories (see accession codes and the webpage associated with this study).

Next, we asked what mediates the increase in CTCF–RBP interactions in differentiation. As we saw above, the levels of the RBPs do not change upon ES-to-NS transition. Hence, other factors probably impact the CTCF–RBP dialogue. CTCF interacts with RNA, and the removal of RNA or ablation of the RNA-binding domain of CTCF weakens CTCF clustering in the nucleoplasm, hampers its binding to DNA, disrupts architectural loops and leads to loss of insulation at a subset of TAD boundaries^58,61,100^. To define the role of RNAs in regulating CTCF–Ddx5 or CTCF–Fus contacts, we implemented a procedure to acutely deplete RNA from the cells (Fig. 5a). We found an absolute dependence of CTCF–RBP interactions on the presence of RNA (Fig. 5b).

CTCF co-immunoprecipitates with many ncRNAs^61,101,100^, and ncRNAs impact CTCF functions in a highly nuanced manner^58,61,85–87,100,102,103,104^. To address which RNAs contribute to regulating CTCF–RBP interactions in differentiation, we considered a database of ncRNA partners of CTCF^61^. Forty-one ncRNAs featured a significant change in expression upon neural induction of ES cells, including Pou3f3 Adjacent Non-Coding Transcript 1 (Pantr1, *P*-adj. < 0.01, DESeq2 method; Fig. 5c). Pantr1 was not only robustly induced upon ES-to-NS transition (Fig. 5c,d) but also expressed at a high level in the NS cells (Fig. 5d).

RNA species may favour CTCF binding in *cis*. Our chromatin immunoprecipitation sequencing (ChIP-seq) analysis revealed largely preserved CTCF binding at the Pantr1 locus in ES and NS cells (Fig. 5d). Thus, we hypothesized that the role of Pantr1 in regulating CTCF functions will probably manifest itself in *trans*. Corroborating this view, three-dimensional (3D) RNA fluorescence in situ hybridization (RNA-FISH) coupled with tetramethylrhodamine (TMR) staining revealed colocalization of CTCF with Pantr1 (Fig. 5e). Roughly 60% of Pantr1 puncta were opposed to CTCF clusters (Fig. 5f), indicating a tight interaction between Pantr1 and CTCF on chromatin in NS cells.

To better understand the contribution of Pantr1 to the regulation of chromatin topology, we engineered CTCF^HALO^ ES cells with a deletion in the 5′ end of the Pantr1 locus (Methods) and obtained NS cells from them. Pantr1 expression was decreased over tenfold in the mutant NS cells (Fig. 5g). While the loss of Pantr1 had no impact on the level of CTCF (Fig. 5h), it led to a marked loss of CTCF–Ddx5 interactions (Fig. 5i) and a significantly reduced interaction between CTCF and Fus, as revealed by both PLA (Fig. 5j) and co-immunoprecipitation coupled with western blot (Fig. 5k). Thus, transcriptional activation of lncRNAs upon loss of pluripotency leads to an enhanced pairing between RBPs and CTCF in the NS cells.

## Pantr1 strengthens chromatin loops and TAD borders in NS cells

**Fig. 6:**
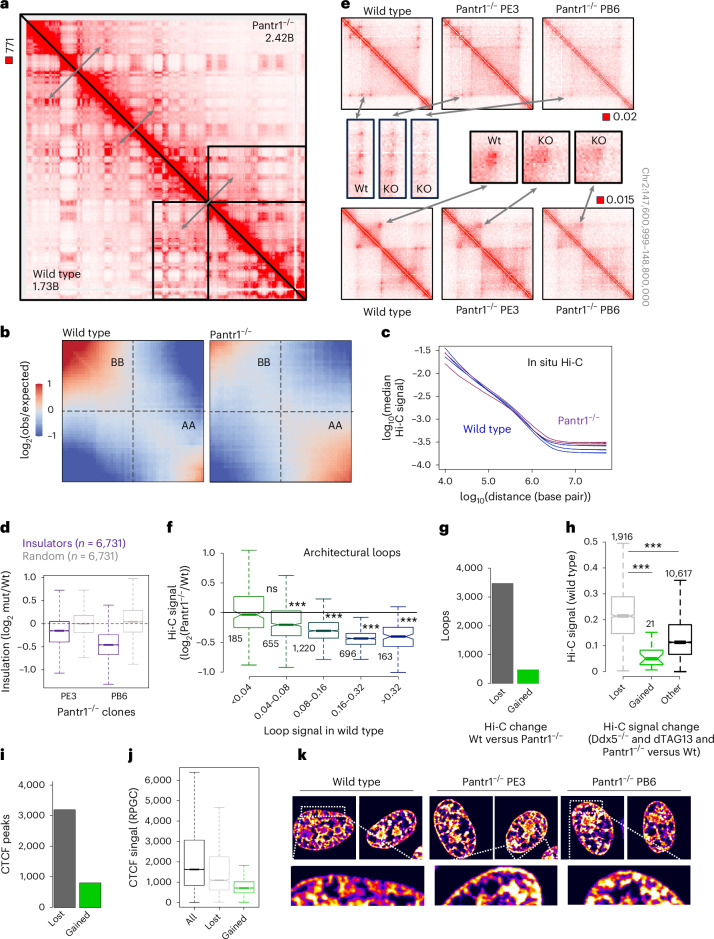
Loss of Pantr1 leads to weakening of CTCF-anchored chromatin topology in NS cells. **a**, The interaction profile of chromosome 2 in wild-type (lower triangle) and Pantr1^−/−^ NS cells (upper triangle; *N*_experiment_ = 1; *N*_wild-type clone_ = 1; *N*_Pantr1−/−_ _clone_ = 2). **b**, Saddle plots in wild-type and Pantr1-knockout NS cells (samples as in **a**; obs, observed). **c**, Hi-C signal as a function of genomic distance (samples as in **a**). **d**, Removal of Pantr1 leads to insulator weakening. Numbers of loops in each group are displayed (mut, mutant). **e**, Exemplary Hi-C profiles in wild-type and Pantr1^−/−^ NS cells. **f**, Loss of Pantr1 leads to weakening of strong architectural loops (***P < 0.01, two-sided *t*-test). **g**, There are more lost than gained loops in Pantr1^−/−^ NS cells compared with their wild-type counterparts. **h**, Loops with Hi-C signal diminished in both Ddx5-depleted and Pantr1^−/−^ NS cells display overall high Hi-C signal in the wild-type NS cells (***P < 0.01, two-sided t-test). **i**, More CTCF peaks lose than gain CTCF signal in the Pantr1^−/−^ NS cells. **j**, CTCF peaks that lose CTCF signal in the Pantr1^−/−^ NS cells compared with the wild-type cells feature high levels of CTCF. **k**, The distribution of CTCF in the cell nucleus in wild-type and Pantr1^−/−^ NS cells. CTCF clusters are enriched at the nuclear rim in the Pantr1^−/−^ NS cells compared with their wild-type counterparts. Microscopy images were acquired with a Zeiss LSM800 confocal microscope with an AiryScan detector. Each box spans the first and third quartiles, the line inside the box indicates the median, and whiskers indicate the smallest (bottom) and largest (top) non-outlier in the data. Source numerical data are available in extended and source data as well as in data repositories (see also the webpage associated with this study).

To further assess how Pantr1 impacts chromatin structure, we carried out in situ Hi-C. We found expansion of euchromatic compartment A (Fig. 6a,b) and an increase in long-range interactions (>10 Mb; Fig. 6c) accompanied by the loss of architectural loops and TAD boundary strengths in the NS cells lacking Pantr1 (Fig. 6b–f). ChIP-seq revealed that more sites lowered than enhanced CTCF signal in the knockout compared with wild-type NS cells (Fig. 6i and Methods), and sites lacking CTCF binding in Pantr1^−/−^ NS cells were high-occupancy CTCF peaks (Fig. 6j). Hence, like Ddx5, Pantr1 stabilizes architectural loops and CTCF binding to its strong sites in the neural cells.

Interestingly, we found that the effect on CTCF clustering in the nucleoplasm was minor in Pantr1^−/−^ NS cells compared with the changes induced by the loss of RBPs. Notably, Pantr1 loss led to an increase in the interactions between CTCF and the nuclear rim (Fig. 6k), indicating a redistribution of CTCF protein in the nucleoplasm when this lncRNA is gone.

**Extended Data Fig. 7:**
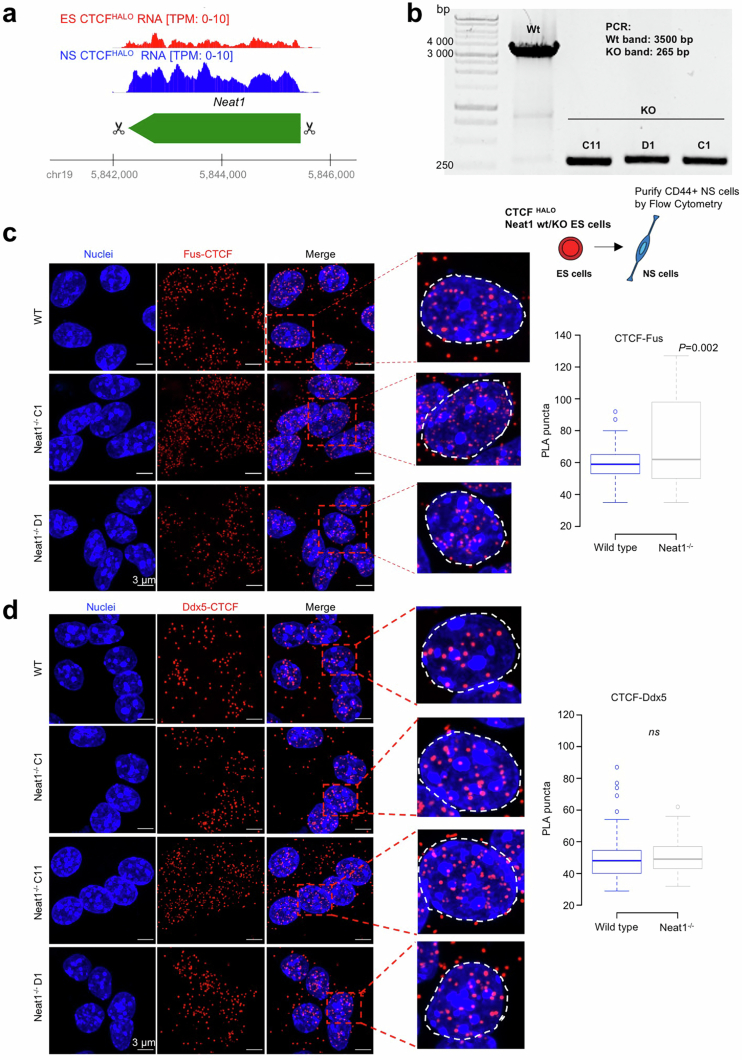
Relates to Fig. 5| Neat1 removal does not affect CTCF-Fus nor CTCF-Ddx5 interactions. **a**. Removal of Neat1 gene in the CTCF^HALO^ ES cells using CRISPR-Cas9. Left: Neat1 locus. The positions of sgRNAs are indicated with scissors. **b**. Genotyping results of Neat1^−/−^ clones confirming removal of the entire locus, experiment was performed two times. **c**. The association between CTCF and Fus in NS cells is not affected by the removal of Neat1. Left: a representative example of proximity ligation assay (PLA) readout in NS cells (CTCF-Fus interaction, Zeiss LSM800 confocal microscope with Airyscan mode, λex 594 nm; λem 624 nm). Right: boxplot showing the distribution of the per nucleus number of CTCF-Fus PLA puncta in NS cells (*P* = 0.002, two-sided t-test N_ESnuclei_ = 30, N_NSnuclei_ = 28; box spans first and third quartile, line inside the box indicates median, whiskers indicate smallest (bottom) and largest (top) non-outlier in the data). **d**. The association between CTCF and Ddx5 in NS cells is not affected by the removal of Neat1. Left: a representative example of proximity ligation assay (PLA) readout in NS cells. Right: boxplot showing the distribution of the per nucleus number of CTCF-Ddx5 PLA puncta in NS cells (*P* = 0.502, two-sided t-test, non-significant, N_ESnuclei_ = 30, N_NSnuclei_ = 28; box spans first and third quartile, line inside the box indicates median, whiskers indicate smallest (bottom) and largest (top) non-outlier in the data). Source numerical data and unprocessed gel are available in source data as well as in data repositories (see accession codes and the webpage associated with this study).

Next, we sought to check whether other lncRNAs that interact with CTCF could also impact CTCF-RBP interactions in NS cells. Nuclear Enriched Abundant Transcript 1 (Neat1) was shown to interact with Ddx5 and Fus^105,106^. It can also be co-purified with CTCF^61^. Like Pantr1, Neat1 is transcriptionally upregulated in the NS cells (Fig. 5c). To test whether Neat1’s presence in the NS cells would affect CTCF–Ddx5 and CTCF–Fus contacts, we obtained Neat1^−/−^ ES cells and differentiated them to NS cells (Extended Data Fig. 7a,b). PLA revealed no differences in the frequency of CTCF–Ddx5 and CTCF–Fus interactions between wild-type and Neat1^−/−^ NS cells (Extended Data Fig. 7c,d). Thus, Pantr1 appears to act as a specific amplifier of Ddx5–CTCF and Fus-CTCF interactions in the NS cells.

Summarizing our data thus far, RBPs affect CTCF binding to DNA and its capacity to form clusters and long-range architectural loops, hallmarks of chromatin topology in differentiated cells. We show that CTCF–RBP interactions depend on the presence of RNA. We identify that lncRNA Pantr1 is central for RBP-mediated chromatin structuring in ES cell differentiation to neural cells.

## Increase in insulatory role of CTCF upon neural induction

**Fig. 7:**
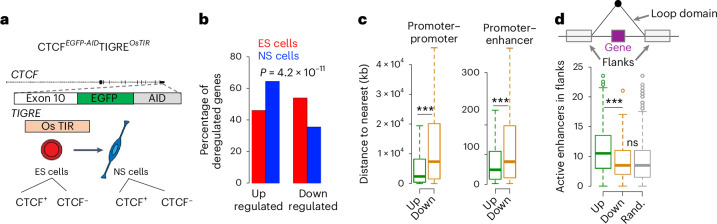
Gain of architectural functions of CTCF upon neural induction of ES cells. **a**, The experimental design to assess the impact of CTCF removal on chromatin activity and gene expression in ES and NS cells. CTCF degradation in the CTCF-AID cells is induced by treatment with an auxin analogue IAA (the ES cells are from ref. ^31^). **b**, Removal of CTCF more frequently leads to gene upregulation than downregulation in the NS cells. An opposite effect is seen in the ES cells, where CTCF loss leads primarily to gene downregulation (*P* = 4.2 × 10^−11^, Fisher’s exact test). **c**, Genes that feature increased expression upon CTCF removal in NS cells tend to reside at a shorter genomic distance from each other and from active enhancers (promoter–promoter, *n*_up_ = 358, *n*_down_ = 195, Kolmogorov–Smirnov test *P* = 3.4 × 10^−7^; promoter–enhancer, *n*_up_ = 358, *n*_down_ = 198, two-sided Kolmogorov–Smirnov test *P* = 5.4 × 10^−4^; the box spans the first and third quartiles, the line inside the box indicates the median, and whiskers indicate the smallest (bottom) and largest (top) non-outlier in the data). **d**, Genes upregulated upon CTCF removal are more frequently flanked by active enhancers than the downregulated or randomly sampled loci (analysis in the NS cells). Loop domains containing DEGs or randomly picked loci were considered in the analysis. Active enhancers (ATAC-seq peaks intersecting H3K27ac peaks located outside promoter regions) were counted in the 500-kb flanks of the two loop anchors (schematic above the box plot; ***P < 0.001, two-sided, Kolmogorov–Smirnov test). Loop annotation based on the in situ Hi-C data from ref. ^10^ (each box spans the first and third quartiles, the line inside the box indicates the median, and whiskers indicate the smallest (bottom) and largest (top) non-outlier in the data; *n*_up_ = 219, *n*_down_ = 125 and *n*_random_ = 1,690). Source numerical data are available in extended and source data as well as in data repositories (see accession codes and the webpage associated with this study). Random, random.

**Extended Data Fig. 8:**
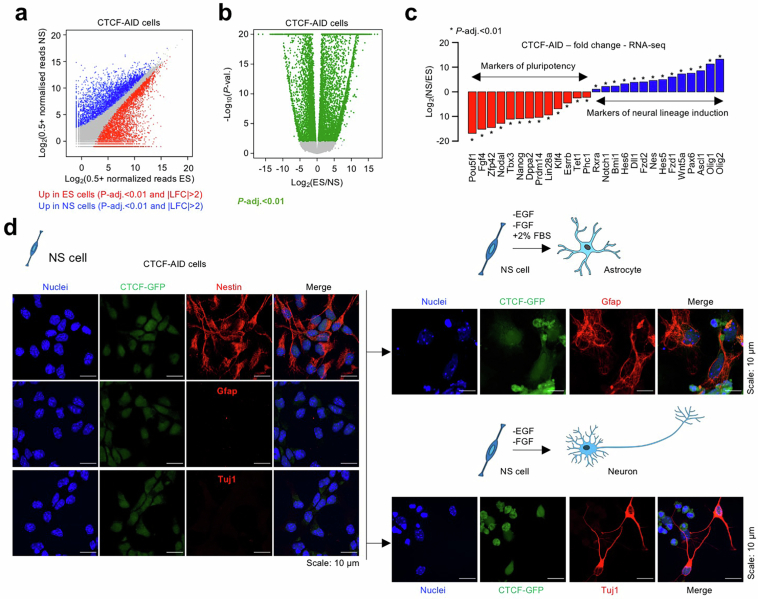
Relates to Fig. 6| Gain of architectural functions of CTCF upon neural induction of ES cells translates to enhanced insulator role of CTCF. **a**. Neural induction of the CTCF-AID ES cells leads to profound changes in the transcriptome. RNA-seq data was normalized using *DESeq2*. **b**. Volcano plot for *DESeq2* analysis to identify differentially expressed genes upon ES-to-NS transition of the CTCF-AID cells (*P-adj. < *0.01)*. **c**. *DESeq2*-normalized Log_2_ (fold change) of gene expression of a panel of pluripotency and neural lineage markers in CTCF-AID ES and NS cells (*P-adj. < *0.01)*. **d**. Characterisation of CTCF-AID NS cells. Immunofluorescence-based detection of Nestin (NS cell marker), Tuj1 (neuronal marker) and Glial Fibrillary Acidic Protein (GFAP, astrocyte marker) in NS cells grown in the presence of EGF and FGF, which favour the self-renewal of the NS cells and therefore their undifferentiated state (left). Detection of Tuj1 and GFAP in the NS cells that were differentiated into astrocytic lineage (withdrawal of EGF, FGF, the addition of 2% Foetal Bovine Serum; top right panel) or neuronal lineage (removal of EGF and FGF). Zeiss LSM800 confocal microscope with Airyscan mode was used to acquire the images. Source numerical data are available in source data as well as in data repositories (see accession codes and the webpage associated with this study) experiment was performed three times.

RNA–RBP-mediated consolidation of chromatin topology accompanying development strongly suggests fundamental changes in the functionality of CTCF in differentiation and the gain of insulator function of CTCF upon loss of pluripotency. To address this proposal experimentally, we measured the impact of CTCF removal on gene expression in ES and NS cells. We considered a previously established CTCF-AID ES cell line amenable for an acute removal of CTCF protein upon administering auxin, indole acetic acid (IAA) to the culture medium^31^ (Fig. 7a and Methods). Using the abovementioned procedure, we obtained a highly homogeneous population of CTCF-AID NS cells (Extended Data Fig. 8a–c) that robustly upregulate NS cell markers (Extended Data Fig. 8a–c). The CTCF-AID NS cells differentiated into Tuj1^+^ neurons and GFAP^+^ astrocytes, which validated their multipotent precursor identity (Extended Data Fig. 8d).

**Extended Data Fig. 9:**
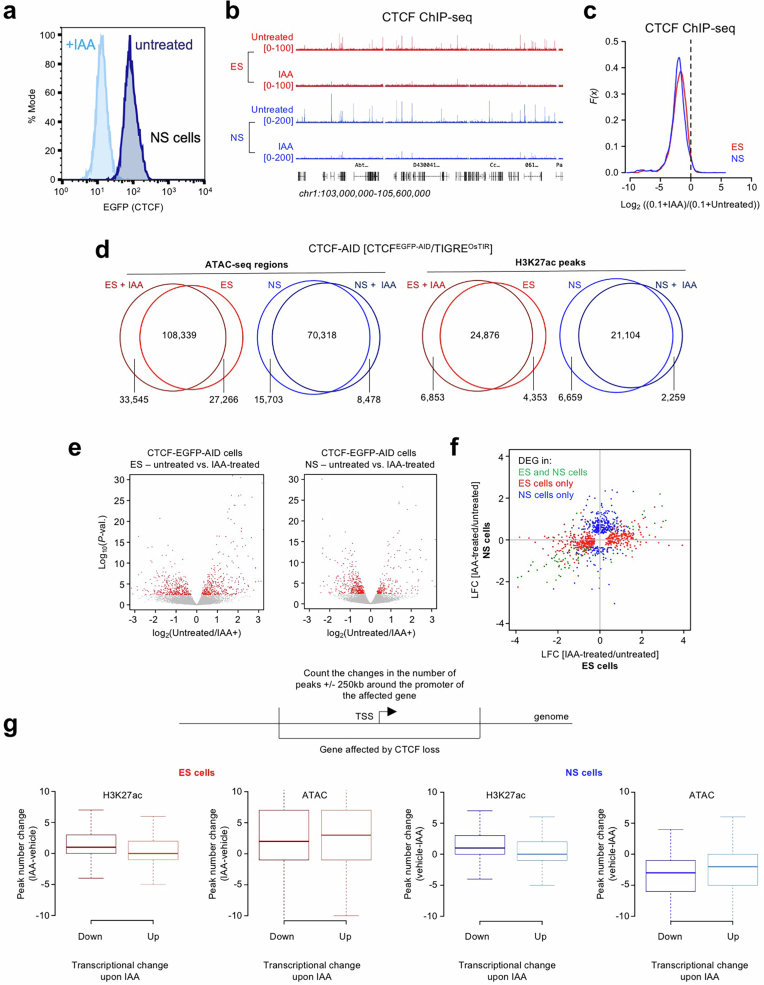
Relates to Fig. 7| Gain of architectural functions of CTCF upon neural induction of ES cells translates to enhanced insulator role of CTCF. **a**. CTCF removal in the CTCF-AID NS cells. Green fluorescence reports on the level of CTCF before and after the IAA (auxin analogue) treatment. CTCF is efficiently removed upon 24-hour exposure to IAA. **b**. RPGC-normalized ChIP-seq tracks of CTCF enrichment in ES and NS CTCF-AID cells in the presence and absence of IAA. **c**. Log2 fold change of CTCF signal upon IAA treatment in ES and NS CTCF-AID cells. Peak coordinates of CTCF in the untreated conditions were considered, and the RPGC normalized signal was integrated around the peak summits (+/− 50 base pairs) in the treated and untreated cells. **d**. Removal of CTCF does not lead to overt changes in chromatin openness and H3K27ac peaks in ES and NS cells. Venn diagrams compare peak sets identified in the untreated and IAA-treated cells. **e**. Volcano plot of *DESeq2* analysis of RNA-seq data of untreated and IAA-treated ES and NS CTCF-AID cells. Red – *P*-adj. < 0.1. ES and NS cell conditions were analysed separately. **f**. Comparison of the transcriptional effects of CTCF removal in ES and NS cells (Log2 fold change of gene expression was computed using *DESeq2*). DEGs identified in at least one comparison were considered (*P*-adj. < 0.1). Blue – genes affected by CTCF removal in the NS cells only; red – genes affected by the CTCF removal in the ES cells only; green – genes affected in the two comparisons. **g**. Relationship between ATAC-seq and ChIP-seq peak changes and differential gene expression upon CTCF removal. Net number of peaks in 500kb intervals centred around TSS of genes differentially expressed upon CTCF removal are displayed for ES and NS cells (ES: H3K27ac_down_ = 418, H3K27ac_up_ = 357; ATAC_down_ = 418, ATAC_up_ = 357; NS: H3K27ac_down_ = 198, H3K27ac_up_ = 358; ATAC_down_ = 198, ATAC_up_ = 358; box spans first and third quartile, line inside the box indicates median, whiskerers indicate smalest (bottom) and largest (top) non-outlier in the data). Source numerical data are available in source data as well as in data repositories (see accession codes and the webpage associated with this study).

To capture the effects of CTCF loss on gene regulation, we incubated the ES and NS for 24 h with IAA^31,107^ (Extended Data Fig. 9a–c). As anticipated^31,108^, acute removal of CTCF did not exert a global and pronounced impact on neither chromatin openness nor the level of H3K27ac, which marks active promoters and enhancers^109,110^ (Extended Data Fig. 9d). Thus, transcriptional effects of IAA treatment will primarily arise due to loss of architectural functions of CTCF (see also below).

RNA sequencing (RNA-seq) in control and the IAA-treated cells revealed 1,250 differentially expressed genes (DEGs; *P*-adj. < 0.1, DESeq2 method; Extended Data Fig. 9e,f and Supplementary Table 2): 775 loci featured altered expression in the ES cells. By comparison, 556 genes were affected by the IAA treatment in the NS cells (Supplementary Table 2).

**Extended Data Fig. 10:**
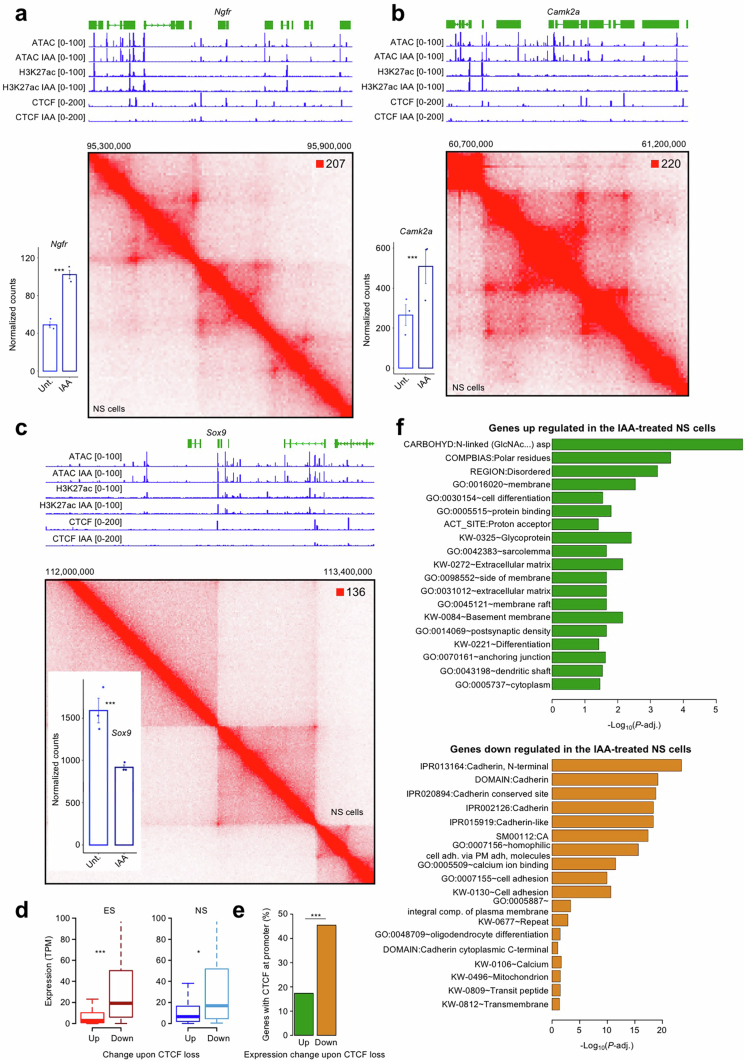
Relates to Fig. 7| Gain of architectural functions of CTCF upon neural induction of ES cells translates to enhanced insulator role of CTCF. **a**. Hi-C (data from ref. ^56^) and chromatin activity profiles (ChIP-seq and ATAC-seq profiles – this study) at the Nerve growth factor receptor (*Ngfr*) locus in NS cells. ChIP-seq and ATAC-seq were performed in NS cells derived from the CTCF-AID ES cells in the presence and absence of IAA. Three independent experiment was performed. Left panel: bar graph depicting the normalized expression of *Ngfr* in the untreated and IAA-treated NS cells (*** *P* < 0.01, *P* = 1.3 × 10^−5^; *DESeq2* method). **b**. Chromatin structure and activity at Calcium/Calmodulin Dependent Protein Kinase II Alpha (*Camk2a*) locus. Three independent experiment was performed. Analysis analogous as in panel **a**. Left panel: bar graph depicting the normalized expression of *Camk2a* in the untreated and IAA-treated NS cells (*** *P* < 0.01, *P* = 1.4 × 10^−3^; *DESeq2* method). **c**. Chromatin structure and activity at SRY-Box Transcription Factor 9 (Sox9) locus. The analysis is analogous to the one presented in panel **a** (*** *P* < 0.01; *P* = 3 × 10-8, *DESeq2* method). **d**. Box-plot of the transcript per million sequenced read pairs (TPM) normalized expression level of genes that are either up- or down-regulated upon CTCF loss in the ES (left) and NS (right) cells (Three independent experiment was performed. Box spans first and third quartile, line inside the box indicates median, whiskerers indicate smalest (bottom) and largest (top) non-outlier in the data; *P = 0.012; ***P = 2.1 × 10^−7^). **e**. Percentage of genes that are up- or downregulated upon CTCF loss in the NS and that feature a CTCF binding site at its promoter (Fisher’s exact test, *P* = *2.4* × *10*^−*7*^). **f**. Enrichment of ontologies for sets of genes featuring an increased (green) or decreased (orange) expression upon CTCF removal in the NS cells (*P*-adj. < 0.1, *DESeq2* method). Analysis was performed using DAVID tool (https://david.ncifcrf.gov/). Source numerical data are available in source data as well as in data repositories (see accession codes and the webpage associated with this study).

Remarkably, while there were more downregulated than upregulated genes upon CTCF removal in the ES cells, in the NS cells, the loss of CTCF led to significantly more gene activation than repression (Fig. 7b, odds 2.12, *P* = 4.2 × 10^−11^, Fisher’s test). Multiple additional analyses suggested that gene activation upon IAA treatment reflected aberrant exposure of genes to enhancers. Indeed, peaks of H3K27ac and chromatin openness were not affected by CTCF removal (Extended Data Figs. 9g and 10a–c) compared with genes downregulated in the IAA-treated NS cells; genes activated upon CTCF loss in NS cells not only were expressed at a significantly lower level (Extended Data Fig. 10d) but were also at shorter genomic distances from each other (Fig. 7c, Kolmogorov–Smirnov test, *P* = 3.4 × 10^−7^) and from enhancers active in the NS cells (Fig. 7c, Kolmogorov–Smirnov test, *P* = 5.4 × 10^−4^). Importantly, loop domains (genomic interval between anchors of an architectural loop) that contained the promoters of genes that featured increased expression in the IAA-treated cells were flanked by, on average, 12 enhancers active in the NS cells in contrast to 8 found around loop domains embedding promoters of genes downregulated upon IAA treatment or randomly sampled promoters (Fig. 7d, two-sided *t*-test, *P* = 6 × 10^−6^). Likewise, considering the 81 genes commonly deregulated in the two cell types, the majority (59/81, 73%) were downregulated in the absence of CTCF (Extended Data Fig. 9f). As anticipated^31^, promoters of genes activated upon CTCF loss featured CTCF binding two times less frequently than the upregulated loci (Extended Data Fig. 10e, Fisher’s exact test, *P* = 2.4 × 10^−7^). Thus, gene downregulation upon IAA treatment appears to primarily reflect the role of the promoter-bound CTCF. By contrast, gene activation following CTCF removal appears to result from the aberrant exposure of promoters to active enhancers.

**Fig. 8:**
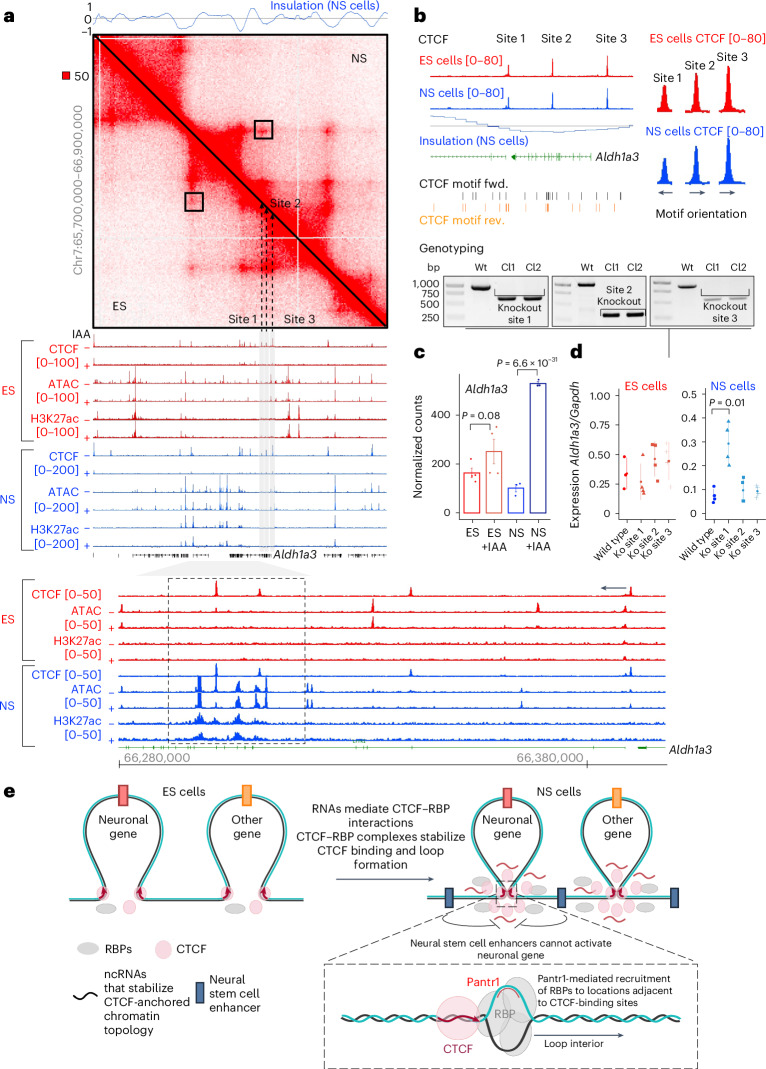
Gain of architectural functions of CTCF at Aldh1a3 locus and the model. **a**, Enhancement of CTCF–CTCF loops at the Aldh1a3 locus upon the ES-to-NS cell transition. Hi-C^10^ and chromatin activity profiles (RPGC-normalized in this study) at the Aldh1a3 locus in the control and IAA-treated ES and NS cells (red and blue, respectively). Insulation is displayed above the interactome plot. Three CTCF sites are close to the Aldh1a3 gene (grey boxes). Black-box: CTCF-anchored loop featuring an increase in Hi-C signal upon ES-to-NS transition; dashed lines: CTCF binding sites at the Aldh1a3 locus; grey area: region intersecting putative regulatory elements at the Aldh1a3 locus (zoomed in in the panel below); black arrow indicates the orientation of the CTCF motif at the CTCF binding site within the 3′ end of the Aldh1a3 locus. **b**, Zoom on Aldh1a3 locus together with the profile of CTCF binding and motif (fwd., forward; rev., reverse) orientation in ES (top) and NS (bottom) 46C cells. The three CTCF sites (sites 1, 2 and 3) were removed individually in the ES cells using CRISPR–Cas9 (bottom; two clones per region were obtained, validation of the deletion was performed twice). The ES cells were differentiated into NS cells. **c**, RNA-seq inferred expression of Aldh1a3 in untreated and IAA-treated CTCF-AID cells. *P* values were obtained with the DESeq2 method (ES, *P* = 0.08; NS, *P* = 6.6 × 10^−31^). **d**, qRT–PCR-based assessment of Aldh1a3 expression in the wild-type and CTCF-binding site-knockout ES and NS cells (*P* *=* 0.01, two-sided *t*-test; see **a** for an annotation of CTCF sites and genotyping; individual points indicate replicates in the qRT–PCR reactions). **e**, Model. Loss of pluripotency is linked with a gain of expression of RNAs that interact with CTCF, including Pantr1. Pantr1 RNA associates with DNA close to the CTCF motif within CpG-rich sequences. The sites also feature high propensity to form G4q (not displayed for simplicity). The Pantr1 locations close to CTCF locations attract RBPs that probably form local protein aggregates (perhaps phase-separated condensates), which may slow down cohesin, thereby stabilizing loop formation and consolidating TAD borders and chromatin structure in differentiation. A robust insulator function of CTCF in the NS cells is key to restraining the expression of neuronal genes, which would otherwise be efficiently upregulated by enhancers active in the NS cells. Source numerical data are available in extended and source data as well as in data repositories (see accession codes and the webpage associated with this study).

To validate our predictions further, we sought to test the contribution of individual CTCF binding sites at loci upregulated upon CTCF loss. Gene encoding Aldehyde Dehydrogenase 1 Family Member a3 (Aldh1a3) is located within a domain demarcated by anchors of loops that strengthen upon the ES-to-NS transition (Fig. 8a,b). While in both cell types CTCF loss did not affect chromatin openness nor the H3K27ac enrichment at Aldh1a3 locus (Fig. 8a), CTCF removal enhanced the expression of Aldh1a3 gene, yet only in the NS cells (Fig. 8c). ChIP-seq revealed three CTCF-binding sites proximal to the Aldh1a3 promoter; analysis of Hi-C data showed that sites 1 and 2 featured architectural functions, while site 3 did not seem to anchor loops (Fig. 8a,b). We removed each of these sites using CRISPR–Cas9 (Fig. 8b). None of the deletions impacted Aldh1a3 expression in the ES cells. Removal of site 1 led to a 2.4-fold upregulation of Aldh1a3 mRNA level in NS cells (Fig. 8d), while sites 2 and 3 did not regulate Aldh1a3 expression in the NS cells. Thus, the dynamic gain of insulator competence at CTCF-binding site 1 is reflected by an increased strength of the loop anchored by this CTCF peak. These data are consistent with the degron experiments and further validate the proposal that the insulator action of CTCF at the Aldh1a3 locus is enhanced upon loss of pluripotency.

## CTCF shapes NS cell transcriptome

To address the possible functional importance of the gain of chromatin insulation in the ES-to-NS transition, we compared genes affected by the removal of CTCF in the two cell types. DEGs were primarily cell type specific; only 81 loci (6.5%; Extended Data Fig. 9f) were scored as DEGs in both ES and NS cells, which is lower than the number expected by chance (29% of genes featured similar expression in the ES and NS cells, Fisher’s *P* < 2.2 × 10^−16^). Thus, CTCF regulates the expression of cell-type-specific sets of genes.

During the development of the nervous system, NS cells initially differentiate into neurons. Subsequently, upon the so-called glial switch, the NS cells acquire the capacity to generate astrocytes and, ultimately, oligodendrocytes^111^. Gene Ontology analysis revealed that CTCF removal in the NS cells led to an enhanced expression of genes related to cell differentiation (GO:0030154, *P*-adj. = 2.9 × 10^−^^2^; Extended Data Fig. 10a–d), including factors promoting neuronal cell fate such as nerve growth factor receptor^112^ (*Ngfr*; Extended Data Fig. 10a), distal less homeobox 1 and 2 (*Dlx1* and *Dlx2*), noggin (*Nog*) and genes related to postsynaptic density (GO:0014069, *P*-adj. = 2.2 × 10^−2^), including calcium/calmodulin-dependent protein kinase II alpha (Camk2a; Extended Data Fig. 10b). By contrast, the downregulated group was enriched in genes implicated in oligodendrocyte development (GO:0048709, *P*-adj. = 3.5 × 10^−2^; Extended Data Fig. 10a), including *Sox9* (Extended Data Fig. 10c) and *Olig1*. Furthermore, the group of downregulated genes featured loci coding cell adhesion proteins (*n* = 28, GO:0007155, *P*-adj. = 1.2 × 10^−10^), primarily cadherins (*n* = 21, *P*-adj. = 3.5 × 10^−24^), which is consistent with the known role of CTCF in the regulation of cadherin gene expression^113^. Hence, CTCF contributes to regulating cell-type-specific genes implicated in the development of the nervous system. Upregulation of neuronal genes and downregulation of pro-glial loci upon CTCF removal in the NS cells strongly suggests that CTCF contributes to the control of the proper timing of gene expression in the developing nervous system.

Altogether, we posit a model describing the mechanism and the functional contribution of chromatin architectural consolidation upon exit from pluripotency (Fig. 8e). ES cell differentiation leads to transcriptional upregulation of Pantr1, a ncRNA partner of CTCF that mediates the interactions between CTCF and RBPs. The CTCF–RBP–RNA interactions help foster CTCF clustering, DNA binding and chromatin loop stability in the lineage-committed cells. The resulting robust insulator activity in the NS cells blocks the untimely expression of neuronal genes in the NS cells, thereby contributing to cell fate control during development.

## Discussion

Pluripotent stem cells exhibit a unique chromatin signature, characterized by weaker CTCF-anchored loops and TAD boundaries and more dynamic association between architectural proteins and DNA than lineage-committed cells^9,10,114^. Here, we investigated the mechanisms and the functional role of the consolidation of CTCF-centred chromatin architecture in differentiation.

We reveal a global increase in interactions between CTCF and RBPs, including DEAD-box helicase Ddx5, known to modulate CTCF functions in differentiated cells^85,90^, and on Fus, which has not been previously linked to CTCF in development. We find that Ddx5 and Fus are, by and large, dispensable for CTCF clustering and binding to DNA in the pluripotent cells. By contrast, in NS cells, RBPs stabilize CTCF clustering and binding to DNA and favour loop formation. The general effect of RBPs on chromatin architecture echoes the previous data showing global loop enhancement upon loss of pluripotency. We identified lncRNA Pantr1 as the mediator of the increase in CTCF–RBP interactions and chromatin architectural consolidation upon ES-to-NS cell transition. Pantr1 affects numerous loops in the NS cells, yet not all these interactions are dependent on the presence of this lncRNA. We hypothesize that other RNA partners of CTCF may enhance its architectural activity, which collectively results in the consolidation of chromatin topology in differentiation (Fig. 8). Understanding how the composition of RNA–RBP–CTCF complexes affects chromatin insulation in development remains a critical area of investigation.

How do RNA and RBPs impact CTCF functions mechanistically? The CG-rich DNA motifs bound by CTCF are prone to forming secondary structures, including G-quadruplexes (G4q)^97,98^, some of which also favour R-loop formation^98^. When present in the vicinity of CTCF-bound sites, these structures may foster CTCF binding^98,102,115^ and slow down cohesin-mediated loop extrusion^115^, thereby impacting chromatin structure. However, when G4q are formed at the CTCF recognition site, they are probably incompatible with CTCF binding^95,98^ (although some reports suggest otherwise^97,116^). The RNA helicase Ddx5 counteracts G4q and R-loop formation^117,118^. As Ddx5 knockout leads to weakened CTCF-anchored loops and CTCF–DNA interactions, our data suggest a dual role for Ddx5: dismantling G4q to facilitate CTCF motif recognition and modulating R-loop formation at sites adjacent to the CTCF motif. The G4q-enhanced R-loop formation involving Pantr1 RNA may serve as a recruitment platform for RBPs to loop anchors. Indeed, RBPs, including Fus and Nono, massively co-purify with R-loops in vivo^116^. Furthermore, many RBPs, including Fus, contain intrinsically disordered regions that facilitate biomolecular condensate formation. Hence, we propose that Pantr1-driven R-loop formation in the vicinity of CTCF-binding sites and the related G4q help recruit RBPs to loop anchors in NS cells, thereby fostering chromatin topology. One can imagine that the protein-rich aggregates near CTCF-binding sites slow cohesin movement, leading to more stable architectural loops^97,115,116^. However, this mechanism requires further examination; genome topology engineering experiments could provide insights in this direction. Enforcing loop formation using CRISPR–dCas9 revealed that Ddx5 is recruited to sites involved in stable chromatin interactions^119^. Notably, while ectopic loop formation was possible in the absence of Ddx5, its long-term maintenance required the presence of the helicase^119^, indicating the role of Ddx5 in the regulation of loop stability. Given that the CRISPR–Cas9 system relies on the formation of DNA–RNA hybrids, it appears that R-loops and RBPs are central for loop stabilization during differentiation.

How RNA modulates CTCF functions is not fully understood. Direct CTCF–RNA interactions have been proposed^58,61,85–87,100,102–104^. However, recently, Guo et al. used denaturing purification conditions, thereby revealing that CTCF does not bind RNA directly^120^. Hence, the CTCF–RNA interactions are robustly captured upon cross-linking or in permissive wash conditions, reinforcing the idea that these interactions occur within the same condensates rather than through direct RNA binding. This view reconciles the published observations and highlights the role of RNAs in guiding RBPs to specific sites in the genome.

Intergenic CTCF-binding sites insulate promoter–enhancer interactions, shaping the specificity of the dialogue between *cis*-regulatory elements^24,121,122^. Consistent with this, loop domains consolidated upon the ES-to-NS transition frequently demarcate promoter–enhancer pairs active in mature neurons^9^. We find that genes activated upon acute CTCF loss in NS cells are embedded in enhancer-rich neighbourhoods. Thus, the gain in their expression probably entails loss of insulator activity upon CTCF degradation. Our genome editing experiments support this model. Notably, genes upregulated upon CTCF loss in the NS cells are related to neuronal functions, suggesting that CTCF-mediated insulation helps suppress genes normally expressed later in development. At later developmental stages, dedicated enhancers within loop domains would ensure precise gene expression control. Our data also suggest that CTCF plays a role in balancing the pro-neuronal and pro-glial developmental potential of NS cells, although its function in the glial switch requires further investigation.

Heterozygous CTCF loss is linked to mental retardation^36–38,123^. Interestingly, neuronal cells frequently establish long-range loops that involve neuropsychiatric disease loci^44^. Whether neural lineage requires long-range loop formation to a greater extent than other lineages remains an open question that is worthwhile addressing in the future.

## Methods

### Cell lines

All the ES cell lines used in this study are derivatives of the E14 and E14Tg2a mouse ES cells.

The CTCF^HALO^ line was obtained by J.X. and R.C. using the ATCC CRL1821 line. The 46C ES cell line (SOX1-GFP-puro, PMID: 12524553)^124^ was a gift from A. Smith, University of Cambridge, and CTCF-AID-GFP^31^ ES cell line was a gift from E. Nora and B. Bruneau, Gladstone Institute (#EN52.9.1 PMID: 28525758). The Ddx5^−/−^, Fus^−/−^, Pantr1^−/−^, Neat1^−/−^ and Ddx5^FKBP^ knock-in (KI) cells were obtained using the CTCF^HALO^ ES cells. Knockout of CTCF sites at the Aldh1a3 locus was carried out using 46C ES cells.

### ES cell culture in standard conditions (FBS/LIF)

The ES cells were grown on 0.2% (v/v) gelatin-coated (Sigma-Merck, G9391-100G) culture plastic in ES cell culture medium (Glasgow Minimum Essential Medium (Invitrogen, 11710035) supplemented with 10% (v/v) EmbryoMax ES Cell Qualified FBS (Sigma-Merck, ES-009-b), 2 ng ml^−1^ LIF (EMBL, Protein Expression, and Purification Core Facility), 1 mM 2-mercaptoethanol (Sigma-Merck, 615226), supplemented with non-essential amino acids (Thermo Fisher, 11140035), l-glutamine (Thermo Fisher, A2916801) and Na-pyruvate (Thermo Fisher, 11360070) according to the manufacturer’s recommendation. Cells were maintained at 37 °C in 5% (v/v) CO_2_. Cells were detached from the plastic using Accutase (Sigma-Merck, A6964) and routinely split at a density of 30,000 cells cm^−2^ every 48 h. The medium was exchanged daily.

### ES cell culture in chemically defined conditions (2i/LIF)

ES (2i/LIF) cells were cultured in a serum-free medium composed of Dulbecco’s modified Eagle medium–Nutrient Mixture F-12 (DMEM–F12) (Thermo Fisher, 31331028) 0.5× N2 (Thermo Fisher, 17502048) and 0.5× B27 (Thermo Fisher, 17504044) (2.5 and 5 ml supplements per 500 ml respectively), 0.012% bovine serum albumin fraction V (BSA) (Thermo Fisher, 15460037), 1% non-essential amino acids (Thermo Fisher, 11140035), 0.03 M d-(+)-glucose (Sigma-Merck, G8270-1KG), 4.5 mM HEPES (Thermo Fisher, 15630056) and 0.1 mM β-mercaptoethanol (Sigma-Merck, 615226). The culture medium was further supplemented with 3 mM GSK3 inhibitor CHIR99021 (Reagent Direct, 27-H76), 1 mM MEK inhibitor PD0325901 (Reagent Direct, 39-C68) and 2 ng ml^−1^ LIF (EMBL, Protein Expression and Purification Core Facility). Cells propagated for at least four but fewer than ten passages in the 2i/LIF conditions were considered.

### NS cell differentiation and culture

ES cells grown in the presence of FBS were plated at a density of 15,000 cells cm^−2^ gelatin-coated culture plastic in neural differentiation medium comprising DMEM–F12 (Thermo Fisher, 31331028) supplemented with 0.5× of N2 (Thermo Fisher, 17502048) and B27 (Thermo Fisher, 17504044), 0.012% BSA (Thermo Fisher, 15460037), non-essential amino acids (Thermo Fisher, 11140035), 0.03M d-(+)-glucose (Sigma-Merck, G8270-1KG), 4.5 mM HEPES (Thermo Fisher, 15630056) and 0.1 mM β-mercaptoethanol (Sigma-Merck, 615226). The medium was exchanged every 24 h for 6 days. Cells were dislodged using Accutase (Sigma-Merck, A6964) and seeded onto a laminin-coated surface (10 μg cm^−2^ laminin, minimum 4 h coating time at 37 °C, Sigma-Merck, L2020-1MG). Following the detachment, cells were grown in a neural differentiation medium supplemented with recombinant murine EGF (EMBL, Protein Expression and Purification Core Facility) and bFGF (EMBL, Protein Expression and Purification Core Facility) to a final concentration of 10 ng ml^−1^. Cells were split at 80% confluence. The medium was exchanged daily.

### NS cell differentiation to neurons and astrocytes

NS cells grown with growth factors (EGF and FGF, 10 ng ml^−1^) were seeded at a density of 50,000 cells cm^−2^ on laminin-coated cell culture plastic. For neuronal differentiation, cells were allowed to spontaneously differentiate via withdrawal of growth factors in N2B27-supplemented medium, whereas, for astrocyte differentiation, cells were grown in N2B27-supplemented medium in the presence of 2% FBS. The medium was exchanged daily. The differentiation was carried out for 7 days.

### Purification of CD44-expressing NS cells

To obtain a homogenous population of wild-type as well as Ddx5^−/−^ or Fus^−/−^ or Pantr1^−/−^ neural progenitors, cells expressing CD44 were purified using flow cytometry^125^. In brief, NS cells were detached from the culture plastic using Accutase. Then, the cell pellet was washed once with phosphate-buffered saline (PBS). Cells were then incubated with blocking buffer (0.5% BSA–PBS) for 30 min at 4 °C. The cells were washed with Dulbecco’s PBS (DPBS) once and incubated with the anti-CD44 antibody (1:200 BD Pharmingen PE Rat Anti-Mouse CD44, 553134) for 40 min at 4 °C. The cells were washed twice with DPBS. CD44-positive cells were selected using BD FACSDiva software (version 8.0.1), and the purified cells were sorted with a BD FACSAria II cell sorter.

### Auxin-induced degradation of CTCF

CTCF-AID-GFP ES and NS cells were seeded at their respective densities. After 24 h, cells were incubated with 500 μM of IAA (Sigma-Merck, I5148-2G) diluted in the respective cell culture medium to induce CTCF degradation for 24 h at 37 ºC. Cells were detached using Accutase and washed once with PBS, and CTCF depletion was assessed with a BD FACSCalibur flow cytometer or used for assay for transposase-accessible chromatin using sequencing (ATAC-seq), ChIP-seq or RNA-seq library preparation. Flow cytometer data were analysed using FlowJo software (version 10.8.1).

### dTAG13-induced degradation of Ddx5

Ddx5^FKBP^ ES and NS cells were seeded at a density of 35,000 cells cm^−2^. After 24 h, cells were incubated with 500 nM of dTAG13 (Torcis, 6605) diluted in the respective cell culture medium to induce Ddx5 degradation for 24 h at 37 °C. Subsequently, cells were detached using Accutase and washed once with PBS, and Ddx5 depletion was assessed with western blot analysis or used for Hi-C and ChIP-seq library preparation.

### Western blot analysis

Cells were dislodged using Accutase (Sigma-Merck, SCR005) and spun down for 3 min at 300*g*. Ice-cold RIPA buffer supplemented with 1× Complete Mini EDTA-free protease inhibitor cocktail (Roche, 11836170001) and Benzonase (1:2000 Merck, 014-5KU) was added to the cell pellet (100 μl RIPA per 1 million cells). After 30 min incubation on ice, the extracts were centrifuged for 20 min at 10,000*g* at 4 °C and the supernatant was collected and kept on ice.

Protein concentration was estimated using Pierce Coomassie Plus (Bradford) Assay Kit (Thermo Fisher, 23226) following the manufacturer’s recommendations. Protein lysates were mixed with 4× Laemmli Sample Buffer (Bio-Rad, 1610747) and boiled for 5 min at 98 °C. Next, 20 μg protein was resolved on SDS–PAGE gel (stacking 4% and resolving 10%) at 100 V for 2 h and transferred to a nitrocellulose membrane (0.2 μm, Bio-Rad, 1620112) at 100 V for 1.5 h at 4 °C. Membrane blocking was performed by incubating with either LICOR Intercept blocking buffer (Licor, 27-60001) or 5% milk prepared in Tris-buffered saline (TBS, Bio-Rad, 1706435) with 0.05% Tween-20 (Sigma-Merck, P1379-100ML) (TBS-T) for 1 h at room temperature (RT). The membrane was next incubated with the primary antibodies at the following concentrations: anti-CTCF (1:2,000, Cell Signaling Technology, 2899S), anti-Fus (1:10,000, Bethyl, A300-294A), anti-Ddx5 (1:5,000, Bethyl, A200-523A) anti-Nono (1:1,000, Proteintech, 11058-1-AP) and anti-β-actin (1:5000 Proteintech, 66009-1-Ig) in the LICOR Intercept blocking buffer overnight at 4 °C with shaking. The membrane was washed three times with TBS-T for 5 min followed by incubation with 1:15,000 secondary antibodies IRDye680 (Licor, 925-68070) and IRDye800 (Licor, 925-32211) at RT for 1 h. The membrane was washed three times with TBS-T for 5 min and visualized on the Chemidoc system (Bio-Rad), and blot images were quantified using the Image Studio Software version 6.0 (box plots were prepared in Microsoft Office Excel (https://microsoft.com) version 16.78.3).

### Co-immunoprecipitation experiments

We utilized anti-HALO M270 beads to efficiently capture HALO-tagged proteins. Buffers were prepared following the published approaches^85^ and the HALO-Trap Magnetic Particles M-270 (Product code: otd) protocol. In brief, cells were detached with Accutase and washed in the culture medium. The cells were then cross-linked with 0.2% formaldehyde in the culture medium for 10 min at RT. The reaction was quenched by adding glycine to the final concentration of 0.2 M; the suspension was incubated for 5 min at RT. The cells were then spun at 500*g* for 5 min at 4 °C and washed once with ice-cold PBS. Cells were then lysed with RIPA lysis buffer containing SUPERase•In RNase Inhibitor at 1 U μl^−1^ and incubated on ice for 30 min. The cell lysates were then spun at 15,000*g* for 10 min at 4 °C. Next, 10% of the sample was set aside and treated as input; the remaining sample was precleared with 15 μl of M270 beads on ice for 15 min (prewashing). HALO-tagged CTCF was pulled down by adding 25 μl of M270 beads for 1 h at RT with rotation. Beads were then collected on a magnet and washed three times with washing buffer for 5 min at RT. The beads were then resuspended in 1× Laemmli buffer and incubated at 95 °C for 5 min. Western blot was performed as above to determine the abundance of CTCF (anti-CTCF; CST 2899S) and Ddx5 and Fus (anti-Ddx5; A200-523A and anti-Fus; sc-47711). Blots were developed using the Bio-Rad Chemidoc imaging system, and blot images were quantified using the Image Studio Software version 6.0. Box plots were prepared in Microsoft Office Excel (https://microsoft.com) version 16.78.3.

### Isolation of chromatin-bound proteins

To extract the chromatin-bound protein from ES and NS cells, we used a subcellular protein fractionation kit for cultured cells from Thermo Fischer (78840). We followed the manufacturer’s protocol for sample preparation (https://assets.thermofisher.com/TFSAssets/LSG/manuals/MAN0011667_Subcellular_Protein_Fraction_CulturedCells_UG.pdf). All the steps were done on ice. In brief, cells were dislodged using Accutase, spun at 500*g* for 3 min and washed with ice-cold PBS. The cells were spun again at 500*g* for 3 min at 4 °C. Ice-cold CEB buffer was added to the cells, and the mix was incubated on ice for 10 min. The samples were centrifugated for 5 min at 500*g* at 4 °C, and the supernatant was removed. Then, the cell pellet was resuspended in ice-cold MEB and incubated for 10 min on ice. The samples were then spun at 3,000*g* for 5 min at 4 °C. The supernatant was discarded, ice-cold NEB was added and the cells were incubated on ice for 30 min (the cells were mixed by pipetting every 10 min to make sure the lysis occurred uniformly and efficiently). The samples were then spun at 5,000*g* for 5 min at 4 °C to extract the soluble nuclear fraction. The pellet was dissolved with NEB containing CaCl_2_ and micrococcal nuclease. The mix was incubated at RT for 15 min. After incubation, samples were mixed by vortexing and spun at 16,000*g*for 5 min. The supernatant was collected to obtain the chromatin-bound nuclear extract in a prechilled tube on ice. Then, 1× Laemmli buffer was added to the sample and incubated at 95 °C for 5 min. Samples were run on 10% SDS–PAGE followed by western blot analysis to detect the enrichment of chromatin-bound CTCF in ES and NS cells.

### 3D RNA-FISH

Custom Stellaris Quasar670-conjugated FISH probes were designed against Pantr1 by utilizing the Stellaris RNA FISH Probe Designer (Biosearch Technologies) available at www.biosearchtech.com/stellarisdesigner (version 4.2). The Pantr1 probe sequence is presented Supplementary Table 3.

CTCF^HALO^ ES and NS cells were seeded at a density of 50,000 cells cm^−2^ on 18-mm round coverslips. To stain CTCF, 24 h later, the cells were incubated with 5 µM TMR ligand (Promega, G8252) in the respective culture medium for 30 min at 37 °C in a 5% (v/v) CO_2_ incubator. Cells were washed with PBS twice for a brief period (5 min incubation) and once for 30 min at 37 °C. Following an established protocol^126^, cells were fixed with 3.7% formaldehyde for 10 min at RT in PBS, washed twice with PBS at RT and permeabilized with 70% ethanol for 1 h at 4 °C. Then, the coverslips were incubated with wash buffer A containing 10% formamide for 5 min at RT. Probe hybridization was carried out in hybridization buffer containing 10% formamide and 125 nM probes, in the dark for 16 h at 37 °C. Next, the cells were washed with wash buffer A, which included 10% formamide, for 30 min at 37 °C. Next, the cells were incubated with buffer B for 5 min at RT. Finally, the coverslips were mounted on slides with Vectashield antifade mounting medium containing 4′,6-diamidino-2-phenylindole (DAPI). Zeiss LSM800-based Inverted Axio Observer Z.1 with an AiryScan detector, Plan Apochromat 63×/1.4 oil differential interference contrast (DIC) objective and diode lasers 405, 561 and 670 nM were used to acquire consecutive images at a focal distance of 0.13 µm.

Image analysis was done using Fiji software version 2.1.0/1.53c. To remove background, we set one threshold to each channel. We used this channel-specific threshold for each image and removed values below the threshold value. Next, we built a *z* stack picture for fluorescence intensity in each channel. The fraction of Pantr1 puncta overlapping with CTCF-enriched regions was assessed manually for each nucleus in each picture. The analysis of individual planes yielded similar results. Box plots were prepared in Microsoft Office Excel (https://microsoft.com) version 16.78.3.

### Generation of CTCF^HALO^ ES cells

The single guide RNAs (sgRNAs) targeting a region upstream of the C-terminus of the CTCF gene were designed using an online tool (MIT CRISPR Designer, forward sequence: caccGCGTGAGGTCTCCGTTGG, reverse sequence aaacCCAACGGAGACCTCACGC) and cloned into pX330 CRISPR/Cas9 vector (Addgene). To construct a targeting vector for the HALO-tag KI, two homology arms corresponding to the 500-bp regions upstream and downstream of the C-terminus of the CTCF gene, respectively, were polymerase chain reaction (PCR) amplified from E14 ES cells DNA. HALO-tag DNA was PCR amplified from pHTC HALOTag CMV-neo vector (Promega), inserted between the two homology arms through ‘stitch PCR’ and then cloned into Zero-Blunt-Puro plasmid at the EcoRV site.

Two million E14 ES cells were electroporated with a mixture of 2 µg of pX330-sgRNA and 2 µg of targeting vector in 100 µl reaction using program A-030 (Lonza Mouse Embryonic Stem Cell Nucleofector Kit). Cells were cultured in 10-cm culture dishes (2i+LIF medium) for 2 days, then briefly selected with puromycin at 0.7 μg ml^−1^ for 4 days, followed by 2 days of culture without puromycin.

Individual ES cell colonies were picked into a 96-well plate for further culture, genotyping and sequencing to confirm HALO-tag insertion. Primers used for genotyping KI cells are as follows:

Ctcf_5′_Out_Fwd GAACCGCCCAGTCATTTCAC

Ctcf_3′_Out_Rv AACTTTGCCAAGAAAGAGGCA

Primers used for generating homology arms of targeting vectors are as follows:

Ctcf_5′arm_FwdAGGGCTGGATTTTTTTTTCCCTGCCC

Ctcf_5′arm_Rv (including silent mutations at the sgRNA recognition site) TGGCTCGAGGCTAGCtCGaTCCATCATaCTcAGaATCATtTCgGGgGTcAGaTCgCCaTTaGGaGCGTCTGTGGTGGCTGCCTGA

Ctcf_3′arm_FwdCGGTTAAGGCGCGCCTGCTGGGGCCTTGCTCGG

Ctcf_3′arm_RvTTCAGGACAGAAACTGATCGTAGCATGCC

linker_HTC_Fwd GCTAGCCTCGAGCCAACCACTGAGGATC

linker_HTC_RvGGCGCGCCTTAACCGGAAATCTCCAGAGTAGACAG

### Generation of Ddx5^FKBP^ ES cells

We used an sgRNA design tool (http://crispor.tefor.net) to design the guides targeting the Ddx5 locus region on chr11:106,779,390–106,789,735. The Ddx5-FKBP KI cassette was designed containing AM-tag, FKBP, RFP657 and HA-tag sequence and was obtained by DNA synthesis with Novogene. Homology arms were appended with stitch PCR. Genomic fragments were amplified using the following oligonucleotides:

5′arm_Fwd: GAAGGGTCGAACTCGGTC; 5′arm_Rv:ATAGGCCTGGCTCAGGATCACATTTCCCTTTCTCTGTGGGTCCTGGCCCATGGCGTCAATGGTGGCG;

3′arm_Fwd: GTGACAGGGATAGAGGACGCGATCGAGGGTGAGTGTGACAAGAG;

3′arm_Rv: GTGGGTTTATCAGGTGGCAAAC).

Two sgRNAs targeting 10 and 15 bp upstream of the 3′ end of exon 1 of the *Ddx5*gene were cloned into the BbsI and BsmbI sites of a modified pSpCas9(BB)-2A-GFP (PX458) (Addgene, #48138), which contains an ampicillin resistance gene, according to the Zhang Lab General Cloning Protocol (https://www.addgene.org/crispr/zhang/). The KI cassette was cloned to a donor plasmid (pMAX-GFP) harbouring a kanamycin resistance gene.

CTCF^HALO^ ES cells were seeded at a density of 35,000 cells cm^−2^, and after 24 h, cells were co-transfected with 3 μg of plasmid containing sgRNAs and 3 μg of the donor plasmid with the KI cassette using Lipofectamine Stem Transfection Reagent (Thermo Fisher, STEM00008) according to the manufacturer’s instructions. At 36 h, double-positive (RFP657-positive and GFP-positive) cells were purified using flow cytometry (FACSAria BDII). Subsequently, cells were plated at a density of 150 cells cm^−2^ on a 0.2% gelatin-coated Petri dish. Single colonies of cells were picked at day 5 onto 96-well plates. Mouse Direct PCR Kit (Bimake, B40015) and M-PCR OPTI Mix (Bimake, B45012) were used to screen for colonies for homozygous insertion of the KI cassette.

### Additional CRISPR–Cas9 genome editing in the CTCF^HALO^ and Sox1-GFP ES cells

We targeted Ddx5, Fus, Pantr1 and Neat1 loci in CTCF^HALO^ ES cells. The CTCF binding sites at the Aldh1a3 were targeted in SOX1-GFP-puro ES cells (the 46C line). For genome editing, ES cells were grown under standard conditions (see above).

We used an sgRNA design tool (http://crispor.tefor.net) to design the guides targeting the Ddx5 locus region on chr11: 106,779,390–106,789,735, Fus locus on region chr7:127,966,309–127,965,835, Pantr1 on the region chr1:42,694,916–42,692,353, Neat1 lncRNA (chr19: 5842235–5845557) and three Ctcf-binding sites in Aldh1a3 locus at following locations: KO#1 (chr7:66,389,290–66,389,666), KO#2 (chr7: 66,409,322–66,410,004) and KO#3 (chr7:66,434,748–66,435,124).

For each genomic target, two different sgRNAs were designed and synthesized as short oligos. Oligos were annealed and cloned into the BbsI site of the 2A-GFP (PX458) plasmid (Addgene, #48138) according to the Zhang Lab General Cloning Protocol (https://www.addgene.org/crispr/zhang/).

ES cells that were seeded on the previous day (37,000 cells cm^−2^ per well of a 6-well plate) were co-transfected with the two px458 plasmids containing the sgRNA (3 µg of each plasmid was used) using Lipofectamine Stem Transfection Reagent (Thermo Fisher, STEM00008) according to the manufacturer’s instructions. Twenty-four hours after transfection, the GFP-expressing cells were purified using flow cytometry (FACSAria BDII). Cells were seeded on a 0.2% gelatin-coated Petri dish (150 cells cm^−2^). After 5 days, single colonies were manually picked and transferred into 96-well plates (VWR International, 734-2317P). Colonies were genotyped using Mouse Direct PCR Kit (Bimake, B40015) and M-PCR OPTI Mix (Bimake, B45012).

The genotyping PCR reactions were carried out as follows:

DDX5: initial denaturation 95 °C, 3 min, followed by 35 cycles of denaturation 95 °C, 20 s; hybridization 61 °C, 20 s; extension 68 °C, 4 min. The final extrusion was performed at 68 °C for 5 min.

Fus: initial denaturation 95 °C, 3 min, followed by 35 cycles of denaturation 95 °C, 20 s; hybridization 57 °C, 20 s; extension 68 °C, 2.45 min. The final extrusion was performed at 68 °C for 5 min.

Pantr1 external PCR: initial denaturation 95 °C, 3 min, followed by 35 cycles of denaturation 95 °C, 30 s; hybridization 58 °C, 30 s; extension 68 °C, 1.40 min. The final extrusion was performed at 68 °C for 5 min.

Pantr1 internal PCR: initial denaturation 95 °C, 3 min, followed by 35 cycles of denaturation 95 °C, 20 s; hybridization 52 °C, 20 s; extension 72 °C, 25 s. The final extension was performed at 72 °C for 5 min.

Neat1 PCR: initial denaturation 95 °C, 3 min, followed by 35 cycles of denaturation 95 °C, 30 s; hybridization 56 °C, 30 s; extension 68 °C, 2.30 min. The final extrusion was performed at 68 °C for 5 min.

Ddx5 KI: initial denaturation 94 °C, 5 min, followed by 35 cycles of denaturation 94 °C, 20 s; hybridization 61.7 °C, 30 s; extension 72 °C, 1 min 45 s. The final extrusion was performed at 72 °C for 5 min.

#1 Ctcf binding site in Aldh1a3: initial denaturation 95 °C, 3 min, followed by 35 cycles of denaturation 95 °C, 20 s; hybridization 60 °C, 20 s; extension 72 °C, 30 s. The final extension was performed at 72 °C for 5 min.

#2 Ctcf binding site in Aldh1a3: initial denaturation 95 °C, 3 min, followed by 35 cycles of denaturation 95 °C, 20 s; hybridization 60 °C, 20 s; extension 72 °C, 30 s. The final extension was performed at 72 °C for 5 min.

#3 Ctcf binding site in Aldh1a3: initial denaturation 95 °C, 3 min, followed by 35 cycles of denaturation 95 °C, 20 s; hybridization 52 °C, 20 s; extension 72 °C, 30 s. The final extension was performed at 72 °C for 5 min.

For verification of proper genome editing, the following primers were applied:

Fus_sgRNA_1_FWDcaccGTTTGCCCACATTCGGGTACT

Fus_sgRNA_1_RVaaacAGTACCCGAATGTGGGCAAA

Fus_sgRNA_2_FWDcaccGGCCCGCCCACGGAACAGTG

Fus_sgRNA_2_RVaaacCACTGTTCCGTGGGCGGGCC

Fus_genotyping_FWDAGGCTTCCTACTTCAGCCTC

Fus_genotyping_RVCACCACCTCTGTGAATCACAG

Ddx5_sgRNA_1_FWDcaccGGCACCTCATTCATTTCCAT

Ddx5_sgRNA_1_RVaaacATGGAAATGAATGAGGTGCC

Ddx5_sgRNA_2_FWDcaccTGAAAACCACTCAGTACTAG

Ddx5_sgRNA_2_RVaaacCTAGTACTGAGTGGTTTTCA

Ddx5_genotyping_FWDGAGGAGGCGGTCCAGACTATAAAAG

Ddx5_genotyping _RVAGGGACAATCTCTGACTTCAAGG

Aldh1a3_KO_#1 _sgRNA1_FWD caccGAGTATTCAACTGTACCCAGT

Aldh1a3_KO_#1 _sgRNA1_RVaaacACTGGGTACAGTTGAATACTC

Aldh1a3_KO_#1_ sgRNA2_FWDcaccGGTCCTCAGACCAATTAGCA

Aldh1a3_KO_#1_ sgRNA2_RVaaacTGCTAATTGGTCTGAGGACC

Aldh1a3_KO_#1_ genotyping_FWDGTGCAAAGAACATTGACAGA

Aldh1a3_KO_#1_ genotyping_RVAACTGTGATTGTAGGTGGAG

Aldh1a3_KO_#2_sgRNA1_FWDcaccGCCTACTACAAACCTATCTGC

Aldh1a3_KO_#2_ sgRNA1_RVaaacGCAGATAGGTTTGTAGTAGGC

Aldh1a3_KO_#2_ sgRNA2_FWDcaccGTATTGGCTTAGCAAGGGCAT

Aldh1a3_KO_#2_ sgRNA2_RVaaacATGCCCTTGCTAAGCCAATAC

Aldh1a3_KO_#2_ genotyping_FWDTACCTCTGTGGAGCCGGTG

Aldh1a3_KO_#2 _genotyping_RVGAACCAGCTGTGGACCGG

Aldh1a3_KO_#3 _sgRNA1_FWDcaccGCCAAACTTCAGTGGTGCATA

Aldh1a3_KO_#3 _sgRNA1_RVaaacTATGCACCACTGAAGTTTGGC

Aldh1a3_KO_#3_ sgRNA2_FWDcaccGCACCACCGAGACTTCAGCTA

Aldh1a3_KO_#3_ sgRNA2_RVaaacTAGCTGAAGTCTCGGTGGTGC

Aldh1a3_KO_ #3_ genotyping_FWDAGCACTGGGCTTGCATC

Aldh1a3_KO_#3_ genotyping_RVGGTAGGCACTGAGGAAA

Pantr1_ genotyping_external_FWDACGCGAGAGATTTGTAAAG

Pantr1_ genotyping_external_RVTCATTACAAACCACTGCATT

Pantr1_ genotyping_internal_FWDATTTCTCTAGAGGGCTCAC

Pantr1_ genotyping_ internal _RVCGATTTGAGAACTAAGTACG

Pantr1_ KO_sgRNA1_FWDcaccgCCTAGTTAAAGCTGCAAGTG

Pantr1_ KO_sgRNA1_RVaaacCACTTGCAGCTTTAACTAGGC

Pantr1_ KO_sgRNA2_FWDcaccgGCGAGTCCGACCGCTTGCTG

Pantr1_ KO_sgRNA2_RVaaacCAGCAAGCGGTCGGACTCGCC

Neat1_KO_sgRNA1_FWDcaccgATCTAGGCCTAACTATATGA

Neat1_KO_sgRNA1_RVaaacTCATATAGTTAGGCCTAGATC

Neat1_KO_sgRNA2_FWDcaccGTAAACGGAACGATTCCTCCA

Neat1_KO_sgRNA2_RVaaacTGGAGGAATCGTTCCGTTTAC

Neat1_genotyping_FWDTGCCATTATCCCATGACTCAG

Neat1_genotyping_RVTTCATCCTGTGACGCACC

Ddx5_KI_genotyping FWDAATGCTGCAGTACAAAACCAC

Ddx5_KI_genotyping RVCAGGTTTGCCCTCACATTTC

Ddx5_KI_sgRNA1_FWDcaccgCTAGTGACCGAGACCGCGGC

Ddx5_KI_sgRNA1_RVaaacGCCGCGGTCTCGGTCACTAGC

Ddx5_KI_sgRNA2_FWDcaccgTATTCTAGTGACCGAGACCG

Ddx5_KI_sgRNA2_RVaaacCGGTCTCGGTCACTAGAATAC

### RNA extraction, reverse transcription and quantitative real-time PCR

Pellets of 250,000 cells were lysed in TRI reagent (Merck, T9424). RNA was isolated with a Direct-zol RNA MiniPrep kit (Zymo, R2050), according to the manufacturer’s instructions. RNA quality was examined with Nanodrop (Thermo Scientific). The High-Capacity cDNA Reverse Transcription Kit (Thermo Fisher, 4368814) was used to obtain complementary DNA from 600 ng of RNA in a 20-μl reaction volume. For all samples, negative controls without reverse transcriptase enzymes were also prepared.

The real-time quantitative PCR (qPCR) assays were carried out using CFX Opus Real-Time PCR Systems (Bio-Rad). The 10-μl reaction mix consisted of 4 μl Fast SYBR Green Master Mix (Thermo Fisher, 4385616), 0.5 μl primer solution (10 pmol μl^−1^) and 4.5 μl cDNA solution (diluted 1:80). PCR conditions were as follows: 95 °C for 3 min followed by 40 cycles of 95 °C for 10 s and 60 °C for 30 s.

### RNA-seq

RNA was isolated with the Direct-zol RNA MiniPrep Kit (Zymo Research, R2050), according to the manufacturer’s instructions. RNA quality was examined with the Agilent RNA 6000 Nano Kit (Agilent, 5067-1511). Samples featuring RNA integrity number >8 were considered for further analysis. RNA-seq libraries were prepared using the KAPA mRNA HyperPrep Kit (Roche, 8098115702), with 1 μg RNA as the starting material. Library preparation was performed according to the manufacturer’s instructions, using UMI in xGen UDI-UMI Adapters (IDT 10005903). The size of DNA fragments was examined with TapeStation DNA ScreenTape & Reagents (Agilent 5067-5585; 5067-5584) on TapeStation4200 Device (Agilent). Libraries were sequenced (2 × 100 bp, paired end) using NovaSeq6000 (Illumina).

### ATAC-seq

ATAC-seq libraries were prepared using an ATAC-seq kit (Active Motif, 53150), according to the manufacturer’s instructions, using 100,000 cells detached from the culture plastic. Libraries were sequenced (2 × 100 bp, paired end) using NovaSeq6000 (Illumina).

### ChIP-seq

ChIP-seq experiments were performed as described previously^9^. Chromatin corresponding to 3 million cells (H3K27ac) or 10 million cells (CTCF) was considered. The following antibodies were used in ChIP: anti-H3K27ac (Cell Signaling, 8173S; 1:100), and anti-CTCF (Sigma-Merck, 07-729, 5 μl per 10 million cells). Libraries were prepared using the Ovation Ultralow V2 DNA-Seq Library Preparation Kit (Tecan, 0344NB-32), according to the manufacturer’s recommendations. Libraries were sequenced (2 × 100 bp, paired end) using NovaSeq6000 (Illumina).

### In situ Hi-C

Pellets of 5 million formaldehyde cross-linked cells were used. In situ Hi-C was performed as described previously^4^, with modification at the library preparation step, which was done using the NEBNext Ultra II DNA Library Kit (NEB, E7103S), according to the manufacturer’s instructions. Libraries were sequenced (2 × 150 paired end) using a NovaSeq6000 (Illumina).

### ChIP–SICAP

ChIP–SICAP was carried out as described previously^127^. The cells were fixed by resuspending the cells in formaldehyde 1.5% (v/v) in PBS for 15 min, quenched by 125 mM glycine and stored at −80 °C. For each replicate, 12 million cells were sonicated using Bioruptor Pico. After immunoprecipitation with the CTCF antibody (10 μg per chromatin extract, anti-CTCF (D31H2) XP Rabbit mAb #3418, Cell Signaling Technology), chromatin fragments were captured on Protein-A beads, and DNA was biotinylated by TdT in the presence of biotin-11-ddUTP. The beads were washed six times using PBS–Triton X-100 1% (v/v), and the chromatin fragments were eluted by 7.5% (w/v) SDS and 200 mM dithiothreitol (DTT). The eluted protein–DNA complexes were captured again by protease-resistant streptavidin (prS) beads (PMID: 32400114). The beads were washed three times using 1% (v/v) PBS–SDS 1%, once with 2 M NaCl, twice with 20% (v/v) 2-propanol and five times with 40% (v/v) acetonitrile. Finally, the beads were transferred to PCR tubes and resuspended in 100 mM AMBIC buffer and 10 mM DTT. The beads were incubated at 50 °C for 15 min. Then, proteins were alkylated by 20 mM iodoacetimide for 30 min in the dark. Iodoacetimide was neutralized by adding 10 mM DTT. The proteins were digested on the beads by adding 300 ng LysC and incubating overnight at 37 °C. The supernatant was transferred to new PCR tubes and further digested by adding 100 ng Trypsin Gold for 6 h. The peptides were cleaned using stage tips and analysed on an Orbitrap Fusion mass spectrometrer operating in data-dependent acquisition mode.

### Fluorescence recovery after photobleaching

CTCF^HALO^ cells were seeded on laminin (Sigma-Merck, L2020-1MG)-coated four-chamber 35-mm glass Petri dishes (IBL BAUSTOFF, 220.120.022) at a density of 35,000 cells cm^−2^. Twenty-four hours later, cells were incubated with 5 µM TMR, a HALOTag ligand (Promega, G8252), at 37 ºC for 30 min. Cells were washed three times with fresh medium and incubated at 37 °C for 30 min in the cell culture medium, followed by an additional wash with fresh medium.

FRAP was performed using a Zeiss LSM780 confocal microscope with an incubation chamber maintaining 37 °C 5% CO_2_ and a heated stage. Images were acquired on a 40× water-immersion objective at a zoom corresponding to a 100 nm × 100 nm pixel size with 300 frames acquired at one frame per second (five frames were acquired before the bleach). A circular bleach spot (radius (*r*) = 10 pixels)) was chosen in a region of homogeneous fluorescence at a position at least 1 mm from nuclear or nucleolar boundaries. The spot was bleached using maximal laser intensity for a total of 30 iterations. Three regions of interest were measured for each nucleus: ROI 1, bleached region; ROI 2, nucleus; and ROI 3, background. Data from at least 15–20 cells per condition and per experiment were collected. Regions of interest were chosen manually in ImageJ. The StackregJ plugin for ImageJ was used to correct for nucleus movement. Recovery curve data normalization was performed as in ref. ^65^.

### Immunofluorescence

Cells on coverslips were fixed using 4% paraformaldehyde (PFA; Merck, 158127) in DPBS for 15 min at RT. The coverslips were washed three times with DPBS (Gibco, 21600-069) for 5 min and permeabilized with 0.5% Triton X-100 (Bio-Rad, 1610407) for 15 min at RT. Samples were then incubated in blocking solution (0.5% BSA (BioShop, ALB001) in DPBS) for 1 h. Coverslips were incubated with primary antibodies Oct4 (1:400, Santa Cruz, sc-5279), Nestin (1:100, Developmental Studies Hybridoma Bank, rat-401), GFAP (1:200, Proteintech, 16825-1-AP) and Tubb3 (1:300, Proteintech, 66375-1-Ig) in blocking solution for 1 h at RT. Cells were washed three times with DPBS for 5 min at RT. Cells were subsequently incubated with Alexa Fluor 488/568-conjugated secondary antibody (1:1,000, Thermo Fisher, A-11001) and Hoechst 33342 (1:2,000, Thermo Fisher, 34580) in blocking solution for 1 h at RT. Cells were washed three times with DPBS as described above. Coverslips were then mounted to slides using prolong diamond antifade mounting medium (Thermo Fisher, P36961). Images were acquired in consecutive planes (*z*) at a focal distance of 0.18 µm with Zeiss LSM800 Inverted Axio Observer Z.1, using Plan Apochromat 63×/1.4 oil DIC objectives and diode lasers 405, 488 and 561 nm, in AiryScan mode. The raw images were processed using AiryScan in Zen2.6 software with default parameters.

### Visualization of CTCF in live and paraformaldehyde-fixed cells

CTCF^HALO^ cells were seeded at a density of 35,000 cells cm^−2^. After 24 h, the cells were incubated with 5 µM TMR ligand (Promega, G8252) in culture medium for 30 min at 37 °C in a 5% (v/v) CO_2_ incubator. Cells were washed with PBS twice for a short time (5 min incubation) and once for 30 min at 37 ºC.

To assess CTCF clusters upon acute depletion of Ddx5, CTCF^HALO^Ddx5^FKBP^ ES and NS cells were seeded at a density of 35,000 cells cm^−2^. After 24 h, the cells were treated with either DMSO or 500 nM of dTAG13 dissolved in DMSO (DMSO final concentration 0.01%) and incubated for 24 h at 37 °C in a 5% (v/v) CO_2_ incubator. After treatment, the cells were incubated with 5 µM TMR ligand (Promega, G8252) in culture medium with and without dTAG13 for 30 min, washed twice with PBS and incubated for an additional 30 min in PBS with and without dTAG13.

For live-cell imaging, the TMR-stained cells were incubated with fresh medium. Live-cell imaging was performed in AiryScan mode in Zeiss Cell Discoverer 7 with LSM900, Inverted Axio Observer Z.1 using Plan Apochromat 50×/1.2 Water Autocorr objectives and diode laser 561 nm with an incubation chamber maintaining 37 °C and 5% CO_2_ and a heated stage. Images were acquired with *z* stacks at a focal distance of 0.18 µm at 16-bit depth. The raw images were processed using AiryScan in Zen2.6 software with default parameters.

For STED imaging, the TMR-stained cells were fixed using 4% PFA in DPBS for 15 min at RT. The cells were next washed with DPBS three times for 5 min. Cells were mounted to slides using glycerol with DABCO solution (Sigma-Aldrich D27802, 25 mg ml^−1^ in a 90% glycerol–PBS mix). Images were acquired at a focal distance of 0.23 µm at 16-bit depth on Stellaris 8 STED Falcon, using Tau-STED 2D/3D + Depletion Lasers 775 nM with HC PL APO CS2 93×/1.30 GLYC objective. Laserlines 660 and 775 nm were used.

For CTCF imaging after preextraction, the fraction of CTCF unbound to DNA was removed by incubating the TMR-stained cells with freshly made preextraction buffer (10 mM pH 6.8 KOH, 100 mM NaCl, 300 mM sucrose, 1 mM EGTA, 1 mM MgCl_2_, 1 mM DTT + 0.5% Triton X-100 + 1× protease inhibitor) for 5 min on ice. Preextracted cells were then fixed using 4% PFA in DPBS for 15 min at RT. The cells were washed three times with DPBS for 5 min. The coverslip was mounted onto a microscopy slide using Prolong Diamond Antifade solution (Thermo Fisher, P36961). Images were acquired using (1) Zeiss LSM800 Inverted Axio Observer Z.1, using Plan Apochromat 63×/1.4 oil DIC objectives and diode lasers 561 nm, in AiryScan mode, and (2) Stellaris 8 STED Falcon, using Tau-STED 2D/3D + Depletion Lasers 775 nM with HC PL APO CS2 93×/1.30 GLYC objective. Laserlines 660 and 775 nm were used. For LSM800, images were acquired with *z* stacks at a focal distance of 0.13 µm at 16-bit depth, while for STED, images were acquired with *z* stacks at a focal distance of 0.18 µm at 16-bit depth. The raw images were processed using AiryScan in Zen2.6 software with default parameters.

For flow cytometry analysis, the TMR-stained cells were detached from the culture plastic using Accutase and fixed using 4% PFA in DPBS for 15 min at RT. A BD FACSCalibur flow cytometer was used to assess the per-cell fluorescence intensity. Data were analysed using FlowJo software (version 10.8.1).

### RNaseA treatment

CTCF^HALO^ cells were grown on coverslips. On the day of the experiment, the coverslips were incubated with permeabilization buffer (0.25% Tween-20 (Sigma- Merck, P1379-100ML), 0,005% digitonin (Sigma- Merck, 300410-250MG) and DPBS with Ca^2+^ and Mg^2+^ (Biowest, X0520-500)) with or without 500 μg ml^−1^ RNaseA (Thermo Fisher, EN0531) for 30 min at 37 °C. The coverslips were washed with DPBS once. The cells were fixed with 4% PFA in DPBS for 15 min at RT. The coverslips containing the fixed cells were washed three times with DPBS for 5 min and incubated with Hoechst 33342 (1:2,000, Invitrogen, H3570) for 5 min at RT. To assess the total RNA content, the coverslips were treated with 100 μM of Pyronin Y (Sigma-Merck, 83200-5G) in DPBS for 2 min. The coverslips were washed three times with DPBS for 5 min and mounted on microscope slides with Prolong Diamond Antifade solution (Thermo Fisher, P36961).

Confocal images were acquired on Zeiss LSM800 Inverted Axio Observer Z.1, using Plan Apochromat 63×/1.4 oil DIC objectives and diode lasers 405 and 488 nm in individual planes at a focal distance of 0.56 µm at 8-bit depth.

### Proximity ligation assay

The assay was performed using Duolink PLA Fluorescence Protocol (Sigma-Merck, DUO92101) using CTCF^HALO^ cells. All the steps were performed according to the manufacturer’s protocol. In brief, cells were grown on coverslips. On the day of the experiment, the cells on the coverslips were fixed using 4% PFA (Merck-Sigma, 252549) in DPBS for 15 min at RT. The coverslips were washed three times with DPBS for 5 min at RT followed by permeabilization with 0.5% of Triton X-100 in DPBS for 15 min at RT. The coverslips were washed three times with DPBS for 5 min and incubated with Duolink PLA blocking solution for 1 h at 37 °C. Samples were then incubated with primary antibodies: CTCF (1:50, Santa Cruz sc-271474), Ddx5 (1:50, 26385-1-AP), Fus (1:50, 11570-1-AP) and Nono (1:50, 11058-1-AP) for 1 h at RT in Duolink Antibody Diluent and then washed with Duolink wash buffer A (WB-A) (2×, 5 min). Subsequently, coverslips were incubated with the PLA Probe diluted in Duolink Antibody Diluent for 1 h at 37 °C and washed twice with WB-A for 5 min. For probe ligation, Duolink 1× ligase was added and incubated at 37 °C for 30 min. Samples were washed twice with WB-A for 5 min. Coverslips were next incubated with Duolink polymerase for 100 min at 37 °C. Coverslips were washed twice with Duolink 1× wash buffer B for 10 min and mounted with Duolink In Situ Mounting Medium with DAPI. Images were acquired with *z* stacks at a focal distance of 0.13 µm on Zeiss LSM800 Inverted Axio Observer Z.1, using Plan Apochromat 63×/1.4 oil DIC objectives and diode lasers 405 and 561 nm, in AiryScan mode. The raw images were processed using AiryScan in Zen2.6 software with default parameters.

PLA particle analysis was done using Fiji software version 2.1.0/1.53c. In brief, background removal preprocessing for the PLA was performed as described^128^. Then, PLA probe particles of the size range 0–10 μm^2^ were analysed with the Analyze particles plugin, and interactions (particles) were counted manually for a single nucleus.

### Computational analyses

#### RNA-seq data preprocessing

Raw RNA-seq reads were trimmed using TrimGalore version 0.6.7, using parameters ‘--paired -q 30--stringency 3--length 30’. Reads were aligned to the *Mus musculus* (mm10/GRCm38) genome using STAR version 2.7.10 with default parameters and ‘outFilterMultimapNmax 1’. We used featureCounts version 2.0.3 with parameters ‘-p -O--countReadPairs -t exon -g gene_id’ to obtain per-gene RNA-seq read counts using ‘Mus_musculus.GRCm38.101.gtf’ from Ensembl’s release version 101 as a reference. Transcript-per-million-normalized files were obtained using bamCoverage tool from deeptools v3.5.

#### Preprocessing of and peak calling in the ATAC-seq and ChIP-seq data

Raw reads were trimmed using TrimGalore version 0.6.7, using parameters ‘--paired -q 30--stringency 3--length 30’, and alignment was performed using bowtie2 using parameters ‘--very- sensitive -X 2000’. All the ATAC-seq, H3K27ac ChIP-seq and CTCF ChIP-seq data were aligned to the *Mus musculus* (mm10/GRCm38) genome. The alignments were filtered to remove duplicates using alignmentSieve (using parameters ‘--minFragmentLength 40 --ignoreDuplicates’), which is available as a part of the deeptools package version 3.5. Reads mapping to black-listed regions (https://github.com/Boyle-Lab/Blacklist/blob/master/lists/mm10-blacklist.v2.bed.gz) were removed using samtools version 1.13. Next, bamCoverage was used to generate Reads per genomic content (RPGC)-normalized bigwig files.

Peak calling was performed using MACS2 (Model-based Analysis for ChIP-Seq) version 2.2.7.1 using parameters ‘--no-model’. The effective genome size required as one of the input parameters for the program was kept at default for mice. RPGC-normalized files were obtained using bamCoverage tool from deeptools v3.5.

#### Hi-C data preprocessing

Raw Hi-C reads were trimmed using TrimGalore version 0.6.7. The fastq files were processed using the Juicer Pipeline^129^ version 2.13.07, using default options and *Mus musculus* (mm10/GRCm38) genome assembly. Restriction digestion sites for MboI in the mouse genome were available from the Juicer package.

#### Topological data analysis

Robust tools from persistent homology (PH) have been used to analyse the distribution of CTCF in the nuclei of ES and NS cell types. The process initiates with a 3D stack of greyscale images. Individual nuclei are segmented independently for each slice using the watershed algorithm [watershed], guided by manually selected markers. After a manual quality check applied to all segmented images, 5 out of 96 images were excluded due to an absence of clear segmentation between nuclei. The remaining images were standardized, mapping the voxel values to the [0,1] interval, with the minimum greyscale value being mapped to 0 and the maximum to 1.

PH analysis was conducted on the masked and standardized images. The concept of PH is illustrated in Extended Data Fig. 1e. In brief, in PH analysis, voxels are added to the image in descending order with respect to their grayscale levels. At each iteration, the algorithm records the topological features in different dimensions. Specifically, for dimension 0, it tracks the creation (birth) and merging (death) of connected components. Analogous birth–death events are recorded for topological features of dimensions 1 and 2. A feature of dimension 1 represents a cycle or loop, created when it closes and terminated when it becomes filled in. Two-dimensional features denote voids entirely enclosed by voxels, which cease to exist when filled from within. Each such feature is characterized by the greyscale levels at its birth and death, stored as a pair of numbers called a birth–death pair. A collection of birth–death pairs from all zero-, one- and two-dimensional features allows us to build a persistence diagram in the corresponding dimension. These three persistence diagrams are used as feature representations of the input image stack and are mapped to corresponding vectors using three primary vectorization techniques: persistence images^130^, Betti curves^131^ and persistence statistics^132^.

The vectorized diagrams serve as input to random forest and support vector machine classifiers to distinguish between ES and NS nuclei. Classification involved a 70/30 training/test set and 5-fold cross-validation and was carried out using the Python library scikit-learn. The average classification performance on the test set was approximately 90% (100% for the training set).

In addition to supervised classification, unsupervised approaches using clustering techniques were applied to the three vectorized persistent diagrams. When using *k*-means clustering with *k* = 3, an agreement of around 90% was observed between the labels assigned by *k*-means and the biological labels. Notably, the NS cells form one cluster, while the ES cells divide into two clusters. The label-guided projection of the obtained clusters can be found in Extended Data Fig. 1e.

#### CTCF cluster analysis

For cluster analysis using AiryScan, 3D images were acquired for CTCF-TMR-stained cells. The raw images were processed using AiryScan in Zen2.6 software with default parameters. Image analysis was performed using FIJI software version 2.1.0/1.53c. In all the images, the signal intensity threshold was kept constant, and the volume and number of clusters were measured using the 3D Objects Counter v2.0 plugin. The visual representation of cluster assemblies was analysed with the Volume Viewer plugin with similar axial positions in Volume and Slice & Border mode in all the images.

For cluster analysis using STED, 3D images for CTCF-TMR-stained cells were acquired, and the clusters were determined using the central plane of each image. Image analysis was performed using FIJI software version 2.1.0/1.53c. The raw images were preprocessed as follows: images were Gaussian blur (Sigma: 1.5), followed by Background subtraction (rolling ball radius:10 pixels and sliding paraboloid). The images were then converted to Binary images and Watershed. The clusters were then analysed using the Analyze Particle parameter.

#### Nuclear size analysis

Three-dimensional images of the DAPI-stained nuclei were acquired using Zeiss LSM800 Inverted Axio Observer Z.1 with Plan Apochromat 63×/1.4 oil DIC objectives and diode lasers 405 nm, in AiryScan mode. Images were acquired with *z* stacks at a focal distance of 0.13 µm at 16-bit depth. The raw images were processed using AiryScan in Zen2.6 software with default parameters. The nucleus volume was determined using the 3D Object counter v2.0 plugin in Fijji 2.16.0/1.54p.

#### ChIP–SICAP analysis

RAW files were analysed using Proteome Discoverer (2.1). Tandem mass spectra were searched against the UniProt (Swissprot) database (*Mus musculus*) using the Sequest HT node. Trypsin/P and LysC were chosen as enzyme specificity, allowing a maximum of two missed cleavages. Cysteine carbamidomethylation was chosen as the fixed modification, and methionine oxidation and protein N-terminal acetylation were used as variable modifications. Likewise, in the Precursor Ions Quantifiers node, Normalization and Scaling, normalization mode, ‘Specific Protein Amount’ was chosen to calculate the normalization factor from the abundances of CTCF protein from the FASTA file. The false discovery rate (FDR) for both proteins and peptides was set to 1% using the Percolator node.

Statistical analysis was performed using RStudio. The limma package was used to determine Bayesian-moderated *t*-test *P* values and Benjamini–Hochberg-adjusted *P* values (*P*values or FDRs). We, therefore, considered *P*-adj. < 0.1 as significantly enriched proteins.

#### Identification of cell-type-specific loops from Hi-C data

Loop calling was done using HiCCUPS using the default parameters as a part of Juicer 2.13.07. Loops called by HiCCUPS in the NS cells were considered.

#### CTCF motif directionality

We considered DNA sequences of the mouse genome (mm10). We used the CTCF motifs from the HOCOMOCO v11 database^133^. We used FIMO^134^ to scan the whole mouse genome for the CTCF motif, using parameter ‘--text’. Peaks of CTCF binding were identified as indicated above. The 50-bp regions centred at the peak summits were considered, and CTCF motifs found by FIMO were extracted. The motif with the highest score was identified and considered in the following analyses.

#### Comparisons of ChIP-seq signal between conditions

In the analysis examining the impact of IAA treatment on CTCF signal in ES and NS cells, CTCF peak locations identified from ChIP-seq libraries of untreated cells were used. The RPGC-normalized signal at the peak summit was extracted from the ChIP-seq bigwig files using a custom script in R. The values obtained from the untreated or IAA-treated conditions were compared with each other.

In the analysis comparing the CTCF signal in the wild-type and Ddx5^−/−^ ES and NS cells, CTCF peak locations obtained in the wild-type cells were considered. The average CTCF signal in the 100-bp region centred on the peak summit was obtained from the RPGC or raw read files in the wild-type and Ddx5^−/−^ cells.

DESeq2 version 1.32.0 was considered to quantitatively compare the area under the curve (AUC) signal of CTCF in wild type and Ddx5^−/−^ NS cells, The AUC was retrieved from the raw bigwig files in each sample. DEseq2 was applied with parameter fit set to ‘local’. The analysis provided a list of peaks with altered CTCF signal at an FDR of 25%. The analysis of RPGC-normalized signals at these locations confirmed the robustness of this approach.

Peaks altered upon dTAG13 treatment were identified using two biological replicates of DMSO-dTAG13-treated sample pairs. Peaks featuring change in CTCF abundance were instances in which the CTCF signal was congruently altered by at least 25% upon treatment in both replicates.

In the analysis of the CTCF signal in Pantr1-knockout NS cells, we considered CTCF peak locations identified in the wild-type samples. We then identified peaks that changed AUC (RPGC-normalized signal) in both Pantr1^−/−^ clones by at least 25% in the same direction.

#### Analysis of CTCF peaks featuring changes in CTCF binding upon Ddx5 loss

Peaks for which we scored a congruent change in CTCF signal in both Ddx5^−/−^ NS cells and upon acute depletion of Ddx5 protein were considered in the analysis. The 500-bp DNA sequence centred at the CTCF peak summit was retrieved using the getSeq function from the R/Bioconductor package Biostrings, taking advantage of the BSgenome.Mmusculus.UCSC.mm10 object from the R/Bioconductor package BSgenome.Mmusculus.UCSC.mm10. The motifs from the Hocomoco database were obtained (data object ‘hocomoco’ from the R/Bioconductor package motifbreakR).

The occurrence of the hocomoco TF motifs was then assessed in the CTCF peak sequences using countPWM from the R/Bioconductor package Biostrings. A minimal score of 80% was required to call a hit (min.score parameter in the countPWM function).

Then, for each TF, the fraction of sequences containing the motif was computed and compared for peaks featuring diminished or enhanced CTCF signal upon Ddx5 loss. Colour indicated a significant skew in the proportion of the peaks in the comparison (fold change (FC) >1.25, corrected *P* for Fisher’s exact test <0.1; we used the fdrtool function from the fdrtools R/Bioconductor package to estimate the *q* value).

Peaks were annotated to genomic features using the annotatePeak function from the R/Bioconductor package ChIPseeker, with TxDb.Mmusculus.UCSC.mm10.knownGene as the reference and the tssRegion parameter set to c(−3,000, 3,000), meaning that regions ±3 kb around the transcription start site were considered as promoters.

The CG nucleotides were counted using R/Bioconductor package Biostrings function vcountPattern.

#### G4q analysis

G4q were analysed using R/Bioconductor package pqsfinder. For the genome-wide prediction of G4q at CTCF peaks at loop anchors, G4q were assessed in the 2-kb window centred at the 5′ end of the CTCF motif. The max_defects parameter was set to 0, and the minimal_score parameter was fixed to 10.

When comparing peaks which featured changes in CTCF abundance upon Ddx5 loss, G4q were assessed in the 500-bp window centred at the CTCF peak summit; the max_defects was set to 0, and the minimal score was fixed to 20.

#### Identification of enhancers and promoters

ATAC- and ChIP-seq data obtained using 46C ES (2i/LIF) and NS cells were considered. A database of gene models (gtf file Mus_musculus.GRCm38.101.gtf) was then used to extract promoter locations (±500 bp around the annotated transcription start sites).

Enhancers were defined as ATAC-seq peaks overlapping with regions enriched in H3K27ac and found outside promoters defined above.

#### In situ Hi-C in wild-type and Ddx5^−/−^ cells

The .hic files were obtained as described above. Next, the ligation frequency matrices (LFM, resolution of 10,000 bp) for the wild-type and Ddx5^−/−^ cells (two clones: CB1 and CE10; the LFMs were summed up) were obtained using the function dump from juicer. The matrices were normalized using iterative proportional fit (IPF) as described previously^9^.

#### APA analysis of loops

Aggregate peak analysis (APA) was performed using the IPF-normalized files and loop coordinates obtained in the wild-type NS cells. Loops spanning more than 100 kb of genomic distance were considered in the APA plot.

#### Loop scores and the identification of architectural loops

Juicer may call loops in areas where the local background is high. We thus needed to filter out the instances in which the loop signal (5 × 5 pixel square centred in the loop centroid) was low relative to the overall signal in the donut area surrounding the loop centroid (donut was defined as the 19 × 19 pixel square with the central 9 × 9 pixel area around the loop centroid removed).

To identify the highest-confidence loops for further analysis, we introduced the loop score (LS), which is directly inspired by the APA score, and we applied it to each loop individually. We considered the average of the IPF-normalized signal in the 5 × 5 pixel square around the loop centroid pixel (*x*_*ij*_) as the loop signal. The local background was defined by computing the average signal of 15 randomly drawn 5 × 5 pixel squares in the donut surrounding the 5 × 5 pixel central square at a distance of 8 pixels between from the loop centroid. The LS was defined as the logarithm base 2 of loop signal to the local background signal.

Architectural loops were loops with LS >1. We further identified instances in which the 5′ loop anchor overlapped wth at least one CTCF peak with a motif in a forward orientation while the 3′ loop anchor overlapped with at least one CTCF peak with a CTCF motif in a reverse orientation.

#### Identification of condition-specific loops

The effects of perturbations were assessed at the level of normalized Hi-C interaction signals (IPF values) and the level of loop signals. For both computations, loop centroids were considered. For each loop, the normalized HiC signal in a square of 5 × 5 pixels centred at the loop centroid bin was summed up and considered as the loop strength. First, we identified loops where, in all the wild-type versus mutant combinations, the loop signal was consistently higher in one condition versus the other. Second, to account for the local background, we further retained calls whereby the LS was consistently higher in all intercondition combinations of wild-type and mutant samples. This gave us the final lists of loops, either weaker or stronger in knockout compared with wild-type NS cells.

#### Analysis of compartments

Knight–Ruiz-normalized Hi-C matrices were taken directly from the .hic file generated by Juicer. To define A/B compartments, an eigenvector decomposition was performed on the normalized Hi-C contact matrices. Pearson correlation was then assessed, and principal component analysis was applied to compute the first three eigenvectors. The eigenvector with the highest absolute correlation with a phasing track (for example, GC% or gene density, automatically computed from the reference genome) was selected. The normalized Hi-C interaction matrix was sorted on the basis of the selected eigenvector values, ranging from the lowest (B compartment) to the highest (A compartment). The sorted maps were subsequently normalized to the expected interaction frequencies. In the resulting interaction matrix, the upper left corner represents the strongest B–B interactions, the lower right corner represents the strongest A–A interactions, and the upper right and lower left corners represent B–A and A–B interactions, respectively^16^ (https://bioconductor.org/books/release/OHCA/pages/workflow-chicken.html).

#### Analysis of Hi-C signal in the function of genomic distance

IPF-normalized Hi-C signal was retrieved at each off diagonal in the Hi-C matrix at a resolution of 10 kb. Next, the median for each off diagonal was displayed. Chromosome 14 was removed from the analysis as it featured library-specific effects in this plot, unrelated to genotype.

#### Analysis of insulation

The insulation score (IS) was computed as described previously^9^ with minor modifications. In brief, we isolated insulators (peaks of IS) and then compared the IS in the wild-type and Ddx5^−/−^ cells. The IS was estimated genome-wide at a resolution of 10 kb, considering three squares of 3 × 3 pixels, 10 bins from the diagonal (also displayed in the script). In brief, we considered the IPF-normalized interaction matrices of the wild-type and Ddx5^−/−^ cells (CB1 and CE10 LFMs were summed up before the IPF normalization). Peaks of insulation were defined as at least three consecutive bins featuring IS >0.75 (*N* = 6,823). For each bin within the insulation peak, the change of IS values was computed (IS_wild-type_ − IS_Ddx5−/−_). Finally, the average IS difference was considered for each peak.

#### Differential gene expression

DESeq2^135^ version 1.32.0 with default parameters was used to identify DEGs. We considered the comparisons between the IAA-treated and untreated ES (2i/LIF) and NS cells separately.

#### Analysis of the expression change of ncRNAs that interact with CTCF

The full list of ncRNAs interacting with CTCF was considered on the basis of the annotations published by Saldana-Meyer et al. (https://genesdev.cshlp.org/content/28/7/723/suppl/DC1).

We retrieved the annotation of mouse ncRNAs for the ensemble identifiers provided in the table. The list was next manually curated to identify possible missing lncRNAs. We specifically focused on the lncRNAs as defined in the table. We identified 58 lncRNAs that had also been annotated in the mouse genome.

We ran DESeq2 on the full transcriptome from the processed RNA-seq data from 46C ES and NS cells (polyA+ RNA species), using default parameters and including two biological replicates for each cell type. Then, once DEGs were identified, we retrieved the log_2_(FC) for the RNAs that featured differential abundance (*P*-adj. < 0.1).

#### Analysis of loop domains with respect to genes differentially expressed upon CTCF removal

We considered the promoter list from the analysis above, and for each gene, we identified its 5′-most promoter. For each of the three sets of genes (up, down and random), we identified the smallest loop domain (shortest as measured as the number of base pairs between the midpoints of the loop anchors) that contained its promoter. Then, we defined the left (−500 kb, beginning of the loop domain) and the right (+500 kb, end of the loop domain) flanks and counted the enhancers in these two flanks (46C NS cell ATAC-seq and H3K27ac ChIP-seq data were considered). The box plot in Fig. 4d displays the summed-up number of enhancers in the two flanks.

### Statistics and reproducibility

To mitigate the unspecific effects that might have arisen as a consequence of clonal expansion of cells, we obtained wild-type and mutant clones in parallel. Wild-type cells were single-cell clones of ES cells transfected with Cas9-expression vectors without sgRNAs; the mutant clones were obtained by transfecting the ES cells with vectors allowing the expression of Cas9 and the relevant sgRNAs. Both wild-type and mutant clones underwent flow cytometry-based isolation and clonal expansion. All the cell lines were genotyped routinely. All the NS cell lines were obtained similarly, by purifying the CD44-expressing cells. Analyses of CTCF clusters were performed three times. Each time, ES and NS cell preparations were separated into two parts: one was analysed by near super-resolution microscopy at the Nencki Institute; the second portion of ES and NS preparations was analysed by STED microscopy at EMBL in Heidelberg. Data analysis was performed separately, reaching the same conclusion. ChIP–SICAP, ChIP-seq and RNA-seq were performed on two biological replicate ES and NS pairs (or on DMSO-dTAG13-treated NS cells) in parallel in one experiment to reduce batch effects. In situ Hi-C comparing wild-type and Ddx5^−/−^ NS cells was performed on two clones of CTCF^HALO^ NS (wild-type) and two clones of Ddx5^−/−^ NS cells in one experiment to reduce batch effects. Hi-C in DMSO- or dTAG13-treated CTCF^HALO^Ddx5^FKBP^ NS cells was performed once and repeated subsequently, reaching the same conclusion. In Fig. 6, Hi-C was performed in one wild-type and two Pantr1^−/−^ NS cell clones. The IAA treatment was performed in four (ES) and three (NS) biological replicates in two distinct library preparation experiments for ES and NS cells. Validation of the effect of CTCF site removal on the expression of Aldh1a3 was performed in two distinct differentiation experiments. The 3D-FISH experiments were performed twice: in one experiment, ES and NS cells were profiled in parallel, and in the second experiment, only NS cells were considered. The experiments were not randomized. The investigators were not blinded to group allocation during experiments and outcome assessments, except for the CTCF cluster analysis, where colleagues at EMBL were blinded to the cell genotypes. Statistics were performed using R (version 4.2.1; RRID: SCR_001905). Normality and equal variances were tested where necessary.

### Reporting summary

Further information on research design is available in the Nature Portfolio Reporting Summary linked to this article.

## Online content

Any methods, additional references, Nature Portfolio reporting summaries, source data, extended data, supplementary information, acknowledgements, peer review information; details of author contributions and competing interests; and statements of data and code availability are available at 10.1038/s41556-025-01735-5.

## Supplementary information

Reporting Summary

Supplementary Code 1 Vignette and html with the R code.

Supplementary Tables 1–3 ChIP–SICAP, DEGs and 3D RNA-FISH primers.

## Source data

Source Data Figs. 1–8, and Extended Data Figs. 1–4 and 6–10 Statistical source data.

Source Data Figs. 1, 2, 5 and 7, and Extended Data Figs. 1, 3, 4, 6 and 7 Unprocessed gels or blots.

## Data Availability

Raw sequencing data are available in the ArrayExpress database (http://www.ebi.ac.uk/arrayexpress) under the following accession numbers: RNA-seq: E-MTAB-13558; ATAC-seq: E-MTAB-13559; CTCF ChIP-seq: E-MTAB-13562; H3K27Ac ChIP-seq: E-MTAB-13560; Hi-C: E-MTAB-13572. ChIP–SICAP data are available via ProteomeXchange with identifier PXD048470. Source data are provided with this paper.

## Code availability

Code is available in the Supplementary Information and at https://ctcfdevloops.nencki.edu.pl/.
