# Supplementary material for: RNA-binding proteins mediate the maturation of chromatin topology during differentiation
Source: Nat Cell Biol. 2025 Sep 8;27(9):1510–25. doi: 10.1038/s41556-025-01735-5 (PMC12431861; doi:10.1038/s41556-025-01735-5)
Supplement: Supplementary file 1 — Reporting Summary [file 41556_2025_1735_MOESM1_ESM.pdf]

nature portfolio

Corresponding author(s): Aleksandra Pękowska

Last updated by author(s): 2025/20/05

Reporting Summary

Nature Portfolio wishes to improve the reproducibility of the work that we publish. This form provides structure for consistency and transparency in reporting. For further information on Nature Portfolio policies, see our Editorial Policies and the Editorial Policy Checklist.

Statistics

For all statistical analyses, confirm that the following items are present in the figure legend, table legend, main text, or Methods section.

n/a	Confirmed	
☐	☒	The exact sample size (*n*) for each experimental group/condition, given as a discrete number and unit of measurement
☐	☒	A statement on whether measurements were taken from distinct samples or whether the same sample was measured repeatedly
☐	☒	The statistical test(s) used AND whether they are one- or two-sided<br>*Only common tests should be described solely by name; describe more complex techniques in the Methods section.*
☐	☒	A description of all covariates tested
☐	☒	A description of any assumptions or corrections, such as tests of normality and adjustment for multiple comparisons
☐	☒	A full description of the statistical parameters including central tendency (e.g. means) or other basic estimates (e.g. regression coefficient) AND variation (e.g. standard deviation) or associated estimates of uncertainty (e.g. confidence intervals)
☐	☒	For null hypothesis testing, the test statistic (e.g. *F*, *t*, *r*) with confidence intervals, effect sizes, degrees of freedom and *P* value noted<br>*Give P values as exact values whenever suitable.*
☒	☐	For Bayesian analysis, information on the choice of priors and Markov chain Monte Carlo settings
☒	☐	For hierarchical and complex designs, identification of the appropriate level for tests and full reporting of outcomes
☒	☐	Estimates of effect sizes (e.g. Cohen's *d*, Pearson's *r*), indicating how they were calculated

Our web collection on statistics for biologists contains articles on many of the points above.

Software and code

Policy information about availability of computer code

Data collection	Gene accession omnibus (GEO); Arrayexpress; mouse genome (GRCm38/mm10), Zeiss LSM800 Inverted Axio Observer Z.1 with Plan Apochromat 63x/1.4 Oil DIC objectives and Diode lasers 405, 488, 561 and 670 nm, Stellaris 8 STED Falcon, using Tau-STED 2D/3D + Depletion Lasers 775 nM with HC PL APO CS2 93x/1.30 GLYC objective.Zeiss LSM780 Inverted Axio Observer Z.1 with Plan Apochromat 40x/1.4 Oil DIC objectives and Diode lasers 561 nm, BD FACSAria II cell sorter, BD FACSCalibur, NovaSeq 6000
Data analysis	We included an analysis vignette which shows how the analysis was peformed. We included all the necessay information about the software in the Methods section. Additionally, we included the information on the github repository and will provide all the intermediate files on our webpage.<br>Fiji ver. 2.1.0/1.53c http://imagej.net<br>Image Studio™ Software version 6.0<br>ZEN ZEISS https://zeiss.com/ver 2.6<br>Microsoft Office Excel https://microsoft.com version 16.78.3<br>Bioconductor https:// bioconductor.org pacakage Biostrings<br>TrimGalore ver. 0.6.7 https://github.com/FelixKrueger/TrimGalore<br>STAR version 2.7.10<br>bowtie2 https://github.com/BenLangmead/bowtie2 version 2.5.1<br>MACS2 https://github.com/macs3-project/MACS macs2 2.2.7.1<br>SAMTOOLS https://github.com/Boyle-Lab/Blacklist/blob/master/lists/mm10-blacklist.v2.bed.gz version 1.13<br>HOCOMOCO v11 full database https://github.com/GabrielHoffman/tfbsDB<br>FIMO https://meme-suite.org/meme/doc/fimo.html<br>DAVID https://david.ncifcrf.gov version v2024q4

NCBI BLAST https://blast.ncbi.nlm.nih.gov/
rMATS turbo https://github.com/Xinglab/rmats-turbo rMATS_turbo_v4_1_2
Juicer https://github.com/theaidenlab/juicer/wiki vesrion 2.13.07
Integrated Genome Browser https://bioviz.org version 10.1.0
UCSC Genome Browser ttps://genome.ucsc.edu
CRISPOR http://crispor.org
fasterq-dump tool ver 3.0.7
BD FACSDiva version 8.0.1
FlowJo version 10.8.1
Script https://ctcfdevloops.nencki.edu.pl/
TDA https://github.com/janfsenge/TDA-Chromatin-Analysis
tibble_3.2.1
tidyverse_2.0.0
dplyr_1.1.4
compEpiTools_1.26.0
topGO_2.44.0
SparseM_1.81
GO.db_3.13.0
graph_1.70.0
TxDb.Mmusculus.UCSC.mm10.knownGene_3.10.0
GenomicFeatures_1.44.2
pheatmap_1.0.12
RColorBrewer_1.1-3
ggplot2_3.5.1
goseq_1.44.0
geneLenDataBase_1.28.0
BiasedUrn_2.0.12
fgsea_1.18.0
gwasrapidd_0.99.17
ggVennDiagram_1.5.2
vsn_3.60.0
VennDiagram_1.7.3
futile.logger_1.4.3
gplots_3.2.0
biomaRt_2.48.3
geneplotter_1.70.0
annotate_1.70.0
XML_3.99-0.18
lattice_0.22-6
LSD_4.1-0
org.Hs.eg.db_3.13.0
AnnotationDbi_1.54.1
DESeq2_1.32.0
SummarizedExperiment_1.22.0
Biobase_2.52.0
MatrixGenerics_1.4.3
matrixStats_1.5.0
sf_1.0-12
rtracklayer_1.52.1
GenomicRanges_1.44.0
GenomeInfoDb_1.28.4
IRanges_2.26.0
S4Vectors_0.30.
BiocGenerics_0.38.0
Hmisc_5.0-1
class_7.3-23
Rsamtools_2.8.0
lmtest_0.9-40
foreach_1.5.2
crayon_1.5.3
nlme_3.1-162
backports_1.5.0
GOSemSim_2.18.1
rlang_1.1.4
limma_3.48.3
filelock_1.0.3
BiocParallel_1.26.2
rjson_0.2.23
bit64_4.5.2
glue_1.8.0
rngtools_1.5.2
motifStack_1.36.1
classInt_0.4-9
DOSE_3.18.3
tidyselect_1.2.1
zoo_1.8-12

GenomicAlignments_1.28.0
xtable_1.8-4
magrittr_2.0.3
evaluate_1.0.1
quantmod_0.4.27
cli_3.6.3
zlibbioc_1.38.0
rstudioapi_0.17.1
furrr_0.3.1
bslib_0.8.0
rpart_4.1.24
fastmatch_1.1-6
ensembldb_2.16.4
lambda.r_1.2.4
methylPipe_1.26.0
treeio_1.16.2
xfun_0.50
cluster_2.1.8
urca_1.3-3
caTools_1.18.3
tidygraph_1.2.3
KEGGREST_1.32.0
ggrepel_0.9.6
biovizBase_1.40.0
ape_5.8-1
listenv_0.9.1
future_1.34.0
TFMPvalue_0.0.9
png_0.1-8
withr_3.0.2
bitops_1.0-9
ggforce_0.4.2
plyr_1.8.9
AnnotationFilter_1.16.0
e1071_1.7-16
pillar_1.10.1
cachem_1.1.0
fs_1.6.5
TTR_0.24.3
xts_0.13.1
vctrs_0.6.5
generics_0.1.3
NMF_0.28
tools_4.1.0
foreign_0.8-87
munsell_0.5.1
tweenr_2.0.3
proxy_0.4-27
DelayedArray_0.18.0
fastmap_1.2.0
compiler_4.1.0
abind_1.4-8
TxDb.Hsapiens.UCSC.hg19.knownGene_3.2.2
Gviz_1.36.2
GenomeInfoDbData_1.2.6
gridExtra_2.3
deldir_1.0-6
BiocFileCache_2.0.0
jsonlite_1.8.9
affy_1.70.0
tidytree_0.4.6
carData_3.0-5
genefilter_1.74.1
lazyeval_0.2.2
tseries_0.10-54
car_3.1-3
doParallel_1.0.17
latticeExtra_0.6-30
splitstackshape_1.4.8
checkmate_2.3.2
rmarkdown_2.29
cowplot_1.1.3
dichromat_2.0-0.1
igraph_1.4.2
survival_3.8-3
yaml_2.3.10

plotrix_3.8-4
htmltools_0.5.8.1
memoise_2.0.1
VariantAnnotation_1.38.0
BiocIO_1.2.0
locfit_1.5-9.10
quadprog_1.5-8
graphlayouts_1.2.1
viridisLite_0.4.2
digest_0.6.37
rappdirs_0.3.3
futile.options_1.0.1
registry_0.5-1
units_0.8-1
RSQLite_2.3.9
yulab.utils_0.1.9
data.table_1.16.4
fracdiff_1.5-2
blob_1.2.4
preprocessCore_1.54.0
splines_4.1.0
Formula_1.2-5
labeling_0.4.3
ProtGenerics_1.24.0
RCurl_1.98-1.16
broom_1.0.7
hms_1.1.3
colorspace_2.1-1
base64enc_0.1-3
BiocManager_1.30.25
aplot_0.1.10
nnet_7.3-20
sass_0.4.9
Rcpp_1.0.13-1
enrichplot_1.12.3
tzdb_0.4.0
parallelly_1.41.0
R6_2.5.1
lifecycle_1.0.4
formatR_1.14
curl_6.1.0
ggsignif_0.6.4
affyio_1.62.0
jquerylib_0.1.4
DO.db_2.9
qvalue_2.24.0
iterators_1.0.14
htmlwidgets_1.6.4
polyclip_1.10-7
shadowtext_0.1.4
timechange_0.3.0
gridGraphics_0.5-1
marray_1.70.0
mgcv_1.9-1
globals_0.16.3
htmlTable_2.4.3
patchwork_1.3.0
codetools_0.2-20
gtools_3.9.5
prettyunits_1.2.0
dbplyr_2.5.0
gridBase_0.4-7
gtable_0.3.6
DBI_1.2.3
ggfun_0.0.9
httr_1.4.7
KernSmooth_2.23-20
stringi_1.8.4
progress_1.2.3
reshape2_1.4.4
farver_2.1.2
viridis_0.6.5
fdrtool_1.2.18
timeDate_4041.110
ggtree_3.0.4
xml2_1.3.6

boot_1.3-31
restfulr_0.0.15
interp_1.1-4
ade4_1.7-22
ggplotify_0.1.2
bit_4.5.0.1
scatterpie_0.2.4
jpeg_0.1-10
ggraph_2.2.1
pkgconfig_2.0.3
rstatix_0.7.2
knitr_1.49

For manuscripts utilizing custom algorithms or software that are central to the research but not yet described in published literature, software must be made available to editors and reviewers. We strongly encourage code deposition in a community repository (e.g. GitHub). See the Nature Portfolio guidelines for submitting code & software for further information.

Data

Policy information about availability of data

All manuscripts must include a data availability statement. This statement should provide the following information, where applicable:

- Accession codes, unique identifiers, or web links for publicly available datasets
- A description of any restrictions on data availability
- For clinical datasets or third party data, please ensure that the statement adheres to our policy

We considered published ChIP-seq (CTCF data E-MTAB-5732 CTCF_ES_2i_rep1_CTCF, CTCF_ES_2i_rep2_CTCF and CTCF_NS_rep1_CTCF, CTCF_NS_rep2_CTCF) and Hi-C (GEO: GSE96107, Hi-C from the ES cells, and Hi-C from the NPC). Below in this form, we provide the links to access the fastq, .hic, and .bw files corresponding to data generated in this study.
Accession Link Data
E-MTAB-13559 https://www.ebi.ac.uk/biostudies/arrayexpress/studies/E-MTAB-13559?key=7556bb20-779d-4fa8-af58-1640e6b39eac ATAC-seq
E-MTAB-13562 https://www.ebi.ac.uk/biostudies/arrayexpress/studies/E-MTAB-13562?key=20b6208b-c9c4-4f58-81b4-ce55d7b8b8f2 CTCF ChIP-seq
E-MTAB-13560 https://www.ebi.ac.uk/biostudies/arrayexpress/studies/E-MTAB-13560?key=68cacd70-0f56-4cb2-a490-3e1cd549b425 H3K27ac ChIP-seq
E-MTAB-13558 https://www.ebi.ac.uk/biostudies/arrayexpress/studies/E-MTAB-13558?key=c94828b4-4f13-4562-bceb-08359ac76caa RNA-seq
E-MTAB-13572 https://www.ebi.ac.uk/biostudies/arrayexpress/studies/E-MTAB-13572?key=01a4fafe-71a4-461a-8d1f-75420ff1779e Hi-C
The directories above do not need password, the links will take the reviewer directly to the relevant folders.PXD048470 Username: reviewer_pxd048470@ebi.ac.uk ; Password: l01UjlYF SICAP data deposited on https://www.ebi.ac.uk/pride/

Research involving human participants, their data, or biological material

Policy information about studies with human participants or human data. See also policy information about sex, gender (identity/presentation), and sexual orientation and race, ethnicity and racism.

Reporting on sex and gender	NA
Reporting on race, ethnicity, or other socially relevant groupings	NA
Population characteristics	NA
Recruitment	NA
Ethics oversight	NA

Note that full information on the approval of the study protocol must also be provided in the manuscript.

Field-specific reporting

Please select the one below that is the best fit for your research. If you are not sure, read the appropriate sections before making your selection.

☒ Life sciences ☐ Behavioural & social sciences ☐ Ecological, evolutionary & environmental sciences

For a reference copy of the document with all sections, see nature.com/documents/nr-reporting-summary-flat.pdf

Life sciences study design

All studies must disclose on these points even when the disclosure is negative.

Sample size	No statistical method was used to pre-determine the sample size. Instead, for each experiment, the sample size was chosen based on established literature methods and our personal experiences. We considered at least two individual clones per each genetic modification. We performed experiments in replicates (PLA, at least two individual experiments, RNA-seq 2-4 replicate experiments, microscopy 2-3 replicate

experiments. We included an experiment whereby the ES and NS wild type and mutant cells were analyzed using Airyscan microscopy in one lab and by STED microscopy in another lab. The experiment was designed to challenge our observations on the impact of differentiation as well as Ddx5 and FUS on CTCF clustering. We encoded the sample labels and the experimentalist in the other lab acquired the images without knowing to which group the samples belonged to). Sequencing data (RNA-seq, ChIP-seq, ATAC-seq and Hi-C) was scrutinized for quality. ChIP-SICAP was performed in two replicates.

Data exclusions

We screened for quality of sequencing data based on fastqc, ChIP-seq peak numbers, ChIP or ATAC-seq signal strength, Hi-C long-to-short range interactions. If needed, we excluded samples showing poor number of ChIP-seq peaks (<5000, one sample in this project) and non satisfactory signal (high noise, unclear peaks). In Hi-C the reason to eliminate was a low ratio of long range to short range interactions.

Replication

Most experiments were independently repeated two to three times with successful replication. RNA-seq experiments were done in multipe (2-4) independent replicates. We included clonal expanded wild type cells as controls (in experiments such as Hi-C, ChIP-seq, PLA).

Randomization

This study did not utilize randomization, as it is not relevant to cell line studies.

Blinding

Blinding was not implemented since the experiments involved a limited number of investigators. In microscopy experiments, images were collected using consistent channel parameters and laser settings across all groups within the same experimental set. To confirm the findings, all experiments were independently repeated several times.

Reporting for specific materials, systems and methods

We require information from authors about some types of materials, experimental systems and methods used in many studies. Here, indicate whether each material, system or method listed is relevant to your study. If you are not sure if a list item applies to your research, read the appropriate section before selecting a response.

Materials & experimental systems

n/a	Involved in the study	
☐	☒	Antibodies
☐	☒	Eukaryotic cell lines
☒	☐	Palaeontology and archaeology
☒	☐	Animals and other organisms
☒	☐	Clinical data
☒	☐	Dual use research of concern
☒	☐	Plants

Methods

n/a	Involved in the study	
☐	☒	ChIP-seq
☐	☒	Flow cytometry
☒	☐	MRI-based neuroimaging

Antibodies

Antibodies used

Rabbit polyclonal anti-CTCF Merck 07-729 (5ul per 10 million cells)
Rabbit polyclona anti-CTCF CST 2899S (1:2000)
Mouse monoclonal anti-CTCF Santa Cruz sc-271474 (1:50)
Rabbit polyclonal anti-H3K27ac Cell Signalling 8173S (1:100)
Rabbit polyclonal anti-FUS Bethyl laboratory A300-294A (1:10000)
Mouse monoclonal anti-FUS Santa Cruz sc-47711 (1:50, 1:1000)
Rabbit polyclonal anti-FUS Proteintech 11570-1-AP (1:50)
Rabbit Polyclonal anti-NONO Proteintech 11058-1-AP (1:50, 1:1000)
Mouse monoclonal anti-OCT4 Santa Cruz sc-5279 (1:400)
Mouse monoclonal anti-Beta-actin Proteintech 66009-1-Ig (1:5000)
Rabbit monoclonal anti-TBP (D5C9H) CST #44059 (1:1000)
Mouse monoclonal anti-Nestin Developmental Studies Hybridoma Bank RAT-401 (1:100)
Rabbit polyclonal anti-DDX5 Bethyl laboratory A300-523A (1:5000)
Rabbit polyclonal anti-DDX5 Proteintech 26385-1-AP (1:50)
Mouse monoclonal anti-TUBB3 Proteintech 66375-1-Ig (1:100)
Rabbit polyclonal anti-GFAP Proteintech 16825-1-AP (1:100)
PE Rat Anti-Mouse CD44 BD Pharmingen™ 553134 (1:200)
Goat anti-Mouse IgG (H+L) Cross-Adsorbed Secondary Antibody, Alexa Fluor™ 488 Thermo Fisher A-11001 (1:1000)
Goat anti-Mouse IgG (H+L) Cross-Adsorbed Secondary Antibody, Alexa Fluor™ 568 Thermo Fisher A-11011 (1:1000)
Goat anti-Mouse IgG (H+L) Cross-Adsorbed Secondary Antibody, Alexa Fluor™ 647 Thermo Fisher A-21244 (1:1000)
IRDye 800CW Goat anti-Rabbit IgG Secondary Antibody Li-cor 925-32211 (1:15000)
IRDye 680RD Goat anti-Mouse IgG Secondary Antibody Li-cor 925-68070 (1:15000)

Validation

Rabbit polyclonal anti-CTCF Merck 07-729
Validation for species: Homo sapiens, Mus musculus, rattus norvegicus, Macaca mulatta, Canis lupus familiaris
Validation for application: Chromatin immunoprecipitation, Western blot
Validation statements on manufacturer's website: None provided
Relevant citations: Anderson, C.J., Talmane, L., Luft, J. et al. Strand-resolved mutagenicity of DNA damage and repair. Nature 630, 744–751 (2024). https://doi.org/10.1038/s41586-024-07490-1
Online database entries: https://www.citeab.com/
Data in your manuscript: The antibody was used for ChIP-seq experiment

Rabbit polyclonal anti-CTCF CST 2899S
Validation for species: Homo sapiens, Mus musculus, rattus norvegicus, Macaca mulatta Validation for application: Western blot, Chromatin immunoprecipitation
Validation statements on manufacturer's website: Western blot analysis of extracts from various cell lines using CTCF Antibody (cell lines used: HeLa, NIH/3t3, C6, COS).
Relevant citations: Choi EH, Kim KP. Cohesin and condensin regulate chromosome topology and play an essential role in maintaining pluripotency in embryonic stem cells. Sci Rep. 2025 Mar 22;15(1):9918. doi: 10.1038/s41598-025-94533-w. PMID: 40121293; PMCID: PMC11929898.
Online database entries: https://www.citeab.com/
Data in your manuscript: The antibody was used in western blot for CTCF-HALO knock-in cells validation

Mouse monoclonal anti-CTCF Santa Cruz sc-271474
Validation for species: Homo sapiens, Mus musculus, rattus norvegicus
Validation for application: Immunofluorecence, Western blot, Chromatin immunoprecipitation
Validation statements on manufacturer's website: None provided
Relevant citations: Andreu MJ, Alvarez-Franco A, Portela M, Gimenez-Llorente D, Cuadrado A, Badia-Careaga C, Tiana M, Losada A, Manzanares M. Establishment of 3D chromatin structure after fertilization and the metabolic switch at the morula-to-blastocyst transition require CTCF. Cell Rep. 2022 Oct 18;41(3):111501. doi: 10.1016/j.celrep.2022.111501. PMID: 36260992.
Online database entries: https://www.citeab.com/
Data in your manuscript: The antibody was used in immunofluorescence assay for PLA experiment

Rabbit polyclonal anti-H3K27ac Cell Signalling 8173S
Validation for species: Homo sapiens, Mus musculus, rattus norvegicus, chlorocebus sabaeus
Validation for application: Immunofluorecence, Western blot, Chromatin immunoprecipitation
Validation statements on manufacturer's website: Chromatin immunoprecipitations were performed with cross-linked chromatin from HeLa cells and Acetyl-Histone H3 (Lys27) (D5E4) XP® Rabbit mAb, using SimpleChIP® Plus Enzymatic Chromatin IP Kit (Magnetic Beads) #9005.
Relevant citations: Weng, Y., Feng, Y., Li, Z. et al. Zfp260 choreographs the early stage osteo-lineage commitment of skeletal stem cells. Nat Commun 15, 10186 (2024). https://doi.org/10.1038/s41467-024-54640-0
Online database entries: https://www.citeab.com/
Data in your manuscript: The antibody was used in ChIP-seq experiment

Rabbit polyclonal anti-FUS Bethyl laboratory A300-294A
Validation for species: Homo sapiens, Mus musculus
Validation for application: Western blot, immunoprecipitation, immunohistochemistry
Validation statements on manufacturer's website: None provided
Relevant citations: Scekic-Zahirovic, J., Sanjuan-Ruiz, I., Kan, V. et al. Cytoplasmic FUS triggers early behavioral alterations linked to cortical neuronal hyperactivity and inhibitory synaptic defects. Nat Commun 12, 3028 (2021). https://doi.org/10.1038/s41467-021-23187-9
Online database entries: https://www.citeab.com/
Data in your manuscript: The antibody was used in western blot for Fus knock-out cells validation

Mouse monoclonal anti-FUS Santa Cruz sc-47711
Validation for species: Homo sapiens, Mus musculus, rattus norvegicus
Validation for application: Western blot, immunoprecipitation, immunohistochemistry
Validation statements on manufacturer's website: None provided
Relevant citations:
1. Hock EM, Maniecka Z, Hruska-Plochan M, Reber S, Laferrière F, Sahadevan M K S, Ederle H, Gittings L, Pelkmans L, Dupuis L, Lashley T, Ruepp MD, Dormann D, Polymenidou M. Hypertonic Stress Causes Cytoplasmic Translocation of Neuronal, but Not Astrocytic, FUS due to Impaired Transportin Function. Cell Rep. 2018 Jul 24;24(4):987-1000.e7. doi: 10.1016/j.celrep.2018.06.094. PMID: 30044993.
2. Lagier-Tourenne, C., Polymenidou, M., Hutt, K. et al. Divergent roles of ALS-linked proteins FUS/TLS and TDP-43 intersect in processing long pre-mRNAs. Nat Neurosci 15, 1488–1497 (2012). https://doi.org/10.1038/nn.3230
Online database entries: https://www.citeab.com/
Data in your manuscript: The antibody was used PLA and western blot experiments

Rabbit polyclonal anti-FUS Proteintech 11570-1-AP
Validation for species: Homo sapiens, Mus musculus, rattus norvegicus
Validation for application: Western blot, immunoprecipitation, immunohistochemistry
Validation statements on manufacturer's website: Knockdown- Western blot result of FUS antibody (11570-1-AP, 1:5000) with si-Control and si-FUS transfected HEK 293 cells.
Relevant citations: Scekic-Zahirovic, J., Sanjuan-Ruiz, I., Kan, V. et al. Cytoplasmic FUS triggers early behavioral alterations linked to cortical neuronal hyperactivity and inhibitory synaptic defects. Nat Commun 12, 3028 (2021). https://doi.org/10.1038/s41467-021-23187-9
Online database entries: https://www.citeab.com/
Data in your manuscript: The antibody was used PLA experiments

Rabbit Polyclonal anti-NONO Proteintech 11058-1-AP
Validation for species: Homo sapiens, Mus musculus, rattus norvegicus
Validation for application: Western blot, immunoprecipitation, immunofluorescence
Validation statements on manufacturer's website: Knockdown
Relevant citations: Qin Y, Chen W, Jiang G, Zhou L, Yang X, Li H, He X, Wang HL, Zhou YB, Huang S, Liu S. Interfering MSN-NONO complex-activated CREB signaling serves as a therapeutic strategy for triple-negative breast cancer. Sci Adv. 2020 Feb 19;6(8):eaaw9960. doi: 10.1126/sciadv.aaw9960. PMID: 32128390; PMCID: PMC7030932.
Online database entries: https://www.citeab.com/
Data in your manuscript: The antibody was used PLA experiments

Mouse monoclonal anti-OCT4 Santa Cruz sc-5279
Validation for species: Homo sapiens, Mus musculus, rattus norvegicus
Validation for application: Western blot, immunoprecipitation, immunofluorescence
Validation statements on manufacturer's website: None provided
Relevant citations: Li W, Karwacki-Neisius V, Ma C, Tan L, Shi Y, Wu F, Shi YG. Nono deficiency compromises TET1 chromatin association and impedes neuronal differentiation of mouse embryonic stem cells. Nucleic Acids Res. 2020 May 21;48(9):4827-4838. doi: 10.1093/nar/gkaa213. PMID: 32286661; PMCID: PMC7229820.
Online database entries: https://www.citeab.com/
Data in your manuscript: The antibody was used immunofluorescence assay for ES cells validation

Mouse monoclonal anti-Nestin Developmental Studies Hybridoma Bank RAT-401
Validation for species: Mus musculus, rattus norvegicus
Validation for application: Western blot, immunoprecipitation, immunocytochemistry
Validation statements on manufacturer's website: None provided
Relevant citations: Eder N, Roncaroli F, Domart MC, Horswell S, Andreiuolo F, Flynn HR, Lopes AT, Claxton S, Kilday JP, Collinson L, Mao JH, Pietsch T, Thompson B, Snijders AP, Ultanir SK. Author Correction: YAP1/TAZ drives ependymoma-like tumour formation in mice. Nat Commun. 2020 Sep 28;11(1):4934. doi: 10.1038/s41467-020-18851-5. Erratum for: Nat Commun. 2020 May 13;11(1):2380. doi: 10.1038/s41467-020-16167-y. PMID: 32985498; PMCID: PMC7522079.
Online database entries: https://www.citeab.com/
Data in your manuscript: The antibody was used immunofluorescence assay for NS cells validation

Rabbit polyclonal anti-DDX5 Bethyl laboratory A300-523A
Validation for species: Homo sapiens, Mus musculus
Validation for application: Western blot, immunoprecipitation, immunohistochemistry
Validation statements on manufacturer's website: None provided
Relevant citations: Saporita AJ, Chang HC, Winkeler CL, Apicelli AJ, Kladney RD, Wang J, Townsend RR, Michel LS, Weber JD. RNA helicase DDX5 is a p53-independent target of ARF that participates in ribosome biogenesis. Cancer Res. 2011 Nov 1;71(21):6708-17. doi: 10.1158/0008-5472.CAN-11-1472. Epub 2011 Sep 21. PMID: 21937682; PMCID: PMC3206203.
Online database entries: https://www.citeab.com/
Data in your manuscript: The antibody was used in a western blot for Ddx5 knock-out cells validation.

Rabbit Polyclonal anti-Ddx5 Proteintech 26385-1-AP
Validation for species: Homo sapiens, Mus musculus, rattus norvegicus
Validation for application: Western blot, immunoprecipitation, immunofluorescence
Validation statements on manufacturer's website: None provided
Relevant citations: None.
Online database entries: https://www.citeab.com/
Data in your manuscript: The antibody was used in PLA experiments

Mouse monoclonal anti-TUBB3 Proteintech 66375-1-Ig
Validation for species: Homo sapiens, Mus musculus, rattus norvegicus
Validation for application: Western blot, immunoprecipitation, immunofluorescence
Validation statements on manufacturer's website: None provided
Relevant citations: Zhang N, Ji Q, Chen Y, Wen X, Shan F. TREM2 deficiency impairs the energy metabolism of Schwann cells and exacerbates peripheral neurological deficits. Cell Death Dis. 2024 Mar 7;15(3):193. doi: 10.1038/s41419-024-06579-9. PMID: 38453910; PMCID: PMC10920707.
Online database entries: https://www.citeab.com/
Data in your manuscript: The antibody was used in immunofluorescence assay

Rabbit polyclonal anti-GFAP Proteintech 16825-1-AP
Validation for species: Homo sapiens, Mus musculus, rattus norvegicus
Validation for application: Western blot, immunoprecipitation, immunofluorescence
Validation statements on manufacturer's website: Knockdown
Relevant citations: Luo R, Hu X, Li X, Lei F, Liao P, Yi L, Zhang X, Zhou B, Jiang R. Dysfunctional astrocyte glutamate uptake in the hypothalamic paraventricular nucleus contributes to visceral pain and anxiety-like behavior in mice with chronic pancreatitis. Glia. 2024 Nov;72(11):2022-2037. doi: 10.1002/glia.24595. Epub 2024 Jul 24. PMID: 39046219.
Online database entries: https://www.citeab.com/
Data in your manuscript: The antibody was used in immunofluorescence assay

PE Rat Anti-Mouse CD44 BD Pharmingen™ 553134
Validation for species: Mus musculus
Validation for application: Flow cytometry/ Cell sorting (FC/FACS)
Validation statements on manufacturer's website: None provided
Relevant citations: Wang Z, Qin X, Hu D, Huang J, Guo E, Xiao R, Li W, Sun C, Chen G. Akkermansia supplementation reverses the tumor-promoting effect of the fecal microbiota transplantation in ovarian cancer. Cell Rep. 2022 Dec 27;41(13):111890. doi: 10.1016/j.celrep.2022.111890. PMID: 36577369.
Online database entries: https://www.citeab.com/
Data in your manuscript: The antibody was used in sorting cells to obtain a population of purified NS cells.

Eukaryotic cell lines

Policy information about cell lines and Sex and Gender in Research

Cell line source(s)	SOX1-GFP-puro mouse embryonic cells Austin Smith PMID: 12524553<br>CTCF-AID-GFP mouse embryonic cells Elphège Nora and Benoit Bruneau #EN52.9.1 PMID: 28525758<br>E14Tg2a mouse ES cells (ATCC – CRL1821)
Authentication	RNA-seq profiling, flow cytometry (acute degradation experiments, HALO staining), PCR-based genotyping, western blot, qRT-PCR, immunofluorescence.
Mycoplasma contamination	Mycoplasma contamination was checked using PCR. Cells were tested negative for mycoplasma contamination.
Commonly misidentified lines (See ICLAC register)	No commonly misidentified cell lines were used in this study

Plants

Seed stocks	NA
Novel plant genotypes	NA
Authentication	NA

ChIP-seq

Data deposition

☒ Confirm that both raw and final processed data have been deposited in a public database such as GEO.

☒ Confirm that you have deposited or provided access to graph files (e.g. BED files) for the called peaks.

Data access links
May remain private before publication.

Accession Link Data
E-MTAB-13562 https://www.ebi.ac.uk/biostudies/arrayexpress/studies/E-MTAB-13562?key=20b6208b-c9c4-4f58-81b4-ce55d7b8b8f2 CTCF ChIP-seq
E-MTAB-13560 https://www.ebi.ac.uk/biostudies/arrayexpress/studies/E-MTAB-13560?key=68cacd70-0f56-4cb2-a490-3e1cd549b425 H3K27ac ChIP-seq
The .bw files can be found here: https://drive.google.com/drive/folders/1g2EVmw6nDsLxGnordRNglnGGQvcx39y-?usp=sharing anyone having this link should be able to view these files
The .bed files are provided in Extende Table 3

Files in database submission

ChIP_Seq_H3K27ac_05-22_MusMus_ESC_AID_KI_CTCF-AID-GFP_OsTIR_TIGRE_Nora_2i_IAA_Rep_1
ChIP_Seq_H3K27ac_05-22_MusMus_ESC_AID_KI_CTCF-AID-GFP_OsTIR_TIGRE_Nora_2i_Rep_1
ChIP_Seq_H3K27ac_05-22_MusMus_es-NPC_AID_KI_CTCF-AID-GFP_OsTIR_TIGRE_Nora_IAA_Rep_1
ChIP_Seq_H3K27ac_05-22_MusMus_es-NPC_AID_KI_CTCF-AID-GFP_OsTIR_TIGRE_Nora_Rep_1
ChIP_Seq_H3K27ac_12-21_MusMus_es-ESC_MOD_SOX1-GFP_2i_Rep_1
ChIP_Seq_H3K27ac_12-21_MusMus_es-NPC_MOD_SOX1-GFP_Rep_1
ChIP_Seq_CTCF_03-23_MusMus_es-NPC_DDX5_KO_CTCF-Cterm_HALO_CB1_Rep_1
ChIP_Seq_CTCF_03-23_MusMus_es-NPC_DDX5_KO_CTCF-Cterm_HALO_CE10_Rep_1
ChIP_Seq_CTCF_05-22_MusMus_es-NPC_AID_KI_CTCF-AID-GFP_OsTIR_TIGRE_Nora_IAA_Rep_1
ChIP_Seq_CTCF_05-22_MusMus_es-NPC_AID_KI_CTCF-AID-GFP_OsTIR_TIGRE_Nora_Rep_1
ChIP_Seq_CTCF_06-23_MusMus_es-NPC_MOD_CTCF-Cterm_HALO_A3_Control_Rep_1
ChIP_Seq_CTCF_07-22_MusMus_ESC_AID_KI_CTCF-AID-GFP_OsTIR_TIGRE_2i-IAA_Rep_1
ChIP_Seq_CTCF_07-22_MusMus_ESC_AID_KI_CTCF-AID-GFP_OsTIR_TIGRE_2i_Rep_1
ChIP_Seq_CTCF_07-22_MusMus_ESC_DDX5_KO_CTCF-Cterm_HALO_CB1_Rep_1
ChIP_Seq_CTCF_07-22_MusMus_ESC_DDX5_KO_CTCF-Cterm_HALO_CE10_Rep_1
ChIP_Seq_CTCF_07-22_MusMus_ESC_MOD_CTCF-Cterm_HALO_Control_Rep_1
ChIP_Seq_CTCF_07-22_MusMus_ESC_MOD_CTCF-Cterm_HALO_Control_Rep_2
ChIP_Seq_CTCF_08-23_MusMus_es-NPC_DDX5_KO_CTCF-Cterm_HALO_CB1_Rep_2
ChIP_Seq_CTCF_08-23_MusMus_es-NPC_MOD_CTCF-Cterm_HALO_A3_Control_Rep_2
ChIP_Seq_CTCF_11-24_MusMus_es-NPC_DDX5_FKBP_KI_CTCF-Cterm_HALO_rep1_4F11_DMSO_Rep_1
ChIP_Seq_CTCF_11-24_MusMus_es-NPC_DDX5_FKBP_KI_CTCF-Cterm_HALO_rep1_4F11_dTAG13_Rep_1
ChIP_Seq_CTCF_11-24_MusMus_es-NPC_DDX5_FKBP_KI_CTCF-Cterm_HALO_rep2_4F11_DMSO_Rep_1
ChIP_Seq_CTCF_11-24_MusMus_es-NPC_DDX5_FKBP_KI_CTCF-Cterm_HALO_rep2_4F11_dTAG13_Rep_1
ChIP_Seq_CTCF_10-24_MusMus_es-NPC_Pantr1_KO_CTCF-Cterm_HALO_PB6_Rep_1

ChIP_Seq_CTCF_10-24_MusMus_es-NPC_Pantr1_KO_CTCF-Cterm_HALO_PE3_Rep_1

Genome browser session (e.g. UCSC)

https://genome.ucsc.edu/s/misbah/ChIP%20Data%20for%20Dehingia%20et.al

Methodology

Replicates

We included two replicates of CTCF ChIP-seq in wild type NS cells, two replicates of CTCF in 4F11 Ddx5-KI DMSO, two replicates of CTCF in 4F11 Ddx5-KI dTAG13, two replicates of CTCF in CB1 Ddx5-/- KO clone (two independent experiments), one replicate in CE10 clone, one replicate of Pantr1-/- KO PB6 clone and one replicate of Pantr1-/- KO PE3 clone. In the case of CTCF and H3K27ac ChIP-seq, we included one replicate per condition (CTCF+/- ES and NS cells, as well as 46C ES and NS cells, CTCF C-term ES and NS cells). The H3K27ac profiles in the wild type and treated cells are similar which further testifies the robustness of our protocol and approach.

Sequencing depth

Sample Number of Reads Uniquely Mapped Reads Read Length Type Percentage Aligned
ChIP_Seq_CTCF_03-23_MusMus_es-NPC_DDX5_KO_CTCF-Cterm_HALO_CB1_Rep_1 20034552 12539546 151 Paired End 62.58960021
ChIP_Seq_CTCF_03-23_MusMus_es-NPC_DDX5_KO_CTCF-Cterm_HALO_CE10_Rep_1 25819122 16863362 151 Paired End 65.31346031
ChIP_Seq_CTCF_05-22_MusMus_es-NPC_AID_KI_CTCF-AID-GFP_OsTIR_TIGRE_Nora_IAA_Rep_1 15054295 11437288 151 Paired End 75.9735876
ChIP_Seq_CTCF_05-22_MusMus_es-NPC_AID_KI_CTCF-AID-GFP_OsTIR_TIGRE_Nora_Rep_1 13254900 9340023 151 Paired End 70.46468099
ChIP_Seq_CTCF_06-23_MusMus_es-NPC_MOD_CTCF-Cterm_HALO_A3_Control_Rep_1 8361728 5705039 151 Paired End 68.22799067
ChIP_Seq_CTCF_07-22_MusMus_ESC_AID_KI_CTCF-AID-GFP_OsTIR_TIGRE_2i_Rep_1 25636732 16321253 151 Paired End 63.66354729
ChIP_Seq_CTCF_07-22_MusMus_ESC_AID_KI_CTCF-AID-GFP_OsTIR_TIGRE_2i-IAA_Rep_1 27292975 18308567 151 Paired End 67.08160983
ChIP_Seq_CTCF_07-22_MusMus_ESC_DDX5_KO_CTCF-Cterm_HALO_CB1_Rep_1 8481250 5944263 151 Paired End 70.0871098
ChIP_Seq_CTCF_07-22_MusMus_ESC_MOD_CTCF-Cterm_HALO_Control_Rep_1 11218171 8662309 151 Paired End 77.21676733
ChIP_Seq_CTCF_07-22_MusMus_ESC_MOD_CTCF-Cterm_HALO_Control_Rep_2 8577518 6688281 151 Paired End 77.97454928
ChIP_Seq_CTCF_08-23_MusMus_es-NPC_DDX5_KO_CTCF-Cterm_HALO_CB1_Rep_2 10802482 6774961 151 Paired End 62.71670714
ChIP_Seq_CTCF_08-23_MusMus_es-NPC_MOD_CTCF-Cterm_HALO_A3_Control_Rep_2 11063929 6912810 151 Paired End 62.48060702
ChIP_Seq_H3K27ac_05-22_MusMus_es-NPC_AID_KI_CTCF-AID-GFP_OsTIR_TIGRE_Nora_IAA_Rep_1 19036938 15477223 151 Paired End 81.3010107
ChIP_Seq_H3K27ac_05-22_MusMus_es-NPC_AID_KI_CTCF-AID-GFP_OsTIR_TIGRE_Nora_Rep_1 16182016 12976473 151 Paired End 80.19070677
ChIP_Seq_H3K27ac_05-22_MusMus_ESC_AID_KI_CTCF-AID-GFP_OsTIR_TIGRE_Nora_2i_IAA_Rep_1 21247453 16415672 151 Paired End 77.25948141
ChIP_Seq_H3K27ac_05-22_MusMus_ESC_AID_KI_CTCF-AID-GFP_OsTIR_TIGRE_Nora_2i_Rep_1 16874071 13389448 151 Paired End 79.34924536
ChIP_Seq_H3K27ac_12-21_MusMus_es-ESC_MOD_SOX1-GFP_2i_Rep_1 27439533 21272692 151 Paired End 77.52570716
ChIP_Seq_H3K27ac_12-21_MusMus_es-NPC_MOD_SOX1-GFP_Rep_1 33580031 25807306 151 Paired End 76.85313334
ChIP_Seq_CTCF_11-24_MusMus_es-NPC_DDX5_FKBP_KI_CTCF-Cterm_HALO_4F11_DMSO-Rep1_Rep_1 11288541 8459434 151 Paired End 74.938240
ChIP_Seq_CTCF_11-24_MusMus_es-NPC_DDX5_FKBP_KI_CTCF-Cterm_HALO_4F11_DMSO-Rep2_Rep_1 11789249 8636620 151 Paired End 73.2584408
ChIP_Seq_CTCF_11-24_MusMus_es-NPC_DDX5_FKBP_KI_CTCF-Cterm_HALO_4F11_dTAG13-Rep1_Rep_1 13838852 10050705 151 Paired End 72.6267251
ChIP_Seq_CTCF_11-24_MusMus_es-NPC_DDX5_FKBP_KI_CTCF-Cterm_HALO_4F11_dTAG13-Rep2_Rep_1 10549946 8455358 151 Paired End 80.1459837
ChIP_Seq_CTCF_10-24_MusMus_es-NPC_Pantr1_KO_CTCF-Cterm_HALO_PB6_Rep_1 16449957 10287956 151 Paired End 62.5409294
ChIP_Seq_CTCF_10-24_MusMus_es-NPC_Pantr1_KO_CTCF-Cterm_HALO_PE3_Rep_1 21050862 15761657 151 Paired End 74.8741643

Antibodies

Rabbit polyclonal anti-CTCF Merck 07-729 (5 ul/10 million cells)
Rabbit polyclonal anti-H3K27ac Cell Signalling 8173S (1:100)

Peak calling parameters

Raw reads were trimmed using TrimGalore version 0.6.7, using parameters '--paired -q 30 --stringency 3 --length 30' and alignment was performed using bowtie2 using parameters '--very- sensitive -X 2000'. All the ATAC-Seq, H3K27ac ChIP-Seq, CTCF ChIP-Seq data were aligned to the Mus musculus (mm10/GRCm38) genome. The alignments were filtered to remove duplicates using alignmentSieve (using parameters '--minFragmentLength 40 –ignoreDuplicates') which is available as a part of the deeptools package version 3.5.1

Peak calling was performed using MACS2 (Model-based Analysis for ChIP-Seq) ver. 2.2.7.1 using parameters '--no-model'. The effective genome size required as one of the input parameters for the program was kept at default for mice. RPGC normalised files were obtained by.

Data quality

Each sequence library was checked with fastQC. Then, having the processed files we assessed the quality of the data. We judged the signal to noise ratio, the shapes of peaks of enrichment (CTCF versus histone modifications). We included peaks called by MACS2 at a 0.1 FDR cutoff. Furthermore, the quality of the CTCF ChIP-seq was judged based on the previous knowledge (location of active

	regions, known regions bound by CTCF). Likewise, we generated heatmaps of ChIP-signal aroud peak summits, average profile of CTCF ChIP-signal around TSS. We also displayed the CTCF data in relation with the published Hi-C (orientation of CTCF bound motifs at TAD borders - as expected, over 85% of CTCF peaks at TAD borders were oriented inwards with respect to the interior of the domain (only TAD borders featuring 1 CTCF peak were included in this sanity check)). Similar, knowledge-based approach was used in the case of H3K27ac data. We checked the correlation between H3K27ac signal around transcription start sites and gene expression (we observed a positive correlation, as expected). We also addressed the H3K27ac enrichment at known distal regulatory elements.
Software	The raw data was analyzed using a pipeline made with the Snakemake framework, where the data is first trimmed for adapters using TrimGalore, followed by alignment to the GRCm38/mm10 genome using bowtie2. BigWig tracks for visualization are created using the bamCoverage tool from the deeptools v3.5 suite and peaks are called using MACS2. Blacklisted regions were removed from consideration.

Flow Cytometry

Plots

Confirm that:

☒ The axis labels state the marker and fluorochrome used (e.g. CD4-FITC).

☒ The axis scales are clearly visible. Include numbers along axes only for bottom left plot of group (a 'group' is an analysis of identical markers).

☒ All plots are contour plots with outliers or pseudocolor plots.

☒ A numerical value for number of cells or percentage (with statistics) is provided.

Methodology

Sample preparation	For Cell sorting: Cells were detached from the culture plastic using Accutase. Then, the cell pellet was washed once with PBS. Cells were then incubated with blocking buffer (0,5% BSA-PBS) for 30 min at 4ºC. Wash the cells once and incubate with CD44 antibody (1:200 BD Pharmingen™ PE Rat Anti-Mouse CD44, 553134) for 40 min at 4ºC. Wash twice with DPBS, and cells were sorted with Cell sorter BD FACSAriaII.<br>For Flow cytometry analysis of the CTCF level: CTCF-HALO cells were seeded on laminin (Sigma-Merck, L2020-1MG)-coated plastic. 24h later, cells were incubated with 5μM Tetramethylrhodamine (TMR), a HaloTag ligand (Promega, G8252) at 37°C for 30 min. Cells were washed three times with fresh media and incubated at 37°C for 30 min in the cell culture medium followed by additional wash with fresh media. Cells were then detached by accutase and analysed by BD FACSAriaII<br>Flow cytometry of CTCF-EGFP-AID cells. Untreated and IAA treated cells were detached by accutase, washed with PBS and processed by BD Facs Calibur. The FL-1 signal (GFP) was analysed in cell population based on FSC and SSC signal.
Instrument	BD FACSAriaII, BD Facs Calibur
Software	BD FACSDiva version 8.0.1<br>FlowJo version 10.8.1
Cell population abundance	NS in this study were all CD44+ cells. We enriched for this population using flow cytometry, an example of a strategy to purify this cell population is shown in Extended Fig. 3b.
Gating strategy	Cells were identified based on FSC-A and SSC-A. Single cells were gated based on FSC-W and FSC-H and SSC-W and SSC-H. The CD44 cells (PE) were identified based on the PE-A versus SSC-A (Extended Fig. 3b, Methods).

☒ Tick this box to confirm that a figure exemplifying the gating strategy is provided in the Supplementary Information.
