# Supplementary material for: RNA-binding proteins mediate the maturation of chromatin topology during differentiation
Source: Nat Cell Biol. 2025 Sep 8;27(9):1510–25. doi: 10.1038/s41556-025-01735-5 (PMC12431861; doi:10.1038/s41556-025-01735-5)
Supplement: Supplementary file 2 — Vignette and html with the R code. [file 41556_2025_1735_MOESM2_ESM.zip › software_vignette/Complete_analysis.html]

RNA binding proteins mediate the maturation of chromatin topology during differentiation


# RNA binding proteins mediate the maturation of chromatin topology during differentiation

#### Aleksandra Pekowska, Misbah Abbas

- Library and data import
- Building objects for future work
- Loops from Bonev et al
- Analysis of transcriptome with and without CTCF (ES and NS cells)
  - ES–>NS validation
  - CTCF targets cell type specific genes
  - Aldh1a3 expression plot
  - Aldh1a3 CTCF-BS KOs
- Expression of up and down regulated genes
- Chromatin signature with and without CTCF - ATAC-seq and ChIP-seq data
  - Peak number changes and deregulation upon CTCF loss
- Peaks in ES and NS cells
- CTCF, gene expression, and loop gain
- Insulators in the NS cells
- PLA
- PLA : Ctcf-Nono
- PLA : FUS-Ddx5
- PANTR1 PLA Ddx5-CTCF
- PANTR1 PLA FUS-CTCF
- qRT-PCR
- CTCF clustering - AiryScan on pre-extracted nuclei
- CTCF peaks
  - CTCF AUC
  - Motif search
  - Factors enriched at CTCF sites losing CTCF signal in Ddx5-/- NS cells
  - Factors enriched at CTCF sites gaining CTCF signal in Ddx5-/- NS cells
  - CTCF motif strength
  - G4
- Load loops
- CTCF and G4 at loop anchors
- Ddx5 and CTCF at loop anchors
- Deep coverage data preparations
- Distance decline plot
- Loop analysis
- Loop signal
- Lost and gained loops in the wt and Ddx5-/- cells
- IPF loop signal in the intervals of genomic distances
- Examples of loops lost in the NS cells
  - APA - general
- 4F11 - global analysis of architectural loops
  - Loops
- Can we link loop loss to Ddx5 effect on CTCF?
  - dTAG13 and chromatin interactions
- Pantr1 - general Hi-C
- CTCF in Pantr1-/-
- APA in Pantr1 on loops lost in Ddx5
- Hi-C: Insulation
- Published data - insulation
- Pantr1 Insulation
- SICAP-ChIP
- Session Info

# Library and data import

```
data_directory='/Volumes/T7/Dehingia_et_al_2025/data/'
chipseq_directory='/Volumes/T7/Dehingia_et_al_2025/data/ChIP-and-ATAC-seq/'
rnaseq_directory='/Volumes/T7/Dehingia_et_al_2025/data/RNA-seq/'
hic_directory='/Volumes/T7/Dehingia_et_al_2025/data/Hi-C/'
outputs_directory = '/Volumes/T7/Dehingia_et_al_2025/data/outputs/'
objects_directory = '/Volumes/T7/Dehingia_et_al_2025/data/objects/'
scripts_directory = '/Volumes/T7/Dehingia_et_al_2025/scripts/'
source_data_directory = '/Volumes/T7/Dehingia_et_al_2025/source_files/'

library(rtracklayer)
```

```
## Warning: pakiet 'rtracklayer' został zbudowany w wersji R 4.1.1
```

```
## Warning: pakiet 'S4Vectors' został zbudowany w wersji R 4.1.1
```

```
## Warning: pakiet 'GenomeInfoDb' został zbudowany w wersji R 4.1.1
```

```
library(sf)
```

```
## Warning: pakiet 'sf' został zbudowany w wersji R 4.1.2
```

```
library(DESeq2)
```

```
## Warning: pakiet 'MatrixGenerics' został zbudowany w wersji R 4.1.1
```

```
library(org.Hs.eg.db)
library(LSD)
library(geneplotter)
library(biomaRt)
```

```
## Warning: pakiet 'biomaRt' został zbudowany w wersji R 4.1.1
```

```
library(gplots)
library(VennDiagram)
```

```
## Warning: pakiet 'VennDiagram' został zbudowany w wersji R 4.1.2
```

```
library(vsn)
library(ggVennDiagram)
library(GenomicRanges)
library(gwasrapidd)
library(fgsea)
library(goseq)
library(ggplot2)
library(RColorBrewer)
```

```
## Warning: pakiet 'RColorBrewer' został zbudowany w wersji R 4.1.2
```

```
library(pheatmap)
library(TxDb.Mmusculus.UCSC.mm10.knownGene)
```

```
## Warning: pakiet 'GenomicFeatures' został zbudowany w wersji R 4.1.1
```

```
library(dplyr)
library(tidyverse)
```

```
## Warning: pakiet 'tidyverse' został zbudowany w wersji R 4.1.2
```

```
## Warning: pakiet 'tibble' został zbudowany w wersji R 4.1.2
```

```
## Warning: pakiet 'forcats' został zbudowany w wersji R 4.1.2
```

```
library(scales)
library(smoothmest)
```

```
## Warning: pakiet 'smoothmest' został zbudowany w wersji R 4.1.2
```

```
## Warning: pakiet 'MASS' został zbudowany w wersji R 4.1.2
```

```
library(BSgenome.Mmusculus.UCSC.mm10)
library(Matrix)
library(ggpubr)
```

```
## Warning: pakiet 'ggpubr' został zbudowany w wersji R 4.1.2
```

```
set.seed(22)

ms=12000*1024^2 
options(future.globals.maxSize=ms)

buildDfForGGBARPLOT = function( normCounts, metaTable, metaColumn, geneSymbol, mappings4genes ){
  # normCounts=ddsFdf; metaTable=as.data.frame(coldata);metaColumn="cell_type";geneSymbol="Aldh1a3";mappings4genes=genemap_unique
  theCol=which(colnames(metaTable)==metaColumn)
  data.frame( norm_counts = normCounts[rownames(normCounts) == unique(mappings4genes$gene_id[mappings4genes$geneName==geneSymbol])],
              annot = metaTable[ match(colnames(normCounts),metaTable$sample), theCol ] )}


source( paste0(scripts_directory,'functions.R' ) )
```

# Building objects for future work

```
gtf = import.gff(paste0(data_directory,'Mus_musculus.GRCm38.101.gtf'))

txdb = TxDb.Mmusculus.UCSC.mm10.knownGene
columns <- c("tx_name", "gene_id","GENEID")
promGR = promoters(txdb, upstream=200, downstream=200,columns=columns)
TSSGR = promoters(txdb, upstream=0, downstream=0,columns=columns)
promGRExt = promoters(txdb, upstream=2000, downstream=2000,columns=columns)
```

```
## Warning in valid.GenomicRanges.seqinfo(x, suggest.trim = TRUE): GRanges object contains 5 out-of-bound ranges located on sequences
##   chr4_GL456350_random, chr4_JH584293_random, chr4_JH584295_random,
##   chr5_JH584296_random, and chrUn_GL456239. Note that ranges located on a
##   sequence whose length is unknown (NA) or on a circular sequence are not
##   considered out-of-bound (use seqlengths() and isCircular() to get the
##   lengths and circularity flags of the underlying sequences). You can use
##   trim() to trim these ranges. See ?`trim,GenomicRanges-method` for more
##   information.
```

```
promGRExt=trim(promGRExt)

transcrpit2ensg = data.frame(tid=gtf$transcript_id,gid=gtf$gene_id,stringsAsFactors = FALSE)
promGRExt$transcipt1 = unlist(strsplit(promGRExt$tx_name,'\\.'))[seq(1,2*length(promGRExt),by=2)]
promGRExt$ensgeneid = transcrpit2ensg$gid[match(promGRExt$transcipt1,promGRExt$transcipt1)]
seqlevelsStyle(promGR)='ucsc'
genemap_unique = data.frame(gene_id=gtf$gene_id,geneName=gtf$gene_name,stringsAsFactors = FALSE)
genemap_unique = genemap_unique[!duplicated(genemap_unique$gene_id),]

promoters_ap = data.frame( chr=as.character(chrom(gtf)),
                           start=as.numeric(start(gtf)),
                           end=as.numeric(end(gtf)),
                           strand=as.character(strand(gtf)),
                           transcript_id=as.character(gtf$transcript_id),
                           gene_id=as.character(gtf$gene_id),
                           gene_name = as.character(gtf$gene_name),
                           gene_biotype = as.character(gtf$gene_biotype),
                           type = gtf$type,
                           stringsAsFactors = FALSE )
length(unique(promoters_ap$gene_id))
```

```
## [1] 55487
```

```
promoters_ap = promoters_ap[promoters_ap$type == "transcript",]
promoters_sp = split(promoters_ap,promoters_ap$gene_id)

## for each gene find the 5-prime most TSS
promoters_tss = do.call('rbind', lapply( promoters_sp, function(x){
    tss = ifelse( as.character(unique(x$strand))=="+", 
                  x[which.min(x$start),'start'], 
                  x[which.max(x$end),'end'] ) 
    if(  as.character(unique(x$strand))=="+") tp = x[which.min(x$start),] else tp = x[which.max(x$end),]
    tp$tss = tss
  return(tp)
  }))

## 
promoters_tss_gr = GRanges(seqnames = promoters_tss$chr,
                           ranges = IRanges(as.numeric(promoters_tss$tss)-500 ,
                                            end=as.numeric(promoters_tss$tss) + 500,
                                            names=promoters_tss$transcript_id),
                           strand = promoters_tss$strand,
                           gene_id = promoters_tss$gene_id,
                           gene_name = promoters_tss$gene_name,
                           gene_biotype = promoters_tss$gene_biotype,
                           tss = promoters_tss$tss)
seqlevelsStyle(promoters_tss_gr) = 'ucsc'
```

```
ctcf_motif = readBed_filterChromsStraded( paste0(data_directory,'CTCF_HUMAN.H11MO.0.A.bed'),
                                          chroms=paste0('chr',c(1:19,'X','Y')), 5 )
```

# Loops from Bonev et al

```
es_loops = importBEDPE_Filter_Loops( paste0(hic_directory, 'Bonev/hiccups_output_esc/' ),
                                     max_size=5000000, extend_anchors_by=0 )
ns_loops = importBEDPE_Filter_Loops( paste0(hic_directory, 'Bonev/hiccups_output_npc/' ),
                                     max_size=5000000, extend_anchors_by=0 )

es_loops_size=es_loops$y2-es_loops$x1
ns_loops_size=ns_loops$y2-ns_loops$x1

es_loops_left_anchor = getGR( es_loops$X.chr1, es_loops$x1, es_loops$x2,0 )
es_loops_right_anchor = getGR( es_loops$X.chr1, es_loops$y1, es_loops$y2,0 )

ns_loops_left_anchor = getGR( ns_loops$X.chr1, ns_loops$x1, ns_loops$x2,0 )
ns_loops_right_anchor = getGR( ns_loops$X.chr1, ns_loops$y1, ns_loops$y2,0 )

seqlevelsStyle(es_loops_left_anchor) = 'ucsc'
seqlevelsStyle(es_loops_right_anchor) = 'ucsc'
seqlevelsStyle(ns_loops_left_anchor) = 'ucsc'
seqlevelsStyle(ns_loops_right_anchor) = 'ucsc'

es_anchors_GR = c( es_loops_left_anchor, es_loops_right_anchor )
ns_anchors_GR = c( ns_loops_left_anchor, ns_loops_right_anchor )

es_ns_loops = compareLoops( es_loops, ns_loops, offset=5000, maxLoopSize=5000000 )
es_spe_loops = es_ns_loops$First_set_specific
ns_spe_loops = es_ns_loops$Second_set_specific
common_loops = es_ns_loops$First_set_common
es_spe_loops_size=es_spe_loops$y2-es_spe_loops$x1
ns_spe_loops_size=ns_spe_loops$y2-ns_spe_loops$x1


es_spe_loops_left_anchor = getGR( es_spe_loops$X.chr1, es_spe_loops$x1, es_spe_loops$x2,0 )
es_spe_loops_right_anchor = getGR( es_spe_loops$X.chr1, es_spe_loops$y1, es_spe_loops$y2,0 )

ns_spe_loops_left_anchor = getGR( ns_spe_loops$X.chr1, ns_spe_loops$x1, ns_spe_loops$x2,0 )
ns_spe_loops_right_anchor = getGR( ns_spe_loops$X.chr1, ns_spe_loops$y1, ns_spe_loops$y2,0 )

en_com_loops_left_anchor = getGR( common_loops$X.chr1, common_loops$x1, common_loops$x2,0 )
en_com_loops_right_anchor = getGR( common_loops$X.chr1, common_loops$y1, common_loops$y2,0 )

es_spe_loops_gr = getGR( es_spe_loops$X.chr1, es_spe_loops$x1, es_spe_loops$y2,0 )
ns_spe_loops_gr = getGR( ns_spe_loops$X.chr1, ns_spe_loops$x1, ns_spe_loops$y2,0 )
cm_loops_gr = getGR( common_loops$X.chr1, common_loops$x1, common_loops$y2,0 )

es_spe_loops_size=width(es_spe_loops_gr)
ns_spe_loops_size=width(ns_spe_loops_gr)

es_loops_gr = getGR( es_loops$X.chr1,es_loops$x1, es_loops$y2, 0 )
ns_loops_gr = getGR( ns_loops$X.chr1,ns_loops$x1, ns_loops$y2, 0 )

es_spe_loops_domain_gr = getGR( es_spe_loops$X.chr1, es_spe_loops$x1+25000, es_spe_loops$y2-25000,0 )
ns_spe_loops_domain_gr = getGR( ns_spe_loops$X.chr1, ns_spe_loops$x1+25000, ns_spe_loops$y2-25000,0 )
common_loops_domain_gr = getGR( common_loops$X.chr1, common_loops$x1+25000, common_loops$y2-25000,0 )

seqlevelsStyle(es_spe_loops_left_anchor) = 'ucsc'
seqlevelsStyle(es_spe_loops_right_anchor) = 'ucsc'
seqlevelsStyle(ns_spe_loops_left_anchor) = 'ucsc'
seqlevelsStyle(ns_spe_loops_right_anchor) = 'ucsc'
seqlevelsStyle(en_com_loops_left_anchor) = 'ucsc'
seqlevelsStyle(en_com_loops_right_anchor) = 'ucsc'
seqlevelsStyle(es_spe_loops_gr) = 'ucsc'
seqlevelsStyle(ns_spe_loops_gr) = 'ucsc'
seqlevelsStyle(es_loops_gr) = 'ucsc'
seqlevelsStyle(ns_loops_gr) = 'ucsc'
seqlevelsStyle(es_spe_loops_domain_gr) = 'ucsc'
seqlevelsStyle(ns_spe_loops_domain_gr) = 'ucsc'
seqlevelsStyle(common_loops_domain_gr) = 'ucsc'
seqlevelsStyle(cm_loops_gr) = 'ucsc'


es_spe_loops_filt = es_spe_loops[-queryHits(findOverlaps(es_spe_loops_gr,c(en_com_loops_left_anchor,en_com_loops_right_anchor))),]
ns_spe_loops_filt = ns_spe_loops[-queryHits(findOverlaps(ns_spe_loops_gr,c(en_com_loops_left_anchor,en_com_loops_right_anchor))),]
es_spe_loops_filt_gr = getGR(es_spe_loops_filt$X.chr1,es_spe_loops_filt$x1,es_spe_loops_filt$y2,0)
ns_spe_loops_filt_gr = getGR(ns_spe_loops_filt$X.chr1,ns_spe_loops_filt$x1,ns_spe_loops_filt$y2,0)
```

# Analysis of transcriptome with and without CTCF (ES and NS cells)

Check differentiation of ES 46 and Nora ES cells

```
rna_46C = data.frame(es1=read.delim(paste0(rnaseq_directory,'gene_counts_FEB_2022.txt'),skip=1)[,13],
                     es2=read.delim(paste0(rnaseq_directory,'gene_counts_FEB_2022.txt'),skip=1)[,14],
                     ns1=read.delim(paste0(rnaseq_directory,'gene_counts_FEB_2022.txt'),skip=1)[,8],
                     ns2=read.delim(paste0(rnaseq_directory,'gene_counts_FEB_2022.txt'),skip=1)[,10],
                     row.names=read.delim(paste0(rnaseq_directory,'gene_counts_FEB_2022.txt'),skip=1)[,1])
coldata46c = data.frame(cell_type=c('ES','ES','NS','NS'),genotype='46C',row.names=colnames(rna_46C))

load( paste0(rnaseq_directory,"CTCF_degron_countdata.RData") )
count_df_filt = requested_countdata[,1:14]
coldata = requested_metadata[1:14,]
coldata$culture[9:14]='NS'
coldata$cell_type = paste(coldata$culture,coldata$condition,sep="_")
ddsF = DESeqDataSetFromMatrix(countData = count_df_filt,
                              colData = coldata,
                              design= ~cell_type )
```

```
## Warning in DESeqDataSet(se, design = design, ignoreRank): some variables in
## design formula are characters, converting to factors
```

```
ddsF = DESeq(ddsF)
```

```
## estimating size factors
```

```
## estimating dispersions
```

```
## gene-wise dispersion estimates
```

```
## mean-dispersion relationship
```

```
## final dispersion estimates
```

```
## fitting model and testing
```

```
ddsFdf = counts(ddsF,normalized=TRUE )
```

## ES–>NS validation

```
Diff_Nora__DDS = DESeqDataSetFromMatrix(countData = count_df_filt[,c(3,4,7,8,10,11,14)],
                                        colData = coldata[c(3,4,7,8,10,11,14),],
                                        design= ~0+culture )
```

```
## Warning in DESeqDataSet(se, design = design, ignoreRank): some variables in
## design formula are characters, converting to factors
```

```
es2i_ns_comp = results(DESeq(Diff_Nora__DDS), contrast=c("culture", "2i", "NS") )
```

```
## estimating size factors
```

```
## estimating dispersions
```

```
## gene-wise dispersion estimates
```

```
## mean-dispersion relationship
```

```
## final dispersion estimates
```

```
## fitting model and testing
```

```
es2i_ns_comp$padj_squished=es2i_ns_comp$padj
es2i_ns_comp$padj_squished[es2i_ns_comp$padj_squished<10^(-20)]=10^(-20)

par(pty="s")
plot(x=es2i_ns_comp$log2FoldChange,y=-log10(es2i_ns_comp$padj_squished),
     col=ifelse(es2i_ns_comp$padj<0.01,"green4","gray"),
     ylim=c(0,20),pch=19,cex=0.5)
axis(1,lwd=2)
axis(2,lwd=2)
box(col="black",lwd=2)
```

```
df = data.frame(LFC=es2i_ns_comp$log2FoldChange,log10pval=-log10(es2i_ns_comp$padj_squished))
write.table(df,file=paste0(source_data_directory,"volcano_differentiationNcells.txt"),row.names = FALSE, sep="\t",quote=FALSE)
```

```
Diff_Nora__DDS = DESeqDataSetFromMatrix(countData = count_df_filt[,c(3,4,7,8,10,11,14)],
                                        colData = coldata[c(3,4,7,8,10,11,14),],
                                        design= ~0+culture )
```

```
## Warning in DESeqDataSet(se, design = design, ignoreRank): some variables in
## design formula are characters, converting to factors
```

```
es2i_ns_comp_counts = counts(DESeq(Diff_Nora__DDS),normalized=TRUE )
```

```
## estimating size factors
```

```
## estimating dispersions
```

```
## gene-wise dispersion estimates
```

```
## mean-dispersion relationship
```

```
## final dispersion estimates
```

```
## fitting model and testing
```

```
es2i_ns_comp = es2i_ns_comp[!is.na(es2i_ns_comp$padj),]
thisColV = rep("gray",nrow(es2i_ns_comp_counts))
thisColV[rownames(es2i_ns_comp_counts) %in% rownames(es2i_ns_comp[es2i_ns_comp$padj<0.01 & es2i_ns_comp$log2FoldChange>0,])] = "red"
thisColV[rownames(es2i_ns_comp_counts) %in% rownames(es2i_ns_comp[es2i_ns_comp$padj<0.01 & es2i_ns_comp$log2FoldChange<0,])] = "blue"

par(pty="s",bty="O")
plot(x=log2(0.5+rowMeans(es2i_ns_comp_counts[,1:4])),
     y=log2(0.5+rowMeans(es2i_ns_comp_counts[,5:7])),
     col=thisColV,xlab="ES", ylab="NS",
     ylim=c(-2,20),xlim=c(-2,20),pch=19,cex=0.25)
axis(1,lwd=2)
axis(2,lwd=2)
box(col="black",lwd=2)
```

```
df = data.frame(ES=log2(0.5+rowMeans(es2i_ns_comp_counts[,1:4])),NS=log2(0.5+rowMeans(es2i_ns_comp_counts[,5:7])))
write.table(df,file=paste0(source_data_directory,"scatterplot_Differentiation_Ncells.txt"),row.names = FALSE, sep="\t",quote=FALSE)
```

## CTCF targets cell type specific genes

```
LFC_thr=0;pval_thr=0.1
ti_DDS = DESeqDataSetFromMatrix(countData = count_df_filt[,1:8],
                                colData = coldata[1:8,],
                                design= ~cell_type )
```

```
## Warning in DESeqDataSet(se, design = design, ignoreRank): some variables in
## design formula are characters, converting to factors
```

```
ti_DDS=DESeq(ti_DDS)
```

```
## estimating size factors
```

```
## estimating dispersions
```

```
## gene-wise dispersion estimates
```

```
## mean-dispersion relationship
```

```
## final dispersion estimates
```

```
## fitting model and testing
```

```
ti = results(ti_DDS, contrast=c("cell_type", "2i_WT", "2i_IAA") )


ns_DDS = DESeqDataSetFromMatrix(countData = count_df_filt[,9:14],
                                colData = coldata[9:14,],
                                design= ~0+cell_type )
```

```
## Warning in DESeqDataSet(se, design = design, ignoreRank): some variables in
## design formula are characters, converting to factors
```

```
ns_DDS = DESeq(ns_DDS)
```

```
## estimating size factors
```

```
## estimating dispersions
```

```
## gene-wise dispersion estimates
```

```
## mean-dispersion relationship
```

```
## final dispersion estimates
```

```
## fitting model and testing
```

```
ns = results(ns_DDS, contrast=c("cell_type", "NS_WT", "NS_IAA") )

es_ns_IAA_merged = merge( as.data.frame(ti), as.data.frame(ns), by='row.names')


ti = ti[!is.na(ti$padj),]
ns = ns[!is.na(ns$padj),]

TI_sig_genes = ( ti[ti$padj<pval_thr & abs(ti$log2FoldChange)>LFC_thr,] )
TI_sig_genes_up = rownames( ti[ti$padj<pval_thr & ti$log2FoldChange<(-1*LFC_thr),] )
TI_sig_genes_dn = rownames( ti[ti$padj<pval_thr & ti$log2FoldChange>LFC_thr,] )
TI_common = rownames( ti[ti$padj>0.1 & abs(ti$log2FoldChange)<log2(1.25),] )

NS_sig_genes = ( ns[ns$padj<pval_thr & abs(ns$log2FoldChange)>LFC_thr,] )
NS_sig_genes_up = rownames( ns[ns$padj<pval_thr & ns$log2FoldChange<(-1 *LFC_thr),] )
NS_sig_genes_dn = rownames( ns[ns$padj<pval_thr & ns$log2FoldChange>LFC_thr,] )
NS_common = rownames( ns[ns$padj>0.1 & abs(ns$log2FoldChange)<log2(1.25),] )

write.table( TI_sig_genes_up, file=paste0(outputs_directory,'TI_sig_genes_up.txt'),
             quote=FALSE, row.names=FALSE, col.names=FALSE, sep='\t' )
write.table( TI_sig_genes_dn, file=paste0(outputs_directory,'TI_sig_genes_dn.txt'),
             quote=FALSE, row.names=FALSE, col.names=FALSE, sep='\t' )
write.table( NS_sig_genes_up, file=paste0(outputs_directory,'NS_sig_genes_up.txt'),
             quote=FALSE, row.names=FALSE, col.names=FALSE, sep='\t' )
write.table( NS_sig_genes_dn, file=paste0(outputs_directory,'NS_sig_genes_dn.txt'),
             quote=FALSE, row.names=FALSE, col.names=FALSE, sep='\t' )

length(unique(c(TI_sig_genes_up,TI_sig_genes_dn,NS_sig_genes_up,NS_sig_genes_dn)))
```

```
## [1] 1250
```

Volcano plots for the extended figure

```
TI = data.frame(Untreated=rowMeans(ddsFdf[,c('ES_2i_1','ES_2i_2','ES_2i_3','ES_2i_4')]),
                IAA = rowMeans(ddsFdf[,c('ES_2i_IAA_1','ES_2i_IAA_2','ES_2i_IAA_3','ES_2i_IAA_4')]),
                stringsAsFactors=FALSE )

par(mar=c(5,5,3,1))
plot(ti$log2FoldChange,-log10(ti$pvalue), 
     col=ifelse(abs(ti$log2FoldChange)>0 & ti$padj<pval_thr,'red3','gray'), 
     pch=19, cex=0.25,
     xlab=expression('log'[2]*'(Untr/IAA)'),
     ylab=expression('-log'[10]*'(P-val)'),
     ylim=c(0,30),xlim=c(-3,3),
     main='ES 2i untr. vs. IAA')
axis(1,lwd=3)
axis(2,lwd=3)
box(lwd=3,col='black')
```

```
df = data.frame(LFC=ti$log2FoldChange,log10pval=-log10(ti$pvalue),col=ifelse(abs(ti$log2FoldChange)>0 & ti$padj<pval_thr,'red3','gray'))
write.table(df,file=paste0(source_data_directory,"volcano_ES_IAA.txt"),row.names = FALSE, sep="\t",quote=FALSE)
```

```
NS = data.frame(Untreated=rowMeans(ddsFdf[,c('NS_FBS_3','NS_FBS_5','NS_FBS_4')]),
                IAA = rowMeans(ddsFdf[,c('NS_FBS_IAA_3','NS_FBS_IAA_5','NS_FBS_IAA_4')]),
                stringsAsFactors=FALSE )

par(mar=c(5,5,3,1))
plot(ns$log2FoldChange,-log10(ns$pvalue), 
     col=ifelse(abs(ns$log2FoldChange)>0 & ns$padj<pval_thr,'red3','gray'), 
     pch=19, cex=0.25,
     ylim=c(0,30),xlim=c(-3,3),
     xlab=expression('log'[2]*'(Untr/IAA)'),
     ylab=expression('-log'[10]*'(P-val)'),
     main='NS')
axis(1,lwd=3)
axis(2,lwd=3)
box(lwd=3,col='black')
```

```
df = data.frame(LFC=ns$log2FoldChange,log10pval=-log10(ns$pvalue),col=ifelse(abs(ns$log2FoldChange)>0 & ns$padj<pval_thr,'red3','gray'))
write.table(df,file=paste0(source_data_directory,"volcano_NS_IAA.txt"),row.names = FALSE, sep="\t",quote=FALSE)
```

More up-regulated genes in the NS cells

```
pval_thr_plot=0.1
x = ti[ti$padj<pval_thr_plot,]
y = ns[ns$padj<pval_thr_plot,]
m=rbind(ES_2i=table( x$log2FoldChange>0 ),
        NS=table( y$log2FoldChange>0 ) )
m
```

```
##       FALSE TRUE
## ES_2i   357  418
## NS      358  198
```

```
fisher.test(m)
```

```
## 
##  Fisher's Exact Test for Count Data
## 
## data:  m
## p-value = 4.223e-11
## alternative hypothesis: true odds ratio is not equal to 1
## 95 percent confidence interval:
##  0.3751452 0.5944608
## sample estimates:
## odds ratio 
##  0.4726291
```

```
par(mar=c(5,10,5,10),mfrow=c(1,1))
barplot(100*(m/rowSums(m)), 
        beside=T, ylim=c(0,80),
        col=c('red','blue'), ylab='%',
        names=c('Up','Down'),lwd=1.5)
fisher.test(matrix(c(49,51,65,35),2,2))
```

```
## 
##  Fisher's Exact Test for Count Data
## 
## data:  matrix(c(49, 51, 65, 35), 2, 2)
## p-value = 0.03188
## alternative hypothesis: true odds ratio is not equal to 1
## 95 percent confidence interval:
##  0.2815690 0.9484736
## sample estimates:
## odds ratio 
##   0.519077
```

```
axis(1,lwd=3,at=c(2,5),c('Up','Down'))
axis(2,lwd=3)
```

Direction of change

```
es_ns_IAA_merged_filt = es_ns_IAA_merged[! is.na(es_ns_IAA_merged$padj.x),]
es_ns_IAA_merged_filt = es_ns_IAA_merged_filt[! is.na(es_ns_IAA_merged_filt$padj.y),]
es_ns_IAA_merged_filt = es_ns_IAA_merged_filt[rowSums(es_ns_IAA_merged_filt[,c('padj.x','padj.y')]<0.1)>0,]
es_ns_IAA_merged_filt$geneName = genemap_unique$geneName[match( es_ns_IAA_merged_filt$Row.names,genemap_unique$gene_id ) ]

es_ns_IAA_merged_filt$col = 'black'
es_ns_IAA_merged_filt$col[es_ns_IAA_merged_filt$padj.x<0.1 & es_ns_IAA_merged_filt$padj.y<0.1] = 'green4'
es_ns_IAA_merged_filt$col[es_ns_IAA_merged_filt$padj.x<0.1 & es_ns_IAA_merged_filt$padj.y>0.1] = 'red'
es_ns_IAA_merged_filt$col[es_ns_IAA_merged_filt$padj.x>0.1 & es_ns_IAA_merged_filt$padj.y<0.1] = 'blue'


es_ns_IAA_merged_filt$size = 0.5
es_ns_IAA_merged_filt$size[es_ns_IAA_merged_filt$padj.x<0.1 & es_ns_IAA_merged_filt$padj.y<0.1] = 0.5

par(mar=c(5,5,3,1))
plot(-1*es_ns_IAA_merged_filt$log2FoldChange.x,
     -1*es_ns_IAA_merged_filt$log2FoldChange.y, 
     col=es_ns_IAA_merged_filt$col, 
     pch=19, cex=es_ns_IAA_merged_filt$size,
     ylim=c(-4,4),xlim=c(-4,4),
     xlab=expression('ES [log'[2]*'(IAA/Untreated)]'),
     ylab=expression('NS [log'[2]*'(IAA/Untreated)]'),
     main='Without versus with CTCF')
abline( v=0,col='gray',lwd=3)
abline( h=0,col='gray',lwd=3)
axis(1,lwd=3)
axis(2,lwd=3)
box(col='black',lwd=3)

points( es_ns_IAA_merged_filt$log2FoldChange.x[es_ns_IAA_merged_filt$Row.names == 'ENSMUSG00000024268'],
        es_ns_IAA_merged_filt$log2FoldChange.y[es_ns_IAA_merged_filt$Row.names == 'ENSMUSG00000024268'], pch=19, cex=0.5, col='black')
```

```
cor.test(es_ns_IAA_merged_filt$log2FoldChange.x, es_ns_IAA_merged_filt$log2FoldChange.y)
```

```
## 
##  Pearson's product-moment correlation
## 
## data:  es_ns_IAA_merged_filt$log2FoldChange.x and es_ns_IAA_merged_filt$log2FoldChange.y
## t = 13.692, df = 979, p-value < 2.2e-16
## alternative hypothesis: true correlation is not equal to 0
## 95 percent confidence interval:
##  0.3469996 0.4521298
## sample estimates:
##       cor 
## 0.4008836
```

```
es_ns_IAA_merged_filt_genes = es_ns_IAA_merged_filt[!is.na(es_ns_IAA_merged_filt$geneName),]
```

```
df = data.frame(ES=-1*es_ns_IAA_merged_filt$log2FoldChange.x,NS=-1*es_ns_IAA_merged_filt$log2FoldChange.y,col=es_ns_IAA_merged_filt$col)
write.table(df,file=paste0(source_data_directory,"scatterplot_IAAimpact_Ncells.txt"),row.names = FALSE, sep="\t",quote=FALSE)
```

## Aldh1a3 expression plot

Plots for Figure 10

```
tp=buildDfForGGBARPLOT( ddsFdf,as.data.frame(coldata),
                     "cell_type",
                     "Sox9",
                     genemap_unique )
tp$annot = factor(tp$annot,levels = c("NS_WT","NS_IAA","2i_WT","2i_IAA"))
ggbarplot(tp, x = "annot", y = "norm_counts", 
          color="annot",
          add = c("mean_se", "jitter"), size=1.25,
          palette=c('blue','blue4','red','coral3'),
          title="Sox9") + theme( axis.line = element_line(colour = 'black', size = 0.75))
```

```
## Warning: The `size` argument of `element_line()` is deprecated as of ggplot2 3.4.0.
## ℹ Please use the `linewidth` argument instead.
## This warning is displayed once every 8 hours.
## Call `lifecycle::last_lifecycle_warnings()` to see where this warning was
## generated.
```

```
tp
```

```
##    norm_counts  annot
## 1     5.448699 2i_IAA
## 2     1.689030 2i_IAA
## 3     3.136609  2i_WT
## 4     4.230981  2i_WT
## 5     7.634598 2i_IAA
## 6     5.358430 2i_IAA
## 7     4.846634  2i_WT
## 8     5.989234  2i_WT
## 9   978.291257 NS_IAA
## 10 1530.401815  NS_WT
## 11 1868.185338  NS_WT
## 12  890.618161 NS_IAA
## 13  889.752300 NS_IAA
## 14 1371.565757  NS_WT
```

```
tp=buildDfForGGBARPLOT( ddsFdf,as.data.frame(coldata),
                     "cell_type",
                     "Ngfr",
                     genemap_unique )
tp$annot = factor(tp$annot,levels = c("NS_WT","NS_IAA","2i_WT","2i_IAA"))
ggbarplot(tp, x = "annot", y = "norm_counts", 
          color="annot",
          add = c("mean_se", "jitter"), size=1.25,
          palette=c('blue','blue4','red','coral3'),
          title="Ngfr") + theme( axis.line = element_line(colour = 'black', size = 0.75)) + ylim(c(0,125))
```

```
## Warning: Removed 8 rows containing non-finite outside the scale range
## (`stat_summary()`).
```

```
## Warning: Removed 2 rows containing missing values or values outside the scale range
## (`geom_bar()`).
```

```
## Warning: Removed 8 rows containing missing values or values outside the scale range
## (`geom_point()`).
```

```
tp
```

```
##    norm_counts  annot
## 1    530.34002 2i_IAA
## 2    913.76539 2i_IAA
## 3    564.58968  2i_WT
## 4    829.27229  2i_WT
## 5    329.67584 2i_IAA
## 6    306.62127 2i_IAA
## 7    428.44241  2i_WT
## 8    387.05422  2i_WT
## 9    101.76855 NS_IAA
## 10    47.31081  NS_WT
## 11    44.81172  NS_WT
## 12   110.09226 NS_IAA
## 13    94.72400 NS_IAA
## 14    55.29920  NS_WT
```

```
tp=buildDfForGGBARPLOT( ddsFdf,as.data.frame(coldata),
                     "cell_type",
                     "Camk2a",
                     genemap_unique )
tp$annot = factor(tp$annot,levels = c("NS_WT","NS_IAA","2i_WT","2i_IAA"))
ggbarplot(tp, x = "annot", y = "norm_counts", 
          color="annot",
          add = c("mean_se", "jitter"), size=1.25,
          palette=c('blue','blue4','red','coral3'),
          title="Camk2a") + theme( axis.line = element_line(colour = 'black', size = 0.75)) + ylim(c(0,600))
```

```
tp
```

```
##    norm_counts  annot
## 1     187.0720 2i_IAA
## 2     167.2140 2i_IAA
## 3     196.0381  2i_WT
## 4     205.2026  2i_WT
## 5     263.7407 2i_IAA
## 6     270.8984 2i_IAA
## 7     268.5035  2i_WT
## 8     243.3126  2i_WT
## 9     591.7349 NS_IAA
## 10    165.2450  NS_WT
## 11    285.8679  NS_WT
## 12    338.0397 NS_IAA
## 13    595.7813 NS_IAA
## 14    344.1648  NS_WT
```

## Aldh1a3 CTCF-BS KOs

```
library(dplyr) 
library(ggplot2)
library(ggpubr)
theme_set(theme_bw())

mm = read.delim(paste0(data_directory,"Aldh1a3_ES-NS qPCR.txt"),header=TRUE)
mm = mm[-1,]
mm$Genotype=factor(mm$Genotype,levels=c("Wild_type","KO_1","KO_2","KO_3"))

mmES = mm[mm$Cell_type=="ES",]
m = ggplot(mmES, aes(x = Genotype, y = Expression))

m + geom_jitter(
  aes(shape = Genotype, color = Genotype), 
  position = position_jitter(0.2),
  size = 5 ) +
  stat_summary(
    aes(color = Genotype),
    fun.data="mean_sdl",  fun.args = list(mult=1), 
    geom = "pointrange",  size = 0.7) + scale_color_manual(values =  c("red","coral3", "coral3", "coral3")) + ylim(0,1) + theme( axis.line = element_line(colour = "black", 
                      linewidth = 1, linetype = "solid"))
```

```
mmNS = mm[mm$Cell_type=="NS",]
m = ggplot(mmNS, aes(x = Genotype, y = Expression))

m + geom_jitter(
  aes(shape = Genotype, color = Genotype), 
  position = position_jitter(0.1),
  size = 5 ) +
  stat_summary(
    aes(color = Genotype),
    fun.data="mean_sdl",  fun.args = list(mult=1), 
    geom = "pointrange",  size = 0.7 ) + scale_color_manual(values =  c("blue","steelblue3", "steelblue3", "steelblue3")) + ylim(0,0.5) + theme( axis.line = element_line(colour = "black", 
                      linewidth = 1, linetype = "solid"))
```

```
t.test( mmNS$Expression[mmNS$Genotype=="Wild_type"],mmNS$Expression[mmNS$Genotype=="KO_1"])
```

```
## 
##  Welch Two Sample t-test
## 
## data:  mmNS$Expression[mmNS$Genotype == "Wild_type"] and mmNS$Expression[mmNS$Genotype == "KO_1"]
## t = -4.7588, df = 3.6289, p-value = 0.0113
## alternative hypothesis: true difference in means is not equal to 0
## 95 percent confidence interval:
##  -0.35052505 -0.08552556
## sample estimates:
##  mean of x  mean of y 
## 0.07367433 0.29169964
```

Check if the differentiation between ES2i and NS worked in the Nora and the 46C cells.

```
marker_genes=c("Prdm14","Nanog","Fgf4","Nodal","Lin28a","Zfp42","Dppa2","Esrrb","Tbx3","Klf4","Pou5f1","Phc1","Tet1","Dll1","Pax6","Notch1","Rxra","Hes6","Wnt5a","Fzd2","Fzd1","Nes","Bmi1","Hes5","Ascl1","Olig1","Olig2")

ens2gene = data.frame(gtf$gene_name,gtf$gene_id)
ens2gene = ens2gene[!duplicated(ens2gene$gtf.gene_id),]
marker_genes = data.frame(gene_names=marker_genes,
                          gene_id = ens2gene$gtf.gene_id[match(marker_genes,ens2gene$gtf.gene_name)])

marker_genes$lfc = -1*es2i_ns_comp$log2FoldChange[match(marker_genes$gene_id,rownames(es2i_ns_comp))]
marker_genes$padj = es2i_ns_comp$padj[match(marker_genes$gene_id,rownames(es2i_ns_comp))]
marker_genes = marker_genes[order(marker_genes$lfc,decreasing=FALSE),]

barplot(marker_genes$lfc,col=ifelse(marker_genes$lfc<0,"red","blue"),
        names=marker_genes$gene_names,las=2,ylim=c(-20,20),cex.axis = 1.5)
```

```
df = data.frame(LFC=marker_genes$lfc,col=ifelse(marker_genes$lfc<0,"red","blue"))
write.table(df,file=paste0(source_data_directory,"barplot_ES__NS_differentiation.txt"))
```

# Expression of up and down regulated genes

```
rnL = read.delim(paste0(rnaseq_directory,'gene_counts_FEB_2022.txt'),skip=1)[,c(1,6)]
requested_countdata_tpm = data.frame( ES = rowSums( requested_countdata[,c(3,4,7,8)] ),
                                      NS = rowSums( requested_countdata[,c(10,11,14)] ),
                                      Length = rnL$Length[match(rownames(requested_countdata),rnL$Geneid)] )
requested_countdata_tpm = GetTPM(requested_countdata_tpm,1:2,rownames(requested_countdata_tpm))

par(mfrow=c(1,2),bty='n')
boxplot( requested_countdata_tpm[rownames(requested_countdata_tpm) %in% TI_sig_genes_up, "ES"],
         requested_countdata_tpm[rownames(requested_countdata_tpm) %in% TI_sig_genes_dn, "ES"],
         outline=FALSE,col='white', ylab="Expression (TPM)",
         border=c('red','red4'),lwd=3,ylim=c(0,100))
axis(1,lwd=3,at=c(1,2),c("Up","Down"))
axis(2,lwd=3)
boxplot( requested_countdata_tpm[rownames(requested_countdata_tpm) %in% NS_sig_genes_up, "NS"],
         requested_countdata_tpm[rownames(requested_countdata_tpm) %in% NS_sig_genes_dn, "NS"],
         outline=FALSE,col='white', border=c('blue','steelblue3'),
         ylab="Expression (TPM)",
         lwd=3,ylim=c(0,100))
axis(1,lwd=3,at=c(1,2),c("Up","Down"))
axis(2,lwd=3)
```

```
t.test(requested_countdata_tpm[rownames(requested_countdata_tpm) %in% TI_sig_genes_up, "ES"],
       requested_countdata_tpm[rownames(requested_countdata_tpm) %in% TI_sig_genes_dn, "ES"])
```

```
## 
##  Welch Two Sample t-test
## 
## data:  requested_countdata_tpm[rownames(requested_countdata_tpm) %in% TI_sig_genes_up, "ES"] and requested_countdata_tpm[rownames(requested_countdata_tpm) %in% TI_sig_genes_dn, "ES"]
## t = -5.2704, df = 441.46, p-value = 2.134e-07
## alternative hypothesis: true difference in means is not equal to 0
## 95 percent confidence interval:
##  -102.59209  -46.86077
## sample estimates:
## mean of x mean of y 
##  12.29998  87.02641
```

```
t.test(requested_countdata_tpm[rownames(requested_countdata_tpm) %in% NS_sig_genes_up, "NS"],
         requested_countdata_tpm[rownames(requested_countdata_tpm) %in% NS_sig_genes_dn, "NS"])
```

```
## 
##  Welch Two Sample t-test
## 
## data:  requested_countdata_tpm[rownames(requested_countdata_tpm) %in% NS_sig_genes_up, "NS"] and requested_countdata_tpm[rownames(requested_countdata_tpm) %in% NS_sig_genes_dn, "NS"]
## t = -2.5177, df = 210.1, p-value = 0.01256
## alternative hypothesis: true difference in means is not equal to 0
## 95 percent confidence interval:
##  -83.12087 -10.11752
## sample estimates:
## mean of x mean of y 
##  21.75334  68.37254
```

```
theNtimes = max(c(length(TI_sig_genes_up),length(TI_sig_genes_dn),length(NS_sig_genes_up),length(NS_sig_genes_dn)))
df = data.frame( ES_up_by_IAA = rep(NA,theNtimes), ES_dn_by_IAA = rep(NA,theNtimes),
                 NS_up_by_IAA = rep(NA,theNtimes), NS_dn_by_IAA = rep(NA,theNtimes) )
df$ES_up_by_IAA[1:length(TI_sig_genes_up)] = requested_countdata_tpm[rownames(requested_countdata_tpm) %in% TI_sig_genes_up, "ES"]
df$ES_dn_by_IAA[1:length(TI_sig_genes_dn)] = requested_countdata_tpm[rownames(requested_countdata_tpm) %in% TI_sig_genes_dn, "ES"]
df$NS_up_by_IAA[1:length(NS_sig_genes_up)] = requested_countdata_tpm[rownames(requested_countdata_tpm) %in% NS_sig_genes_up, "ES"]
df$NS_dn_by_IAA[1:length(NS_sig_genes_dn)] = requested_countdata_tpm[rownames(requested_countdata_tpm) %in% NS_sig_genes_dn, "ES"]
write.table(df,file=paste0(source_data_directory,"boxplots_TPM.txt"),row.names = FALSE, sep="\t",quote=FALSE)
```

# Chromatin signature with and without CTCF - ATAC-seq and ChIP-seq data

ATAC peaks +/- CTCF

```
ti_atac_peaks_Nora_unt = readBed_filterChroms(paste0(chipseq_directory,'ES_2i_PLNOV_merged_filtered.bam_peaks.narrowPeak'),
                                     chroms=paste0('chr',c(1:19,'X','Y')), 7)
ti_atac_peaks_Nora_iaa = readBed_filterChroms(paste0(chipseq_directory,'ES_2i_IAA_PLNOV_merged_filtered.bam_peaks.narrowPeak'),
                                     chroms=paste0('chr',c(1:19,'X','Y')), 7)

ns_atac_peaks_Nora_unt = readBed_filterChroms(paste0(chipseq_directory,'NPC_PLDEC_merged_filtered.bam_peaks.narrowPeak'),
                                     chroms=paste0('chr',c(1:19,'X','Y')), 7)
ns_atac_peaks_Nora_iaa = readBed_filterChroms(paste0(chipseq_directory,'NPC_IAA_PLDEC_merged_filtered.bam_peaks.narrowPeak'),
                                     chroms=paste0('chr',c(1:19,'X','Y')), 7)

ti_atac_peaks_Nora_unt_summit = readNarrowPeak2getSummit(paste0(chipseq_directory,'ES_2i_PLNOV_merged_filtered.bam_peaks.narrowPeak'),
                                     chroms=paste0('chr',c(1:19,'X','Y')), 5)
ti_atac_peaks_Nora_iaa_summit = readNarrowPeak2getSummit(paste0(chipseq_directory,'ES_2i_IAA_PLNOV_merged_filtered.bam_peaks.narrowPeak'),
                                     chroms=paste0('chr',c(1:19,'X','Y')), 5)

ns_atac_peaks_Nora_unt_summit = readNarrowPeak2getSummit(paste0(chipseq_directory,'NPC_PLDEC_merged_filtered.bam_peaks.narrowPeak'),
                                     chroms=paste0('chr',c(1:19,'X','Y')), 5)
ns_atac_peaks_Nora_iaa_summit = readNarrowPeak2getSummit(paste0(chipseq_directory,'NPC_IAA_PLDEC_merged_filtered.bam_peaks.narrowPeak'),
                                     chroms=paste0('chr',c(1:19,'X','Y')), 5)
```

```
overlaps=findOverlaps(ti_atac_peaks_Nora_unt,ti_atac_peaks_Nora_iaa)
draw.pairwise.venn(area1=queryLength(overlaps),
                   area2=subjectLength(overlaps),
                   cross.area=length(overlaps),
                   category=c('',''), col=c('red','coral4'),
                   fill=rep('white',2),cat.cex=1.2,lwd=8)
```

```
## (polygon[GRID.polygon.374], polygon[GRID.polygon.375], polygon[GRID.polygon.376], polygon[GRID.polygon.377], text[GRID.text.378], text[GRID.text.379], text[GRID.text.380], text[GRID.text.381], text[GRID.text.382])
```

```
overlaps=findOverlaps(ns_atac_peaks_Nora_unt,ns_atac_peaks_Nora_iaa)
draw.pairwise.venn(area1=queryLength(overlaps),
                   area2=subjectLength(overlaps),
                   cross.area=length(overlaps),
                   category=c('',''), col=c('blue','blue4'),
                   fill=rep('white',2),cat.cex=1.2,lwd=8)
```

```
## (polygon[GRID.polygon.383], polygon[GRID.polygon.384], polygon[GRID.polygon.385], polygon[GRID.polygon.386], text[GRID.text.387], text[GRID.text.388], text[GRID.text.389], text[GRID.text.390], text[GRID.text.391])
```

H3K27ac peaks

```
ti_k27ac_peaks_Nora_unt = readBed_filterChroms(paste0(chipseq_directory,'ChIP_Seq_H3K27ac_05-22_MusMus_ESC_AID_KI_CTCF-AID-GFP_OsTIR_TIGRE_Nora_2i_Rep_1_peaks.narrowPeak'),
                                     chroms=paste0('chr',c(1:19,'X','Y')), 7)
ti_k27ac_peaks_Nora_iaa = readBed_filterChroms(paste0(chipseq_directory,'ChIP_Seq_H3K27ac_05-22_MusMus_ESC_AID_KI_CTCF-AID-GFP_OsTIR_TIGRE_Nora_2i_IAA_Rep_1_peaks.narrowPeak'),
                                     chroms=paste0('chr',c(1:19,'X','Y')), 7)

ns_k27ac_peaks_Nora_unt = readBed_filterChroms(paste0(chipseq_directory,'ChIP_Seq_H3K27ac_05-22_MusMus_es-NPC_AID_KI_CTCF-AID-GFP_OsTIR_TIGRE_Nora_Rep_1_peaks.narrowPeak'),
                                     chroms=paste0('chr',c(1:19,'X','Y')), 7)
ns_k27ac_peaks_Nora_iaa = readBed_filterChroms(paste0(chipseq_directory,'ChIP_Seq_H3K27ac_05-22_MusMus_es-NPC_AID_KI_CTCF-AID-GFP_OsTIR_TIGRE_Nora_IAA_Rep_1_peaks.narrowPeak'),
                                     chroms=paste0('chr',c(1:19,'X','Y')), 7)
```

```
overlaps=findOverlaps(ti_k27ac_peaks_Nora_unt,ti_k27ac_peaks_Nora_iaa)
draw.pairwise.venn(area1=queryLength(overlaps),
                   area2=subjectLength(overlaps),
                   cross.area=length(overlaps),
                   category=c('',''), col=c('red','coral4'),
                   fill=rep('white',2),cat.cex=1.2,lwd=8)
```

```
## (polygon[GRID.polygon.392], polygon[GRID.polygon.393], polygon[GRID.polygon.394], polygon[GRID.polygon.395], text[GRID.text.396], text[GRID.text.397], text[GRID.text.398], text[GRID.text.399], text[GRID.text.400])
```

```
overlaps=findOverlaps(ns_k27ac_peaks_Nora_unt,ns_k27ac_peaks_Nora_iaa)
draw.pairwise.venn(area1=queryLength(overlaps),
                   area2=subjectLength(overlaps),
                   cross.area=length(overlaps),
                   category=c('',''), col=c('blue','blue4'),
                   fill=rep('white',2),cat.cex=1.2,lwd=8)
```

```
## (polygon[GRID.polygon.401], polygon[GRID.polygon.402], polygon[GRID.polygon.403], polygon[GRID.polygon.404], text[GRID.text.405], text[GRID.text.406], text[GRID.text.407], text[GRID.text.408], text[GRID.text.409])
```

## Peak number changes and deregulation upon CTCF loss

```
es_up_proms = GenomicRanges::resize(promoters_tss_gr[promoters_tss_gr$gene_id %in% TI_sig_genes_up],500000,fix='center')
es_dn_proms = GenomicRanges::resize(promoters_tss_gr[promoters_tss_gr$gene_id %in% TI_sig_genes_dn],500000,fix='center')

ns_up_proms = GenomicRanges::resize(promoters_tss_gr[promoters_tss_gr$gene_id %in% NS_sig_genes_up],500000,fix='center')
ns_dn_proms = GenomicRanges::resize(promoters_tss_gr[promoters_tss_gr$gene_id %in% NS_sig_genes_dn],500000,fix='center')

par(mfrow=c(1,4),mar=c(6,5,1,1),pty="m")
es_up_promc = data.frame(unt=countOverlaps(es_up_proms,ti_k27ac_peaks_Nora_unt),
                         iaa=countOverlaps(es_up_proms,ti_k27ac_peaks_Nora_iaa))
es_dn_promc = data.frame(unt=countOverlaps(es_dn_proms,ti_k27ac_peaks_Nora_unt),
                         iaa=countOverlaps(es_dn_proms,ti_k27ac_peaks_Nora_iaa))
boxplot( es_dn_promc$iaa-es_dn_promc$unt, 
         es_up_promc$iaa-es_up_promc$unt, 
         border=c("red4","coral4"),
         outline=FALSE, col="white",names=c("Down","Up"),
         ylim=c(-10,10),lwd=2, ylab="IAA-veh")
write.table(es_up_promc,file=paste0(source_data_directory,"es_up_promc.txt"),quote=FALSE,row.names = FALSE, sep="\t")
write.table(es_dn_promc,file=paste0(source_data_directory,"es_dn_promc.txt"),quote=FALSE,row.names = FALSE, sep="\t")


es_up_promc = data.frame(unt=countOverlaps(es_up_proms,ti_atac_peaks_Nora_unt),
                         iaa=countOverlaps(es_up_proms,ti_atac_peaks_Nora_iaa))
es_dn_promc = data.frame(unt=countOverlaps(es_dn_proms,ti_atac_peaks_Nora_unt),
                         iaa=countOverlaps(es_dn_proms,ti_atac_peaks_Nora_iaa))

boxplot( es_dn_promc$iaa-es_dn_promc$unt, 
         es_up_promc$iaa-es_up_promc$unt, 
         border=c("red4","coral4"),
         outline=FALSE, col="white", names=c("Down","Up"),
         ylim=c(-10,10), lwd=2, ylab="IAA-veh" )
write.table(es_up_promc,file=paste0(source_data_directory,"es_up_promc.txt"),quote=FALSE,row.names = FALSE, sep="\t")
write.table(es_dn_promc,file=paste0(source_data_directory,"es_dn_promc.txt"),quote=FALSE,row.names = FALSE, sep="\t")

ns_dn_promc = data.frame(unt=countOverlaps(ns_dn_proms,ns_k27ac_peaks_Nora_unt),
                         iaa=countOverlaps(ns_dn_proms,ns_k27ac_peaks_Nora_iaa))
ns_up_promc = data.frame(unt=countOverlaps(ns_up_proms,ns_k27ac_peaks_Nora_unt),
                         iaa=countOverlaps(ns_up_proms,ns_k27ac_peaks_Nora_iaa))
boxplot( ns_dn_promc$unt-ns_dn_promc$iaa, 
         ns_up_promc$unt-ns_up_promc$iaa, 
         border=c("blue4","steelblue3"),
         outline=FALSE, col="white",names=c("Down","Up"),
         ylim=c(-10,10), lwd=2, ylab="veh-IAA" )
write.table(ns_dn_promc,file=paste0(source_data_directory,"ns_dn_promc.txt"),quote=FALSE,row.names = FALSE, sep="\t")
write.table(ns_up_promc,file=paste0(source_data_directory,"ns_up_promc.txt"),quote=FALSE,row.names = FALSE, sep="\t")

ns_dn_promc = data.frame(unt=countOverlaps(ns_dn_proms,ns_atac_peaks_Nora_unt),
                         iaa=countOverlaps(ns_dn_proms,ns_atac_peaks_Nora_iaa))
ns_up_promc = data.frame(unt=countOverlaps(ns_up_proms,ns_atac_peaks_Nora_unt),
                         iaa=countOverlaps(ns_up_proms,ns_atac_peaks_Nora_iaa))
boxplot( ns_dn_promc$unt-ns_dn_promc$iaa, 
         ns_up_promc$unt-ns_up_promc$iaa, 
         border=c("blue4","steelblue3"),
         outline=FALSE, col="white",names=c("Down","Up"),
         ylim=c(-10,10), lwd=2, ylab="veh-IAA" )
```

```
write.table(ns_dn_promc,file=paste0(source_data_directory,"ns_dn_promc.txt"),quote=FALSE,row.names = FALSE, sep="\t")
write.table(ns_up_promc,file=paste0(source_data_directory,"ns_up_promc.txt"),quote=FALSE,row.names = FALSE, sep="\t")
```

CTCF controls - we define peaks in untreated cells and look at CTCF signal in untreated and treated cells

```
tictmm10Nora_peak = readNarrowPeak2getSummit( paste0(chipseq_directory, 'ChIP_Seq_CTCF_07-22_MusMus_ESC_AID_KI_CTCF-AID-GFP_OsTIR_TIGRE_2i_Rep_1_peaks.narrowPeak'),
                                     chroms=paste0('chr',c(1:19,'X','Y')), 5)

nsctmm10Nora_peak = readNarrowPeak2getSummit( paste0(chipseq_directory, 'ChIP_Seq_CTCF_05-22_MusMus_es-NPC_AID_KI_CTCF-AID-GFP_OsTIR_TIGRE_Nora_Rep_1_peaks.narrowPeak'),
                                     chroms=paste0('chr',c(1:19,'X','Y')), 5)

ti_nora_ctcf_untreated_bp = import.bw(paste0(chipseq_directory,'ChIP_Seq_CTCF_07-22_MusMus_ESC_AID_KI_CTCF-AID-GFP_OsTIR_TIGRE_2i_Rep_1_RPGC.bw'))
ti_nora_ctcf_iaa_bp = import.bw(paste0(chipseq_directory,'ChIP_Seq_CTCF_07-22_MusMus_ESC_AID_KI_CTCF-AID-GFP_OsTIR_TIGRE_2i-IAA_Rep_1_RPGC.bw'))
ns_nora_ctcf_untreated_bp = import.bw(paste0(chipseq_directory,'ChIP_Seq_CTCF_05-22_MusMus_es-NPC_AID_KI_CTCF-AID-GFP_OsTIR_TIGRE_Nora_Rep_1_RPGC.bw'))
ns_nora_ctcf_iaa_bp = import.bw(paste0(chipseq_directory,'ChIP_Seq_CTCF_05-22_MusMus_es-NPC_AID_KI_CTCF-AID-GFP_OsTIR_TIGRE_Nora_IAA_Rep_1_RPGC.bw'))

seqlevelsStyle(ti_nora_ctcf_untreated_bp)='ucsc'
seqlevelsStyle(ns_nora_ctcf_untreated_bp)='ucsc'
seqlevelsStyle(ti_nora_ctcf_iaa_bp)='ucsc'
seqlevelsStyle(ns_nora_ctcf_iaa_bp)='ucsc'

Ctcf_nora_summit_ranges_ES = do.call('rbind', lapply( as.list(seq(1:length(tictmm10Nora_peak))), function(i){
  g = tictmm10Nora_peak[i]
  tss = start(g)
  chr = as.character( chrom(g) )
  peak=i
      return( data.frame( chr = rep( (chr), 200),
                  starts = seq( tss-1000, tss+990, length.out=200),
                  ends =  seq( tss-1000, tss+990, length.out=200)+9,
                  peak = rep( peak, 200) ) )
} ) )
Ctcf_nora_summit_ranges_NS = do.call('rbind', lapply( as.list(seq(1:length(nsctmm10Nora_peak))), function(i){
  g = nsctmm10Nora_peak[i]
  tss = start(g)
  chr = as.character( chrom(g) )
  peak=i
      return( data.frame( chr = rep( (chr), 200),
                  starts = seq( tss-1000, tss+990, length.out=200),
                  ends =  seq( tss-1000, tss+990, length.out=200)+9,
                  peak = rep( peak, 200) ) )
} ) )

Ctcf_nora_summit_ranges_ES = GRanges(seqnames = Rle(Ctcf_nora_summit_ranges_ES$chr),
                               ranges = IRanges(as.numeric(Ctcf_nora_summit_ranges_ES$starts),
                                                end = as.numeric(Ctcf_nora_summit_ranges_ES$ends),
                                                names = seq(1, nrow(Ctcf_nora_summit_ranges_ES))),
                               strand = Rle(rep("*", nrow(Ctcf_nora_summit_ranges_ES))),
                               peak = Ctcf_nora_summit_ranges_ES$peak )

Ctcf_nora_summit_ranges_NS = GRanges(seqnames = Rle(Ctcf_nora_summit_ranges_NS$chr),
                               ranges = IRanges(as.numeric(Ctcf_nora_summit_ranges_NS$starts),
                                                end = as.numeric(Ctcf_nora_summit_ranges_NS$ends),
                                                names = seq(1, nrow(Ctcf_nora_summit_ranges_NS))),
                               strand = Rle(rep("*", nrow(Ctcf_nora_summit_ranges_NS))),
                               peak = Ctcf_nora_summit_ranges_NS$peak )

save(Ctcf_nora_summit_ranges_ES,file=paste0(objects_directory,'Ctcf_nora_summit_ranges_ES.RData'))
save(Ctcf_nora_summit_ranges_NS,file=paste0(objects_directory,'Ctcf_nora_summit_ranges_NS.RData'))

Ctcf_nora_summit_ranges_ES_untreated = getSignalInBins( Ctcf_nora_summit_ranges_ES, ti_nora_ctcf_untreated_bp, 1 )
Ctcf_nora_summit_ranges_ES_iaa = getSignalInBins( Ctcf_nora_summit_ranges_ES, ti_nora_ctcf_iaa_bp, 1 )

Ctcf_nora_summit_ranges_NS_untreated = getSignalInBins( Ctcf_nora_summit_ranges_NS, ns_nora_ctcf_untreated_bp, 1 )
Ctcf_nora_summit_ranges_NS_iaa = getSignalInBins( Ctcf_nora_summit_ranges_NS, ns_nora_ctcf_iaa_bp, 1 )

save( Ctcf_nora_summit_ranges_ES_untreated, 
      Ctcf_nora_summit_ranges_ES_iaa, 
      Ctcf_nora_summit_ranges_NS_untreated, 
      Ctcf_nora_summit_ranges_NS_iaa,
      file=paste0(objects_directory,'Ctcf_signal_Nora_CTCF_peaks.RData'))
```

Display the effect of IAA on CTCF level.

```
load(paste0(objects_directory,'Ctcf_signal_Nora_CTCF_peaks.RData'))
x=log2((0.1+Ctcf_nora_summit_ranges_ES_iaa)/(0.1+Ctcf_nora_summit_ranges_ES_untreated))
y=log2((0.1+Ctcf_nora_summit_ranges_NS_iaa)/(0.1+Ctcf_nora_summit_ranges_NS_untreated))

par(mfrow=c(1,1),mar=c(5,5,1,1))
plot(density(x[,100]),col='red',lwd=3,ty='l',
     xlim=c(-10,10),main='',axes=F,ylim=c(0,0.5))
axis(1,lwd=3)
axis(2,lwd=3)
lines(density(y[,100]),col='blue',lwd=3 )
abline(v=0,lwd=3,col='black',lty=2)
```

```
t.test(x[,100])
```

```
## 
##  One Sample t-test
## 
## data:  x[, 100]
## t = -185.78, df = 13849, p-value < 2.2e-16
## alternative hypothesis: true mean is not equal to 0
## 95 percent confidence interval:
##  -1.985657 -1.944194
## sample estimates:
## mean of x 
## -1.964926
```

```
t.test(y[,100])
```

```
## 
##  One Sample t-test
## 
## data:  y[, 100]
## t = -213.78, df = 18626, p-value < 2.2e-16
## alternative hypothesis: true mean is not equal to 0
## 95 percent confidence interval:
##  -2.215984 -2.175719
## sample estimates:
## mean of x 
## -2.195851
```

```
df = data.frame(ES=density(x[,100])$y,NS=density(y[,100])$y)
write.table(df,file=paste0(source_data_directory,"ChIP-seq_FC_IAA.txt"),row.names = FALSE, sep="\t",quote=FALSE)
```

# Peaks in ES and NS cells

```
ti_atac_peaks = readBed_filterChroms(paste0(chipseq_directory,'ATAC_Seq_03-22_MusMus_ESC_MOD_SOX1-GFP_46C_2i_Rep_1_peaks.narrowPeak'),
                                     chroms=paste0('chr',c(1:19,'X','Y')), 7)
ns_atac_peaks = readBed_filterChroms(paste0(chipseq_directory,'ATAC_Seq_05-22_MusMus_es-NPC_MOD_SOX1-GFP_46C_Rep_1_peaks.narrowPeak'),
                                     chroms=paste0('chr',c(1:19,'X','Y')), 7)

ti_atac_summits = readNarrowPeak2getSummit(paste0(chipseq_directory,'ATAC_Seq_03-22_MusMus_ESC_MOD_SOX1-GFP_46C_2i_Rep_1_peaks.narrowPeak'),
                                     chroms=paste0('chr',c(1:19,'X','Y')), 5)
ns_atac_summits = readNarrowPeak2getSummit(paste0(chipseq_directory,'ATAC_Seq_05-22_MusMus_es-NPC_MOD_SOX1-GFP_46C_Rep_1_peaks.narrowPeak'),
                                     chroms=paste0('chr',c(1:19,'X','Y')), 5)


ctcf_motif$presence = 1

K27peaks_ES = readBed_filterChroms(paste0(chipseq_directory,'ChIP_Seq_12-21_MusMus_es-ESC_H3K27ac_MOD_SOX1-GFP_2i_Rep_1.narrowPeak'),
                                     chroms=paste0('chr',c(1:19,'X','Y')), 7)
K27peaks_NS = readBed_filterChroms( paste0(chipseq_directory, 'ChIP_Seq_12-21_MusMus_es-NPC_H3K27ac_MOD_SOX1-GFP_Rep_1.narrowPeak'),
                                     chroms=paste0('chr',c(1:19,'X','Y')), 7)

K27peaks_summit_ES  = readNarrowPeak2getSummit(paste0(chipseq_directory, 'ChIP_Seq_12-21_MusMus_es-ESC_H3K27ac_MOD_SOX1-GFP_2i_Rep_1.narrowPeak'),
                                         chroms=paste0('chr',c(1:19,'X','Y')), 5)

K27peaks_summit_NS = readNarrowPeak2getSummit(paste0(chipseq_directory, 'ChIP_Seq_12-21_MusMus_es-NPC_H3K27ac_MOD_SOX1-GFP_Rep_1.narrowPeak'),
                                         chroms=paste0('chr',c(1:19,'X','Y')), 5)


ti_enhancers = K27peaks_ES[-queryHits(findOverlaps(K27peaks_ES,promGRExt))]
ns_enhancers = K27peaks_NS[-queryHits(findOverlaps(K27peaks_NS,promGRExt))]

ti_enhancers_atac = ti_atac_peaks[queryHits(findOverlaps(ti_atac_peaks,ti_enhancers))]
ns_enhancers_atac = ns_atac_peaks[queryHits(findOverlaps(ns_atac_peaks,ns_enhancers))]
```

# CTCF, gene expression, and loop gain

Induced genes are closer to each other than reduced genes

```
par(mfrow=c(1,2),mar=c(5,5,1,1),cex.axis=1)
boxplot(elementMetadata(distanceToNearest(promoters_tss_gr[which(promoters_tss_gr$gene_id %in% NS_sig_genes_up)]))$distance/1000,
        elementMetadata(distanceToNearest(promoters_tss_gr[which(promoters_tss_gr$gene_id %in% NS_sig_genes_dn)]))$distance/1000,
        outline=FALSE,border=c('green4','orange3'),col='white',names=c("Up","Down"),ylab="distance (kbp)")
ks.test(elementMetadata(distanceToNearest(promoters_tss_gr[which(promoters_tss_gr$gene_id %in% NS_sig_genes_up)]))$distance,
        elementMetadata(distanceToNearest(promoters_tss_gr[which(promoters_tss_gr$gene_id %in% NS_sig_genes_dn)]))$distance)
```

```
## Warning in
## ks.test(elementMetadata(distanceToNearest(promoters_tss_gr[which(promoters_tss_gr$gene_id
## %in% : p-value will be approximate in the presence of ties
```

```
## 
##  Two-sample Kolmogorov-Smirnov test
## 
## data:  elementMetadata(distanceToNearest(promoters_tss_gr[which(promoters_tss_gr$gene_id %in% NS_sig_genes_up)]))$distance and elementMetadata(distanceToNearest(promoters_tss_gr[which(promoters_tss_gr$gene_id %in% NS_sig_genes_dn)]))$distance
## D = 0.24865, p-value = 3.431e-07
## alternative hypothesis: two-sided
```

```
## distance to nearest enhancer
boxplot(elementMetadata(distanceToNearest(promoters_tss_gr[which(promoters_tss_gr$gene_id %in% NS_sig_genes_up)],ns_enhancers_atac))$distance/1000,
        elementMetadata(distanceToNearest(promoters_tss_gr[which(promoters_tss_gr$gene_id %in% NS_sig_genes_dn)],ns_enhancers_atac))$distance/1000,
        outline=FALSE,border=c('green4','orange3'),col='white',names=c("Up","Down"))
```

Boxplot data

```
theNtimes = max( c(length(NS_sig_genes_up),length(NS_sig_genes_dn)))
df = data.frame( Prom_prom_up = rep(NA,theNtimes), 
                 Prom_prom_dn = rep(NA,theNtimes),
                 Prom_enh_up = rep(NA,theNtimes), 
                 Prom_enh_dn = rep(NA,theNtimes) )
df$Prom_prom_up[1:length(elementMetadata(distanceToNearest(promoters_tss_gr[which(promoters_tss_gr$gene_id %in% NS_sig_genes_up)]))$distance/1000)] = elementMetadata(distanceToNearest(promoters_tss_gr[which(promoters_tss_gr$gene_id %in% NS_sig_genes_up)]))$distance/1000
df$Prom_prom_dn[1:length(elementMetadata(distanceToNearest(promoters_tss_gr[which(promoters_tss_gr$gene_id %in% NS_sig_genes_dn)]))$distance/1000)] = elementMetadata(distanceToNearest(promoters_tss_gr[which(promoters_tss_gr$gene_id %in% NS_sig_genes_dn)]))$distance/1000

df$Prom_enh_up[1:length(elementMetadata(distanceToNearest(promoters_tss_gr[which(promoters_tss_gr$gene_id %in% NS_sig_genes_up)],ns_enhancers_atac))$distance/1000)] = elementMetadata(distanceToNearest(promoters_tss_gr[which(promoters_tss_gr$gene_id %in% NS_sig_genes_up)],ns_enhancers_atac))$distance/1000
df$Prom_enh_dn[1:length(elementMetadata(distanceToNearest(promoters_tss_gr[which(promoters_tss_gr$gene_id %in% NS_sig_genes_dn)],ns_enhancers_atac))$distance/1000)] = elementMetadata(distanceToNearest(promoters_tss_gr[which(promoters_tss_gr$gene_id %in% NS_sig_genes_dn)],ns_enhancers_atac))$distance/1000

write.table(df,file=paste0(source_data_directory,"boxplots_Prom_enh_Prom_Prom_distance.txt"),row.names = FALSE, sep="\t",quote=FALSE)
```

# Insulators in the NS cells

Do we see that architectural CTCF peaks separate the induced genes from enhancers more often than reduced genes?

Find the smallest loop domain for each up and down gene, identify regions that flank the insulators (500 upstream), find the region with the highest number of enhancers and compare for induced and reduced genes. We will also check the number of CTCF peaks.

```
nsctmm10peak = readBed_filterChroms( paste0(chipseq_directory, 'NS_CTCF_ChIP_NIAMS_merged_filtered_peaks.narrowPeak'),
                                     chroms=paste0('chr',c(1:19,'X','Y')), 7)

nsctmm10 = readNarrowPeak2getSummit( paste0(chipseq_directory,'NS_CTCF_ChIP_NIAMS_merged_filtered_peaks.narrowPeak'),
                                     chroms=paste0('chr',c(1:19,'X','Y')), 5)
nsctmm10$score = nsctmm10peak$score
nsctmm10peak = appendMotifInformation( nsctmm10peak, ctcf_motif,summitFile=nsctmm10 )


ctcf_peaks_ns_mm10_strand = nsctmm10peak[which(nsctmm10peak$motif_strand != "*")]
strand(ctcf_peaks_ns_mm10_strand) = ctcf_peaks_ns_mm10_strand$motif_strand


sum( elementMetadata(distanceToNearest(promoters_tss_gr[which(promoters_tss_gr$gene_id %in% NS_sig_genes_up)],ctcf_peaks_ns_mm10_strand,ignore.strand=TRUE))$distance == 0)/length(NS_sig_genes_up)
```

```
## [1] 0.1731844
```

```
sum( elementMetadata(distanceToNearest(promoters_tss_gr[which(promoters_tss_gr$gene_id %in% NS_sig_genes_dn)],ctcf_peaks_ns_mm10_strand,ignore.strand=TRUE))$distance == 0)/length(NS_sig_genes_dn)
```

```
## [1] 0.4545455
```

```
m=matrix(c(sum( elementMetadata(distanceToNearest(promoters_tss_gr[which(promoters_tss_gr$gene_id %in% NS_sig_genes_up)],ctcf_peaks_ns_mm10_strand,ignore.strand=TRUE))$distance == 0),
                     length(NS_sig_genes_up),
                     sum( elementMetadata(distanceToNearest(promoters_tss_gr[which(promoters_tss_gr$gene_id %in% NS_sig_genes_dn)],ctcf_peaks_ns_mm10_strand,ignore.strand=TRUE))$distance == 0),
                     length(NS_sig_genes_dn)),2,2)
fisher.test(m)
```

```
## 
##  Fisher's Exact Test for Count Data
## 
## data:  m
## p-value = 2.369e-07
## alternative hypothesis: true odds ratio is not equal to 1
## 95 percent confidence interval:
##  0.2591761 0.5589507
## sample estimates:
## odds ratio 
##  0.3815533
```

```
par(mfrow=c(1,1))
barplot(100*m[1,]/m[2,],col=c('green4','orange3'),
        ylab="%",ylim=c(0,50),names=c("up","down"))
axis(2,lwd=2)
```

```
m
```

```
##      [,1] [,2]
## [1,]   62   90
## [2,]  358  198
```

How many enhancers flank the loop domains that contain different classes of genes?

```
dis2anchor_induced = c()
induced_intervals_left = data.frame()
induced_intervals_right = data.frame()
induced_smallest_loop = c()
induced_largest_loop = c()


for( gene in NS_sig_genes_up ){
  thisGene=promoters_tss_gr[which(promoters_tss_gr$gene_id %in% gene)]
  tp = findOverlaps(ns_loops_gr,thisGene)
  if(length(tp)>0){
  loops = ns_loops_gr[queryHits(tp)]
  loops2 = loops[which.max(width(loops))]
  loops = loops[which.min(width(loops))]
  
  induced_smallest_loop = rbind(induced_smallest_loop,
                                data.frame(chrom(loops),start(loops),end(loops)))
  induced_largest_loop = rbind(induced_largest_loop, data.frame(chrom(loops2),start(loops2),end(loops2)))
  
  dis2anchor_induced = c(dis2anchor_induced,elementMetadata(distanceToNearest(thisGene,c(ns_loops_left_anchor[loops$loop],ns_loops_right_anchor[loops$loop])))$distance)
  induced_intervals_left = rbind(induced_intervals_left,data.frame(chrom(loops),start(loops)-500000,start(loops)))
  induced_intervals_right = rbind(induced_intervals_right,data.frame(chrom(loops),end(loops),end(loops)+500000))}
}
induced_smallest_loop_gr = getGR(induced_smallest_loop[,1],induced_smallest_loop[,2],induced_smallest_loop[,3],0)

induced_intervals_left_gr = getGR(induced_intervals_left[,1],induced_intervals_left[,2],induced_intervals_left[,3],0)
induced_intervals_right_gr = getGR(induced_intervals_right[,1],induced_intervals_right[,2],induced_intervals_right[,3],0)
induced_smallest_loop_gr = getGR(induced_smallest_loop[,1],induced_smallest_loop[,2],induced_smallest_loop[,3],0)
induced_largest_loop_gr = getGR(induced_largest_loop[,1],induced_largest_loop[,2],induced_largest_loop[,3],0)

## reduced genes
dis2anchor_reduced = c()
reduced_intervals_left = data.frame()
reduced_intervals_right = data.frame()
reduced_smallest_loop = c()
reduced_largest_loop = c()

for( gene in NS_sig_genes_dn ){
  thisGene=promoters_tss_gr[which(promoters_tss_gr$gene_id %in% gene)]
  tp = findOverlaps(ns_loops_gr,thisGene)
  if(length(tp)>0){
  loops = ns_loops_gr[queryHits(tp)]
  loops2 = loops[which.max(width(loops))]
  loops = loops[which.min(width(loops))]
  reduced_smallest_loop = rbind(reduced_smallest_loop,
                                data.frame(chrom(loops),start(loops),end(loops)))
  reduced_largest_loop = rbind(reduced_largest_loop,
                                data.frame(chrom(loops2),start(loops2),end(loops2)))
  dis2anchor_reduced = c(dis2anchor_reduced,elementMetadata(distanceToNearest(thisGene,c(ns_loops_left_anchor[loops$loop],ns_loops_right_anchor[loops$loop])))$distance)
  reduced_intervals_left = rbind(reduced_intervals_left,data.frame(chrom(loops),start(loops)-500000,start(loops)))
  reduced_intervals_right = rbind(reduced_intervals_right,data.frame(chrom(loops),end(loops),end(loops)+500000))}
}


reduced_intervals_left_gr = getGR(reduced_intervals_left[,1],reduced_intervals_left[,2],reduced_intervals_left[,3],0)
reduced_intervals_right_gr = getGR(reduced_intervals_right[,1],reduced_intervals_right[,2],reduced_intervals_right[,3],0)
reduced_smallest_loop_gr = getGR(reduced_smallest_loop[,1],reduced_smallest_loop[,2],reduced_smallest_loop[,3],0)
reduced_largest_loop_gr = getGR(reduced_largest_loop[,1],reduced_largest_loop[,2],reduced_largest_loop[,3],0)

## ------------
dis2anchor_random = c()
random_intervals_left = data.frame()
random_intervals_right = data.frame()
random_smallest_loop = c()
random_largest_loop = c()

for( gene in promoters_tss_gr[sample(seq(1,length(promoters_tss_gr)),2000,replace = FALSE)]$gene_id ){
  thisGene=promoters_tss_gr[which(promoters_tss_gr$gene_id %in% gene)]
  tp = findOverlaps(ns_loops_gr,thisGene)
  if(length(tp)>0){
  loops = ns_loops_gr[queryHits(tp)]
  loops2 = loops[which.max(width(loops))]
  loops = loops[which.min(width(loops))]
  random_smallest_loop = rbind(random_smallest_loop,
                               data.frame(chrom(loops),start(loops),end(loops)))
  random_largest_loop = rbind(random_largest_loop,
                               data.frame(chrom(loops2),start(loops2),end(loops2)))
  dis2anchor_random = c(dis2anchor_random,elementMetadata(distanceToNearest(thisGene,c(ns_loops_left_anchor[loops$loop],ns_loops_right_anchor[loops$loop])))$distance)
  random_intervals_left = rbind(random_intervals_left,data.frame(chrom(loops),start(loops)-500000,start(loops)))
  random_intervals_right = rbind(random_intervals_right,data.frame(chrom(loops),end(loops),end(loops)+500000))}
}


random_intervals_left_gr = getGR(random_intervals_left[,1],random_intervals_left[,2],random_intervals_left[,3],0)
random_intervals_right_gr = getGR(random_intervals_right[,1],random_intervals_right[,2],random_intervals_right[,3],0)
random_smallest_loop_gr = getGR(random_smallest_loop[,1],random_smallest_loop[,2],random_smallest_loop[,3],0)
random_largest_loop_gr = getGR(random_largest_loop[,1],random_largest_loop[,2],random_largest_loop[,3],0)


## ----------
induced_left_right = data.frame(left=countOverlaps(induced_intervals_left_gr,
                                                   ns_enhancers_atac),

                                right = countOverlaps(induced_intervals_right_gr,
                                                      ns_enhancers_atac))

reduced_left_right = data.frame(left=countOverlaps(reduced_intervals_left_gr,
                                                   ns_enhancers_atac),

                                right = countOverlaps(reduced_intervals_right_gr,
                                                      ns_enhancers_atac))

random_left_right = data.frame(left=countOverlaps(random_intervals_left_gr,
                                                   ns_enhancers_atac),

                                right = countOverlaps(random_intervals_right_gr,
                                                      ns_enhancers_atac))
```

Check the landscape in the loops

```
induced_largest_loop_gr$loop = paste(chrom(induced_largest_loop_gr),start(induced_largest_loop_gr),sep="_")
reduced_largest_loop_gr$loop = paste(chrom(reduced_largest_loop_gr),start(reduced_largest_loop_gr),sep="_")
induced_largest_loop_gr_filt = induced_largest_loop_gr[which(! duplicated(induced_largest_loop_gr$loop))]
reduced_largest_loop_gr_filt = reduced_largest_loop_gr[which(! duplicated(reduced_largest_loop_gr$loop))]

boxplot(rowSums(induced_left_right[which(! duplicated(induced_largest_loop_gr$loop)),]),
        rowSums(reduced_left_right[which(! duplicated(reduced_largest_loop_gr$loop)),]),
        rowSums(random_left_right),col="white",border=c("green4","orange3","gray60"),
        names=c("up","down","random"),ylab="number of enhancers in flanks")
axis(1,lwd=2, at=c(1,2,3),c("up","down","random"))
axis(2,lwd=2)
box(col='black',lwd=2)
```

```
t.test(rowSums(induced_left_right[which(! duplicated(induced_largest_loop_gr$loop)),]),
       rowSums(reduced_left_right[which(! duplicated(reduced_largest_loop_gr$loop)),]))
```

```
## 
##  Welch Two Sample t-test
## 
## data:  rowSums(induced_left_right[which(!duplicated(induced_largest_loop_gr$loop)), ]) and rowSums(reduced_left_right[which(!duplicated(reduced_largest_loop_gr$loop)), ])
## t = 4.6081, df = 313.74, p-value = 5.919e-06
## alternative hypothesis: true difference in means is not equal to 0
## 95 percent confidence interval:
##  2.053708 5.114292
## sample estimates:
## mean of x mean of y 
##    12.000     8.416
```

```
t.test(rowSums(random_left_right[which(! duplicated(reduced_largest_loop_gr$loop)),]),rowSums(reduced_left_right))
```

```
## 
##  Welch Two Sample t-test
## 
## data:  rowSums(random_left_right[which(!duplicated(reduced_largest_loop_gr$loop)), ]) and rowSums(reduced_left_right)
## t = -2.8088, df = 243.77, p-value = 0.005376
## alternative hypothesis: true difference in means is not equal to 0
## 95 percent confidence interval:
##  -3.6673615 -0.6439448
## sample estimates:
## mean of x mean of y 
##  7.064000  9.219653
```

```
theNtimes = max( length(rowSums(induced_left_right[which(! duplicated(induced_largest_loop_gr$loop)),])),
                 length(rowSums(reduced_left_right[which(! duplicated(reduced_largest_loop_gr$loop)),])),
                 length(rowSums(random_left_right)) )
df = data.frame( induced = rep(NA,theNtimes),
                 reduced = rep(NA,theNtimes),
                 random = rep(NA,theNtimes) )

df$induced[1:length(rowSums(induced_left_right[which(! duplicated(induced_largest_loop_gr$loop)),]))] = rowSums(induced_left_right[which(! duplicated(induced_largest_loop_gr$loop)),])
df$reduced[1:length(rowSums(reduced_left_right[which(! duplicated(reduced_largest_loop_gr$loop)),]))] = rowSums(reduced_left_right[which(! duplicated(reduced_largest_loop_gr$loop)),])
df$random[1:length(rowSums(random_left_right))] = rowSums(random_left_right)

write.table(df,file=paste0(source_data_directory,"boxplots_flanks.txt"),row.names = FALSE, sep="\t",quote=FALSE)
```

# PLA

```
rnase = read.delim(paste0(data_directory,"PLA_microscopy/Fig-RNaseA-PLA-Ddx5-CTCF_Fus-CTCF-WT-ES_NS.txt"),
                   header=TRUE)
rnase$Cond=factor(paste(rnase$Cell.type, rnase$Treatment),
                  levels=c("ESC Untreated","ESC RNaseA", "NSC Untreated", "NSC RNaseA"))

par(bty="n",mfrow=c(2,1),mar=c(3,3,2,1))
boxplot( split(rnase[rnase$PLA=="Ddx5-Ctcf",2],
               rnase$Cond[rnase$PLA=="Ddx5-Ctcf"]),
         col="white",border=c("red","red","blue","blue"),
         lwd=2,ylim=c(0,20),axes=FALSE,main="Ddx5-Ctcf")
axis(1,c(1,2,3,4),
     c("","", "", ""),
     lwd=2)
axis(2,lwd=2)

boxplot( split(rnase[rnase$PLA=="Fus-Ctcf",2],
               rnase$Cond[rnase$PLA=="Fus-Ctcf" ]),
         col="white",border=c("red","red","blue","blue"),
         lwd=2,ylim=c(0,15),main="Fus-Ctcf",axes=FALSE)
axis(1,c(1,2,3,4),
     c("ESC Unt.","ESC RNaseA", "NSC Unt.", "NSC RNaseA"),
     lwd=2)
axis(2,lwd=2)
```

# PLA : Ctcf-Nono

```
Ctcf_nono = read.delim(paste0(data_directory,"PLA_microscopy/Fig-PLA-Nono-Ctcf-WT-ES_NS.txt"),
                   header=TRUE)
x = split(Ctcf_nono[,2],Ctcf_nono$Cell.type)

par(bty="n",mfrow=c(1,1),mar=c(3,3,2,1),pty="m")
boxplot( x,
         col="white",border=c("red","blue"),
         lwd=2,ylim=c(0,40),axes=FALSE,main="Ctcf-Nono")
axis(1,c(1,2), c("ES","NS"), lwd=2)
axis(2,lwd=2)
```

```
t.test(x[[1]],x[[2]])
```

```
## 
##  Welch Two Sample t-test
## 
## data:  x[[1]] and x[[2]]
## t = -3.0945, df = 33.582, p-value = 0.003958
## alternative hypothesis: true difference in means is not equal to 0
## 95 percent confidence interval:
##  -13.552177  -2.804965
## sample estimates:
## mean of x mean of y 
##  13.00000  21.17857
```

# PLA : FUS-Ddx5

```
ddx_fus = read.delim(paste0(data_directory,"PLA_microscopy/Fig-PLA-Ddx5-Fus-WT-ES_NS.txt"),
                   header=TRUE)
par(bty="n",mfrow=c(1,1),mar=c(3,3,2,1))
boxplot( split(ddx_fus[,2],
               ddx_fus$Cell.type),
         col="white",border=c("red","blue"),
         lwd=2,ylim=c(0,40),axes=FALSE,main="Ddx5-Fus")
axis(1,c(1,2),
     c("ES","NS"),
     lwd=2)
axis(2,lwd=2)
```

# PANTR1 PLA Ddx5-CTCF

```
pantr1 = read.delim(paste0(data_directory,"PLA_microscopy/PLA_Ddx5-CTCF_end_Dec_2023.txt"),
                   header=TRUE)
pantr1$Genotype = factor(pantr1$Genotype,levels=c("Wildtype-A2","Wildtype-A3",
                                                  "Pantr1-KO-PB6","Pantr1-KO-PE3"))


pantr1s = split(pantr1[,2],pantr1$Genotype)
par(bty="n",mfrow=c(1,1),mar=c(3,3,2,1))
boxplot( pantr1s[c(1,2,4,3)],
         col="white",border=c("blue","blue","gray60","gray60"),
         lwd=2,ylim=c(0,100),axes=FALSE,main="CTCF-Ddx5")
axis(1,c(1,2,3,4),
     c("Wt1","Wt2",
       "PE3","PB6"),
     lwd=2)
axis(2,lwd=2)
```

```
t.test(c(pantr1s[[1]],pantr1s[[2]]), c(pantr1s[[3]],pantr1s[[4]]))
```

```
## 
##  Welch Two Sample t-test
## 
## data:  c(pantr1s[[1]], pantr1s[[2]]) and c(pantr1s[[3]], pantr1s[[4]])
## t = 18.201, df = 129.76, p-value < 2.2e-16
## alternative hypothesis: true difference in means is not equal to 0
## 95 percent confidence interval:
##  38.40084 47.76715
## sample estimates:
## mean of x mean of y 
##  66.28947  23.20548
```

# PANTR1 PLA FUS-CTCF

```
pantr1_fus = read.delim(paste0(data_directory,"PLA_microscopy/PLA-Fus-CTCF-Pantr-KO-new.txt"),
                   header=TRUE)
pantr1_fus$Genotype = factor(pantr1_fus$Genotype,
                             levels=c("Wildtype-A2","Wildtype-A3",
                                      "Pantr1-KO-PB6","Pantr1-KO-PE3"))
pantr1_fus = pantr1_fus[pantr1_fus$Genotype %in% c("Wildtype-A2","Wildtype-A3","Pantr1-KO-PB6","Pantr1-KO-PE3"),]
pantr1_fus$Genotype = droplevels(pantr1_fus$Genotype)

pantr1s = split(pantr1_fus[,2],pantr1_fus$Genotype)
par(bty="n",mfrow=c(1,1),mar=c(3,3,2,1))
boxplot( pantr1s,
         col="white",border=c("blue","blue","gray60","gray60"),
         lwd=2,ylim=c(0,100),axes=FALSE,main="CTCF-FUS")
axis(1,c(1,2,3,4),
     c("Wt1","Wt2",
       "PE3","PB6"),
     lwd=2)
axis(2,lwd=2)
```

```
t.test(c(unlist(pantr1s[c(1,2)])),c(unlist(pantr1s[c(3,4)])))
```

```
## 
##  Welch Two Sample t-test
## 
## data:  c(unlist(pantr1s[c(1, 2)])) and c(unlist(pantr1s[c(3, 4)]))
## t = 6.2177, df = 120.78, p-value = 7.483e-09
## alternative hypothesis: true difference in means is not equal to 0
## 95 percent confidence interval:
##  10.27250 19.87033
## sample estimates:
## mean of x mean of y 
##  58.63768  43.56627
```

# qRT-PCR

```
pantr1_qPCR = c(0.34,0,0.01,0,0.01,0)
names(pantr1_qPCR) = c("Wt +","Wt -","PB6+","PB6-","PE3+","PE3-")
barplot(pantr1_qPCR,col=c("blue","blue",rep("gray",4)),ylim=c(0,0.4) )
axis(2,lwd=2)
```

# CTCF clustering - AiryScan on pre-extracted nuclei

```
ctcf_clusters = read.delim(paste0(data_directory,"PLA_microscopy/Figure_CTCF-Clusters-WT_ES_NS.txt"),header=TRUE)

par(bty="n",mfrow=c(1,1),mar=c(3,3,2,1))
x=split(ctcf_clusters[,1],ctcf_clusters$Cell.type)
boxplot( x, col="white",border=c("red","blue"),
         lwd=2,ylim=c(0,200),axes=FALSE,main="CTCF cluster size")
axis(1,c(1,2),
     c('ES','NS'),
     lwd=2)
axis(2,lwd=2)
```

```
t.test(x[[1]],x[[2]])
```

```
## 
##  Welch Two Sample t-test
## 
## data:  x[[1]] and x[[2]]
## t = -5.9133, df = 64.346, p-value = 1.403e-07
## alternative hypothesis: true difference in means is not equal to 0
## 95 percent confidence interval:
##  -67.54590 -33.43454
## sample estimates:
## mean of x mean of y 
##  19.67186  70.16207
```

# CTCF peaks

Overwhelming majority of CTCF peaks intersects a motif.

```
ctcf_motif = readBed_filterChromsStraded( paste0(data_directory,'CTCF_HUMAN.H11MO.0.A.bed'),
                                          chroms=paste0('chr',c(1:19,'X','Y')), 5 )
ctcf_wt_NS_peaks = import.bed( paste0(chipseq_directory,'ChIP_SEQ_CTCF_Merged_rpgc_narrow_summits.bed'))
seqlevelsStyle(ctcf_wt_NS_peaks)="ucsc"
ctcf_wt_NS_peaks = ctcf_wt_NS_peaks[which(chrom(ctcf_wt_NS_peaks) %in% paste0("chr",c(1:19,"x")))]

ctcf_wt_NS_peaks = GenomicRanges::resize(ctcf_wt_NS_peaks,width=200,fix="center")
ctcf_wt_NS_peaks = appendMotifInformation( ctcf_wt_NS_peaks, ctcf_motif,summitFile=ctcf_wt_NS_peaks )
table(ctcf_wt_NS_peaks$motif_strand)
```

```
## 
##     *     +     - 
##  5038 18472 18722
```

```
ctcf_wt_NS_peaks_summit = GenomicRanges::resize(ctcf_wt_NS_peaks,fix="center",1)
names(ctcf_wt_NS_peaks_summit) = seq(1:length(ctcf_wt_NS_peaks_summit))
ctcf_wt_NS_peaks_resized = GenomicRanges::resize( ctcf_wt_NS_peaks_summit, fix="center",width=500 )

write.table( ctcf_wt_NS_peaks,
             file=paste0(data_directory,"ctcf_wt_NS_peaks.txt"),
             sep='\t',quote=FALSE,row.names=FALSE,col.names=TRUE )
```

## CTCF AUC

```
a3_1_ctcf_rpgc = import.bw(paste0(chipseq_directory,"ChIP_Seq_CTCF_06-23_MusMus_es-NPC_MOD_CTCF-Cterm_HALO_A3_Control_Rep_1_RPGC.bw"))
a3_2_ctcf_rpgc = import.bw(paste0(chipseq_directory,"ChIP_Seq_CTCF_08-23_MusMus_es-NPC_MOD_CTCF-Cterm_HALO_A3_Control_Rep_2_RPGC.bw"))
cb1_ctcf_rpgc = import.bw(paste0(chipseq_directory,"ChIP_Seq_CTCF_03-23_MusMus_es-NPC_DDX5_KO_CTCF-Cterm_HALO_CB1_Rep_1_RPGC.bw"))
ce10_ctcf_rpgc = import.bw(paste0(chipseq_directory,"ChIP_Seq_CTCF_03-23_MusMus_es-NPC_DDX5_KO_CTCF-Cterm_HALO_CE10_Rep_1_RPGC.bw"))
seqlevelsStyle(a3_1_ctcf_rpgc) = "ucsc"
seqlevelsStyle(a3_2_ctcf_rpgc) = "ucsc"
seqlevelsStyle(cb1_ctcf_rpgc) = "ucsc"
seqlevelsStyle(ce10_ctcf_rpgc) = "ucsc"
```

```
a3_1_ctcf_raw = import.bw(paste0(chipseq_directory,"ChIP_Seq_CTCF_06-23_MusMus_es-NPC_MOD_CTCF-Cterm_HALO_A3_Control_Rep_1_filtered.bw"))
a3_2_ctcf_raw = import.bw(paste0(chipseq_directory,"ChIP_Seq_CTCF_08-23_MusMus_es-NPC_MOD_CTCF-Cterm_HALO_A3_Control_Rep_2_filtered.bw"))
cb1_ctcf_raw = import.bw(paste0(chipseq_directory,"ChIP_Seq_CTCF_03-23_MusMus_es-NPC_DDX5_KO_CTCF-Cterm_HALO_CB1_Rep_1_filtered.bw"))
ce10_ctcf_raw = import.bw(paste0(chipseq_directory,"ChIP_Seq_CTCF_03-23_MusMus_es-NPC_DDX5_KO_CTCF-Cterm_HALO_CE10_Rep_1_filtered.bw"))
seqlevelsStyle(a3_1_ctcf_raw) = "ucsc"
seqlevelsStyle(a3_2_ctcf_raw) = "ucsc"
seqlevelsStyle(cb1_ctcf_raw) = "ucsc"
seqlevelsStyle(ce10_ctcf_raw) = "ucsc"
```

```
ctcf_NS_ranges = do.call('rbind', lapply( as.list(seq(1:length(ctcf_wt_NS_peaks_summit))), 
                                          function(i){
  g = ctcf_wt_NS_peaks_summit[i]
  tss = start(g)
  chr = as.character( chrom(g) )
  peak=names(ctcf_wt_NS_peaks_summit)[i]
      return( data.frame( chr = rep( (chr), 200),
                          starts = seq( tss-1000, tss+990, length.out=200),
                          ends =  seq( tss-1000, tss+990, length.out=200)+9,
                          peak = rep( peak, 200) ) ) } ) )


ctcf_NS_ranges = GRanges(seqnames = Rle(ctcf_NS_ranges$chr),
                         ranges = IRanges(as.numeric(ctcf_NS_ranges$starts),
                                          end = as.numeric(ctcf_NS_ranges$ends),
                                          names = seq(1, nrow(ctcf_NS_ranges))),
                         strand = Rle(rep("*", nrow(ctcf_NS_ranges))),
                         peak = ctcf_NS_ranges$peak )

save(ctcf_NS_ranges,file=paste0(objects_directory,'ctcf_NS_ranges.RData'))

a3_1_ctcf_rpgc_ctcf_AP = getSignalInBins(ctcf_NS_ranges,a3_1_ctcf_rpgc,1)
a3_2_ctcf_rpgc_ctcf_AP = getSignalInBins(ctcf_NS_ranges,a3_2_ctcf_rpgc,1)
cb1_ctcf_rpgc_ctcf_AP = getSignalInBins(ctcf_NS_ranges,cb1_ctcf_rpgc,1)
ce10_ctcf_rpgc_ctcf_AP = getSignalInBins(ctcf_NS_ranges,ce10_ctcf_rpgc,1)
rownames(a3_1_ctcf_rpgc_ctcf_AP) = names(ctcf_wt_NS_peaks_summit)
rownames(a3_2_ctcf_rpgc_ctcf_AP) = names(ctcf_wt_NS_peaks_summit)
rownames(cb1_ctcf_rpgc_ctcf_AP) = names(ctcf_wt_NS_peaks_summit)
rownames(ce10_ctcf_rpgc_ctcf_AP) = names(ctcf_wt_NS_peaks_summit)

save(a3_1_ctcf_rpgc_ctcf_AP,
     a3_2_ctcf_rpgc_ctcf_AP,
     cb1_ctcf_rpgc_ctcf_AP,
     ce10_ctcf_rpgc_ctcf_AP,
     file=paste0(objects_directory,"CTCF_Ddx5ko_NS_A3_RPGC.RData"))

a3_1_ctcf_raw_ctcf_AP = getSignalInBins(ctcf_NS_ranges,a3_1_ctcf_raw,1)
a3_2_ctcf_raw_ctcf_AP = getSignalInBins(ctcf_NS_ranges,a3_2_ctcf_raw,1)
cb1_ctcf_raw_ctcf_AP = getSignalInBins(ctcf_NS_ranges,cb1_ctcf_raw,1)
ce10_ctcf_raw_ctcf_AP = getSignalInBins(ctcf_NS_ranges,ce10_ctcf_raw,1)
rownames(a3_1_ctcf_raw_ctcf_AP) = names(ctcf_wt_NS_peaks_summit)
rownames(a3_2_ctcf_raw_ctcf_AP) = names(ctcf_wt_NS_peaks_summit)
rownames(cb1_ctcf_raw_ctcf_AP) = names(ctcf_wt_NS_peaks_summit)
rownames(ce10_ctcf_raw_ctcf_AP) = names(ctcf_wt_NS_peaks_summit)

save(a3_1_ctcf_raw_ctcf_AP,
     a3_2_ctcf_raw_ctcf_AP,
     cb1_ctcf_raw_ctcf_AP,
     ce10_ctcf_raw_ctcf_AP,
     file=paste0(objects_directory,"CTCF_Ddx5ko_NS_A3_RAW.RData"))
```

```
load(paste0(objects_directory,"CTCF_Ddx5ko_NS_A3_RAW.RData"))
load(paste0(objects_directory,'ctcf_NS_ranges.RData'))
a=90; b=110

elbat_ctcf = data.frame( a3_1 = ( rowSums(a3_1_ctcf_raw_ctcf_AP[,a:b])),
                         a3_2 = ( rowSums(a3_2_ctcf_raw_ctcf_AP[,a:b])),
                         cb1 = ( rowSums(cb1_ctcf_raw_ctcf_AP[,a:b])),
                         ce10 = ( rowSums(ce10_ctcf_raw_ctcf_AP[,a:b])) )
metadata_CTCF_peaks = data.frame( genotype = c("wt","wt","Ddx5","Ddx5"),
                                  clone = c("a3_1","a3_2","cb1","ce10"),
                                  row.names = colnames(elbat_ctcf))
Ddx5_ctcf_Deseq2_RPGC = DESeqDataSetFromMatrix(
 countData = elbat_ctcf,
 colData = metadata_CTCF_peaks,
 design = ~ genotype )
```

```
## converting counts to integer mode
```

```
## Warning in DESeqDataSet(se, design = design, ignoreRank): some variables in
## design formula are characters, converting to factors
```

```
Ddx5_ctcf_Deseq2_RPGC = DESeq(Ddx5_ctcf_Deseq2_RPGC, fitType = 'local')
```

```
## estimating size factors
```

```
## estimating dispersions
```

```
## gene-wise dispersion estimates
```

```
## mean-dispersion relationship
```

```
## final dispersion estimates
```

```
## fitting model and testing
```

```
res_Ddx5_ctcf_Deseq2_RPGC = results(Ddx5_ctcf_Deseq2_RPGC, 
                              contrast = c("genotype", "Ddx5", "wt"))
summary( res_Ddx5_ctcf_Deseq2_RPGC )
```

```
## 
## out of 42232 with nonzero total read count
## adjusted p-value < 0.1
## LFC > 0 (up)       : 343, 0.81%
## LFC < 0 (down)     : 398, 0.94%
## outliers [1]       : 0, 0%
## low counts [2]     : 0, 0%
## (mean count < 7)
## [1] see 'cooksCutoff' argument of ?results
## [2] see 'independentFiltering' argument of ?results
```

```
ctcf_normalized = counts( Ddx5_ctcf_Deseq2_RPGC,normalized=TRUE )
```

```
heatscatter( x=res_Ddx5_ctcf_Deseq2_RPGC$baseMean,
             y=res_Ddx5_ctcf_Deseq2_RPGC$log2FoldChange, 
             pch=19, cex=0.1, add.contour = T, 
             xlim=c(0,1200),ylim=c(-2,2),
             xlab=expression(Log[2] ~ (Signal)),
             ylab=expression(Log[2] ~ (LFC)), 
             main="WT mutant")
abline( h=0 )
axis(1,lwd=2)
axis(2,lwd=2)
box(col="black",lwd=2)

df = data.frame( M=res_Ddx5_ctcf_Deseq2_RPGC$baseMean,A=res_Ddx5_ctcf_Deseq2_RPGC$log2FoldChange)
write.table(df,file=paste0(source_data_directory,"MA_ddx5KO.txt"),row.names = FALSE, sep="\t",quote=FALSE)
```

```
res_Ddx5_ctcf_Deseq2_RPGC = res_Ddx5_ctcf_Deseq2_RPGC[!is.na(res_Ddx5_ctcf_Deseq2_RPGC$pvalue),]
res_Ddx5_ctcf_Deseq2_RPGC = res_Ddx5_ctcf_Deseq2_RPGC[!is.na(res_Ddx5_ctcf_Deseq2_RPGC$padj),]

res_Ddx5_ctcf_Deseq2_RPGC = res_Ddx5_ctcf_Deseq2_RPGC[!is.na(res_Ddx5_ctcf_Deseq2_RPGC$padj),]
res_Ddx5_ctcf_Deseq2_RPGC$col = "gray90"

res_Ddx5_ctcf_Deseq2_RPGC$col[res_Ddx5_ctcf_Deseq2_RPGC$padj<0.25 & res_Ddx5_ctcf_Deseq2_RPGC$log2FoldChange>0]="#049494"
res_Ddx5_ctcf_Deseq2_RPGC$col[res_Ddx5_ctcf_Deseq2_RPGC$padj<0.25 & res_Ddx5_ctcf_Deseq2_RPGC$log2FoldChange<0 ]="#305494"
res_Ddx5_ctcf_Deseq2_RPGC$pvalue[res_Ddx5_ctcf_Deseq2_RPGC$pvalue<0.0000000001] = 0.0000000001


par(mfrow=c(1,1),mar=c(5,5,5,5))
plot(res_Ddx5_ctcf_Deseq2_RPGC$log2FoldChange,
     -log10(res_Ddx5_ctcf_Deseq2_RPGC$pvalue),
     col=res_Ddx5_ctcf_Deseq2_RPGC$col,pch=19, cex=0.25,
     ylab=expression(-Log[10] ~ (P-val.)),
     xlab=expression(Log[2] ~ (Ddx5/WT)), 
     axes=FALSE, xlim=c(-3,3),cex.lab=1.5 )
axis(1,lwd=2,cex.axis=1.5)
axis(2,lwd=2,cex.axis=1.5)
box(lwd=2, col='black')
points( x=res_Ddx5_ctcf_Deseq2_RPGC$log2FoldChange[res_Ddx5_ctcf_Deseq2_RPGC$col=="#049494"],
        y=-log10(res_Ddx5_ctcf_Deseq2_RPGC$pvalue[res_Ddx5_ctcf_Deseq2_RPGC$col=="#049494"]),
        col="#049494")
points( x=res_Ddx5_ctcf_Deseq2_RPGC$log2FoldChange[res_Ddx5_ctcf_Deseq2_RPGC$col=="#305494"],
        y=-log10(res_Ddx5_ctcf_Deseq2_RPGC$pvalue[res_Ddx5_ctcf_Deseq2_RPGC$col=="#305494"]),
        col="#305494")
```

```
table(res_Ddx5_ctcf_Deseq2_RPGC$col)
```

```
## 
## #049494 #305494  gray90 
##     932    1099   40201
```

```
df = data.frame( LFC=res_Ddx5_ctcf_Deseq2_RPGC$log2FoldChange,
                 P=res_Ddx5_ctcf_Deseq2_RPGC$log2FoldChange)
write.table(df,file=paste0(source_data_directory,"MA_ddx5KO.txt"),row.names = FALSE, sep="\t",quote=FALSE)
```

```
lost_peaks = rownames(res_Ddx5_ctcf_Deseq2_RPGC[res_Ddx5_ctcf_Deseq2_RPGC$padj<0.2 & res_Ddx5_ctcf_Deseq2_RPGC$log2FoldChange<0,])
gained_peaks = rownames(res_Ddx5_ctcf_Deseq2_RPGC[res_Ddx5_ctcf_Deseq2_RPGC$padj<0.2 & res_Ddx5_ctcf_Deseq2_RPGC$log2FoldChange>0,])

lost_Ddx5 = ctcf_wt_NS_peaks_resized[which(names(ctcf_wt_NS_peaks_resized) %in% lost_peaks)]
gained_Ddx5 = ctcf_wt_NS_peaks_resized[which(names(ctcf_wt_NS_peaks_resized) %in% gained_peaks)]

write.table( res_Ddx5_ctcf_Deseq2_RPGC,
             file=paste0(objects_directory,"res_Ddx5_ctcf_Deseq2_RPGC.txt"),
             sep='\t',quote=FALSE,row.names=FALSE,col.names=TRUE )
```

```
load(paste0(objects_directory,"CTCF_Ddx5ko_NS_A3_RPGC.RData"))
par(mfrow=c(1,2),mar=c(5,5,5,3))
plot( x=seq(-1000,1000,length.out=200),
      y=colMeans(a3_1_ctcf_rpgc_ctcf_AP[rownames(a3_1_ctcf_rpgc_ctcf_AP) %in% lost_peaks,]),
      ty="l", col="blue3", ylim=c(0,100), 
      ylab="RPGC", xlab="Distance from CTCF peak summit")
lines(x=seq(-1000,1000,length.out=200,lwd=2),
      y=colMeans(a3_2_ctcf_rpgc_ctcf_AP[rownames(a3_2_ctcf_rpgc_ctcf_AP) %in% lost_peaks,]),
      col="blue3",lwd=2 )
```

```
## Warning: W poleceniu 'seq.default(-1000, 1000, length.out = 200, lwd = 2)':
##  dodatkowy argument 'lwd' zostanie odrzucony
```

```
lines(x=seq(-1000,1000,length.out=200),
      y=colMeans(cb1_ctcf_rpgc_ctcf_AP[rownames(cb1_ctcf_rpgc_ctcf_AP) %in% lost_peaks,]),
      col="steelblue3",lwd=2 )
lines(x=seq(-1000,1000,length.out=200),
      y=colMeans(ce10_ctcf_rpgc_ctcf_AP[rownames(ce10_ctcf_rpgc_ctcf_AP) %in% lost_peaks,]),
      col="steelblue3",lwd=2 )


plot( x=seq(-1000,1000,length.out=200),
      y=colMeans(a3_1_ctcf_rpgc_ctcf_AP[rownames(a3_1_ctcf_rpgc_ctcf_AP) %in% gained_peaks,]),
      ty="l", col="blue3", ylim=c(0,100), ylab="RPGC", xlab="Distance from CTCF peak summit",lwd=2)
lines(x=seq(-1000,1000,length.out=200),
      y=colMeans(a3_2_ctcf_rpgc_ctcf_AP[rownames(a3_2_ctcf_rpgc_ctcf_AP) %in% gained_peaks,]),
      col="blue3",lwd=2 )
lines(x=seq(-1000,1000,length.out=200),
      y=colMeans(cb1_ctcf_rpgc_ctcf_AP[rownames(cb1_ctcf_rpgc_ctcf_AP) %in% gained_peaks,]),
      col="steelblue3",lwd=2 )
lines(x=seq(-1000,1000,length.out=200),
      y=colMeans(ce10_ctcf_rpgc_ctcf_AP[rownames(ce10_ctcf_rpgc_ctcf_AP) %in% gained_peaks,]),
      col="steelblue3",lwd=2 )
```

```
df = data.frame( location=seq(-1000,1000,length.out=200),
                 lost_wt1=colMeans(a3_1_ctcf_rpgc_ctcf_AP[rownames(a3_1_ctcf_rpgc_ctcf_AP) %in% lost_peaks,]),
                 lost_wt2=colMeans(a3_2_ctcf_rpgc_ctcf_AP[rownames(a3_2_ctcf_rpgc_ctcf_AP) %in% lost_peaks,]),
                 lost_cb1=colMeans(cb1_ctcf_rpgc_ctcf_AP[rownames(cb1_ctcf_rpgc_ctcf_AP) %in% lost_peaks,]),
                 lost_ce10=colMeans(ce10_ctcf_rpgc_ctcf_AP[rownames(ce10_ctcf_rpgc_ctcf_AP) %in% lost_peaks,]),
                 gained_wt1=colMeans(a3_1_ctcf_rpgc_ctcf_AP[rownames(a3_1_ctcf_rpgc_ctcf_AP) %in% gained_peaks,]),
                 gained_wt2=colMeans(a3_2_ctcf_rpgc_ctcf_AP[rownames(a3_2_ctcf_rpgc_ctcf_AP) %in% gained_peaks,]),
                 gained_cb1=colMeans(cb1_ctcf_rpgc_ctcf_AP[rownames(cb1_ctcf_rpgc_ctcf_AP) %in% gained_peaks,]),
                 gained_ce10=colMeans(ce10_ctcf_rpgc_ctcf_AP[rownames(ce10_ctcf_rpgc_ctcf_AP) %in% gained_peaks,]))
                 
write.table(df,file=paste0(source_data_directory,"AP_CTCF_wt_ddx5KO.txt"),row.names = FALSE, sep="\t",quote=FALSE)
```

```
DMSO1_ctcf_rpgc = import.bw(paste0(chipseq_directory,"ChIP_Seq_CTCF_11-24_MusMus_es-NPC_DDX5_FKBP_KI_CTCF-Cterm_HALO+DDX5-FKBP-KI_NPC_Ddx5-FKBP_KI_4F11_DMSO-Rep1_Rep_1_RPGC.bw"))
dTAG1_ctcf_rpgc = import.bw(paste0(chipseq_directory,"ChIP_Seq_CTCF_11-24_MusMus_es-NPC_DDX5_FKBP_KI_CTCF-Cterm_HALO+DDX5-FKBP-KI_NPC_Ddx5-FKBP_KI_4F11_dTAG13-Rep1_Rep_1_RPGC.bw"))
DMSO2_ctcf_rpgc = import.bw(paste0(chipseq_directory,"ChIP_Seq_CTCF_11-24_MusMus_es-NPC_DDX5_FKBP_KI_CTCF-Cterm_HALO+DDX5-FKBP-KI_NPC_Ddx5-FKBP_KI_4F11_DMSO-Rep2_Rep_1_RPGC.bw"))
dTAG2_ctcf_rpgc = import.bw(paste0(chipseq_directory,"ChIP_Seq_11-24_MusMus_es-NPC_DDX5_FKBP_KI_CTCF-Cterm_HALO+DDX5-FKBP-KI_NPC_Ddx5-FKBP_KI_4F11_dTAG13-Rep2_Rep_1_RPGC.bw"))
seqlevelsStyle(DMSO1_ctcf_rpgc) = "ucsc"
seqlevelsStyle(dTAG1_ctcf_rpgc) = "ucsc"
seqlevelsStyle(DMSO2_ctcf_rpgc) = "ucsc"
seqlevelsStyle(dTAG2_ctcf_rpgc) = "ucsc"


DMSO1_ctcf_rpgc_AP = getSignalInBins(ctcf_NS_ranges,DMSO1_ctcf_rpgc,1)
dTAG1_ctcf_rpgc_AP = getSignalInBins(ctcf_NS_ranges,dTAG1_ctcf_rpgc,1)
DMSO2_ctcf_rpgc_AP = getSignalInBins(ctcf_NS_ranges,DMSO2_ctcf_rpgc,1)
dTAG2_ctcf_rpgc_AP = getSignalInBins(ctcf_NS_ranges,dTAG2_ctcf_rpgc,1)
rownames(DMSO1_ctcf_rpgc_AP) = ctcf_wt_NS_peaks_summit$name
rownames(dTAG1_ctcf_rpgc_AP) = ctcf_wt_NS_peaks_summit$name
rownames(DMSO2_ctcf_rpgc_AP) = ctcf_wt_NS_peaks_summit$name
rownames(dTAG2_ctcf_rpgc_AP) = ctcf_wt_NS_peaks_summit$name

save(DMSO1_ctcf_rpgc_AP,
     dTAG1_ctcf_rpgc_AP,
     DMSO2_ctcf_rpgc_AP,
     dTAG2_ctcf_rpgc_AP,
     file=paste0(objects_directory,"CTCF_4F11_NS_RPGC.RData"))
```

```
load(paste0(objects_directory,"CTCF_4F11_NS_RPGC.RData"))
a=90;b=110
c4f11_ctcf = data.frame( dmso1 = ( rowSums(DMSO1_ctcf_rpgc_AP[,a:b])),
                         dmso2 = ( rowSums(DMSO2_ctcf_rpgc_AP[,a:b])),
                         dTAG1 = ( rowSums(dTAG1_ctcf_rpgc_AP[,a:b])),
                         dTAG2 = ( rowSums(dTAG2_ctcf_rpgc_AP[,a:b])),
                         row.names = rownames(DMSO1_ctcf_rpgc_AP))
```

Check with raw signal like Ddx5

```
load(paste0(objects_directory,'ctcf_NS_ranges.RData'))
DMSO1_ctcf_raw = import.bw(paste0(chipseq_directory,"ChIP_Seq_CTCF_11-24_MusMus_es-NPC_DDX5_FKBP_KI_CTCF-Cterm_HALO+DDX5-FKBP-KI_NPC_Ddx5-FKBP_KI_4F11_DMSO-Rep1_Rep_1_filtered_unnormalized.bw"))
dTAG1_ctcf_raw = import.bw(paste0(chipseq_directory,"ChIP_Seq_CTCF_11-24_MusMus_es-NPC_DDX5_FKBP_KI_CTCF-Cterm_HALO+DDX5-FKBP-KI_NPC_Ddx5-FKBP_KI_4F11_dTAG13-Rep1_Rep_1_filtered_unnormalized.bw"))
DMSO2_ctcf_raw = import.bw(paste0(chipseq_directory,"ChIP_Seq_CTCF_11-24_MusMus_es-NPC_DDX5_FKBP_KI_CTCF-Cterm_HALO+DDX5-FKBP-KI_NPC_Ddx5-FKBP_KI_4F11_DMSO-Rep2_Rep_1_filtered_unnormalized.bw"))
dTAG2_ctcf_raw = import.bw(paste0(chipseq_directory,"ChIP_Seq_11-24_MusMus_es-NPC_DDX5_FKBP_KI_CTCF-Cterm_HALO+DDX5-FKBP-KI_NPC_Ddx5-FKBP_KI_4F11_dTAG13-Rep2_Rep_1_filtered_unnormalized.bw"))
seqlevelsStyle(DMSO1_ctcf_raw) = "ucsc"
seqlevelsStyle(dTAG1_ctcf_raw) = "ucsc"
seqlevelsStyle(DMSO2_ctcf_raw) = "ucsc"
seqlevelsStyle(dTAG2_ctcf_raw) = "ucsc"

DMSO1_ctcf_raw_AP = getSignalInBins(ctcf_NS_ranges,DMSO1_ctcf_raw,1)
dTAG1_ctcf_raw_AP = getSignalInBins(ctcf_NS_ranges,dTAG1_ctcf_raw,1)
DMSO2_ctcf_raw_AP = getSignalInBins(ctcf_NS_ranges,DMSO2_ctcf_raw,1)
dTAG2_ctcf_raw_AP = getSignalInBins(ctcf_NS_ranges,dTAG2_ctcf_raw,1)
rownames(DMSO1_ctcf_raw_AP) = ctcf_wt_NS_peaks_summit$name
rownames(dTAG1_ctcf_raw_AP) = ctcf_wt_NS_peaks_summit$name
rownames(DMSO2_ctcf_raw_AP) = ctcf_wt_NS_peaks_summit$name
rownames(dTAG2_ctcf_raw_AP) = ctcf_wt_NS_peaks_summit$name

save(DMSO1_ctcf_raw_AP,
     dTAG1_ctcf_raw_AP,
     DMSO2_ctcf_raw_AP,
     dTAG2_ctcf_raw_AP,
     file=paste0(objects_directory,"CTCF_4F11_NS_RAW.RData"))

dTAGD_ctcf = data.frame( dmso1 = ( rowSums(DMSO1_ctcf_raw_AP[,a:b])),
                         dmso2 = ( rowSums(DMSO2_ctcf_raw_AP[,a:b])),
                         dtag1 = ( rowSums(dTAG1_ctcf_raw_AP[,a:b])),
                         dtag2 = ( rowSums(dTAG2_ctcf_raw_AP[,a:b])) )
metadata_dTAG = data.frame( treatment = c("dmso","dmso","dtag","dtag"),
                                  row.names = colnames(dTAGD_ctcf))


dTAG_ctcf_Deseq2 = DESeqDataSetFromMatrix(
 countData = dTAGD_ctcf,
 colData = metadata_dTAG,
 design = ~ treatment )
```

```
## converting counts to integer mode
```

```
## Warning in DESeqDataSet(se, design = design, ignoreRank): some variables in
## design formula are characters, converting to factors
```

```
dTAG_ctcf_Deseq2 = DESeq(dTAG_ctcf_Deseq2, fitType = 'local')
```

```
## estimating size factors
```

```
## estimating dispersions
```

```
## gene-wise dispersion estimates
```

```
## mean-dispersion relationship
```

```
## final dispersion estimates
```

```
## fitting model and testing
```

```
res_dTAG_ctcf_Deseq2 = results(dTAG_ctcf_Deseq2, 
                              contrast = c("treatment", "dtag", "dmso"))
summary( res_dTAG_ctcf_Deseq2 )
```

```
## 
## out of 42232 with nonzero total read count
## adjusted p-value < 0.1
## LFC > 0 (up)       : 287, 0.68%
## LFC < 0 (down)     : 256, 0.61%
## outliers [1]       : 0, 0%
## low counts [2]     : 0, 0%
## (mean count < 0)
## [1] see 'cooksCutoff' argument of ?results
## [2] see 'independentFiltering' argument of ?results
```

```
dtag_normalized = counts( dTAG_ctcf_Deseq2,normalized=TRUE )
```

```
heatscatter( x=res_dTAG_ctcf_Deseq2$baseMean,
             y=res_dTAG_ctcf_Deseq2$log2FoldChange, 
             pch=19, cex=0.1, add.contour = T, 
             xlim=c(0,1200),ylim=c(-2,2),
             xlab=expression(Log[2] ~ (Signal)),
             ylab=expression(Log[2] ~ (LFC)), 
             main="dTAG13/DMSO")
abline( h=0 )
axis(1,lwd=2)
axis(2,lwd=2)
box(col="black",lwd=2)
```

```
df = data.frame( M=res_dTAG_ctcf_Deseq2$baseMean,A=res_dTAG_ctcf_Deseq2$log2FoldChange)
write.table(df,file=paste0(source_data_directory,"MA_dTAG.txt"),row.names = FALSE, sep="\t",quote=FALSE)
```

Peaks lost/gained upon dTAG13 and in genetic loss of Ddx5

```
gained_all = which(1.25*c4f11_ctcf$dmso1<c4f11_ctcf$dTAG1 & 1.25*c4f11_ctcf$dmso2<c4f11_ctcf$dTAG2 & res_Ddx5_ctcf_Deseq2_RPGC$log2FoldChange>0 & res_Ddx5_ctcf_Deseq2_RPGC$padj<0.25 )
lost_all = which(c4f11_ctcf$dmso1>1.25*c4f11_ctcf$dTAG1 & c4f11_ctcf$dmso2<1.25*c4f11_ctcf$dTAG2 & res_Ddx5_ctcf_Deseq2_RPGC$log2FoldChange<0 & res_Ddx5_ctcf_Deseq2_RPGC$padj<0.25 )
length(gained_all)
```

```
## [1] 124
```

```
length(lost_all)
```

```
## [1] 251
```

```
gained_4F11 = rownames(c4f11_ctcf)[which(1.25*c4f11_ctcf$dmso1<c4f11_ctcf$dTAG1 & 1.25*c4f11_ctcf$dmso2<c4f11_ctcf$dTAG2  )]
lost_4F11 = rownames(c4f11_ctcf)[which(c4f11_ctcf$dmso1>1.25*c4f11_ctcf$dTAG1 & c4f11_ctcf$dmso2<1.25*c4f11_ctcf$dTAG2   )]
length(gained_4F11)
```

```
## [1] 4581
```

```
length(lost_4F11)
```

```
## [1] 8022
```

```
gained_4F11_id = which(1.25*c4f11_ctcf$dmso1<c4f11_ctcf$dTAG1 & 1.25*c4f11_ctcf$dmso2<c4f11_ctcf$dTAG2  )
lost_4F11_id = which(c4f11_ctcf$dmso1>1.25*c4f11_ctcf$dTAG1 & c4f11_ctcf$dmso2<1.25*c4f11_ctcf$dTAG2   )
length(gained_4F11_id)
```

```
## [1] 4581
```

```
length(lost_4F11_id)
```

```
## [1] 8022
```

```
acute_loss_ctcf = ctcf_wt_NS_peaks_resized[which(ctcf_wt_NS_peaks_resized$name %in% lost_4F11 )]
acute_gain_ctcf = ctcf_wt_NS_peaks_resized[which(ctcf_wt_NS_peaks_resized$name %in% gained_4F11 )]

export.bed(ctcf_NS_ranges[gained_all], 
           con = paste0( data_directory,"gained_all.bed" ) )
export.bed(ctcf_NS_ranges[lost_all], 
           con = paste0( data_directory,"lost_all.bed" ) )
```

Coordinates of gained and lost peaks for further analyses.

```
lost_Ddx5 = ctcf_wt_NS_peaks_resized[lost_all]
gained_Ddx5 = ctcf_wt_NS_peaks_resized[ gained_all ]

lost_ctcf_sequence = Biostrings::getSeq(BSgenome.Mmusculus.UCSC.mm10,lost_Ddx5)
```

```
## Warning in .Seqinfo.mergexy(x, y): Each of the 2 combined objects has sequence levels not in the other:
##   - in 'x': chr1_GL456210_random, chr1_GL456211_random, chr1_GL456212_random, chr1_GL456213_random, chr1_GL456221_random, chr4_GL456216_random, chr4_GL456350_random, chr4_JH584292_random, chr4_JH584293_random, chr4_JH584294_random, chr4_JH584295_random, chr5_GL456354_random, chr5_JH584296_random, chr5_JH584297_random, chr5_JH584298_random, chr5_JH584299_random, chr7_GL456219_random, chrX_GL456233_random, chrY_JH584300_random, chrY_JH584301_random, chrY_JH584302_random, chrY_JH584303_random, chrUn_GL456239, chrUn_GL456359, chrUn_GL456360, chrUn_GL456366, chrUn_GL456367, chrUn_GL456368, chrUn_GL456370, chrUn_GL456372, chrUn_GL456378, chrUn_GL456379, chrUn_GL456381, chrUn_GL456382, chrUn_GL456383, chrUn_GL456385, chrUn_GL456387, chrUn_GL456389, chrUn_GL456390, chrUn_GL456392, chrUn_GL456393, chrUn_GL456394, chrUn_GL456396, chrUn_JH584304
##   - in 'y': GL456210.1, GL456211.1, GL456212.1, GL456216.1, GL456221.1, GL456233.1, GL456350.1, GL456354.1, GL456359.1, GL456366.1, GL456367.1, GL456368.1, GL456370.1, GL456372.1, GL456378.1, GL456383.1, GL456385.1, GL456389.1, GL456390.1, GL456392.1, GL456394.1, GL456396.1, JH584293.1, JH584294.1, JH584299.1, JH584304.1
##   Make sure to always combine/compare objects based on the same reference
##   genome (use suppressWarnings() to suppress this warning).
```

```
gained_ctcf_sequence = Biostrings::getSeq(BSgenome.Mmusculus.UCSC.mm10,gained_Ddx5)
```

```
## Warning in .Seqinfo.mergexy(x, y): Each of the 2 combined objects has sequence levels not in the other:
##   - in 'x': chr1_GL456210_random, chr1_GL456211_random, chr1_GL456212_random, chr1_GL456213_random, chr1_GL456221_random, chr4_GL456216_random, chr4_GL456350_random, chr4_JH584292_random, chr4_JH584293_random, chr4_JH584294_random, chr4_JH584295_random, chr5_GL456354_random, chr5_JH584296_random, chr5_JH584297_random, chr5_JH584298_random, chr5_JH584299_random, chr7_GL456219_random, chrX_GL456233_random, chrY_JH584300_random, chrY_JH584301_random, chrY_JH584302_random, chrY_JH584303_random, chrUn_GL456239, chrUn_GL456359, chrUn_GL456360, chrUn_GL456366, chrUn_GL456367, chrUn_GL456368, chrUn_GL456370, chrUn_GL456372, chrUn_GL456378, chrUn_GL456379, chrUn_GL456381, chrUn_GL456382, chrUn_GL456383, chrUn_GL456385, chrUn_GL456387, chrUn_GL456389, chrUn_GL456390, chrUn_GL456392, chrUn_GL456393, chrUn_GL456394, chrUn_GL456396, chrUn_JH584304
##   - in 'y': GL456210.1, GL456211.1, GL456212.1, GL456216.1, GL456221.1, GL456233.1, GL456350.1, GL456354.1, GL456359.1, GL456366.1, GL456367.1, GL456368.1, GL456370.1, GL456372.1, GL456378.1, GL456383.1, GL456385.1, GL456389.1, GL456390.1, GL456392.1, GL456394.1, GL456396.1, JH584293.1, JH584294.1, JH584299.1, JH584304.1
##   Make sure to always combine/compare objects based on the same reference
##   genome (use suppressWarnings() to suppress this warning).
```

```
write.table( c4f11_ctcf,file=paste0(data_directory,"c4f11_ctcf.txt"),
             sep='\t',quote=FALSE,row.names=FALSE,col.names=TRUE )
write.table( elbat_ctcf,file=paste0(data_directory,"elbat_ctcf.txt"),
             sep='\t',quote=FALSE,row.names=FALSE,col.names=TRUE )

write.table( res_Ddx5_ctcf_Deseq2_RPGC,
             file=paste0(data_directory,"res_Ddx5_ctcf_Deseq2_RPGC.txt"),
             sep='\t',quote=FALSE,row.names=FALSE,col.names=TRUE )

write.table( res_dTAG_ctcf_Deseq2,file=paste0(data_directory,"res_dTAG_ctcf_Deseq2.txt"),
             sep='\t',quote=FALSE,row.names=FALSE,col.names=TRUE )
```

#### Scatterplot in Extended Figure

```
ctcf_wt_ES_peaks = import.bed(paste0(chipseq_directory,'ChIP_Seq_CTCF_07-22_MusMus_ESC_MOD_CTCF-Cterm_HALO_Control_merged_summits.bed'))
seqlevelsStyle(ctcf_wt_ES_peaks)="ucsc"
  
ctcf_wt_ES_Halo_ranges = do.call('rbind', lapply( as.list(seq(1:length(ctcf_wt_ES_peaks))), function(i){
  g = ctcf_wt_ES_peaks[i]
  tss = start(g)
  chr = as.character( chrom(g) )
  peak=i
      return( data.frame( chr = rep( (chr), 200),
                          starts = seq( tss-1000, tss+990, length.out=200),
                          ends =  seq( tss-1000, tss+990, length.out=200)+9,
                          peak = rep( peak, 200) ) ) } ) )


ctcf_wt_ES_Halo_ranges = GRanges(seqnames = Rle(ctcf_wt_ES_Halo_ranges$chr),
                               ranges = IRanges(as.numeric(ctcf_wt_ES_Halo_ranges$starts),
                                                end = as.numeric(ctcf_wt_ES_Halo_ranges$ends),
                                                names = seq(1, nrow(ctcf_wt_ES_Halo_ranges))),
                               strand = Rle(rep("*", nrow(ctcf_wt_ES_Halo_ranges))),
                               peak = ctcf_wt_ES_Halo_ranges$peak )
seqlevelsStyle(ctcf_wt_ES_Halo_ranges) = 'ucsc'
save(ctcf_wt_ES_Halo_ranges,file=paste0(objects_directory,'ctcf_wt_ES_Halo_ranges.RData'))


es_cterm_wt_ctcf1 = import.bw(paste0(chipseq_directory,"ChIP_Seq_CTCF_07-22_MusMus_ESC_MOD_CTCF-Cterm_HALO_Control_Rep_1_RPGC.bw"))
es_cterm_wt_ctcf2 = import.bw(paste0(chipseq_directory,"ChIP_Seq_CTCF_07-22_MusMus_ESC_MOD_CTCF-Cterm_HALO_Control_Rep_2_RPGC.bw"))

es_cterm_cb1_ctcf = import.bw(paste0(chipseq_directory,"ChIP_Seq_CTCF_07-22_MusMus_ESC_DDX5_KO_CTCF-Cterm_HALO_CB1_Rep_1_RPGC.bw"))
es_cterm_ce10_ctcf = import.bw(paste0(chipseq_directory,"ChIP_Seq_CTCF_07-22_MusMus_ESC_DDX5_KO_CTCF-Cterm_HALO_CE10_Rep_1_RPGC.bw"))
seqlevelsStyle(es_cterm_wt_ctcf1) = "ucsc"
seqlevelsStyle(es_cterm_wt_ctcf2) = "ucsc"
seqlevelsStyle(es_cterm_cb1_ctcf) = "ucsc"
seqlevelsStyle(es_cterm_ce10_ctcf) = "ucsc"


es_cterm_wt_ctcf_AP1 = getSignalInBins(ctcf_wt_ES_Halo_ranges,es_cterm_wt_ctcf1,1)
es_cterm_wt_ctcf_AP2 = getSignalInBins(ctcf_wt_ES_Halo_ranges,es_cterm_wt_ctcf2,1)

es_cterm_cb1_ctcf_AP = getSignalInBins(ctcf_wt_ES_Halo_ranges,es_cterm_cb1_ctcf,1)
es_cterm_ce10_ctcf_AP = getSignalInBins(ctcf_wt_ES_Halo_ranges,es_cterm_ce10_ctcf,1)

save(es_cterm_wt_ctcf_AP1,es_cterm_wt_ctcf_AP2,
     es_cterm_cb1_ctcf_AP,es_cterm_ce10_ctcf_AP,
     file=paste0(outputs_directory,"CTCF_Ddx5ko_ES.RData"))
```

Plot

```
load(paste0(outputs_directory,"CTCF_Ddx5ko_ES.RData"))
load(paste0(objects_directory,"CTCF_Ddx5ko_NS_A3_RPGC.RData"))

es_cterm_ddx5_wt = (es_cterm_wt_ctcf_AP1+es_cterm_wt_ctcf_AP2)/2
es_cterm_ddx5_ko = (es_cterm_ce10_ctcf_AP+es_cterm_cb1_ctcf_AP)/2
ns_cterm_ddx5_wt = (a3_1_ctcf_rpgc_ctcf_AP+a3_2_ctcf_rpgc_ctcf_AP)/2
ns_cterm_ddx5_ko = (cb1_ctcf_rpgc_ctcf_AP+ce10_ctcf_rpgc_ctcf_AP)/2

boxplot(log10(rowSums(es_cterm_ddx5_ko[,95:105]))-log10(rowSums(es_cterm_ddx5_wt[,95:105])),
        log10(rowSums(ns_cterm_ddx5_ko[,95:105]))-log10(rowSums(ns_cterm_ddx5_wt[,95:105])),
        outline=FALSE, border=c("red","blue"),col="white",names=c("ES","NS") )
axis(1,lwd=2,at=c(1,2),c("ES","NS"))
axis(2,lwd=2)
box(col="black",lwd=2)
```

```
N = max(c(nrow(es_cterm_ddx5_ko),nrow(ns_cterm_ddx5_ko)))
df = data.frame( ES = rep(NA, N), NS=rep(NA,N))
df$ES[1:nrow(es_cterm_ddx5_ko)] = log10(rowSums(es_cterm_ddx5_ko[,95:105]))-log10(rowSums(es_cterm_ddx5_wt[,95:105]))
df$NS[1:nrow(ns_cterm_ddx5_ko)] = log10(rowSums(ns_cterm_ddx5_ko[,95:105]))-log10(rowSums(ns_cterm_ddx5_wt[,95:105]))

write.table(df,file=paste0(source_data_directory,"boxplot_CTCF_level.txt"),row.names = FALSE, sep="\t",quote=FALSE)
```

## Motif search

In 500bp window centered at the peak summit.

```
library(motifbreakR)
```

```
## Ładowanie wymaganego pakietu: MotifDb
```

```
## See system.file("LICENSE", package="MotifDb") for use restrictions.
```

```
## Warning: zastępowanie poprzedniego importu 'S4Vectors::as.data.frame' przez
## 'motifStack::as.data.frame' podczas ładowanie przestrzeni nazw 'motifbreakR'
```

```
## 
## Dołączanie pakietu: 'motifbreakR'
```

```
## Następujący obiekt został zakryty z 'package:LSD':
## 
##     homer
```

```
data(hocomoco)

findAllHOCOMOCO = function( this_seq, hcdb ){
  # this_seq = lost_ctcf_sequence[[10]]; hcdb = hocomoco; i=1
  do.call("c",lapply(as.list(seq(1,length(hcdb))), function(x){
    countPWM( hcdb[[x]], this_seq, min.score="80%" )
  }))
}

lost_ctcf_motif = do.call("rbind",lapply(as.list(1:length(lost_ctcf_sequence)),function(x){findAllHOCOMOCO(lost_ctcf_sequence[[x]],hocomoco)}))
gained_ctcf_motif = do.call("rbind",lapply(as.list(1:length(gained_ctcf_sequence)),function(x){findAllHOCOMOCO(gained_ctcf_sequence[[x]],hocomoco)}))
```

```
lost_tfs_freq = colMeans(lost_ctcf_motif>0)
gained_tfs_freq = colMeans(gained_ctcf_motif>0)

lost_tfs_N = colSums(lost_ctcf_motif>0)
gained_tfs_N = colSums(gained_ctcf_motif>0)

res_Fisher = do.call("rbind",lapply(as.list(1:length(lost_tfs_N)), function(x){
  m = matrix(c(lost_tfs_N[x],nrow(lost_ctcf_motif),
               gained_tfs_N[x],nrow(gained_ctcf_motif)),2,2)
  m = fisher.test(m)
  return( data.frame(pval = round(m$p.value,5), est = m$estimate) )
}))
res_Fisher$padj = fdrtool::fdrtool( res_Fisher$pval, statistic="pvalue", plot=FALSE )$qval
```

```
## Step 1... determine cutoff point
## Step 2... estimate parameters of null distribution and eta0
## Step 3... compute p-values and estimate empirical PDF/CDF
## Step 4... compute q-values and local fdr
```

```
rownames(res_Fisher) = names(hocomoco)

colVector = rep( "gray", nrow(res_Fisher) )
colVector[res_Fisher$padj<0.1 & res_Fisher$est>1.25] = "purple4"
colVector[res_Fisher$padj<0.1 & res_Fisher$est<1/1.25] = "orange3"

extract_TF = function(x){
  tp = unlist(strsplit( x,"Hsapiens-HOCOMOCO-"))
  tp = tp[seq(2,length(tp),by=2)]
  tp = unlist(strsplit( tp,"_"))
  return( tp[seq(1,length(tp),by=2)] )
}

rich_Lost = extract_TF(rownames(res_Fisher[colVector=="purple4",]))
rich_Gained = extract_TF(rownames(res_Fisher[colVector=="orange3",]))

par(pty="s",mar=c(4,4,4,4),mfrow=c(1,1))
plot( x=lost_tfs_freq, 
      y=gained_tfs_freq,
      xlim=c(0,1),ylim=c(0,1),
      xlab="Lost",ylab="Gained",
      pch=19, cex=0.75,
      col=colVector )
abline(a=0,b=1,col="gray",lwd=2)
axis(1,lwd=2)
axis(2,lwd=2)
box(col="black",lwd=2)
text( x=lost_tfs_freq[colVector=="purple4"]+0.013, 
      y=gained_tfs_freq[colVector=="purple4"]-0.013, 
      rich_Lost,cex=0.4)
text( x=lost_tfs_freq[colVector=="orange3"]+0.013, 
      y=gained_tfs_freq[colVector=="orange3"]-0.013, 
      rich_Gained,cex=0.4)
```

```
write.table( res_Fisher,file=paste0(data_directory,"res_Fisher.txt"),
             sep='\t',quote=FALSE,row.names=FALSE,col.names=TRUE )
```

## Factors enriched at CTCF sites losing CTCF signal in Ddx5-/- NS cells

MAZ is an example. This does not make any sense. Ddx5 loss should stabilise G4, these sould fister CTCF binding… We see that loss of CTCF is observed at sites prone to forming G4 quadruplexes.

```
rich_Lost
```

```
##  [1] "AP2A"  "AP2B"  "AP2C"  "AP2D"  "ARNT2" "ARNT"  "ATF6A" "BHE41" "CTCF" 
## [10] "E2F2"  "E2F3"  "E2F4"  "EGR1"  "EGR2"  "EGR4"  "ELK1"  "GABPA" "GLIS3"
## [19] "HES1"  "HESX1" "HIF1A" "HTF4"  "INSM1" "KLF1"  "KLF4"  "KLF6"  "MAZ"  
## [28] "MBD2"  "MECP2" "MYOG"  "NFKB1" "NR0B1" "NRF1"  "P73"   "PLAG1" "PLAL1"
## [37] "SP1"   "SP1"   "SP2"   "SP3"   "STAT3" "WT1"   "ZBT7A" "ZFX"   "ZIC1" 
## [46] "ZIC3"  "ZN148"
```

## Factors enriched at CTCF sites gaining CTCF signal in Ddx5-/- NS cells

```
rich_Gained
```

```
##  [1] "ALX1"  "ARI3A" "ARI3A" "BARX2" "DLX2"  "DLX3"  "EVI1"  "FOXP3" "GATA5"
## [10] "HNF6"  "HXA10" "HXA5"  "HXB6"  "HXB7"  "HXB8"  "HXD13" "IRF4"  "LHX3" 
## [19] "MEF2A" "MEF2D" "NFIL3" "NKX31" "PBX1"  "PDX1"  "PIT1"  "PO2F1" "PO3F2"
## [28] "PO4F2" "SOX2"  "TAL1"  "TBP"   "THB"   "ZFHX3"
```

## CTCF motif strength

```
par(mfrow=c(1,1),mar=c(5,5,1,1),pty="m")
boxplot( ctcf_wt_NS_peaks[lost_all]$motif_score,
         ctcf_wt_NS_peaks[gained_all]$motif_score, 
         outline=FALSE, col="white",border=c("purple4","orange3"),
         names=c("lost","gained"),xlab="CTCF peaks",lwd=2, 
         ylab="CTCF motif score" )
t.test( ctcf_wt_NS_peaks[lost_all]$motif_score, ctcf_wt_NS_peaks[gained_all]$motif_score ) # a significant difference
```

```
## 
##  Welch Two Sample t-test
## 
## data:  ctcf_wt_NS_peaks[lost_all]$motif_score and ctcf_wt_NS_peaks[gained_all]$motif_score
## t = 6.7954, df = 225.54, p-value = 9.517e-11
## alternative hypothesis: true difference in means is not equal to 0
## 95 percent confidence interval:
##  3.533429 6.419644
## sample estimates:
## mean of x mean of y 
##  15.77321  10.79667
```

```
axis(1,lwd=2,at=c(1,2),c("lost","gained"))
axis(2,lwd=2)
box(col="black",lwd=2)
```

```
theNtimes = max(c(length(lost_all),length(gained_all)))
df = data.frame( lost_score = rep(NA,theNtimes), 
                 gained_score = rep(NA,theNtimes) )
df$lost_score[1:length(lost_all)] = ctcf_wt_NS_peaks[lost_all]$motif_score
df$gained_score[1:length(gained_all)] = ctcf_wt_NS_peaks[gained_all]$motif_score
write.table(df,file=paste0(source_data_directory,"boxplots_CTCF_motif_strength.txt"),row.names = FALSE, sep="\t",quote=FALSE)
```

## G4

```
library(ChIPseeker)
```

```
## Registered S3 methods overwritten by 'treeio':
##   method              from    
##   MRCA.phylo          tidytree
##   MRCA.treedata       tidytree
##   Nnode.treedata      tidytree
##   Ntip.treedata       tidytree
##   ancestor.phylo      tidytree
##   ancestor.treedata   tidytree
##   child.phylo         tidytree
##   child.treedata      tidytree
##   full_join.phylo     tidytree
##   full_join.treedata  tidytree
##   groupClade.phylo    tidytree
##   groupClade.treedata tidytree
##   groupOTU.phylo      tidytree
##   groupOTU.treedata   tidytree
##   is.rooted.treedata  tidytree
##   nodeid.phylo        tidytree
##   nodeid.treedata     tidytree
##   nodelab.phylo       tidytree
##   nodelab.treedata    tidytree
##   offspring.phylo     tidytree
##   offspring.treedata  tidytree
##   parent.phylo        tidytree
##   parent.treedata     tidytree
##   root.treedata       tidytree
##   rootnode.phylo      tidytree
##   sibling.phylo       tidytree
```

```
## Registered S3 method overwritten by 'ggtree':
##   method      from 
##   identify.gg ggfun
```

```
## ChIPseeker v1.28.3  For help: https://guangchuangyu.github.io/software/ChIPseeker
## 
## If you use ChIPseeker in published research, please cite:
## Guangchuang Yu, Li-Gen Wang, Qing-Yu He. ChIPseeker: an R/Bioconductor package for ChIP peak annotation, comparison and visualization. Bioinformatics 2015, 31(14):2382-2383
```

```
## 
## Dołączanie pakietu: 'ChIPseeker'
```

```
## Następujący obiekt został zakryty z 'package:ggVennDiagram':
## 
##     overlap
```

```
library(sigminer)
```

```
## Registered S3 methods overwritten by 'registry':
##   method               from 
##   print.registry_field proxy
##   print.registry_entry proxy
```

```
## Registered S3 method overwritten by 'sigminer':
##   method      from
##   print.bytes Rcpp
```

```
## sigminer version 2.3.1
## - Star me at https://github.com/ShixiangWang/sigminer
## - Run hello() to see usage and citation.
```

```
library(pqsfinder)

lost_Ddx5 = lost_Ddx5[which(chrom(lost_Ddx5) %in% paste0("chr",c(1:19,"X")))]
gained_Ddx5 = gained_Ddx5[which(chrom(gained_Ddx5) %in% paste0("chr",c(1:19,"X")))]

lost_ctcf_sequence = Biostrings::getSeq(BSgenome.Mmusculus.UCSC.mm10,lost_Ddx5)
```

```
## Warning in .Seqinfo.mergexy(x, y): Each of the 2 combined objects has sequence levels not in the other:
##   - in 'x': chr1_GL456210_random, chr1_GL456211_random, chr1_GL456212_random, chr1_GL456213_random, chr1_GL456221_random, chr4_GL456216_random, chr4_GL456350_random, chr4_JH584292_random, chr4_JH584293_random, chr4_JH584294_random, chr4_JH584295_random, chr5_GL456354_random, chr5_JH584296_random, chr5_JH584297_random, chr5_JH584298_random, chr5_JH584299_random, chr7_GL456219_random, chrX_GL456233_random, chrY_JH584300_random, chrY_JH584301_random, chrY_JH584302_random, chrY_JH584303_random, chrUn_GL456239, chrUn_GL456359, chrUn_GL456360, chrUn_GL456366, chrUn_GL456367, chrUn_GL456368, chrUn_GL456370, chrUn_GL456372, chrUn_GL456378, chrUn_GL456379, chrUn_GL456381, chrUn_GL456382, chrUn_GL456383, chrUn_GL456385, chrUn_GL456387, chrUn_GL456389, chrUn_GL456390, chrUn_GL456392, chrUn_GL456393, chrUn_GL456394, chrUn_GL456396, chrUn_JH584304
##   - in 'y': GL456210.1, GL456211.1, GL456212.1, GL456216.1, GL456221.1, GL456233.1, GL456350.1, GL456354.1, GL456359.1, GL456366.1, GL456367.1, GL456368.1, GL456370.1, GL456372.1, GL456378.1, GL456383.1, GL456385.1, GL456389.1, GL456390.1, GL456392.1, GL456394.1, GL456396.1, JH584293.1, JH584294.1, JH584299.1, JH584304.1
##   Make sure to always combine/compare objects based on the same reference
##   genome (use suppressWarnings() to suppress this warning).
```

```
gained_ctcf_sequence = Biostrings::getSeq(BSgenome.Mmusculus.UCSC.mm10,gained_Ddx5)
```

```
## Warning in .Seqinfo.mergexy(x, y): Each of the 2 combined objects has sequence levels not in the other:
##   - in 'x': chr1_GL456210_random, chr1_GL456211_random, chr1_GL456212_random, chr1_GL456213_random, chr1_GL456221_random, chr4_GL456216_random, chr4_GL456350_random, chr4_JH584292_random, chr4_JH584293_random, chr4_JH584294_random, chr4_JH584295_random, chr5_GL456354_random, chr5_JH584296_random, chr5_JH584297_random, chr5_JH584298_random, chr5_JH584299_random, chr7_GL456219_random, chrX_GL456233_random, chrY_JH584300_random, chrY_JH584301_random, chrY_JH584302_random, chrY_JH584303_random, chrUn_GL456239, chrUn_GL456359, chrUn_GL456360, chrUn_GL456366, chrUn_GL456367, chrUn_GL456368, chrUn_GL456370, chrUn_GL456372, chrUn_GL456378, chrUn_GL456379, chrUn_GL456381, chrUn_GL456382, chrUn_GL456383, chrUn_GL456385, chrUn_GL456387, chrUn_GL456389, chrUn_GL456390, chrUn_GL456392, chrUn_GL456393, chrUn_GL456394, chrUn_GL456396, chrUn_JH584304
##   - in 'y': GL456210.1, GL456211.1, GL456212.1, GL456216.1, GL456221.1, GL456233.1, GL456350.1, GL456354.1, GL456359.1, GL456366.1, GL456367.1, GL456368.1, GL456370.1, GL456372.1, GL456378.1, GL456383.1, GL456385.1, GL456389.1, GL456390.1, GL456392.1, GL456394.1, GL456396.1, JH584293.1, JH584294.1, JH584299.1, JH584304.1
##   Make sure to always combine/compare objects based on the same reference
##   genome (use suppressWarnings() to suppress this warning).
```

```
CpG = DNAString("CG")
lost_CpG = vcountPattern(CpG, lost_ctcf_sequence)
gained_CpG = vcountPattern(CpG, gained_ctcf_sequence)

par(mfrow=c(1,1),mar=c(5,5,1,1),pty="m")
boxplot( lost_CpG, 
         gained_CpG, 
         outline=FALSE, col="white",border=c("purple4","orange3"),
         names=c("lost","gained"),xlab="CTCF peaks",lwd=2, 
         ylab="number of CpGs/peak" )
t.test( lost_CpG, gained_CpG ) # a significant difference
```

```
## 
##  Welch Two Sample t-test
## 
## data:  lost_CpG and gained_CpG
## t = 11.237, df = 369.69, p-value < 2.2e-16
## alternative hypothesis: true difference in means is not equal to 0
## 95 percent confidence interval:
##  12.87545 18.33777
## sample estimates:
## mean of x mean of y 
## 21.187251  5.580645
```

```
axis(1,lwd=2,at=c(1,2),c("lost","gained"))
axis(2,lwd=2)
box(col="black",lwd=2)
```

```
theNtimes = max(c(length(lost_all),length(gained_all)))
df = data.frame( lost_score = rep(NA,theNtimes), 
                 gained_score = rep(NA,theNtimes) )
df$lost_score[1:length(lost_all)] = lost_CpG
df$gained_score[1:length(gained_all)] = gained_CpG
write.table(df,file=paste0(source_data_directory,"boxplots_CpG_motif_strength.txt"),row.names = FALSE, sep="\t",quote=FALSE)
```

```
lost_ctcf_sequence_G4 = lapply( as.list(1:length(lost_ctcf_sequence)), 
                                     function(le){
                                       pqsfinder(lost_ctcf_sequence[le][[1]], 
                                                 max_defects = 0, 
                                                 min_score = 20)
                                       })
gained_ctcf_sequence_G4 = lapply( as.list(1:length(gained_ctcf_sequence)), 
                                      function(le){
                                       
                                       pqsfinder(gained_ctcf_sequence[le][[1]], 
                                                 max_defects = 0,
                                                 min_score = 20)})

save( lost_ctcf_sequence_G4,gained_ctcf_sequence_G4,
      file=paste0(objects_directory,"G4_analysis.RData"))
```

```
load(paste0(objects_directory,"G4_analysis.RData"))
lost_G4_general = unlist(lapply(lost_ctcf_sequence_G4,function(x){length(score(x))}))
gained_G4_general = unlist(lapply(gained_ctcf_sequence_G4,function(x){length(score(x))}))


par(mfrow=c(1,1),mar=c(5,5,1,1),pty="m")
boxplot( lost_G4_general, 
         gained_G4_general, 
         outline=FALSE, col="white",border=c("purple4","orange3"),
         names=c("lost","gained"),xlab="CTCF peaks",lwd=2, 
         ylab="number of putative G4" )
t.test( lost_G4_general, gained_G4_general ) # a significant difference
```

```
## 
##  Welch Two Sample t-test
## 
## data:  lost_G4_general and gained_G4_general
## t = 8.5811, df = 360.38, p-value = 2.842e-16
## alternative hypothesis: true difference in means is not equal to 0
## 95 percent confidence interval:
##  1.748968 2.788945
## sample estimates:
## mean of x mean of y 
##  4.099602  1.830645
```

```
axis(1,lwd=2,at=c(1,2),c("lost","gained"))
axis(2,lwd=2)
box(col="black",lwd=2)
```

```
theNtimes = max(c(length(lost_all),length(gained_all)))
df = data.frame( lost_score = rep(NA,theNtimes), 
                 gained_score = rep(NA,theNtimes) )
df$lost_score[1:length(lost_all)] = lost_G4_general
df$gained_score[1:length(gained_all)] = gained_G4_general
write.table(df,file=paste0(source_data_directory,"boxplots_number_G4.txt"),row.names = FALSE, sep="\t",quote=FALSE)
```

```
load(paste0(objects_directory,"G4_analysis.RData"))
extractMaxScore = function( sr ){
  tp=score(sr)
  if(length(tp)>0) { 
    tp=tp[is.finite(tp)]
    return( max(tp) )}
}
lost_G4_general = unlist(lapply(lost_ctcf_sequence_G4,extractMaxScore))
gained_G4_general = unlist(lapply(gained_ctcf_sequence_G4,extractMaxScore))


par(mfrow=c(1,1),mar=c(5,5,1,1),pty="m")
boxplot( lost_G4_general, 
         gained_G4_general, 
         outline=FALSE, col="white",border=c("purple4","orange3"),
         names=c("lost","gained"),xlab="CTCF peaks",lwd=2, 
         ylab="Max G4 score" )
t.test( lost_G4_general, gained_G4_general ) # a significant difference
```

```
## 
##  Welch Two Sample t-test
## 
## data:  lost_G4_general and gained_G4_general
## t = 3.2901, df = 228.44, p-value = 0.00116
## alternative hypothesis: true difference in means is not equal to 0
## 95 percent confidence interval:
##   3.460778 13.795382
## sample estimates:
## mean of x mean of y 
##  47.77391  39.14583
```

```
axis(1,lwd=2,at=c(1,2),c("lost","gained"))
axis(2,lwd=2)
box(col="black",lwd=2)
```

```
theNtimes = max(c(length(lost_all),length(gained_all)))
df = data.frame( lost_score = rep(NA,theNtimes), 
                 gained_score = rep(NA,theNtimes) )
df$lost_score[1:length(lost_G4_general)] = lost_G4_general
df$gained_score[1:length(gained_G4_general)] = gained_G4_general
write.table(df,file=paste0(source_data_directory,"boxplots_max_G4_score.txt"),row.names = FALSE, sep="\t",quote=FALSE)
```

```
load(paste0(objects_directory,"all_CTCF_sequence_G4.RData"))
## average profile of G4
getAPvector_G4 = function(G5res,peak_width, scoreThr){
  # G5res=all_CTCF_sequence_G4[[4]];peak_width=1000;scoreThr=20
  tpgr = IRanges(seq(1,peak_width,by=5),seq(5,peak_width,by=5))
  theser = G5res@ranges[which(G5res@elementMetadata$score>scoreThr)]
  if(length(theser)>0){res =  countOverlaps(tpgr,theser)}
  if(length(theser)==0){res =  rep(0, length(tpgr))}
  return(res)
}

G4_all_CTCF_AP = do.call("rbind",lapply(all_CTCF_sequence_G4,
                                        function(x){getAPvector_G4(x,peak_width=2000,scoreThr=10)}))
```

# Load loops

```
getGR = function(chr,start,end,offset){
  tp = GRanges(seqnames = Rle(chr),
                  ranges = IRanges(start-offset,
                                   end = end+offset),
                  strand = Rle(rep("*", length(start))),
                  loop = seq(1, length(start)) ) 
  seqlevelsStyle(tp)='ucsc' 
  return(tp)}

genome = BSgenome.Mmusculus.UCSC.mm10
si = seqinfo(genome)

ga = binAnno( si, as.list(paste0('chr', c(1:19,'X','Y'))), 10000)
ga$binid = unlist(lapply( split(ga,ga$chr)[paste0('chr', c(1:19,'X','Y'))], function(x){seq(1,nrow(x))}) )

gagr = GRanges(seqnames = Rle(ga$chr),
                 ranges = IRanges(as.numeric(ga$start),
                                  end = as.numeric(ga$end),
                                  names = seq(1, nrow(ga))),
                 strand = Rle(rep("*", nrow(ga))),
               binid = ga$binid)

ga5 = binAnno( si, as.list(paste0('chr', c(1:19,'X','Y'))), 5000)
ga5$binid = unlist(lapply( split(ga5,ga5$chr)[paste0('chr', c(1:19,'X','Y'))], function(x){seq(1,nrow(x))}) )
gagr5 = GRanges(seqnames = Rle(ga5$chr),
                 ranges = IRanges(as.numeric(ga5$start),
                                  end = as.numeric(ga5$end),
                                  names = seq(1, nrow(ga5))),
                 strand = Rle(rep("*", nrow(ga5))),
               binid = ga5$binid,
               seqlengths = seqlengths(BSgenome.Mmusculus.UCSC.mm10))
```

```
## Warning in valid.GenomicRanges.seqinfo(x, suggest.trim = TRUE): GRanges object contains 21 out-of-bound ranges located on sequences
##   chr1, chr2, chr3, chr4, chr5, chr6, chr7, chr8, chr9, chr10, chr11,
##   chr12, chr13, chr14, chr15, chr16, chr17, chr18, chr19, chrX, and chrY.
##   Note that ranges located on a sequence whose length is unknown (NA) or
##   on a circular sequence are not considered out-of-bound (use
##   seqlengths() and isCircular() to get the lengths and circularity flags
##   of the underlying sequences). You can use trim() to trim these ranges.
##   See ?`trim,GenomicRanges-method` for more information.
```

```
gagr5 = trim(gagr5)
names(gagr5) = paste(chrom(gagr5),names(gagr5),sep="_")

loops_a1_a3 = read.delim( paste0(hic_directory,"A1_A3_merged_loops.bedpe"),
                       as.is=TRUE )
loops_a1_a3 = loops_a1_a3[-1,]
loops_a1_a3 = do.call("rbind",lapply(as.list(paste0("chr",c(1:19,"X","Y"))),function(chr){
  tp = loops_a1_a3[loops_a1_a3$X.chr1==chr,]
  tp = tp[order(tp$x1,decreasing=FALSE),]
  return(tp)
}))

rownames(loops_a1_a3) = paste0( loops_a1_a3$X.chr1,":",loops_a1_a3$x1,"-",loops_a1_a3$y2 )

loops_A1_A3_left = GRanges( seqnames = Rle(loops_a1_a3$X.chr1),
                         ranges = IRanges(start=loops_a1_a3$centroid1,
                                             end=loops_a1_a3$centroid1),
                         names=rownames(loops_a1_a3) )

loops_A1_A3_right = GRanges( seqnames = Rle(loops_a1_a3$chr2),
                          ranges = IRanges(start=loops_a1_a3$centroid2,
                                             end=loops_a1_a3$centroid2),
                          names=rownames(loops_a1_a3) )
loops_a1_a3$left_binID = gagr$binid[ subjectHits(findOverlaps(loops_A1_A3_left,gagr))]
loops_a1_a3$right_binID = gagr$binid[ subjectHits(findOverlaps(loops_A1_A3_right,gagr))]

loops_a1_a3$left_binID_random = gagr$binid[ subjectHits(findOverlaps(loops_A1_A3_left,gagr))]+8 #+8
loops_a1_a3$right_binID_random = gagr$binid[ subjectHits(findOverlaps(loops_A1_A3_right,gagr))]+8 #-8
loops_a1_a3$size = loops_a1_a3$y2-loops_a1_a3$x2

loops_a1_a3$left_CTCF = overlapsAny( GenomicRanges::resize( loops_A1_A3_left, 20000,fix="center"), ctcf_wt_NS_peaks[which(ctcf_wt_NS_peaks$motif_strand=="+")])
loops_a1_a3$right_CTCF = overlapsAny( GenomicRanges::resize( loops_A1_A3_right, 20000,fix="center"), ctcf_wt_NS_peaks[which(ctcf_wt_NS_peaks$motif_strand=="-")])

## ------
loops_to_consider = which( loops_a1_a3$size>200000 &  rowSums(loops_a1_a3[,c("left_CTCF","right_CTCF")])==2 )
anchors_left_big = GenomicRanges::resize(loops_A1_A3_left,fix="center",width=20000)
anchors_right_big = GenomicRanges::resize(loops_A1_A3_right,fix="center",width=20000)

random_loops = loops_a1_a3
random_loops$left_binID = random_loops$left_binID_random
random_loops$right_binID = random_loops$right_binID_random
random_loops$size = 10000 * (random_loops$right_binID-random_loops$left_binID)
```

# CTCF and G4 at loop anchors

```
ctcf_wt_NS_peaks_at_loop_anchors = unique(queryHits(findOverlaps(ctcf_wt_NS_peaks,c(GenomicRanges::resize( loops_A1_A3_left, 20000,fix="center"),GenomicRanges::resize( loops_A1_A3_right, 20000,fix="center")))))
ctcf_wt_NS_peaks_not_at_loop_anchors = (1:length(ctcf_wt_NS_peaks))[-unique(queryHits(findOverlaps(ctcf_wt_NS_peaks,c(GenomicRanges::resize( loops_A1_A3_left, 20000,fix="center"),GenomicRanges::resize( loops_A1_A3_right, 20000,fix="center")))))]

ctcf_wt_NS_peaks_correct_ori_left = ctcf_wt_NS_peaks[ which(ctcf_wt_NS_peaks$motif_strand=="+")]
ctcf_wt_NS_peaks_correct_ori_left = ctcf_wt_NS_peaks_correct_ori_left[unique(queryHits(findOverlaps(ctcf_wt_NS_peaks_correct_ori_left,GenomicRanges::resize( loops_A1_A3_left, 10000,fix="center"))))]

ctcf_wt_NS_peaks_correct_ori_right = ctcf_wt_NS_peaks[ which(ctcf_wt_NS_peaks$motif_strand=="-")]
ctcf_wt_NS_peaks_correct_ori_right = ctcf_wt_NS_peaks_correct_ori_right[unique(queryHits(findOverlaps(ctcf_wt_NS_peaks_correct_ori_right,GenomicRanges::resize( loops_A1_A3_right, 10000,fix="center"))))]
```

```
par(mfrow=c(1,1),mar=c(5,5,5,3))
plot( x=seq(-1000,1000,length.out=200),
      y=colMeans(a3_1_ctcf_rpgc_ctcf_AP[which(ctcf_wt_NS_peaks$name %in% c(ctcf_wt_NS_peaks_correct_ori_left$name,ctcf_wt_NS_peaks_correct_ori_right$name)),]),
      ty="l", col="green4", ylim=c(0,60), 
      ylab="RPGC", xlab="Distance from CTCF peak summit", main="",lwd=2)
lines(x=seq(-1000,1000,length.out=200),
      y=colMeans(a3_1_ctcf_rpgc_ctcf_AP[ctcf_wt_NS_peaks_not_at_loop_anchors,]),
      lwd=2,
      col="brown" )
axis(1,lwd=2)
axis(2,lwd=2)
box(col="black",lwd=2)
```

```
df = data.frame( location=seq(-1000,1000,length.out=200),
                 loop=colMeans(a3_1_ctcf_rpgc_ctcf_AP[which(ctcf_wt_NS_peaks$name %in% c(ctcf_wt_NS_peaks_correct_ori_left$name,ctcf_wt_NS_peaks_correct_ori_right$name)),]),
                 not_loop =colMeans(a3_1_ctcf_rpgc_ctcf_AP[ctcf_wt_NS_peaks_not_at_loop_anchors,]) )
                 
write.table(df,file=paste0(source_data_directory,"AP_CTCF_loop_no_loop.txt"),row.names = FALSE, sep="\t",quote=FALSE)
```

```
library(forecast)
```

```
## Warning: pakiet 'forecast' został zbudowany w wersji R 4.1.2
```

```
## Registered S3 method overwritten by 'quantmod':
##   method            from
##   as.zoo.data.frame zoo
```

```
## 
## Dołączanie pakietu: 'forecast'
```

```
## Następujący obiekt został zakryty z 'package:ggpubr':
## 
##     gghistogram
```

```
load(paste0(objects_directory,"all_CTCF_sequence_G4.RData"))

getAPvector_G4_Pos = function(G5res,peak_width, scoreThr){
    # G5res=all_CTCF_sequence_G4[[4]];peak_width=1000;scoreThr=20
    tpgr = IRanges(seq(1,peak_width,by=20),seq(20,peak_width,by=20))
    theser = G5res@ranges[which(G5res@elementMetadata$score>scoreThr & G5res@elementMetadata$strand=="+")]
    if(length(theser)>0){res = countOverlaps(tpgr,theser)}
    if(length(theser)==0){res =  rep(0, length(tpgr))}
    return(res) }
getAPvector_G4_Neg = function(G5res,peak_width, scoreThr){
    # G5res=all_CTCF_sequence_G4[[4]];peak_width=1000;scoreThr=20
    tpgr = IRanges(seq(1,peak_width,by=20),seq(20,peak_width,by=20))
    theser = G5res@ranges[which(G5res@elementMetadata$score>scoreThr & G5res@elementMetadata$strand=="-")]
    if(length(theser)>0){res = countOverlaps(tpgr,theser)}
    if(length(theser)==0){res =  rep(0, length(tpgr))}
    return(res) }

G4_all_CTCF_AP_Pos = do.call("rbind",lapply(all_CTCF_sequence_G4,
                                        function(x){getAPvector_G4_Pos(x,peak_width=2000,scoreThr=20)}))

G4_all_CTCF_AP_Neg = do.call("rbind",lapply(all_CTCF_sequence_G4,
                                        function(x){getAPvector_G4_Neg(x,peak_width=2000,scoreThr=20)}))

sma1=TTR::SMA((colMeans(G4_all_CTCF_AP_Pos[which(ctcf_wt_NS_peaks$name %in% c(ctcf_wt_NS_peaks_correct_ori_left$name)),]>0)+rev(colMeans(G4_all_CTCF_AP_Neg[which(ctcf_wt_NS_peaks$name %in% c(ctcf_wt_NS_peaks_correct_ori_right$name)),]>0)))/2,n=3)
sma2=TTR::SMA((colMeans(G4_all_CTCF_AP_Neg[which(ctcf_wt_NS_peaks$name %in% c(ctcf_wt_NS_peaks_correct_ori_left$name)),]>0)+rev(colMeans(G4_all_CTCF_AP_Pos[which(ctcf_wt_NS_peaks$name %in% c(ctcf_wt_NS_peaks_correct_ori_right$name)),]>0)))/2,n=3)


par(mfrow=c(1,1),mar=c(5,4,4,1),pty="m")
plot(x=seq(-1000,1000,
           length.out=length(sma1)),
     sma1, 
     ty="l",lwd=2,
     ylab="presence of G4q",
     xlab="Distance from CTCF summit (bp)", col="black",
     ylim=c(0.05,0.1))
lines( x=seq(-1000,1000,length.out=length(sma1)),
       sma2, col="orange2",lwd=2 )
box(col="black",lwd=2)
axis(1,lwd=2)
axis(2,lwd=2)
abline(v=0,lwd=1.5,col="gray")
abline(h=mean(c(sma1[1:50],sma2[1:50]),na.rm=TRUE),
       lty=2,lwd=0.75,col="gray")
abline(h=mean(c(sma1[51:100],sma2[51:100]),na.rm=TRUE),
       lty=2,lwd=0.75,col="gray")
```

```
df = data.frame( location=seq(-1000,1000,
                              length.out=length(sma1)),
                 pos=sma1,
                 neg=sma2)
                 
write.table(df,file=paste0(source_data_directory,"G4dist.txt"),row.names = FALSE, sep="\t",quote=FALSE)
```

# Ddx5 and CTCF at loop anchors

```
m = matrix(c(sum(gained_all %in% ctcf_wt_NS_peaks_not_at_loop_anchors ),
             sum(gained_all %in% ctcf_wt_NS_peaks_at_loop_anchors ),
             sum(lost_all %in% ctcf_wt_NS_peaks_not_at_loop_anchors ),
             sum(lost_all %in% ctcf_wt_NS_peaks_at_loop_anchors ) ),2,2,byrow = T)
barplot(m, beside=TRUE, col= c("orange3","purple4"),
        lwd=2,ylim=c(0,200), names=c("Other","Anchor") )
```

```
fisher.test(m)
```

```
## 
##  Fisher's Exact Test for Count Data
## 
## data:  m
## p-value = 6.906e-14
## alternative hypothesis: true odds ratio is not equal to 1
## 95 percent confidence interval:
##  3.484954 9.770175
## sample estimates:
## odds ratio 
##   5.774276
```

# Deep coverage data preparations

```
load( paste0( hic_directory,"wt_A3_full.RData" ) )
load(paste0( hic_directory,"A1_10kb_hic.RData" ) ) # NT1
load(paste0( hic_directory,"cb1_lib28_10kb.RData" ) ) # cb1_lib28_10kb
load(paste0( hic_directory,"ce10_10kb.RData" ) ) # ce10_10kb
```

# Distance decline plot

```
getDD = function( ipf_chr_object, binSize, distance2consider ){
  # tp = NT1$chr1$balanced;binSize=10000;distance2consider=10000000
  tp = as.data.frame(summary(ipf_chr_object))
  tp$distance = binSize*(abs(tp$j-tp$i))
  tp = tp[ tp$distance<distance2consider,]
  distances = data.frame( seq(0,distance2consider,by=binSize) )
  colnames(distances) = "distance"
  tps = split(tp,tp$distance)
  res = lapply(tps, function(x){ median(x$x) })
  res = do.call("rbind",res)
  distances$x = NA
  distances$x[match(as.numeric(rownames(res)), distances$distance)] = res[,1]
  return(distances) }

wt1_dd = lapply( NT1, function(x){
  ipf_chr_object=x$balanced
  return( getDD(ipf_chr_object,10000,50000000))})
wt3_dd = lapply( wt_A3, function(x){
  ipf_chr_object=x$balanced
  return( getDD(ipf_chr_object,10000,50000000))})
cb1_dd = lapply( cb1_lib28_10kb, function(x){
  ipf_chr_object=x$balanced
  return( getDD(ipf_chr_object,10000,50000000))})
ce10_dd = lapply( ce10_10kb, function(x){
  ipf_chr_object=x$balanced
  return( getDD(ipf_chr_object,10000,50000000))})
```

We can apply mean

```
chroms=c(1,2,3,4,5,6,7,8,9,10,11,12,13,15,16,17,18,19)

wt1_DD = do.call("cbind",lapply( wt1_dd[chroms],function(x){x$x}))
wt3_DD = do.call("cbind",lapply( wt3_dd[chroms],function(x){x$x}))
cb1_DD = do.call("cbind",lapply( cb1_dd[chroms],function(x){x$x}))
ce10_DD = do.call("cbind",lapply( ce10_dd[chroms],function(x){x$x}))

par(mfrow=c(1,1),mar=c(5,5,5,2))
plot( x=log10( wt1_dd[[1]]$distance ),
      y=log10(rowMeans(cbind(wt1_DD,wt3_DD))), 
      ty="l", col="blue3", main="",
      xlab = expression(Log[10] ~ (Distance (bp))),
      ylab =  expression(Log[10] ~ (Median (HiC))),
      ylim=c(-4,-1.5), lwd=2, cex.lab=1.5, cex.axis=1.5 )
lines( x=log10( cb1_dd[[1]]$distance ),
       y=log10(rowMeans(cbind(cb1_DD,ce10_DD))),col="steelblue3",lwd=2)
axis(1,lwd=2,cex.axis=1.5)
axis(2,lwd=2,cex.axis=1.5)
box(col="black",lwd=2)
```

```
df = data.frame( distance=log10( wt1_dd[[1]]$distance ),
                 wt=log10(rowMeans(cbind(wt1_DD,wt3_DD))),
                 ddx5_KO=log10(rowMeans(cbind(cb1_DD,ce10_DD))))
                 
write.table(df,file=paste0(source_data_directory,"DD_wt_mut.txt"),row.names = FALSE, sep="\t",quote=FALSE)
```

# Loop analysis

```
## [1] "chr1"
## [1] "chr2"
## [1] "chr3"
## [1] "chr4"
## [1] "chr5"
## [1] "chr6"
## [1] "chr7"
## [1] "chr8"
## [1] "chr9"
## [1] "chr10"
## [1] "chr11"
## [1] "chr12"
## [1] "chr13"
## [1] "chr14"
## [1] "chr15"
## [1] "chr16"
## [1] "chr17"
## [1] "chr18"
## [1] "chr19"
```

```
## [1] "chr1"
## [1] "chr2"
## [1] "chr3"
## [1] "chr4"
## [1] "chr5"
## [1] "chr6"
## [1] "chr7"
## [1] "chr8"
## [1] "chr9"
## [1] "chr10"
## [1] "chr11"
## [1] "chr12"
## [1] "chr13"
## [1] "chr14"
## [1] "chr15"
## [1] "chr16"
## [1] "chr17"
## [1] "chr18"
## [1] "chr19"
```

```
## [1] "chr1"
## [1] "chr2"
## [1] "chr3"
## [1] "chr4"
## [1] "chr5"
## [1] "chr6"
## [1] "chr7"
## [1] "chr8"
## [1] "chr9"
## [1] "chr10"
## [1] "chr11"
## [1] "chr12"
## [1] "chr13"
## [1] "chr14"
## [1] "chr15"
## [1] "chr16"
## [1] "chr17"
## [1] "chr18"
## [1] "chr19"
```

```
## [1] "chr1"
## [1] "chr2"
## [1] "chr3"
## [1] "chr4"
## [1] "chr5"
## [1] "chr6"
## [1] "chr7"
## [1] "chr8"
## [1] "chr9"
## [1] "chr10"
## [1] "chr11"
## [1] "chr12"
## [1] "chr13"
## [1] "chr14"
## [1] "chr15"
## [1] "chr16"
## [1] "chr17"
## [1] "chr18"
## [1] "chr19"
```

```
i=9;j=13

A1_loop_IPF_signal = unlist(lapply(APA_A1_IPF_sig,function(x){sum(x[i:j,i:j],
                                                                  na.rm = TRUE)}))
A3_loop_IPF_signal = unlist(lapply(APA_A3_IPF_sig,function(x){sum(x[i:j,i:j],
                                                                  na.rm = TRUE)}))

CB1_loop_IPF_signal = unlist(lapply(APA_CB1_IPF_sig,function(x){sum(x[i:j,i:j],
                                                                  na.rm = TRUE)}))
CE10_loop_IPF_signal = unlist(lapply(APA_CE10_IPF_sig,function(x){sum(x[i:j,i:j],
                                                                  na.rm = TRUE)}))


ipf = data.frame(a1=A1_loop_IPF_signal,
                 a3=A3_loop_IPF_signal,
                 cb1 = CB1_loop_IPF_signal,
                 ce10 = CE10_loop_IPF_signal)
```

# Loop signal

Prepare IPF, loop signal and LFM signal.

```
loop_signal = data.frame( a1 = getLoopSignal(APA_A1_IPF_sig,2),
                          a3 = getLoopSignal(APA_A3_IPF_sig,2),
                          cb1 = getLoopSignal(APA_CB1_IPF_sig,2),
                          ce10 = getLoopSignal(APA_CE10_IPF_sig,2))
real_loops = rownames( loop_signal[ rowMeans(loop_signal)>1,] )
real_loops = real_loops[real_loops %in% rownames(loops_a1_a3[rowSums(loops_a1_a3[,c("left_CTCF","right_CTCF")])==2 ,])]

wt_ls = rowMeans( loop_signal[rownames(loop_signal) %in% real_loops,c("a1","a3")])
mt_ls = rowMeans( loop_signal[rownames(loop_signal) %in% real_loops,c("cb1","ce10")])

ko_ls_minus_wt_ls = mt_ls-wt_ls
save( loop_signal, real_loops, wt_ls, mt_ls, ko_ls_minus_wt_ls,
      file=paste0(objects_directory,"loop_singal_Ddx5ko_Wt.RData"))
```

# Lost and gained loops in the wt and Ddx5-/- cells

Differential loops - both the IPF signal and loop signal gained in all the possible combinations.

```
load( paste0(objects_directory,"loop_singal_Ddx5ko_Wt.RData") )
lost_loops = ipf[ipf$a1>ipf$cb1 & ipf$a1>ipf$ce10 & ipf$a3>ipf$cb1 & ipf$a3>ipf$ce10 & loop_signal$a1>loop_signal$cb1 & loop_signal$a1>loop_signal$ce10 & loop_signal$a3>loop_signal$cb1 & loop_signal$a3>loop_signal$ce10,]
gained_loops = ipf[ipf$cb1>ipf$a1 & ipf$cb1>ipf$a3 & ipf$ce10>ipf$a1 & ipf$cb1>ipf$a3 & loop_signal$a1<loop_signal$cb1 & loop_signal$a1<loop_signal$ce10 & loop_signal$a3<loop_signal$cb1 & loop_signal$a3<loop_signal$ce10,]

dim(lost_loops)
```

```
## [1] 1381    4
```

```
dim(gained_loops)
```

```
## [1] 73  4
```

```
par(mfrow=c(1,1),mar=c(5,5,4,1),pty="m")
barplot( c(nrow(lost_loops),nrow(gained_loops)),
         col = c("gray40","green3"), ylim=c(0,1500),
         names=c("lost","gained"),main="Loop change upon Ddx5 loss")
axis(2,lwd=2)
```

```
loop4analysis = rownames(lost_loops)
loop4analysis = loop4analysis[loop4analysis %in% rownames(loops_a1_a3[loops_a1_a3$size>100000,])]
length(loop4analysis)
```

```
## [1] 1173
```

```
A1_loops_A1_sig_AP = Reduce("+",APA_A1_IPF_sig[names(APA_A1_IPF_sig) %in% loop4analysis])/length(loop4analysis) 
A1_loops_A3_sig_AP = Reduce("+",APA_A3_IPF_sig[names(APA_A3_IPF_sig) %in% loop4analysis])/length(loop4analysis) 

A1_loops_CB1_sig_AP = Reduce("+",APA_CB1_IPF_sig[names(APA_CB1_IPF_sig) %in% loop4analysis])/length(loop4analysis) 
A1_loops_CE10_sig_AP = Reduce("+",APA_CE10_IPF_sig[names(APA_CE10_IPF_sig) %in% loop4analysis])/length(loop4analysis) 

loops_wt_sig_AP = ( A1_loops_A1_sig_AP + A1_loops_A3_sig_AP )/2
loops_Ddx5_sig_AP = ( A1_loops_CB1_sig_AP + A1_loops_CE10_sig_AP )/2

loops_wt_sig_AP = t(matrix(apply(loops_wt_sig_AP,2,rev),21,21))
loops_Ddx5_sig_AP = t(matrix(apply(loops_Ddx5_sig_AP,2,rev),21,21))

sum( loops_wt_sig_AP[10:12,10:12] ) / sum( loops_wt_sig_AP[16:18,4:6] )
```

```
## [1] 3.055245
```

```
sum( loops_Ddx5_sig_AP[10:12,10:12] ) / sum( loops_Ddx5_sig_AP[16:18,4:6] )
```

```
## [1] 2.534127
```

```
loops_wt_sig_AP = matrix( cut(as.numeric(loops_wt_sig_AP),
                              c(seq(0.00005,0.0075,length.out=255),Inf),labels = FALSE), 21,21 )
loops_Ddx5_sig_AP = matrix( cut(as.numeric(loops_Ddx5_sig_AP),
                              c(seq(0.00005,0.0075,length.out=255),Inf),labels = FALSE), 21,21 )

loops_wt_sig_AP[1,1]=256
loops_wt_sig_AP[21,21]=1

loops_Ddx5_sig_AP[1,1]=256
loops_Ddx5_sig_AP[21,21]=1

par(mfrow=c(1,2), pty='s',mar=c(2,2,2,2) )
image( loops_wt_sig_AP, 
       col=colorRampPalette(c("black","white","orange","red"))(256),
       axes=FALSE, main="Wild type")
box(col="black")
image( loops_Ddx5_sig_AP, 
       col=colorRampPalette(c("black","white","orange","red"))(256),
       axes=FALSE, main="Ddx5-/-")
box(col="black")
```

# IPF loop signal in the intervals of genomic distances

```
ran_A1_IPF = GetCentroidSignal( random_loops, 
                            NT1, 10,
                            paste0("chr",c(1:19) ), 
                            "balanced" )
ran_A1_IPF_sig = ProcessLoops( ran_A1_IPF )


ran_A3_IPF = GetCentroidSignal( random_loops, 
                            wt_A3, 10,
                            paste0("chr",c(1:19) ), 
                            "balanced" )
ran_A3_IPF_sig = ProcessLoops( ran_A3_IPF )


ran_CB1_IPF = GetCentroidSignal( random_loops, 
                                 cb1_lib28_10kb, 10,
                            paste0("chr",c(1:19) ), 
                            "balanced" )
ran_CB1_IPF_sig = ProcessLoops( ran_CB1_IPF )


ran_CE10_IPF = GetCentroidSignal( random_loops, 
                                 ce10_10kb, 10,
                            paste0("chr",c(1:19) ), 
                            "balanced" )
ran_CE10_IPF_sig = ProcessLoops( ran_CE10_IPF )

i=9;j=13
ran_A1_loop_IPF_signal = unlist(lapply(ran_A1_IPF_sig,function(x){sum(x[i:j,i:j],
                                                                  na.rm = TRUE)}))
ran_A3_loop_IPF_signal = unlist(lapply(ran_A3_IPF_sig,function(x){sum(x[i:j,i:j],
                                                                  na.rm = TRUE)}))

ran_CB1_loop_IPF_signal = unlist(lapply(ran_CB1_IPF_sig,function(x){sum(x[i:j,i:j],
                                                                  na.rm = TRUE)}))
ran_CE10_loop_IPF_signal = unlist(lapply(ran_CE10_IPF_sig,function(x){sum(x[i:j,i:j],
                                                                  na.rm = TRUE)}))


ran = data.frame(a1=ran_A1_loop_IPF_signal,
                 a3=ran_A3_loop_IPF_signal,
                 cb1 = ran_CB1_loop_IPF_signal,
                 ce10 = ran_CE10_loop_IPF_signal)
rand_signal = data.frame( a1 = getLoopSignal(ran_A3_IPF_sig,2),
                          a3 = getLoopSignal(ran_A3_IPF_sig,2),
                          cb1 = getLoopSignal(ran_CB1_IPF_sig,2),
                          ce10 = getLoopSignal(ran_CE10_IPF_sig,2))

save( ran, rand_signal, ran_A1_loop_IPF_signal, ran_A3_loop_IPF_signal, ran_CB1_loop_IPF_signal, 
      ran_CE10_loop_IPF_signal,
      ran_A3_IPF_sig, ran_A3_IPF_sig, ran_CB1_IPF_sig, 
      ran_CE10_IPF_sig,rand_signal,
      file=paste0(data_directory,"loops_signals_all.RData"))
```

Big list

```
load(paste0(data_directory,"loops_signals_all.RData"))
loops_a1_a3$dist_bin = cut(loops_a1_a3$size,c(0,100000,
                                              200000,400000,
                                              800000, 1600000,
                                              100000000),labels=FALSE)
loops_categories = split(rownames(loops_a1_a3),loops_a1_a3$dist_bin)

random_loops$dist_bin = cut(random_loops$size,c(0,100000,200000,
                                                400000,800000,
                                                1600000,
                                                100000000),labels=FALSE)
loops_categories_ran = split(rownames(random_loops),loops_a1_a3$dist_bin)
```

Merge them into one thing

```
id = which( rownames(loop_signal) %in% real_loops )
ratio_ipf_mut_wt = rowMeans(loop_signal[id,c("cb1","ce10")])-rowMeans(loop_signal[id,c("a1","a3")])

ratio_ipf_mut_wt_ran = rowMeans(rand_signal[,c("cb1","ce10")])-rowMeans(rand_signal[,c("a1","a3")])

resl = vector("list",2*length(loops_categories))
resl[c(1,3,5,7,9,11)] = lapply( loops_categories_ran, function(ll){ratio_ipf_mut_wt_ran[names(ratio_ipf_mut_wt_ran) %in% ll]})
resl[c(2,4,6,8,10,12)] = lapply( loops_categories, function(ll){ratio_ipf_mut_wt[names(ratio_ipf_mut_wt) %in% ll]})
resl = lapply(resl,function(x){tp=x[!is.na(x)];tp=tp[is.finite(tp)];return(tp)})


par(mfrow=c(1,1),mar=c(4,4,4,1))
boxplot(resl,outline=FALSE, notch=TRUE,
        ylim=c(-1,1), col="white", border=c("gray","purple4"),
        cex.lab=1,cex.axis=1,
        ylab=expression(Log[2] ~ (Ddx5/WT)),lwd=2)
axis(1,lwd=2,at=1:12,cex.axis=1)
axis(2,lwd=2,cex.axis=1)
box(col="black",lwd=2)
abline(h=0,lwd=2,col="red4")
```

```
t.test(resl[[1]],resl[[2]])
```

```
## 
##  Welch Two Sample t-test
## 
## data:  resl[[1]] and resl[[2]]
## t = -0.80122, df = 40.539, p-value = 0.4277
## alternative hypothesis: true difference in means is not equal to 0
## 95 percent confidence interval:
##  -0.17251789  0.07453693
## sample estimates:
##    mean of x    mean of y 
## -0.053574988 -0.004584509
```

```
t.test(resl[[3]],resl[[4]])
```

```
## 
##  Welch Two Sample t-test
## 
## data:  resl[[3]] and resl[[4]]
## t = -2.8019, df = 38.71, p-value = 0.007892
## alternative hypothesis: true difference in means is not equal to 0
## 95 percent confidence interval:
##  -0.3476802 -0.0561136
## sample estimates:
##   mean of x   mean of y 
## -0.09321102  0.10868587
```

```
t.test(resl[[5]],resl[[6]])
```

```
## 
##  Welch Two Sample t-test
## 
## data:  resl[[5]] and resl[[6]]
## t = 7.2078, df = 1474.6, p-value = 9.048e-13
## alternative hypothesis: true difference in means is not equal to 0
## 95 percent confidence interval:
##  0.05395082 0.09429570
## sample estimates:
##   mean of x   mean of y 
## -0.01644028 -0.09056354
```

```
t.test(resl[[7]],resl[[8]])
```

```
## 
##  Welch Two Sample t-test
## 
## data:  resl[[7]] and resl[[8]]
## t = 12.325, df = 2688.2, p-value < 2.2e-16
## alternative hypothesis: true difference in means is not equal to 0
## 95 percent confidence interval:
##  0.09545635 0.13157593
## sample estimates:
##    mean of x    mean of y 
##  0.004569777 -0.108946363
```

```
t.test(resl[[9]],resl[[10]])
```

```
## 
##  Welch Two Sample t-test
## 
## data:  resl[[9]] and resl[[10]]
## t = 11.114, df = 1742.6, p-value < 2.2e-16
## alternative hypothesis: true difference in means is not equal to 0
## 95 percent confidence interval:
##  0.1177987 0.1682873
## sample estimates:
##   mean of x   mean of y 
##  0.01828049 -0.12476249
```

```
t.test(resl[[11]],resl[[12]])
```

```
## 
##  Welch Two Sample t-test
## 
## data:  resl[[11]] and resl[[12]]
## t = 6.0776, df = 796.74, p-value = 1.89e-09
## alternative hypothesis: true difference in means is not equal to 0
## 95 percent confidence interval:
##  0.1108845 0.2166815
## sample estimates:
##   mean of x   mean of y 
##  0.04621846 -0.11756454
```

```
unlist(lapply(resl,length))
```

```
##  [1] 2695   40 2264   38 2742  768 2535  909 1516  565  683  198
```

```
theNtimes = max(unlist(lapply(resl,length)))
df = data.frame( gr1_matched=rep(NA,theNtimes),
                 gr1_loops=rep(NA,theNtimes),
                 gr2_matched=rep(NA,theNtimes),
                 gr2_loops=rep(NA,theNtimes),
                 gr3_matched=rep(NA,theNtimes),
                 gr3_loops=rep(NA,theNtimes),
                 gr4_matched=rep(NA,theNtimes),
                 gr4_loops=rep(NA,theNtimes),
                 gr5_matched=rep(NA,theNtimes),
                 gr5_loops=rep(NA,theNtimes),
                 gr6_matched=rep(NA,theNtimes),
                 gr6_loops=rep(NA,theNtimes))
df$gr1_matched[1:length(resl[[1]])] = resl[[1]]
df$gr1_loops[1:length(resl[[2]])] = resl[[2]]
df$gr2_matched[1:length(resl[[3]])] = resl[[3]]
df$gr2_loops[1:length(resl[[4]])] = resl[[4]]
df$gr3_matched[1:length(resl[[5]])] = resl[[5]]
df$gr3_loops[1:length(resl[[6]])] = resl[[6]]
df$gr4_matched[1:length(resl[[7]])] = resl[[7]]
df$gr4_loops[1:length(resl[[8]])] = resl[[8]]
df$gr5_matched[1:length(resl[[9]])] = resl[[9]]
df$gr5_loops[1:length(resl[[10]])] = resl[[10]]
df$gr6_matched[1:length(resl[[11]])] = resl[[11]]
df$gr6_loops[1:length(resl[[12]])] = resl[[12]]

write.table(df,file=paste0(source_data_directory,"boxplots_loop_change_wt_Ddx5_distance.txt"),row.names = FALSE, sep="\t",quote=FALSE)
```

# Examples of loops lost in the NS cells

```
rm(list=c("wt_A3","NT1","cb1_lib28_10kb","ce10_10kb"))
gc()
```

```
##              used   (Mb) gc trigger    (Mb) limit (Mb)   max used    (Mb)
## Ncells   26686194 1425.2   46187740  2466.7         NA   57734675  3083.4
## Vcells 1296007590 9887.8 4377481799 33397.6     131072 5471852248 41747.0
```

```
load(paste0( hic_directory,"ko_10kb.RData"))
load(paste0( hic_directory,"wt_A1_A3_10kb.RData"))
names(wt_10kb) = paste0("chr",c(1:19,"X"))
names(ko_10kb) = paste0("chr",c(1:19,"X"))
```

```
par(mfrow=c(1,1),mar=c(3,3,3,3), pty="s")
w=0;chr="chr10";s=102200000;en=103200000
TOP = getMatrixForARegion( lowertri=wt_10kb,
                             uppertri=ko_10kb,
                             CHROM=chr,
                             START=s-w,
                             END=en+w,
                             ga=ga,
                             upperLimit=0.012 )
```

```
## <sparse>[ <logic> ]: .M.sub.i.logical() maybe inefficient
```

```
image( TOP,axes=FALSE, col=colorRampPalette(c("white","red"))(256))
box(col="black",lwd=3)
```

```
par(mfrow=c(1,1),mar=c(3,3,3,3), pty="s")
w=0;chr="chr12";s=15600000;en=16800000
TOP = getMatrixForARegion( lowertri=wt_10kb,
                             uppertri=ko_10kb,
                             CHROM=chr,
                             START=s-w,
                             END=en+w,
                             ga=ga,
                             upperLimit=0.012 )
```

```
## <sparse>[ <logic> ]: .M.sub.i.logical() maybe inefficient
```

```
image( TOP,axes=FALSE, col=colorRampPalette(c("white","red"))(256))
box(col="black",lwd=3)
```

```
par(mfrow=c(1,1),mar=c(3,3,3,3), pty="s")
w=0;chr="chr2";s=37600000;en=38600000


TOP = getMatrixForARegion( lowertri=wt_10kb,
                             uppertri=ko_10kb,
                             CHROM=chr,
                             START=s-w,
                             END=en+w,
                             ga=ga,
                             upperLimit=0.012 )
```

```
## <sparse>[ <logic> ]: .M.sub.i.logical() maybe inefficient
```

```
image( TOP,axes=FALSE, col=colorRampPalette(c("white","red"))(256))
box(col="black",lwd=3)
```

```
par(mfrow=c(1,1),mar=c(3,3,3,3), pty="s",bty="O")
w=0;chr="chr7";s=142200000;en=142800000


TOP = getMatrixForARegion( lowertri=wt_10kb,
                             uppertri=ko_10kb,
                             CHROM=chr,
                             START=s-w,
                             END=en+w,
                             ga=ga,
                             upperLimit=0.012 )
```

```
## <sparse>[ <logic> ]: .M.sub.i.logical() maybe inefficient
```

```
image( TOP,axes=FALSE, col=colorRampPalette(c("white","red"))(256))
box(col="black",lwd=2)
```

## APA - general

```
wt_IPF = GetCentroidSignal( loops_a1_a3,
                            wt_10kb, 11,
                            paste0("chr",c(1:19) ), 
                            "balanced" )
```

```
## [1] "chr1"
## [1] "chr2"
## [1] "chr3"
## [1] "chr4"
## [1] "chr5"
## [1] "chr6"
## [1] "chr7"
## [1] "chr8"
## [1] "chr9"
## [1] "chr10"
## [1] "chr11"
## [1] "chr12"
## [1] "chr13"
## [1] "chr14"
## [1] "chr15"
## [1] "chr16"
## [1] "chr17"
## [1] "chr18"
## [1] "chr19"
```

```
wt_IPF_sig = ProcessLoops( wt_IPF )

ko_IPF = GetCentroidSignal( loops_a1_a3,
                            ko_10kb, 11,
                            paste0("chr",c(1:19) ), 
                            "balanced" )
```

```
## [1] "chr1"
## [1] "chr2"
## [1] "chr3"
## [1] "chr4"
## [1] "chr5"
## [1] "chr6"
## [1] "chr7"
## [1] "chr8"
## [1] "chr9"
## [1] "chr10"
## [1] "chr11"
## [1] "chr12"
## [1] "chr13"
## [1] "chr14"
## [1] "chr15"
## [1] "chr16"
## [1] "chr17"
## [1] "chr18"
## [1] "chr19"
```

```
ko_IPF_sig = ProcessLoops( ko_IPF )
```

```
rm(list=c("wt_10kb","ko_10kb"))
gc()
```

```
##              used    (Mb) gc trigger    (Mb) limit (Mb)   max used    (Mb)
## Ncells   27869940  1488.5   46187740  2466.7         NA   57734675  3083.4
## Vcells 1367615760 10434.1 4377481799 33397.6     131072 5471852248 41747.0
```

# 4F11 - global analysis of architectural loops

```
load(paste0( hic_directory,"4F11_DMSO_10kb_hic.RData" ) ) # DMSO_10kb
load(paste0( hic_directory,"4F11_dTAG_10kb_hic.RData" ) ) # dTAG_10kb
```

## Loops

```
DMSO_4F11_loops_IPF = GetCentroidSignal( loops_a1_a3, 
                                  DMSO_10kb, 10, 
                                  paste0("chr",c(1:19) ),
                                  which_file="balanced"  )
```

```
## [1] "chr1"
## [1] "chr2"
## [1] "chr3"
## [1] "chr4"
## [1] "chr5"
## [1] "chr6"
## [1] "chr7"
## [1] "chr8"
## [1] "chr9"
## [1] "chr10"
## [1] "chr11"
## [1] "chr12"
## [1] "chr13"
## [1] "chr14"
## [1] "chr15"
## [1] "chr16"
## [1] "chr17"
## [1] "chr18"
## [1] "chr19"
```

```
DMSO_4F11_loops_IPF_sig = ProcessLoops( DMSO_4F11_loops_IPF )

dTAG_4F11_loops_IPF = GetCentroidSignal( loops_a1_a3, 
                                  dTAG_10kb, 10, 
                                  paste0("chr",c(1:19) ),
                                  which_file="balanced"  )
```

```
## [1] "chr1"
## [1] "chr2"
## [1] "chr3"
## [1] "chr4"
## [1] "chr5"
## [1] "chr6"
## [1] "chr7"
## [1] "chr8"
## [1] "chr9"
## [1] "chr10"
## [1] "chr11"
## [1] "chr12"
## [1] "chr13"
## [1] "chr14"
## [1] "chr15"
## [1] "chr16"
## [1] "chr17"
## [1] "chr18"
## [1] "chr19"
```

```
dTAG_4F11_loops_IPF_sig = ProcessLoops( dTAG_4F11_loops_IPF )


DMSO_4F11_random_IPF = GetCentroidSignal( random_loops, 
                                  DMSO_10kb, 10, 
                                  paste0("chr",c(1:19) ),
                                  which_file="balanced"  )
```

```
## [1] "chr1"
## [1] "chr2"
## [1] "chr3"
## [1] "chr4"
## [1] "chr5"
## [1] "chr6"
## [1] "chr7"
## [1] "chr8"
## [1] "chr9"
## [1] "chr10"
## [1] "chr11"
## [1] "chr12"
## [1] "chr13"
## [1] "chr14"
## [1] "chr15"
## [1] "chr16"
## [1] "chr17"
## [1] "chr18"
## [1] "chr19"
```

```
DMSO_4F11_random_IPF_sig = ProcessLoops( DMSO_4F11_random_IPF )

dTAG_4F11_random_IPF = GetCentroidSignal( random_loops, 
                                  dTAG_10kb, 10, 
                                  paste0("chr",c(1:19) ),
                                  which_file="balanced"  )
```

```
## [1] "chr1"
## [1] "chr2"
## [1] "chr3"
## [1] "chr4"
## [1] "chr5"
## [1] "chr6"
## [1] "chr7"
## [1] "chr8"
## [1] "chr9"
## [1] "chr10"
## [1] "chr11"
## [1] "chr12"
## [1] "chr13"
## [1] "chr14"
## [1] "chr15"
## [1] "chr16"
## [1] "chr17"
## [1] "chr18"
## [1] "chr19"
```

```
dTAG_4F11_random_IPF_sig = ProcessLoops( dTAG_4F11_random_IPF )
```

```
i=9;j=13

APA_4F11_DMSO_signal = unlist(lapply(DMSO_4F11_loops_IPF_sig,function(x){sum(x[i:j,i:j],
                                                                   na.rm = TRUE)}))
APA_4F11_dTAG_signal = unlist(lapply(dTAG_4F11_loops_IPF_sig,function(x){sum(x[i:j,i:j],
                                                                   na.rm = TRUE)}))

ran_4F11_DMSO_signal = unlist(lapply(DMSO_4F11_random_IPF_sig,function(x){sum(x[i:j,i:j],
                                                                   na.rm = TRUE)}))
ran_4F11_dTAG_signal = unlist(lapply(dTAG_4F11_random_IPF_sig,function(x){sum(x[i:j,i:j],
                                                                   na.rm = TRUE)}))

degron_ipf = data.frame(dmso = APA_4F11_DMSO_signal,
                        dtag = APA_4F11_dTAG_signal)
random_ipf = data.frame(dmso = ran_4F11_DMSO_signal,
                        dtag = ran_4F11_dTAG_signal)
```

This plot is nice. Here we look at loops with anchors intersecting CTCF peaks losign signal upon dTAG13 treatment.

```
loop4analysis = unique(c(anchors_left_big[unique(queryHits(findOverlaps(anchors_left_big,acute_loss_ctcf)))]$names,anchors_right_big[unique(queryHits(findOverlaps(anchors_right_big,acute_loss_ctcf)))]$names))
length(loop4analysis)
```

```
## [1] 5648
```

The fold change of loop signal in the DMSO and dTAG13 treated cells for the loops which lose CTCF

```
fc_dTAG = log2((degron_ipf$dtag)/(degron_ipf$dmso))
sig_dTAG = cut( degron_ipf$dmso,
                c(0,0.04,0.08,0.16,0.32,Inf),
                labels=FALSE )

table(sig_dTAG)
```

```
## sig_dTAG
##    1    2    3    4    5 
##  799 2222 4162 4108 1260
```

```
fcs = split(fc_dTAG,sig_dTAG)
par(pty="m")
boxplot(fcs,outline=FALSE,notch=TRUE, col="white",
        border=colorRampPalette(c("green4","blue4"))(5),lwd=2)
abline(h=0,lwd=2)
axis(1,lwd=2)
axis(2,lwd=2)
box(col="black",lwd=2)
```

```
t.test(fcs[[1]])
```

```
## 
##  One Sample t-test
## 
## data:  fcs[[1]]
## t = 1.2039, df = 798, p-value = 0.229
## alternative hypothesis: true mean is not equal to 0
## 95 percent confidence interval:
##  -0.01127725  0.04705101
## sample estimates:
##  mean of x 
## 0.01788688
```

```
t.test(fcs[[2]])
```

```
## 
##  One Sample t-test
## 
## data:  fcs[[2]]
## t = -8.9669, df = 2221, p-value < 2.2e-16
## alternative hypothesis: true mean is not equal to 0
## 95 percent confidence interval:
##  -0.03910128 -0.02506767
## sample estimates:
##   mean of x 
## -0.03208447
```

```
t.test(fcs[[3]])
```

```
## 
##  One Sample t-test
## 
## data:  fcs[[3]]
## t = -20.027, df = 4161, p-value < 2.2e-16
## alternative hypothesis: true mean is not equal to 0
## 95 percent confidence interval:
##  -0.05603898 -0.04604542
## sample estimates:
##  mean of x 
## -0.0510422
```

```
t.test(fcs[[4]])
```

```
## 
##  One Sample t-test
## 
## data:  fcs[[4]]
## t = -46.118, df = 4107, p-value < 2.2e-16
## alternative hypothesis: true mean is not equal to 0
## 95 percent confidence interval:
##  -0.07787825 -0.07152685
## sample estimates:
##   mean of x 
## -0.07470255
```

```
t.test(fcs[[5]])
```

```
## 
##  One Sample t-test
## 
## data:  fcs[[5]]
## t = -22.315, df = 1259, p-value < 2.2e-16
## alternative hypothesis: true mean is not equal to 0
## 95 percent confidence interval:
##  -0.08480700 -0.07110044
## sample estimates:
##   mean of x 
## -0.07795372
```

```
t.test(fcs[[1]],fcs[[5]])
```

```
## 
##  Welch Two Sample t-test
## 
## data:  fcs[[1]] and fcs[[5]]
## t = 6.2795, df = 886.95, p-value = 5.313e-10
## alternative hypothesis: true difference in means is not equal to 0
## 95 percent confidence interval:
##  0.06588575 0.12579544
## sample estimates:
##   mean of x   mean of y 
##  0.01788688 -0.07795372
```

```
theNtimes = max(unlist(lapply(fcs,length)))
df = data.frame( gr1=rep(NA,theNtimes),
                 gr2=rep(NA,theNtimes),
                 gr3=rep(NA,theNtimes),
                 gr4=rep(NA,theNtimes),
                 gr5=rep(NA,theNtimes))
df$gr1[1:length(fcs[[1]])] = fcs[[1]]
df$gr2[1:length(fcs[[2]])] = fcs[[2]]
df$gr3[1:length(fcs[[3]])] = fcs[[3]]
df$gr4[1:length(fcs[[4]])] = fcs[[4]]
df$gr5[1:length(fcs[[5]])] = fcs[[5]]
write.table(df,file=paste0(source_data_directory,"boxplots_Loop_signal_FC_dTAG.txt"),row.names = FALSE, sep="\t",quote=FALSE)
```

# Can we link loop loss to Ddx5 effect on CTCF?

```
lost_loop_anchors_left = anchors_left_big[which(anchors_left_big$names %in% rownames(lost_loops))]
lost_loop_anchors_right = anchors_right_big[which(anchors_right_big$names %in% rownames(lost_loops))]
all(lost_loop_anchors_left$names==lost_loop_anchors_right$names)
```

```
## [1] TRUE
```

```
l = data.frame( lost_left_lost_ctcf = countOverlaps(lost_loop_anchors_left,acute_loss_ctcf),
                lost_right_lost_ctcf = countOverlaps(lost_loop_anchors_right,acute_loss_ctcf),
                lost_left_lost_ctcf_ko = countOverlaps(lost_loop_anchors_left,ctcf_wt_NS_peaks[as.numeric(rownames(res_Ddx5_ctcf_Deseq2_RPGC[res_Ddx5_ctcf_Deseq2_RPGC$col=="#305494",]))]),
                lost_right_lost_ctcf_ko = countOverlaps(lost_loop_anchors_right,ctcf_wt_NS_peaks[as.numeric(rownames(res_Ddx5_ctcf_Deseq2_RPGC[res_Ddx5_ctcf_Deseq2_RPGC$col=="#305494",]))]),
                row.names = lost_loop_anchors_right$names )

## ko_ls_minus_wt_ls --> all the architectural loops
## rownames(l[rowSums(l[,1:2])>0,]) --> architectural loops whereby I see a loss of CTCF binding in dTAG13 treated cells
## ko_ls_minus_wt_ls[names(ko_ls_minus_wt_ls) %in% rownames(l[rowSums(l[,3:4])>0,])]
par(mfrow=c(1,1),pty="m",mar=c(5,5,5,1),bty="n")
boxplot( ko_ls_minus_wt_ls,
         ko_ls_minus_wt_ls[names(ko_ls_minus_wt_ls) %in% rownames(l[rowSums(l[,1:2])>0,])],
         ko_ls_minus_wt_ls[names(ko_ls_minus_wt_ls) %in% rownames(l[rowSums(l[,3:4])>0,])],
         names=c("all loops","dTAG","KO"),
         notch=TRUE, outline=FALSE,ylim=c(-1,1),
         col=c("white"), border=c("black","gray40","steelblue3"),lwd=2,
         ylab="Loop strength change LFC Mut vs Wt")
axis(1,lwd=2,at=c(1,2,3),c("all loops","dTAG","KO"))
axis(2,lwd=2)
abline(h=0,lwd=2,lty=2)
```

```
t.test( ko_ls_minus_wt_ls, ko_ls_minus_wt_ls[names(ko_ls_minus_wt_ls) %in% rownames(l[rowSums(l[,1:2])>0,])] )
```

```
## 
##  Welch Two Sample t-test
## 
## data:  ko_ls_minus_wt_ls and ko_ls_minus_wt_ls[names(ko_ls_minus_wt_ls) %in% rownames(l[rowSums(l[, 1:2]) > 0, ])]
## t = 20.853, df = 283.56, p-value < 2.2e-16
## alternative hypothesis: true difference in means is not equal to 0
## 95 percent confidence interval:
##  0.2039634 0.2464822
## sample estimates:
##  mean of x  mean of y 
## -0.1026239 -0.3278467
```

```
t.test( ko_ls_minus_wt_ls, ko_ls_minus_wt_ls[names(ko_ls_minus_wt_ls) %in% rownames(l[rowSums(l[,3:4])>0,])] )
```

```
## 
##  Welch Two Sample t-test
## 
## data:  ko_ls_minus_wt_ls and ko_ls_minus_wt_ls[names(ko_ls_minus_wt_ls) %in% rownames(l[rowSums(l[, 3:4]) > 0, ])]
## t = 10.936, df = 47.16, p-value = 1.579e-14
## alternative hypothesis: true difference in means is not equal to 0
## 95 percent confidence interval:
##  0.1893869 0.2747665
## sample estimates:
##  mean of x  mean of y 
## -0.1026239 -0.3347006
```

```
length(ko_ls_minus_wt_ls)
```

```
## [1] 2518
```

```
length( ko_ls_minus_wt_ls[names(ko_ls_minus_wt_ls) %in% rownames(l[rowSums(l[,1:2])>0,])])
```

```
## [1] 195
```

```
length(ko_ls_minus_wt_ls[names(ko_ls_minus_wt_ls) %in% rownames(l[rowSums(l[,3:4])>0,])])
```

```
## [1] 44
```

```
theNtimes = length(ko_ls_minus_wt_ls)
df = data.frame( gr1=rep(NA,theNtimes),
                 gr2=rep(NA,theNtimes),
                 gr3=rep(NA,theNtimes))
df$gr1[1:length(ko_ls_minus_wt_ls)] = ko_ls_minus_wt_ls
df$gr2[1:length(ko_ls_minus_wt_ls[names(ko_ls_minus_wt_ls) %in% rownames(l[rowSums(l[,1:2])>0,])])] = ko_ls_minus_wt_ls[names(ko_ls_minus_wt_ls) %in% rownames(l[rowSums(l[,1:2])>0,])]
df$gr3[1:length(ko_ls_minus_wt_ls[names(ko_ls_minus_wt_ls) %in% rownames(l[rowSums(l[,3:4])>0,])])] = ko_ls_minus_wt_ls[names(ko_ls_minus_wt_ls) %in% rownames(l[rowSums(l[,3:4])>0,])]

write.table(df,file=paste0(source_data_directory,"boxplots_Loop_signal_FC_ctcf_ko_dTAG.txt"),
            row.names = FALSE, sep="\t",quote=FALSE)
```

## dTAG13 and chromatin interactions

```
load(paste0( hic_directory,"4F11_DMSO_5kb_hic.RData" ) ) 
load(paste0( hic_directory,"4F11_dTAG_5kb_hic.RData" ) )
```

```
par(mfrow=c(1,1),mar=c(3,3,3,3), pty="s",bty="O")
chr="chr3";s=123160000;en=123355000;w=150000

TOP = getMatrixForARegion( lowertri=DMSO_5kb,
                             uppertri=dTAG_5kb,
                             CHROM=chr,
                             START=s-w,
                             END=en+w,
                             ga=ga5,
                             upperLimit=0.012 )
```

```
## <sparse>[ <logic> ]: .M.sub.i.logical() maybe inefficient
```

```
image( TOP,axes=FALSE, col=colorRampPalette(c("white","red"))(256))
box(col="black",lwd=3)
```

Loop signals for tables

```
loop_singals_Ddx5 = cbind(ipf,degron_ipf)
write.table( loop_singals_Ddx5, file=paste0(data_directory,"loop_singals_Ddx5.txt"),
             quote=FALSE, row.names=TRUE, col.names = TRUE,
             sep="\t" )
```

# Pantr1 - general Hi-C

```
rm(list=c("DMSO_5kb","dTAG_5kb"))
load(paste0( hic_directory,"A3_rep2_10kb_hic.RData" ) ) # A3_rep2
load(paste0( hic_directory,"PB6_10kb_hic.RData" ) ) # pb6
load(paste0( hic_directory,"PE3_10kb_hic.RData" ) ) # Treated
pe3=Treated
rm(list=c("Treated"))
gc()
```

```
##              used    (Mb) gc trigger    (Mb) limit (Mb)   max used    (Mb)
## Ncells   28026990  1496.9   46187740  2466.7         NA   57734675  3083.4
## Vcells 4777823025 36451.9 6317706219 48200.3     131072 5471852248 41747.0
```

Distance decline

```
getDD = function( ipf_chr_object, binSize, distance2consider ){
  # tp = NT1$chr1$balanced;binSize=10000;distance2consider=10000000
  tp = as.data.frame(summary(ipf_chr_object))
  tp$distance = binSize*(abs(tp$j-tp$i))
  tp = tp[ tp$distance<distance2consider,]
  distances = data.frame( seq(0,distance2consider,by=binSize) )
  colnames(distances) = "distance"
  tps = split(tp,tp$distance)
  res = lapply(tps, function(x){ median(x$x) })
  res = do.call("rbind",res)
  distances$x = NA
  distances$x[match(as.numeric(rownames(res)), distances$distance)] = res[,1]
  return(distances) }

a3_2_dd = lapply( A3_rep2, function(x){
  ipf_chr_object=x$balanced
  return( getDD(ipf_chr_object,10000,50000000))})
pb6_dd = lapply( pb6, function(x){
  ipf_chr_object=x$balanced
  return( getDD(ipf_chr_object,10000,50000000))})
pe3_dd = lapply( pe3, function(x){
  ipf_chr_object=x$balanced
  return( getDD(ipf_chr_object,10000,50000000))})

save( a3_2_dd, pb6_dd, pe3_dd, 
      file=paste0(objects_directory,"Pantr1_DD.RData") )
```

We can apply mean

```
chroms=c(2,3,4,5,6,7,8,9,10,11,12,13,15,16,17,18,19)
load(paste0(objects_directory,"Pantr1_DD.RData"))

wt1_DD = do.call("cbind",lapply( wt1_dd[chroms],function(x){x$x}))
wt3_DD = do.call("cbind",lapply( wt3_dd[chroms],function(x){x$x}))
a3_2_DD = do.call("cbind",lapply( a3_2_dd[chroms],function(x){x$x}))
pb6_DD = do.call("cbind",lapply( pb6_dd[chroms],function(x){x$x}))
pe3_DD = do.call("cbind",lapply( pe3_dd[chroms],function(x){x$x}))

par(mfrow=c(1,1))
plot( x=log10( a3_2_dd$chr1$distance ),
      y=log10(rowMeans(a3_2_DD)), 
      ty="l", col="blue3", main="",
      xlab = expression(Log[10] ~ (Distance (bp))),
      ylab =  expression(Log[10] ~ (Median (HiC))),
      ylim=c(-4,-1.5), lwd=2 )
lines( x=log10( a3_2_dd$chr1$distance ),
       y=log10(rowMeans(wt1_DD)),col="blue3",lwd=2)
lines( x=log10( a3_2_dd$chr1$distance ),
       y=log10(rowMeans(wt3_DD)),col="blue3",lwd=2)

lines( x=log10( a3_2_dd$chr1$distance ),
       y=log10(rowMeans(pb6_DD)),col="deeppink4",lwd=2)
lines( x=log10( a3_2_dd$chr1$distance ),
       y=log10(rowMeans(pe3_DD)),col="deeppink4",lwd=2)

axis(1,lwd=2)
axis(2,lwd=2)
box(col="black",lwd=2)
```

```
df = data.frame( distance=log10( a3_2_dd$chr1$distance ),
                 wt1=log10(rowMeans(a3_2_DD)),
                 wt2=log10(rowMeans(wt1_DD)),
                 wt3=log10(rowMeans(wt3_DD)),
                 pantr1_ko_pb6=log10(rowMeans(pb6_DD)),
                 pantr1_ko_pe3=log10(rowMeans(pe3_DD)) )
                 
write.table(df,file=paste0(source_data_directory,"DD_wt_mutPantr1.txt"),
            row.names = FALSE, sep="\t",quote=FALSE)
```

Example loci

```
chr="chr5";s=99200000;en=100050000;w=300000;ul=0.02 # in the fig.
chr="chr1";s=125775000;en=126425000;w=300000;ul=0.01

TO1 = getMatrixForARegion( lowertri=A3_rep2,
                           uppertri=A3_rep2,
                           CHROM=chr,
                           START=s-w,
                           END=en+w,
                           ga=ga,
                           upperLimit=ul )
```

```
## <sparse>[ <logic> ]: .M.sub.i.logical() maybe inefficient
```

```
TO2 = getMatrixForARegion( lowertri=pe3,
                           uppertri=pe3,
                           CHROM=chr,
                           START=s-w,
                           END=en+w,
                           ga=ga,
                           upperLimit=ul )
```

```
## <sparse>[ <logic> ]: .M.sub.i.logical() maybe inefficient
```

```
TO3 = getMatrixForARegion( lowertri=pb6,
                           uppertri=pb6,
                           CHROM=chr,
                           START=s-w,
                           END=en+w,
                           ga=ga,
                           upperLimit=ul )
```

```
## <sparse>[ <logic> ]: .M.sub.i.logical() maybe inefficient
```

```
par(mfrow=c(1,3),mar=c(1,1,2,1), pty="s",bty="O")
image( TO1,axes=FALSE, col=colorRampPalette(c("white","red"))(256),
       main="Wild type")
box(col="black",lwd=3) 
image( TO2,axes=FALSE, col=colorRampPalette(c("white","red"))(256),
       main="Pantr1 - PE3")
box(col="black",lwd=3) 
image( TO3,axes=FALSE, col=colorRampPalette(c("white","red"))(256),
       main="Pantr1 - PB6")
box(col="black",lwd=3)
```

Loop signal

```
i=9;j=13

a3_2_loops_IPF = GetCentroidSignal( loops_a1_a3, 
                                    A3_rep2, 10, 
                                    paste0("chr",c(1:19) ),
                                    which_file="balanced"  )
```

```
## [1] "chr1"
## [1] "chr2"
## [1] "chr3"
## [1] "chr4"
## [1] "chr5"
## [1] "chr6"
## [1] "chr7"
## [1] "chr8"
## [1] "chr9"
## [1] "chr10"
## [1] "chr11"
## [1] "chr12"
## [1] "chr13"
## [1] "chr14"
## [1] "chr15"
## [1] "chr16"
## [1] "chr17"
## [1] "chr18"
## [1] "chr19"
```

```
a3_2_loops_IPF_sig = ProcessLoops( a3_2_loops_IPF )

pe3_loops_IPF = GetCentroidSignal( loops_a1_a3, 
                                  pe3, 10, 
                                  paste0("chr",c(1:19) ),
                                  which_file="balanced"  )
```

```
## [1] "chr1"
## [1] "chr2"
## [1] "chr3"
## [1] "chr4"
## [1] "chr5"
## [1] "chr6"
## [1] "chr7"
## [1] "chr8"
## [1] "chr9"
## [1] "chr10"
## [1] "chr11"
## [1] "chr12"
## [1] "chr13"
## [1] "chr14"
## [1] "chr15"
## [1] "chr16"
## [1] "chr17"
## [1] "chr18"
## [1] "chr19"
```

```
pe3_loops_IPF_sig = ProcessLoops( pe3_loops_IPF )

pb6_loops_IPF = GetCentroidSignal( loops_a1_a3, 
                                  pb6, 10, 
                                  paste0("chr",c(1:19) ),
                                  which_file="balanced"  )
```

```
## [1] "chr1"
## [1] "chr2"
## [1] "chr3"
## [1] "chr4"
## [1] "chr5"
## [1] "chr6"
## [1] "chr7"
## [1] "chr8"
## [1] "chr9"
## [1] "chr10"
## [1] "chr11"
## [1] "chr12"
## [1] "chr13"
## [1] "chr14"
## [1] "chr15"
## [1] "chr16"
## [1] "chr17"
## [1] "chr18"
## [1] "chr19"
```

```
pb6_loops_IPF_sig = ProcessLoops( pb6_loops_IPF )


A3_loop_signal2 = unlist(lapply(a3_2_loops_IPF_sig,function(x){sum(x[i:j,i:j],
                                                                  na.rm = TRUE)}))
PE3_loop_IPF_signal = unlist(lapply(pe3_loops_IPF_sig,function(x){sum(x[i:j,i:j],
                                                                  na.rm = TRUE)}))
PB6_loop_IPF_signal = unlist(lapply(pb6_loops_IPF_sig,function(x){sum(x[i:j,i:j],
                                                                  na.rm = TRUE)}))

all(names(A3_loop_signal2)==names(PE3_loop_IPF_signal) & names(PE3_loop_IPF_signal)==names(PB6_loop_IPF_signal))
```

```
## [1] TRUE
```

```
ipf_pantr1 = data.frame(a3=A3_loop_signal2,
                        pe3 = PE3_loop_IPF_signal,
                        pb6 = PB6_loop_IPF_signal,
                        row.names = names(A3_loop_signal2))

## random intervals
a3_2_random_loops_IPF = GetCentroidSignal( random_loops, 
                                    A3_rep2, 10, 
                                    paste0("chr",c(1:19) ),
                                    which_file="balanced"  )
```

```
## [1] "chr1"
## [1] "chr2"
## [1] "chr3"
## [1] "chr4"
## [1] "chr5"
## [1] "chr6"
## [1] "chr7"
## [1] "chr8"
## [1] "chr9"
## [1] "chr10"
## [1] "chr11"
## [1] "chr12"
## [1] "chr13"
## [1] "chr14"
## [1] "chr15"
## [1] "chr16"
## [1] "chr17"
## [1] "chr18"
## [1] "chr19"
```

```
a3_2_random_loops_IPF_sig = ProcessLoops( a3_2_random_loops_IPF )

pe3_random_loops_IPF = GetCentroidSignal( random_loops, 
                                  pe3, 10, 
                                  paste0("chr",c(1:19) ),
                                  which_file="balanced"  )
```

```
## [1] "chr1"
## [1] "chr2"
## [1] "chr3"
## [1] "chr4"
## [1] "chr5"
## [1] "chr6"
## [1] "chr7"
## [1] "chr8"
## [1] "chr9"
## [1] "chr10"
## [1] "chr11"
## [1] "chr12"
## [1] "chr13"
## [1] "chr14"
## [1] "chr15"
## [1] "chr16"
## [1] "chr17"
## [1] "chr18"
## [1] "chr19"
```

```
pe3_random_loops_IPF_sig = ProcessLoops( pe3_random_loops_IPF )

pb6_random_loops_IPF = GetCentroidSignal( random_loops, 
                                  pb6, 10, 
                                  paste0("chr",c(1:19) ),
                                  which_file="balanced"  )
```

```
## [1] "chr1"
## [1] "chr2"
## [1] "chr3"
## [1] "chr4"
## [1] "chr5"
## [1] "chr6"
## [1] "chr7"
## [1] "chr8"
## [1] "chr9"
## [1] "chr10"
## [1] "chr11"
## [1] "chr12"
## [1] "chr13"
## [1] "chr14"
## [1] "chr15"
## [1] "chr16"
## [1] "chr17"
## [1] "chr18"
## [1] "chr19"
```

```
pb6_random_loops_IPF_sig = ProcessLoops( pb6_random_loops_IPF )
A3_loop_random_signal2 = unlist(lapply(a3_2_random_loops_IPF_sig,function(x){sum(x[i:j,i:j],
                                                                  na.rm = TRUE)}))
PE3_loop_random_signal = unlist(lapply(pe3_random_loops_IPF_sig,function(x){sum(x[i:j,i:j],
                                                                  na.rm = TRUE)}))
PB6_loop_random_signal = unlist(lapply(pb6_random_loops_IPF_sig,function(x){sum(x[i:j,i:j],
                                                                  na.rm = TRUE)}))

ipf_pantr1_ran = data.frame(a3=A3_loop_random_signal2,
                        pe3 = PE3_loop_random_signal,
                        pb6 = PB6_loop_random_signal,
                        row.names = names(A3_loop_random_signal2))
```

```
write.table( ipf_pantr1_ran, file=paste0(data_directory,"ipf_pantr1_ran.txt"),
             quote=FALSE, row.names=TRUE, col.names = TRUE,
             sep="\t" )
write.table( ipf_pantr1, file=paste0(data_directory,"ipf_pantr1.txt"),
             quote=FALSE, row.names=TRUE, col.names = TRUE,
             sep="\t" )
```

Loop signal - impact of Pantr1

```
loop_signal_pantr1 = data.frame( a3 = getLoopSignal(a3_2_loops_IPF_sig,2),
                                 pe3 = getLoopSignal(pe3_loops_IPF_sig,2),
                                 pb6 = getLoopSignal(pb6_loops_IPF_sig,2))
real_loops_pantr1 = rownames( loop_signal_pantr1[loop_signal_pantr1$a3 >1,] )
real_loops_pantr1 = real_loops_pantr1[real_loops_pantr1 %in% rownames(loops_a1_a3[rowSums(loops_a1_a3[,c("left_CTCF","right_CTCF")])==2 ,])]

a3_ls = loop_signal_pantr1[match( real_loops_pantr1,rownames(loop_signal_pantr1)),"a3"]
pantr1_ls = rowMeans( loop_signal_pantr1[match( real_loops_pantr1,rownames(loop_signal_pantr1)),c("pe3","pb6")])


loops_gained_pantr = ipf_pantr1[which( ipf_pantr1$pe3>ipf_pantr1$a3 & ipf_pantr1$pb6>ipf_pantr1$a3 & loop_signal_pantr1$pe3>loop_signal_pantr1$a3 & loop_signal_pantr1$pb6>loop_signal_pantr1$a3),]

loops_lost_pantr = ipf_pantr1[which( ipf_pantr1$pe3<ipf_pantr1$a3 & ipf_pantr1$pb6<ipf_pantr1$a3 & loop_signal_pantr1$pe3<loop_signal_pantr1$a3 & loop_signal_pantr1$pb6<loop_signal_pantr1$a3),]

par(mfrow=c(1,1),mar=c(5,5,4,1),pty="m")
barplot( c(nrow(loops_lost_pantr),nrow(loops_gained_pantr)),
         col = c("gray40","green3"), ylim=c(0,4000),
         names=c("lost","gained"),main="Loop change upon Pantr1 loss")
axis(2,lwd=2)
```

```
save( loop_signal_pantr1, real_loops_pantr1, a3_ls, pantr1_ls,
      loops_gained_pantr, loops_lost_pantr, file=paste0(data_directory,"pantr1_loops_singals.RData") )
```

```
write.table( loops_lost_pantr,file=paste0(data_directory,"loops_lost_pantr.txt"),
             quote=FALSE,row.names=TRUE, col.names = TRUE,
             sep="\t" )
write.table( loops_gained_pantr,file=paste0(data_directory,"loops_gained_pantr.txt"),
             quote=FALSE,row.names=TRUE, col.names = TRUE,
             sep="\t" )
```

```
load(paste0(data_directory,"pantr1_loops_singals.RData"))
fc_Pantr1 = log2(rowMeans(ipf_pantr1[match(real_loops_pantr1,rownames(ipf_pantr1)),2:3 ])/((ipf_pantr1$a3[match(real_loops_pantr1,rownames(ipf_pantr1)) ])))
sig_Pantr = cut( ipf_pantr1$a3[match(real_loops_pantr1,rownames(ipf_pantr1))],
                c(0,0.04,0.08,0.16,0.32,Inf),
                labels=FALSE )

table(sig_Pantr)
```

```
## sig_Pantr
##    1    2    3    4    5 
##  192  663 1212  677  140
```

```
fcs2 = split(fc_Pantr1,sig_Pantr)
save(fc_Pantr1,sig_Pantr, fcs2, file=paste0(data_directory,"Pantr1_arch_loop_strength.RData"))
```

```
load(paste0(data_directory,"Pantr1_arch_loop_strength.RData"))
par(pty="m")
boxplot(fcs2,outline=FALSE,notch=TRUE, col="white",
        border=colorRampPalette(c("green4","blue4"))(5),lwd=2,
        ylim=c(-1,1), ylab="LFC (Mut vs. Pantr1-/-)")
abline(h=0,lwd=1,lty=2)
axis(1,lwd=2)
axis(2,lwd=2)
box(col="black",lwd=2)
```

```
t.test(fcs2[[1]])
```

```
## 
##  One Sample t-test
## 
## data:  fcs2[[1]]
## t = 1.0192, df = 191, p-value = 0.3094
## alternative hypothesis: true mean is not equal to 0
## 95 percent confidence interval:
##  -0.02758709  0.08657211
## sample estimates:
##  mean of x 
## 0.02949251
```

```
t.test(fcs2[[2]])
```

```
## 
##  One Sample t-test
## 
## data:  fcs2[[2]]
## t = -13.473, df = 662, p-value < 2.2e-16
## alternative hypothesis: true mean is not equal to 0
## 95 percent confidence interval:
##  -0.1970322 -0.1469062
## sample estimates:
##  mean of x 
## -0.1719692
```

```
t.test(fcs2[[3]])
```

```
## 
##  One Sample t-test
## 
## data:  fcs2[[3]]
## t = -44.041, df = 1211, p-value < 2.2e-16
## alternative hypothesis: true mean is not equal to 0
## 95 percent confidence interval:
##  -0.3044588 -0.2784897
## sample estimates:
##  mean of x 
## -0.2914742
```

```
t.test(fcs2[[4]])
```

```
## 
##  One Sample t-test
## 
## data:  fcs2[[4]]
## t = -74.595, df = 676, p-value < 2.2e-16
## alternative hypothesis: true mean is not equal to 0
## 95 percent confidence interval:
##  -0.4474995 -0.4245455
## sample estimates:
##  mean of x 
## -0.4360225
```

```
t.test(fcs2[[5]])
```

```
## 
##  One Sample t-test
## 
## data:  fcs2[[5]]
## t = -23.774, df = 139, p-value < 2.2e-16
## alternative hypothesis: true mean is not equal to 0
## 95 percent confidence interval:
##  -0.4491845 -0.3802065
## sample estimates:
##  mean of x 
## -0.4146955
```

```
t.test(fcs2[[1]],fcs2[[5]])
```

```
## 
##  Welch Two Sample t-test
## 
## data:  fcs2[[1]] and fcs2[[5]]
## t = 13.146, df = 300.5, p-value < 2.2e-16
## alternative hypothesis: true difference in means is not equal to 0
## 95 percent confidence interval:
##  0.3776949 0.5106812
## sample estimates:
##   mean of x   mean of y 
##  0.02949251 -0.41469552
```

```
unlist(lapply(fcs2, length))
```

```
##    1    2    3    4    5 
##  192  663 1212  677  140
```

```
theNtimes = max(unlist(lapply(fcs2,length)))
df = data.frame( gr1=rep(NA,theNtimes),
                 gr2=rep(NA,theNtimes),
                 gr3=rep(NA,theNtimes),
                 gr4=rep(NA,theNtimes),
                 gr5=rep(NA,theNtimes))
df$gr1[1:length(fcs2[[1]])] = fcs2[[1]]
df$gr2[1:length(fcs2[[2]])] = fcs2[[2]]
df$gr3[1:length(fcs2[[3]])] = fcs2[[3]]
df$gr4[1:length(fcs2[[4]])] = fcs2[[4]]
df$gr5[1:length(fcs2[[5]])] = fcs2[[5]]
write.table(df,file=paste0(source_data_directory,"boxplots_arch_Loop_signal_FC_Pantr1.txt"),
            row.names = FALSE, sep="\t",quote=FALSE)
```

# CTCF in Pantr1-/-

```
load(paste0(objects_directory,'ctcf_NS_ranges.RData'))
pe3_1_ctcf_rpgc = import.bw(paste0(chipseq_directory,"ChIP_Seq_CTCF_10-24_MusMus_es-NPC_PANTR1_KO_CTCF-Cterm_HALO_Pantr1_KO_NPC_Pantr1_KO_PE3_Rep_1_RPGC.bw"))
pb6_2_ctcf_rpgc = import.bw(paste0(chipseq_directory,"ChIP_Seq_CTCF_10-24_MusMus_es-NPC_PANTR1_KO_CTCF-Cterm_HALO_Pantr1_KO_NPC_Pantr1_KO_PB6_Rep_1_RPGC.bw"))
seqlevelsStyle(pe3_1_ctcf_rpgc) = "ucsc"
seqlevelsStyle(pb6_2_ctcf_rpgc) = "ucsc"

pe3_1_ctcf_rpgc_AP = getSignalInBins(ctcf_NS_ranges,pe3_1_ctcf_rpgc,1)
pb6_1_ctcf_rpgc_AP = getSignalInBins(ctcf_NS_ranges,pb6_2_ctcf_rpgc,1)

a=90;b=110
pantr_ctcf = data.frame( wt1 = ( rowSums(a3_1_ctcf_rpgc_ctcf_AP[,a:b])),
                         wt2 = ( rowSums(a3_2_ctcf_rpgc_ctcf_AP[,a:b])),
                         pe3 = ( rowSums(pe3_1_ctcf_rpgc_AP[,a:b])),
                         pb6 = ( rowSums(pb6_1_ctcf_rpgc_AP[,a:b])),
                         row.names = rownames(a3_1_ctcf_rpgc_ctcf_AP))

peaks_lost_in_Pantr = which( pantr_ctcf$wt1>1.25*pantr_ctcf$pe3 & pantr_ctcf$wt2>1.25*pantr_ctcf$pe3 & pantr_ctcf$wt1>1.25*pantr_ctcf$pb6 & pantr_ctcf$wt2>1.25*pantr_ctcf$pb6 )

peaks_gained_in_Pantr = which( 1.25*pantr_ctcf$wt1<pantr_ctcf$pe3 & 1.25*pantr_ctcf$wt2<pantr_ctcf$pe3 & 1.25*pantr_ctcf$wt1<pantr_ctcf$pb6 & 1.25*pantr_ctcf$wt2<pantr_ctcf$pb6 )

peaks_at_loops_lost_in_Pantr = unique( c(unique(queryHits(findOverlaps(ctcf_wt_NS_peaks,anchors_left_big[which(anchors_left_big$names %in% rownames(loops_lost_pantr))]))),
                                 unique(queryHits(findOverlaps(ctcf_wt_NS_peaks,anchors_right_big[which(anchors_right_big$names %in% rownames(loops_lost_pantr))])))))


par(mfrow=c(1,1),mar=c(5,5,4,1),pty="m")
barplot( c(length(peaks_lost_in_Pantr),length(peaks_gained_in_Pantr)),
         col = c("gray40","green3"), ylim=c(0,4000),
         names=c("lost","gained"),main="CTCF peak change")
axis(2,lwd=2)
```

```
boxplot( rowSums(pantr_ctcf),
         rowSums(pantr_ctcf[peaks_lost_in_Pantr,]), 
         rowSums(pantr_ctcf[peaks_gained_in_Pantr,]), 
         outline=FALSE,
         notch=TRUE, col="white", border=c("black","gray","green3"),
         lwd=2)
axis(1,lwd=2, at=c(1,2,3))
axis(2,lwd=2)
box(col="black",lwd=2)
```

```
theNtimes = max(c(length(rowSums(pantr_ctcf)),
                  length(rowSums(pantr_ctcf[peaks_lost_in_Pantr,])), 
                  length(rowSums(pantr_ctcf[peaks_gained_in_Pantr,]))))
df = data.frame( All=rep(NA,theNtimes),
                 Lost=rep(NA,theNtimes),
                 Gained=rep(NA,theNtimes))
df$All[1:length((rowSums(pantr_ctcf)))] = (rowSums(pantr_ctcf))
df$Lost[1:length((rowSums(pantr_ctcf[peaks_lost_in_Pantr,])))] = (rowSums(pantr_ctcf[peaks_lost_in_Pantr,]))
df$Gained[1:length((rowSums(pantr_ctcf[peaks_gained_in_Pantr,])))] = (rowSums(pantr_ctcf[peaks_gained_in_Pantr,]))

write.table(df,file=paste0(source_data_directory,"boxplots_CTCF_signal__Pantr1KO.txt"),row.names = FALSE, sep="\t",quote=FALSE)
```

```
write.table( pantr_ctcf, file=paste0(data_directory,"pantr_ctcf.txt"),
             quote=FALSE, row.names=FALSE, col.names = TRUE,
             sep="\t" )
sum(rownames(lost_loops) %in% rownames(loops_lost_pantr))
```

```
## [1] 502
```

```
sum(rownames(gained_loops) %in% rownames(loops_gained_pantr))
```

```
## [1] 11
```

```
sum(peaks_lost_in_Pantr %in%  peaks_at_loops_lost_in_Pantr)
```

```
## [1] 530
```

```
lost_peal_lost_loop_Pantr = data.frame( left=countOverlaps(anchors_left_big[which(anchors_left_big$names %in% rownames(loops_lost_pantr))],ctcf_wt_NS_peaks[ peaks_lost_in_Pantr]),
                                        right=countOverlaps(anchors_right_big[which(anchors_right_big$names %in% rownames(loops_lost_pantr))],ctcf_wt_NS_peaks[peaks_lost_in_Pantr]))
```

# APA in Pantr1 on loops lost in Ddx5

```
lostInAll = which( ipf$a1>ipf$cb1 & ipf$a1>ipf$ce10 & ipf$a3>ipf$cb1 & ipf$a3>ipf$ce10 & ipf_pantr1$a3>ipf_pantr1$pe3 & ipf_pantr1$a3>ipf_pantr1$pb6 & degron_ipf$dmso>degron_ipf$dtag )

gainedInAll = which( ipf$a1<ipf$cb1 & ipf$a1<ipf$ce10 & ipf$a3<ipf$cb1 & ipf$a3<ipf$ce10 & ipf_pantr1$a3<ipf_pantr1$pe3 & ipf_pantr1$a3<ipf_pantr1$pb6 & degron_ipf$dmso<degron_ipf$dtag )

write.table( rownames(ipf)[lostInAll], file=paste0(data_directory,"lostInAll.txt"),
             quote=FALSE, row.names=FALSE, col.names = TRUE,
             sep="\t" )

write.table( rownames(ipf)[gainedInAll], file=paste0(data_directory,"gainedInAll.txt"),
             quote=FALSE, row.names=FALSE, col.names = TRUE,
             sep="\t" )
```

Strong loops are affected by Pantr1/Ddx5

```
load(paste0(data_directory,"IPF_loops.RData"))
loop_strength = rowMeans(cbind(ipf$a1,ipf$a3,ipf_pantr1$a3))
names(loop_strength) = rownames(ipf)
boxplot( loop_strength[names(loop_strength) %in% rownames(ipf)[lostInAll]],
         loop_strength[names(loop_strength) %in% rownames(ipf)[gainedInAll]],
         loop_strength[names(loop_strength) %in% rownames(ipf)[-c(lostInAll,gainedInAll)]],
         outline=FALSE, notch=TRUE,
         names=c("Lost","Gained","Other"),
         col="white",
         border=c("gray","green3","black"),lwd=2,ylab="Hi-C IPF signal")
axis(1,lwd=1,at=c(1,2,3),c("Lost","Gained","Other"))
axis(2,lwd=1)
```

```
length(loop_strength[names(loop_strength) %in% rownames(ipf)[lostInAll]])
```

```
## [1] 1916
```

```
length(loop_strength[names(loop_strength) %in% rownames(ipf)[gainedInAll]])
```

```
## [1] 21
```

```
length(loop_strength[names(loop_strength) %in% rownames(ipf)[-c(lostInAll,gainedInAll)]])
```

```
## [1] 10617
```

```
theNtimes = max(c(length(lostInAll),length(gainedInAll), length(rownames(ipf)[-c(lostInAll,gainedInAll)])))
df = data.frame( lost=rep(NA,theNtimes),
                 gained=rep(NA,theNtimes),
                 other=rep(NA,theNtimes))
df$lost[1:length(loop_strength[names(loop_strength) %in% rownames(ipf)[lostInAll]])] = loop_strength[names(loop_strength) %in% rownames(ipf)[lostInAll]]
df$gained[1:length(loop_strength[names(loop_strength) %in% rownames(ipf)[gainedInAll]])] =  loop_strength[names(loop_strength) %in% rownames(ipf)[gainedInAll]]  
df$other[1:length(loop_strength[names(loop_strength) %in% rownames(ipf)[-c(lostInAll,gainedInAll)]])] =  loop_strength[names(loop_strength) %in% rownames(ipf)[-c(lostInAll,gainedInAll)]]                                                                                   
write.table(df,file=paste0(source_data_directory,"boxplots_Loop_signal__loops_affected_by_Pantr1KO.txt"),
            row.names = FALSE, sep="\t",quote=FALSE)
```

# Hi-C: Insulation

```
getSUMMEDsignal4bins = function( B, D, A, M ){
      # B=bins-HOWFAR; D=distance; A=Area; M=thism
    rows = unlist(lapply(as.list(B), function(b){
    rep( ( (b-D) + seq(-A,A)) , (1+2*A))}))
    
    cols = unlist(lapply(as.list(B), function(b){
    rep( ( (b+D) + seq(-A,A) ), each=(1+2*A))}))
    ids = cbind(rows, cols)
    m = M[ids]
    IDS = rep( names(B), each = (1+2*A)^2 )
    # print(c(min(ids),max(ids)))
    matrices = split( m, IDS )
    return( unlist(lapply(matrices,function(m){sum(m, na.rm=T)})) )}
InsulationScore = function( bin, mat, GAGR, distance, Area, HOWFAR ){
  ## mat=ko_10kb; distance=5; Area=3;chr="chr1";GAGR=gagr;HOWFAR=10
  ## bin = gagr[which(chrom(gagr)!="chrY")]
  ## rm(list=c("mat","distance","Area","chr","GAGR","HOWFAR","B","D","A","M"))
  do.call("rbind",lapply( as.list(unique(as.character(chrom(bin)))),function(chr){
    print(paste0("processing ",chr))
    thism = mat[[chr]]$balanced
    theseb = GenomicRanges::resize( bin[which(chrom(bin)==chr)], 1, fix="center")
    
    bins = GAGR$binid[queryHits(findOverlaps(GAGR,theseb))]
    names(bins) = names(theseb[subjectHits(findOverlaps(GAGR,theseb))])
    
    bins = bins[bins-distance-HOWFAR-Area>0 & bins+distance+HOWFAR+Area<(length(GAGR[which(chrom(GAGR)==chr)]))]
    
    return(data.frame( left=getSUMMEDsignal4bins(B=bins-HOWFAR, D=distance, A=Area, M=thism ),
                       middle=getSUMMEDsignal4bins(B=bins, D=distance, A=Area, M=thism ),
                       right=getSUMMEDsignal4bins(B=bins+HOWFAR, D=distance, A=Area, M=thism ) )) } )) }
InsulationScorePublishedKR = function( bin, mat, GAGR, distance, Area, HOWFAR ){
  ## mat=ko_10kb; distance=5; Area=3;chr="chr1";GAGR=gagr;HOWFAR=10
  ## bin = gagr[which(chrom(gagr)!="chrY")]
  ## rm(list=c("mat","distance","Area","chr","GAGR","HOWFAR","B","D","A","M"))
  do.call("rbind",lapply( as.list(unique(as.character(chrom(bin)))),function(chr){
    print(paste0("processing ",chr))
    thism = mat[[chr]]
    theseb = GenomicRanges::resize( bin[which(chrom(bin)==chr)], 1, fix="center")
    
    bins = GAGR$binid[queryHits(findOverlaps(GAGR,theseb))]
    names(bins) = names(theseb[subjectHits(findOverlaps(GAGR,theseb))])
    
    bins = bins[bins-distance-HOWFAR-Area>0 & bins+distance+HOWFAR+Area<(length(GAGR[which(chrom(GAGR)==chr)]))]
    
    return(data.frame( left=getSUMMEDsignal4bins(B=bins-HOWFAR, D=distance, A=Area, M=thism ),
                       middle=getSUMMEDsignal4bins(B=bins, D=distance, A=Area, M=thism ),
                       right=getSUMMEDsignal4bins(B=bins+HOWFAR, D=distance, A=Area, M=thism ) )) } )) }
processIS = function( IS, GAGR ){
  res = GAGR
  res$binid=NULL
  res$score = 0
  res$score[match(rownames(IS),names(res))] = log2( rowMeans((0.001+IS[,c(1,3)]))/(0.001+IS[,2] ) )
  return(res) }
cleanIS = function(x){
  x[is.na(x)]=0
  x[!is.finite(x)]=0
  return(x) }
```

Genome wide insulation score - prepare gagr again

```
gagr = GRanges(seqnames = Rle(ga$chr),
                 ranges = IRanges(as.numeric(ga$start),
                                  end = as.numeric(ga$end),
                                  names = seq(1, nrow(ga))),
                 strand = Rle(rep("*", nrow(ga))),
               binid = ga$binid,
               seqlengths = seqlengths(BSgenome.Mmusculus.UCSC.mm10))
```

```
## Warning in valid.GenomicRanges.seqinfo(x, suggest.trim = TRUE): GRanges object contains 21 out-of-bound ranges located on sequences
##   chr1, chr2, chr3, chr4, chr5, chr6, chr7, chr8, chr9, chr10, chr11,
##   chr12, chr13, chr14, chr15, chr16, chr17, chr18, chr19, chrX, and chrY.
##   Note that ranges located on a sequence whose length is unknown (NA) or
##   on a circular sequence are not considered out-of-bound (use
##   seqlengths() and isCircular() to get the lengths and circularity flags
##   of the underlying sequences). You can use trim() to trim these ranges.
##   See ?`trim,GenomicRanges-method` for more information.
```

```
gagr = trim(gagr)
names(gagr) = paste(chrom(gagr),names(gagr),sep="_")
```

```
load(paste0( hic_directory,"ko_10kb.RData"))
load(paste0( hic_directory,"wt_A1_A3_10kb.RData"))
names(wt_10kb) = paste0("chr",c(1:19,"X"))
names(ko_10kb) = paste0("chr",c(1:19,"X"))

# 5,1,10
genome_wide_IS_wt = InsulationScore( gagr[which(chrom(gagr)!="chrY")], 
                                     wt_10kb, 
                                     gagr, 5, 1, 15 )
genome_wide_IS_ko = InsulationScore( gagr[which(chrom(gagr)!="chrY")], 
                                     ko_10kb, gagr, 5, 1, 15 )

save(genome_wide_IS_wt,file=paste0(objects_directory,"genome_wide_IS__wt.RData"))
save(genome_wide_IS_ko,file=paste0(objects_directory,"genome_wide_IS__ko.RData"))
```

Insulation at loop anchors - is it affected?

```
load(paste0(objects_directory,"genome_wide_IS__wt.RData"))
load(paste0(objects_directory,"genome_wide_IS__ko.RData"))
```

```
wt_is_genome_wide = log2(rowMeans(genome_wide_IS_wt[,c(1,3)])/genome_wide_IS_wt[,2])
ko_is_genome_wide = log2(rowMeans(genome_wide_IS_ko[,c(1,3)])/genome_wide_IS_ko[,2])

wt_is_genome_wide = cleanIS(wt_is_genome_wide)
ko_is_genome_wide = cleanIS(ko_is_genome_wide)

wt_is_genome_wide_gr = gagr[which(chrom(gagr)!='chrY')]
wt_is_genome_wide_gr$score=0
wt_is_genome_wide_gr$score[match(names(wt_is_genome_wide),names(wt_is_genome_wide_gr))] = wt_is_genome_wide
wt_is_genome_wide_gr$binid=NULL


ko_is_genome_wide_gr = gagr[which(chrom(gagr)!='chrY')]
ko_is_genome_wide_gr$score=0
ko_is_genome_wide_gr$score[match(names(ko_is_genome_wide),names(ko_is_genome_wide_gr))] = ko_is_genome_wide
ko_is_genome_wide_gr$binid=NULL

export.bw( wt_is_genome_wide_gr,con=paste0(objects_directory,"wt_is_genome_wide_gr.bw"))
export.bw( ko_is_genome_wide_gr,con=paste0(objects_directory,"ko_is_genome_wide_gr.bw"))
```

IS at peaks of insulation in the wild type cells

```
is_peaks = wt_is_genome_wide_gr[which(wt_is_genome_wide_gr$score>0.75)]
is_peaks_filt = GenomicRanges::reduce(is_peaks)
is_peaks_filt = is_peaks_filt[which(width(is_peaks_filt)>2)]
is_peaks_filt_binid = as.data.frame( findOverlaps(is_peaks_filt,gagr) )

is_peaks_filt_binid$wt = wt_is_genome_wide_gr$score[is_peaks_filt_binid$subjectHits]
is_peaks_filt_binid$ko = ko_is_genome_wide_gr$score[is_peaks_filt_binid$subjectHits]
is_peaks_filt_binid$wt_rand = wt_is_genome_wide_gr$score[is_peaks_filt_binid$subjectHits-10]
is_peaks_filt_binid$ko_rand = ko_is_genome_wide_gr$score[is_peaks_filt_binid$subjectHits-10]

is_peaks_filt_binid_s = split(is_peaks_filt_binid,is_peaks_filt_binid$queryHits)
save(is_peaks_filt_binid_s,file=paste0(objects_directory,'is_peaks_filt_binid_s.RData'))
```

```
load(paste0(objects_directory,'is_peaks_filt_binid_s.RData'))
wt_mut_peaks = unlist(lapply(is_peaks_filt_binid_s,function(x){mean(x$ko-x$wt)}))
wt_mut_random = unlist(lapply(is_peaks_filt_binid_s,function(x){mean(x$ko_rand-x$wt_rand)}))

par(mfrow=c(1,2),pty='m',mar=c(7,5,1,1))
hist(wt_mut_peaks,n=100,
     xlim=c(-3,3),col="steelblue", ylim=c(0,2000),main='',
     xlab='change in insulation\nDdx5 vs.Wt')
axis(1,lwd=2)
axis(2,lwd=2)
abline(v=0,col="red4",lwd=2,lty=2)
boxplot( wt_mut_peaks, wt_mut_random, outline=FALSE, 
         border=c('blue3','gray'), col='white',notch=TRUE,
         names=c('Peaks','random'),
         ylab='change in insulation (Ddx5 vs. Wt)',las=2)
axis(1,lwd=2,at=c(1,2))
axis(2,lwd=2,las=2)
box(col="black",lwd=2)
abline(h=0,col="red4",lwd=2,lty=2)
```

```
length(wt_mut_peaks)
```

```
## [1] 6132
```

```
length(wt_mut_random)
```

```
## [1] 6132
```

```
df = data.frame( wt_mut_peaks=wt_mut_peaks,
                 wt_mut_random=wt_mut_random)
write.table(df,file=paste0(source_data_directory,"insulation_supplement.txt"),row.names = FALSE, sep="\t",quote=FALSE)
```

# Published data - insulation

```
ns_5kb_chr7 = read.hic_files( hic_directory, "bonev_KR_dump/chr7_5kb_NS_KR_matrix_",".txt", 
                              ga5, "chr7" ) 


genome_wide_IS_ns = InsulationScorePublishedKR( gagr5[which(chrom(gagr5)=="chr7")], 
                                                ns_5kb_chr7, 
                                                gagr5, 5, 3, 10 )
ns_is = log2(genome_wide_IS_ns[,2]/rowMeans(genome_wide_IS_ns[,c(1,3)]))
ns_is = cleanIS(ns_is)


ns_is_gr = gagr5[which(chrom(gagr5)=='chr7')]
ns_is_gr$score=0
ns_is_gr$score[match(names(ns_is),names(ns_is_gr))] = ns_is
ns_is_gr$binid=NULL
export.bw( ns_is_gr, con=paste0( hic_directory,"bonev_KR_dump/IS_chr7_NS_5kb1.bw" ))
```

```
ns_is_gr = import.bw(paste0( hic_directory,"bonev_KR_dump/IS_chr7_NS_5kb1.bw" ))
plot(start(ns_is_gr[(65700/5):(66900/5)]),
     ns_is_gr[(65700/5):(66900/5)]$score,
     ty="l",xaxs="i",ylab="Insulation",xlab="chr7")
abline(h=0)
```

# Pantr1 Insulation

```
rm(list=c("wt_10kb","ko_10kb"))
```

```
## Warning in rm(list = c("wt_10kb", "ko_10kb")): nie znaleziono obiektu 'wt_10kb'
```

```
## Warning in rm(list = c("wt_10kb", "ko_10kb")): nie znaleziono obiektu 'ko_10kb'
```

```
load(paste0( hic_directory,"A3_rep2_10kb_hic.RData" ) ) # A3_rep2
load(paste0( hic_directory,"PB6_10kb_hic.RData" ) ) # pb6
load(paste0( hic_directory,"PE3_10kb_hic.RData" ) ) # Treated
pe3=Treated
rm(list=c("Treated"))
gc()
```

```
##              used    (Mb) gc trigger    (Mb) limit (Mb)   max used    (Mb)
## Ncells   28629340  1529.0   88257021  4713.5         NA   88257021  4713.5
## Vcells 5133133149 39162.7 7581327462 57841.0     131072 7422216756 56627.1
```

```
gagr = GRanges(seqnames = Rle(ga$chr),
                 ranges = IRanges(as.numeric(ga$start),
                                  end = as.numeric(ga$end),
                                  names = seq(1, nrow(ga))),
                 strand = Rle(rep("*", nrow(ga))),
               binid = ga$binid,
               seqlengths = seqlengths(BSgenome.Mmusculus.UCSC.mm10))
gagr = trim(gagr)
names(gagr) = paste(chrom(gagr),names(gagr),sep="_")
genome_wide_IS_a3 = InsulationScore( gagr[which(chrom(gagr)!="chrY")], 
                                     A3_rep2, 
                                     gagr, 5, 1, 15 )
genome_wide_IS_p3 = InsulationScore( gagr[which(chrom(gagr)!="chrY")], 
                                     pe3, 
                                     gagr, 5, 1, 15 )
genome_wide_IS_p6 = InsulationScore( gagr[which(chrom(gagr)!="chrY")], 
                                     pb6, 
                                     gagr, 5, 1, 15 )

save(genome_wide_IS_a3,file=paste0(objects_directory,"genome_wide_IS_a3.RData"))
save(genome_wide_IS_p3,file=paste0(objects_directory,"genome_wide_IS_p3.RData"))
save(genome_wide_IS_p6,file=paste0(objects_directory,"genome_wide_IS_p6.RData"))
```

```
a3_is_genome_wide = log2(rowMeans(genome_wide_IS_a3[,c(1,3)])/genome_wide_IS_a3[,2])
p3_is_genome_wide = log2(rowMeans(genome_wide_IS_p3[,c(1,3)])/genome_wide_IS_p3[,2])
p6_is_genome_wide = log2(rowMeans(genome_wide_IS_p6[,c(1,3)])/genome_wide_IS_p6[,2])

a3_is_genome_wide = cleanIS(a3_is_genome_wide)
p3_is_genome_wide = cleanIS(p3_is_genome_wide)
p6_is_genome_wide = cleanIS(p6_is_genome_wide)

getbwFile = function( iso, binannoF ){
  tp = binannoF[which(chrom(binannoF)!='chrY')]
  tp$score=0
  tp$score[match(names(iso),names(tp))] = iso
  tp$binid=NULL
  return(tp)
}

a3_is_genome_wide_gr = getbwFile( a3_is_genome_wide, gagr )
p3_is_genome_wide_gr = getbwFile( p3_is_genome_wide, gagr )
p6_is_genome_wide_gr = getbwFile( p6_is_genome_wide, gagr )

export.bw( a3_is_genome_wide_gr,con=paste0(objects_directory,"a3_is_genome_wide_gr.bw"))
export.bw( p3_is_genome_wide_gr,con=paste0(objects_directory,"p3_is_genome_wide_gr.bw"))
export.bw( p6_is_genome_wide_gr,con=paste0(objects_directory,"p6_is_genome_wide_gr.bw"))
save( a3_is_genome_wide_gr, p3_is_genome_wide_gr, p6_is_genome_wide_gr,gagr,
      file = paste0( objects_directory,"is_Pantr1.RData" ) )
```

Let’s check if insulation changes at insulators

```
load( paste0( objects_directory,"is_Pantr1.RData" ) )
is_peaks = a3_is_genome_wide_gr[which(a3_is_genome_wide_gr$score>0.75)]
is_peaks_filt = GenomicRanges::reduce(is_peaks)
is_peaks_filt = is_peaks_filt[which(width(is_peaks_filt)>2)]
is_peaks_filt_binid = as.data.frame( findOverlaps(is_peaks_filt,gagr) )

is_peaks_filt_binid$a3 = a3_is_genome_wide_gr$score[is_peaks_filt_binid$subjectHits]
is_peaks_filt_binid$pe3 = p3_is_genome_wide_gr$score[is_peaks_filt_binid$subjectHits]
is_peaks_filt_binid$pb6 = p6_is_genome_wide_gr$score[is_peaks_filt_binid$subjectHits]
is_peaks_filt_binid$a3_rand = a3_is_genome_wide_gr$score[is_peaks_filt_binid$subjectHits-10]
is_peaks_filt_binid$pe3_rand = p3_is_genome_wide_gr$score[is_peaks_filt_binid$subjectHits-10]
is_peaks_filt_binid$pb6_rand = p6_is_genome_wide_gr$score[is_peaks_filt_binid$subjectHits-10]

is_peaks_filt_binid_s = split(is_peaks_filt_binid,is_peaks_filt_binid$queryHits)

a3_p3_peaks = unlist(lapply(is_peaks_filt_binid_s,function(x){mean(x$pe3-x$a3)}))
a3_p3_random = unlist(lapply(is_peaks_filt_binid_s,function(x){mean(x$pe3_rand-x$a3_rand)}))
a3_p6_peaks = unlist(lapply(is_peaks_filt_binid_s,function(x){mean(x$pb6-x$a3)}))
a3_p6_random = unlist(lapply(is_peaks_filt_binid_s,function(x){mean(x$pb6_rand-x$a3_rand)}))

par(mfrow=c(1,1),pty='m',
    mar=c(7,5,3,3))
boxplot( a3_p3_peaks, 
         a3_p3_random,
         a3_p6_peaks, 
         a3_p6_random,
         outline=FALSE, 
         border=c('purple4','gray'), 
         col='white',notch=TRUE,
         names=c('Insulators','random','Insulators','random'),
         ylab='Pantr1 vs. Wt',las=2,lwd=2)
box(col="black",lwd=2)
axis(1,lwd=2,at=c(1,2,3,4), 
     c('Insulators','random','Insulators','random'),las=2)
axis(2,lwd=2,las=2)
abline(h=0,col="red4",lwd=2,lty=2)
```

# SICAP-ChIP

```
es_sicap = read.delim( paste0(data_directory,"ES_SICAP_EMPAIMORETHAN1.txt"))
ns_sicap = read.delim( paste0(data_directory,"NS_SICAP_EMPAIMORETHAN1.txt"))

es_sicap = es_sicap[es_sicap$Benjamini<0.1,]
es_sicap = es_sicap[unlist(lapply(strsplit(es_sicap$Term,":"),function(x){x[[1]]=="GO"})),]

par(mar=c(5,20,1,1))
barplot( rev(-log10(es_sicap$Benjamini[1:30])),
         names=rev(es_sicap$Term[1:30]),las=2,
         horiz=TRUE, col="red")
axis(1,lwd=2,las=2,cex.lab=0.3)
```

```
par(mar=c(5,20,1,1))
barplot( rev(-log10(ns_sicap$Benjamini)[1:10]),
         names=rev(ns_sicap$Term[1:10]),las=2,
         horiz=TRUE, col="blue")
axis(1,lwd=2,las=2)
```

# Session Info

```
sessionInfo()
```

```
## R version 4.1.0 (2021-05-18)
## Platform: x86_64-apple-darwin17.0 (64-bit)
## Running under: macOS Big Sur 10.16
## 
## Matrix products: default
## BLAS:   /Library/Frameworks/R.framework/Versions/4.1/Resources/lib/libRblas.dylib
## LAPACK: /Library/Frameworks/R.framework/Versions/4.1/Resources/lib/libRlapack.dylib
## 
## locale:
## [1] pl_PL.UTF-8/pl_PL.UTF-8/pl_PL.UTF-8/C/pl_PL.UTF-8/pl_PL.UTF-8
## 
## attached base packages:
##  [1] grid      parallel  stats4    stats     graphics  grDevices utils    
##  [8] datasets  methods   base     
## 
## other attached packages:
##  [1] forecast_8.21                            
##  [2] pqsfinder_2.8.0                          
##  [3] sigminer_2.3.1                           
##  [4] ChIPseeker_1.28.3                        
##  [5] motifbreakR_2.6.1                        
##  [6] MotifDb_1.34.0                           
##  [7] ggpubr_0.6.0                             
##  [8] Matrix_1.5-4                             
##  [9] BSgenome.Mmusculus.UCSC.mm10_1.4.0       
## [10] BSgenome_1.60.0                          
## [11] Biostrings_2.60.2                        
## [12] XVector_0.32.0                           
## [13] smoothmest_0.1-3                         
## [14] MASS_7.3-58.3                            
## [15] scales_1.3.0                             
## [16] lubridate_1.9.4                          
## [17] forcats_1.0.0                            
## [18] stringr_1.5.1                            
## [19] purrr_1.0.2                              
## [20] readr_2.1.5                              
## [21] tidyr_1.3.1                              
## [22] tibble_3.2.1                             
## [23] tidyverse_2.0.0                          
## [24] dplyr_1.1.4                              
## [25] TxDb.Mmusculus.UCSC.mm10.knownGene_3.10.0
## [26] GenomicFeatures_1.44.2                   
## [27] pheatmap_1.0.12                          
## [28] RColorBrewer_1.1-3                       
## [29] ggplot2_3.5.1                            
## [30] goseq_1.44.0                             
## [31] geneLenDataBase_1.28.0                   
## [32] BiasedUrn_2.0.12                         
## [33] fgsea_1.18.0                             
## [34] gwasrapidd_0.99.17                       
## [35] ggVennDiagram_1.5.2                      
## [36] vsn_3.60.0                               
## [37] VennDiagram_1.7.3                        
## [38] futile.logger_1.4.3                      
## [39] gplots_3.2.0                             
## [40] biomaRt_2.48.3                           
## [41] geneplotter_1.70.0                       
## [42] annotate_1.70.0                          
## [43] XML_3.99-0.18                            
## [44] lattice_0.22-6                           
## [45] LSD_4.1-0                                
## [46] org.Hs.eg.db_3.13.0                      
## [47] AnnotationDbi_1.54.1                     
## [48] DESeq2_1.32.0                            
## [49] SummarizedExperiment_1.22.0              
## [50] Biobase_2.52.0                           
## [51] MatrixGenerics_1.4.3                     
## [52] matrixStats_1.5.0                        
## [53] sf_1.0-12                                
## [54] rtracklayer_1.52.1                       
## [55] GenomicRanges_1.44.0                     
## [56] GenomeInfoDb_1.28.4                      
## [57] IRanges_2.26.0                           
## [58] S4Vectors_0.30.2                         
## [59] BiocGenerics_0.38.0                      
## 
## loaded via a namespace (and not attached):
##   [1] Hmisc_5.0-1                            
##   [2] class_7.3-23                           
##   [3] Rsamtools_2.8.0                        
##   [4] lmtest_0.9-40                          
##   [5] foreach_1.5.2                          
##   [6] crayon_1.5.3                           
##   [7] nlme_3.1-162                           
##   [8] backports_1.5.0                        
##   [9] GOSemSim_2.18.1                        
##  [10] rlang_1.1.4                            
##  [11] limma_3.48.3                           
##  [12] filelock_1.0.3                         
##  [13] BiocParallel_1.26.2                    
##  [14] rjson_0.2.23                           
##  [15] bit64_4.5.2                            
##  [16] glue_1.8.0                             
##  [17] rngtools_1.5.2                         
##  [18] motifStack_1.36.1                      
##  [19] classInt_0.4-9                         
##  [20] DOSE_3.18.3                            
##  [21] tidyselect_1.2.1                       
##  [22] zoo_1.8-12                             
##  [23] GenomicAlignments_1.28.0               
##  [24] xtable_1.8-4                           
##  [25] magrittr_2.0.3                         
##  [26] evaluate_1.0.1                         
##  [27] quantmod_0.4.27                        
##  [28] cli_3.6.3                              
##  [29] zlibbioc_1.38.0                        
##  [30] rstudioapi_0.17.1                      
##  [31] furrr_0.3.1                            
##  [32] bslib_0.8.0                            
##  [33] rpart_4.1.24                           
##  [34] fastmatch_1.1-6                        
##  [35] ensembldb_2.16.4                       
##  [36] lambda.r_1.2.4                         
##  [37] treeio_1.16.2                          
##  [38] xfun_0.50                              
##  [39] cluster_2.1.8                          
##  [40] urca_1.3-3                             
##  [41] caTools_1.18.3                         
##  [42] tidygraph_1.2.3                        
##  [43] KEGGREST_1.32.0                        
##  [44] ggrepel_0.9.6                          
##  [45] biovizBase_1.40.0                      
##  [46] ape_5.8-1                              
##  [47] listenv_0.9.1                          
##  [48] TFMPvalue_0.0.9                        
##  [49] png_0.1-8                              
##  [50] future_1.34.0                          
##  [51] withr_3.0.2                            
##  [52] bitops_1.0-9                           
##  [53] ggforce_0.4.2                          
##  [54] plyr_1.8.9                             
##  [55] AnnotationFilter_1.16.0                
##  [56] e1071_1.7-16                           
##  [57] pillar_1.10.1                          
##  [58] cachem_1.1.0                           
##  [59] fs_1.6.5                               
##  [60] TTR_0.24.3                             
##  [61] xts_0.13.1                             
##  [62] vctrs_0.6.5                            
##  [63] generics_0.1.3                         
##  [64] NMF_0.28                               
##  [65] tools_4.1.0                            
##  [66] foreign_0.8-87                         
##  [67] munsell_0.5.1                          
##  [68] tweenr_2.0.3                           
##  [69] proxy_0.4-27                           
##  [70] DelayedArray_0.18.0                    
##  [71] fastmap_1.2.0                          
##  [72] compiler_4.1.0                         
##  [73] abind_1.4-8                            
##  [74] TxDb.Hsapiens.UCSC.hg19.knownGene_3.2.2
##  [75] Gviz_1.36.2                            
##  [76] GenomeInfoDbData_1.2.6                 
##  [77] gridExtra_2.3                          
##  [78] deldir_1.0-6                           
##  [79] BiocFileCache_2.0.0                    
##  [80] jsonlite_1.8.9                         
##  [81] affy_1.70.0                            
##  [82] tidytree_0.4.6                         
##  [83] carData_3.0-5                          
##  [84] genefilter_1.74.1                      
##  [85] lazyeval_0.2.2                         
##  [86] tseries_0.10-54                        
##  [87] car_3.1-3                              
##  [88] doParallel_1.0.17                      
##  [89] latticeExtra_0.6-30                    
##  [90] splitstackshape_1.4.8                  
##  [91] checkmate_2.3.2                        
##  [92] rmarkdown_2.29                         
##  [93] cowplot_1.1.3                          
##  [94] dichromat_2.0-0.1                      
##  [95] igraph_1.4.2                           
##  [96] survival_3.8-3                         
##  [97] yaml_2.3.10                            
##  [98] plotrix_3.8-4                          
##  [99] htmltools_0.5.8.1                      
## [100] memoise_2.0.1                          
## [101] VariantAnnotation_1.38.0               
## [102] BiocIO_1.2.0                           
## [103] locfit_1.5-9.10                        
## [104] quadprog_1.5-8                         
## [105] graphlayouts_1.2.1                     
## [106] viridisLite_0.4.2                      
## [107] digest_0.6.37                          
## [108] rappdirs_0.3.3                         
## [109] futile.options_1.0.1                   
## [110] registry_0.5-1                         
## [111] units_0.8-1                            
## [112] RSQLite_2.3.9                          
## [113] yulab.utils_0.1.9                      
## [114] data.table_1.16.4                      
## [115] fracdiff_1.5-2                         
## [116] blob_1.2.4                             
## [117] preprocessCore_1.54.0                  
## [118] splines_4.1.0                          
## [119] Formula_1.2-5                          
## [120] labeling_0.4.3                         
## [121] ProtGenerics_1.24.0                    
## [122] RCurl_1.98-1.16                        
## [123] broom_1.0.7                            
## [124] hms_1.1.3                              
## [125] colorspace_2.1-1                       
## [126] base64enc_0.1-3                        
## [127] BiocManager_1.30.25                    
## [128] aplot_0.1.10                           
## [129] nnet_7.3-20                            
## [130] sass_0.4.9                             
## [131] Rcpp_1.0.13-1                          
## [132] enrichplot_1.12.3                      
## [133] tzdb_0.4.0                             
## [134] parallelly_1.41.0                      
## [135] R6_2.5.1                               
## [136] lifecycle_1.0.4                        
## [137] formatR_1.14                           
## [138] curl_6.1.0                             
## [139] ggsignif_0.6.4                         
## [140] affyio_1.62.0                          
## [141] jquerylib_0.1.4                        
## [142] DO.db_2.9                              
## [143] qvalue_2.24.0                          
## [144] iterators_1.0.14                       
## [145] htmlwidgets_1.6.4                      
## [146] polyclip_1.10-7                        
## [147] shadowtext_0.1.4                       
## [148] timechange_0.3.0                       
## [149] gridGraphics_0.5-1                     
## [150] mgcv_1.9-1                             
## [151] globals_0.16.3                         
## [152] htmlTable_2.4.3                        
## [153] patchwork_1.3.0                        
## [154] codetools_0.2-20                       
## [155] GO.db_3.13.0                           
## [156] gtools_3.9.5                           
## [157] prettyunits_1.2.0                      
## [158] dbplyr_2.5.0                           
## [159] gridBase_0.4-7                         
## [160] gtable_0.3.6                           
## [161] DBI_1.2.3                              
## [162] ggfun_0.0.9                            
## [163] httr_1.4.7                             
## [164] KernSmooth_2.23-20                     
## [165] stringi_1.8.4                          
## [166] progress_1.2.3                         
## [167] reshape2_1.4.4                         
## [168] farver_2.1.2                           
## [169] viridis_0.6.5                          
## [170] fdrtool_1.2.18                         
## [171] timeDate_4041.110                      
## [172] ggtree_3.0.4                           
## [173] xml2_1.3.6                             
## [174] boot_1.3-31                            
## [175] restfulr_0.0.15                        
## [176] interp_1.1-4                           
## [177] ade4_1.7-22                            
## [178] ggplotify_0.1.2                        
## [179] bit_4.5.0.1                            
## [180] scatterpie_0.2.4                       
## [181] jpeg_0.1-10                            
## [182] ggraph_2.2.1                           
## [183] pkgconfig_2.0.3                        
## [184] rstatix_0.7.2                          
## [185] knitr_1.49
```
