# Supplementary material for: RNA-binding proteins mediate the maturation of chromatin topology during differentiation
Source: Nat Cell Biol. 2025 Sep 8;27(9):1510–25. doi: 10.1038/s41556-025-01735-5 (PMC12431861; doi:10.1038/s41556-025-01735-5)
Supplement: Supplementary file 5 — Unprocessed gels or blots. [file 41556_2025_1735_MOESM5_ESM.pdf]

Figure 1

Figure 1fg: Western blot analysis for RBPs Fus (f) and Ddx5 (g) for PLA in WT ES and NS cells

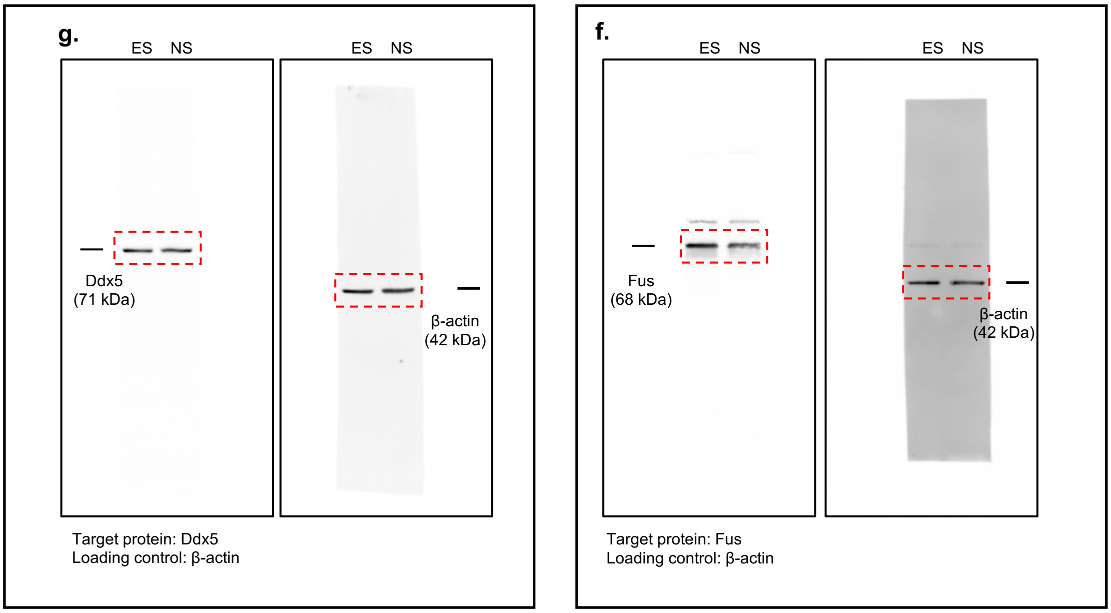

Figure 2

Figure 2d: Agarose gel image for genotyping for homozygous insertion of FKBP-RFP at the Ddx5-Nter locus KI in CTCF-HALO cells

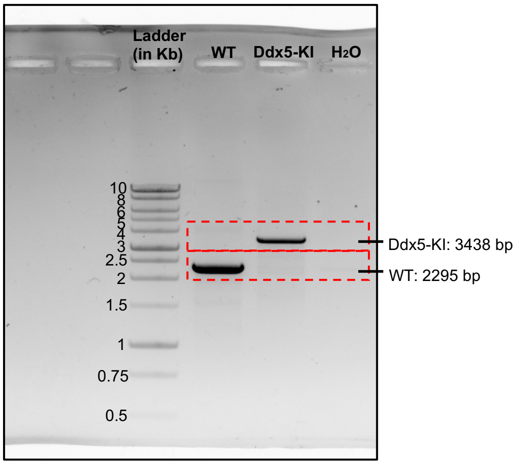

WT: 2295 bp
Ddx5-KI (AM-FKBP_RFP_HA) : 3438 bp
H_2O: Negative control

Figure 2e: Western blot for Ddx5 expression in wild type ES and NS cells.

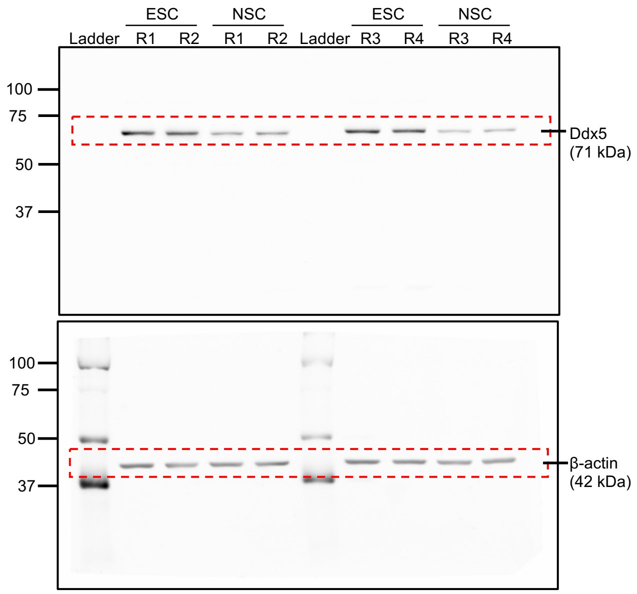

Target protein: Ddx5
Loading control: β-actin

Ddx5- 71 kDa
FKBP- 12.6 kDa
RFP- 27 kDa

Ddx5-KI- 110 kDa

Figure 2e: : Western blot for Ddx5 expression after acute depletion of Ddx5 in NS Ddx5-KI cells

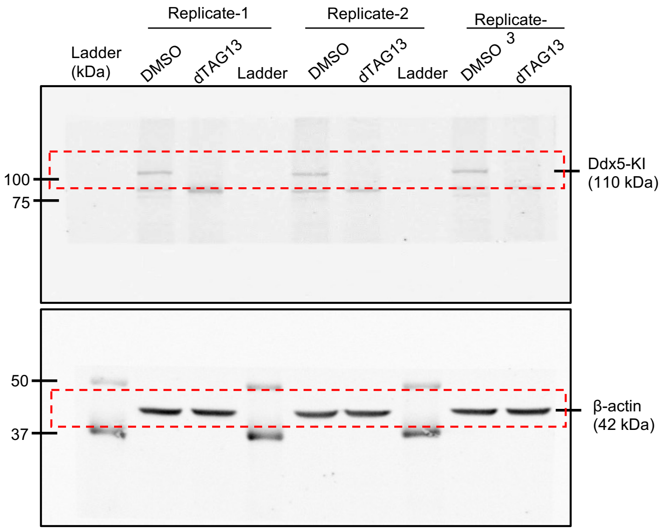

Target protein: Ddx5
Loading control: β-actin

Figure 2e: : Western blot for Ddx5 expression after acute depletion of Ddx5 in ES Ddx5-KI cells

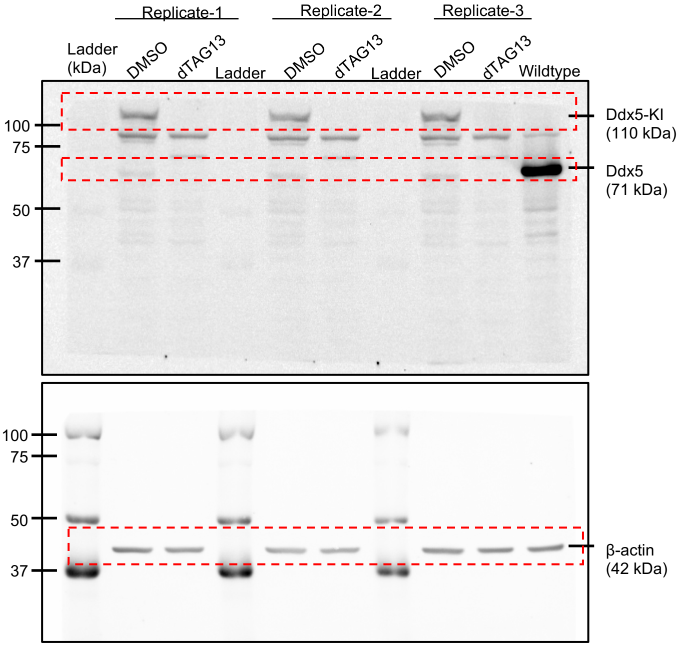

Target protein: Ddx5, Ddx5-KI
Loading control: β-actin

Figure 5

Figure 5k : Co-IP for pulldown of RBP- Fus with CTCF-HALO protein using anti-Halo-M270 beads (Replicate 1)

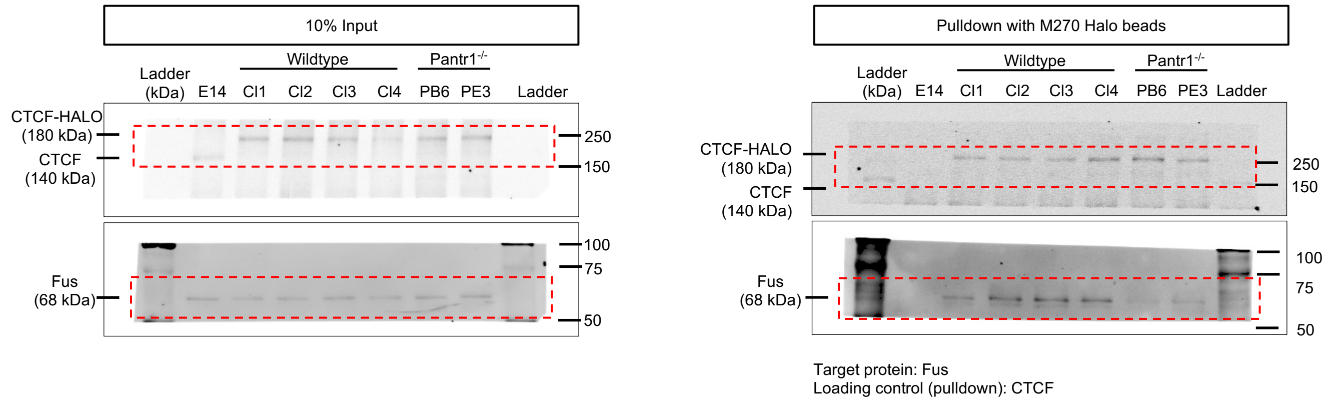

Figure 5k : Co-IP for pulldown of RBP- Fus with CTCF-HALO protein using anti-Halo-M270 beads (Replicate 2)

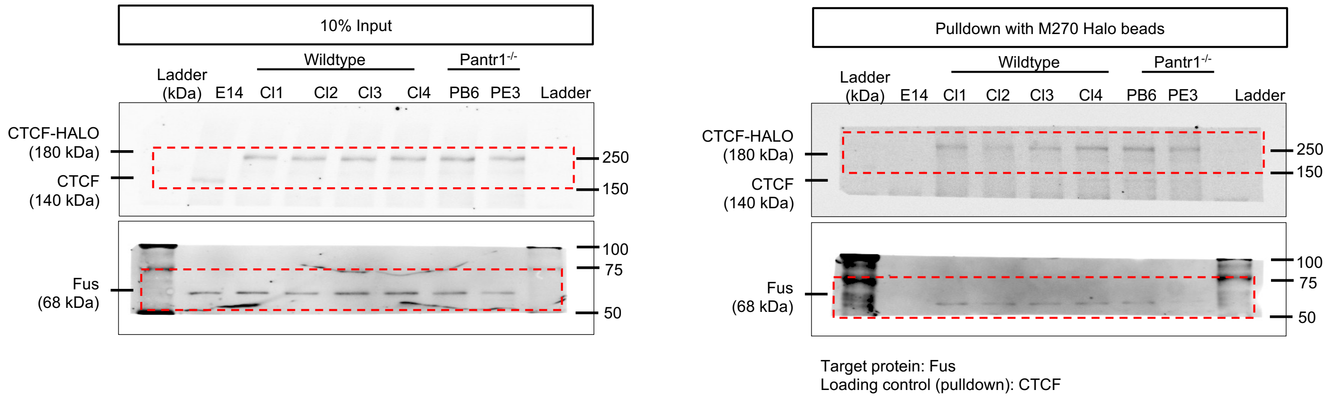

Figure 5

Figure 5k : Co-IP for pulldown of RBP- Ddx5 with CTCF-HALO protein using anti-Halo-M270 beads (Replicate 1)

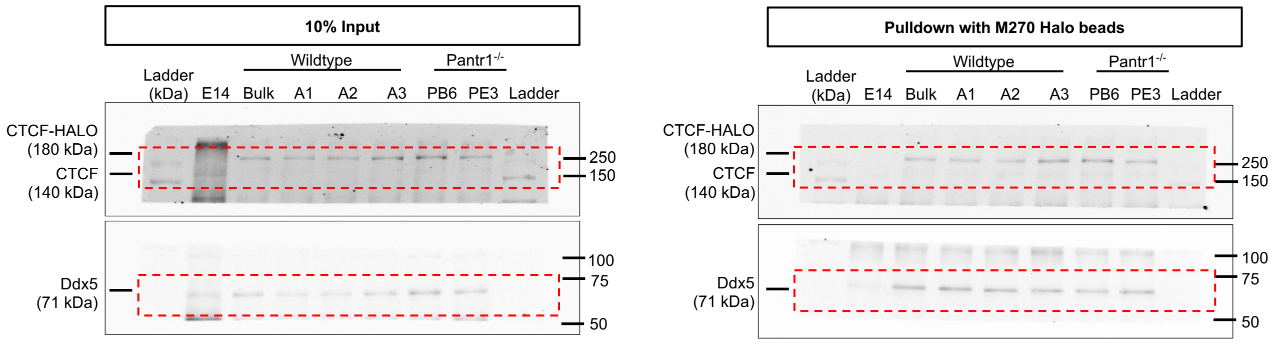

Target protein: Ddx5
Loading control (pulldown): CTCF

Figure 5k : Co-IP for pulldown of RBP- Ddx5 with CTCF-HALO protein using anti-Halo-M270 beads (Replicate 2)

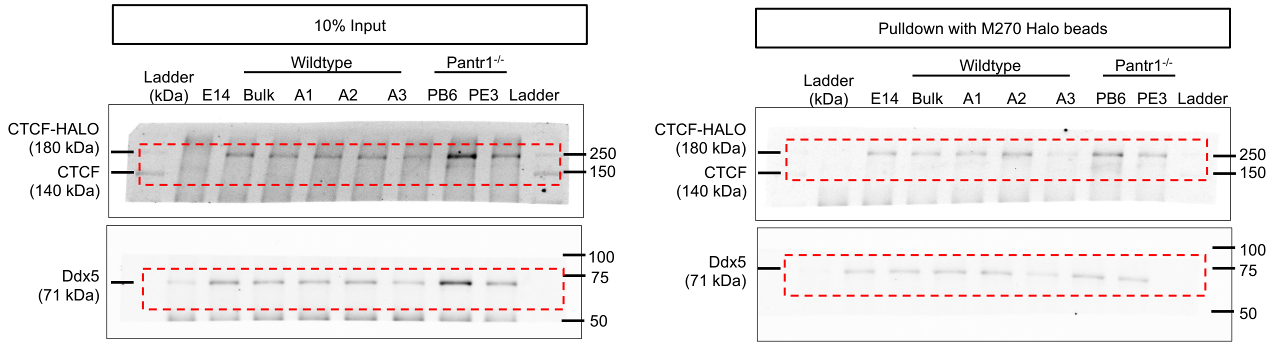

Target protein: Ddx5
Loading control (pulldown): CTCF

Figure 5k : Co-IP for pulldown of RBP- Ddx5 with CTCF-HALO protein using anti-Halo-M270 beads (Replicate 3)

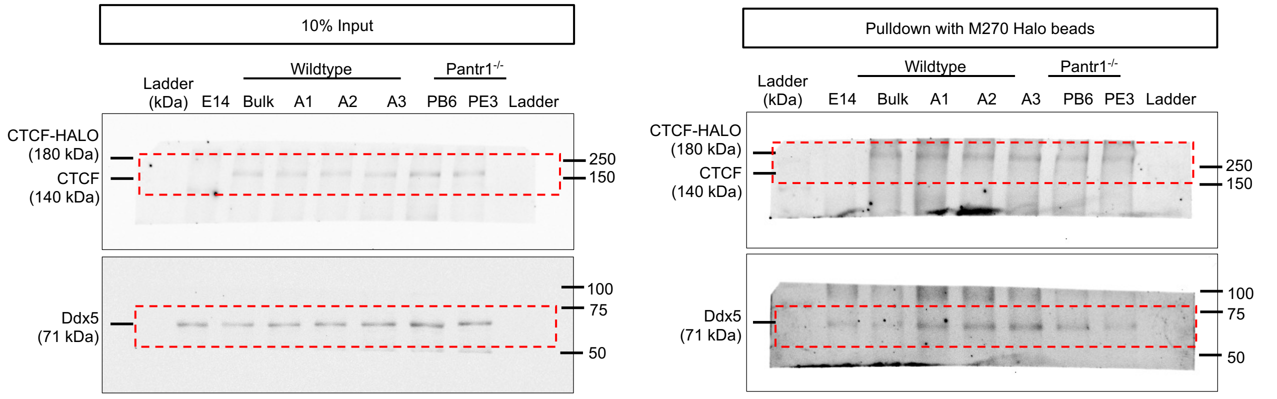

Target protein: Ddx5
Loading control (pulldown): CTCF

Figure 7

Figure 7f : Agarose gel image for genotyping of three CTCF binding site deletion at the Aldh1a3 locus

Aldh1a3 KO#1

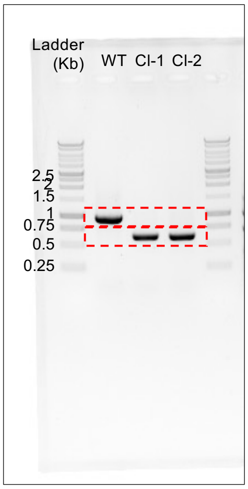

WT: 968 bp
KO #1: 597 bp

Aldh1a3 KO#2

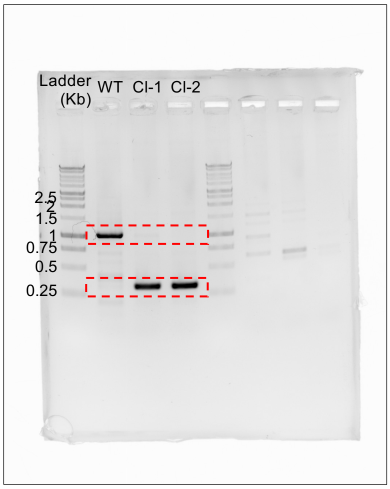

WT: 971 bp
KO #2: 294 bp

Aldh1a3 KO#3

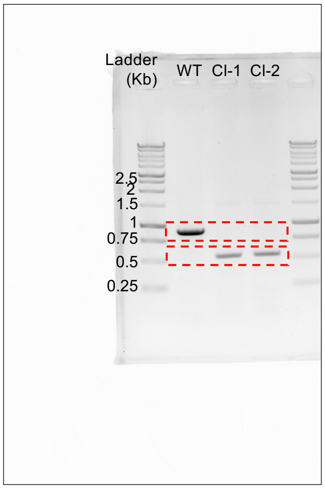

WT: 901 bp
KO #3: 510 bp

WT: 968 bp
Aldh1a3 KO#1: 597 bp

WT: 971 bp
Aldh1a3 KO#2: 294 bp

WT: 901 bp
Aldh1a3 KO#3: 510 bp

Extended Figure 1

Extended Figure 1b: Ctcf expression in ES and NS cells

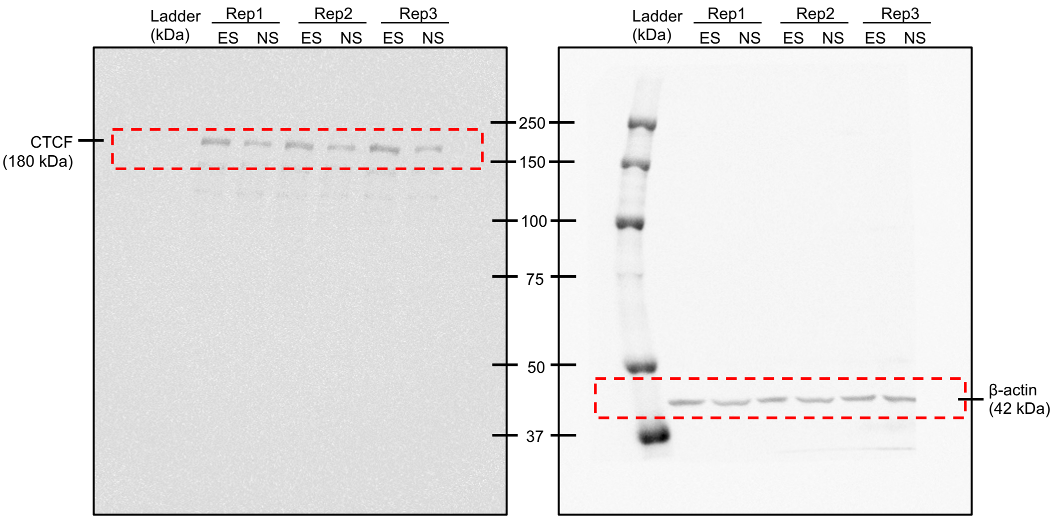

Target protein: CTCF
Loading control: β-actin

Extended Figure 3

Extended Figure 3c: Western blot analysis for RBPs Nono for PLA in WT ES and NS cells

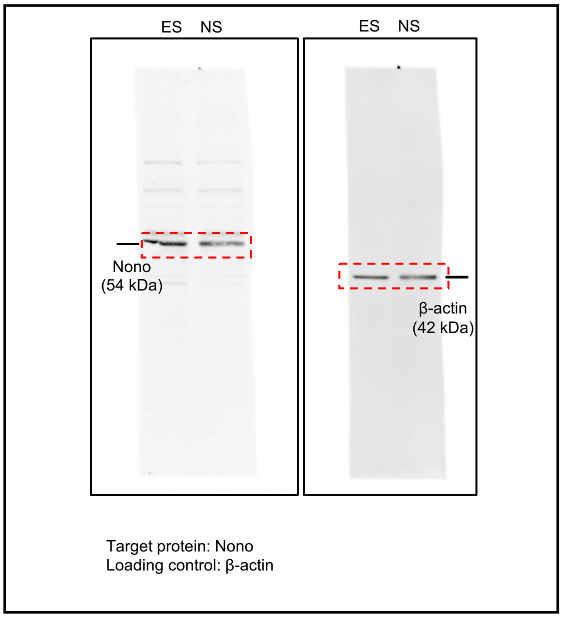

Extended Figure 1

Extended Figure 4b: Validation of Ddx5-KO via genotyping and Protein expression in CTCF-HALO ES cells

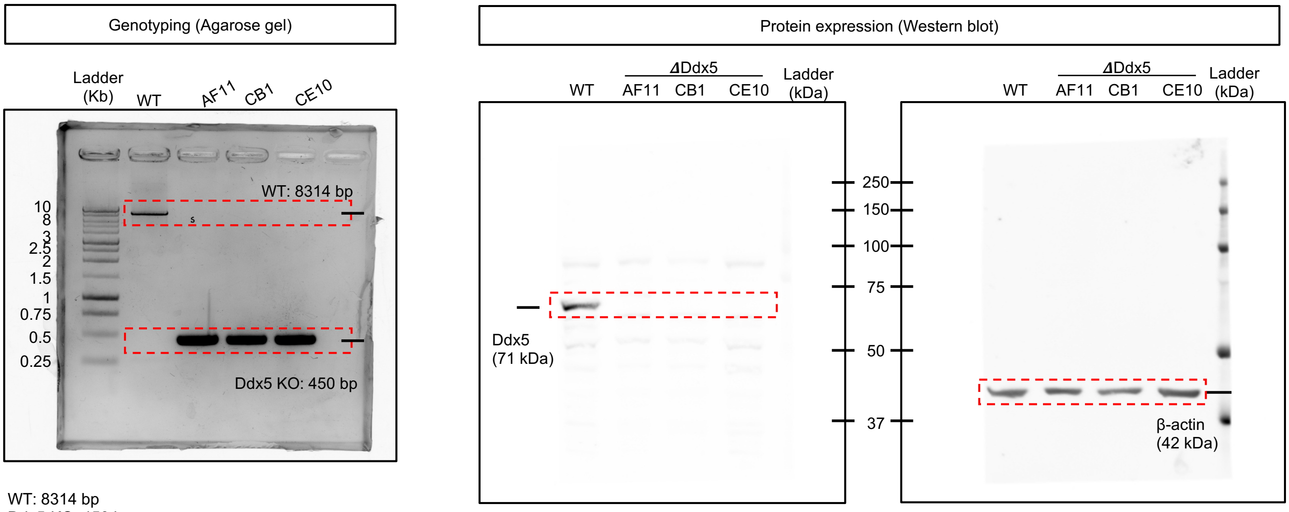

WT: 8314 bp
Ddx5-KO: 450 bp

Target protein: Ddx5
Loading control: β-actin

Extended Figure 4c: Validation of Fus-KO via genotyping and Protein expression in CTCF-HALO ES cells

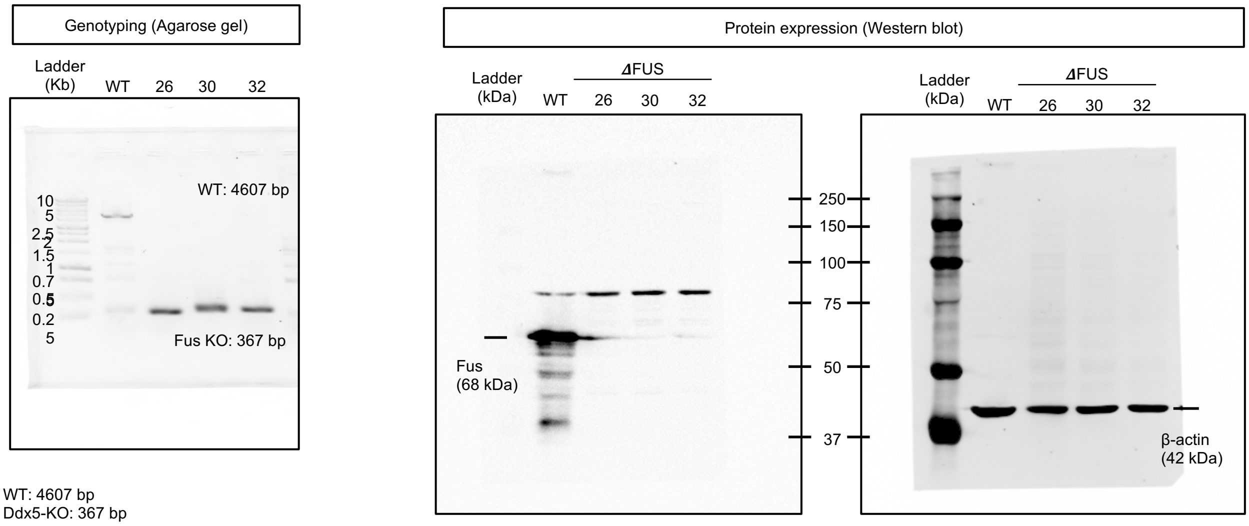

WT: 4607 bp
Ddx5-KO: 367 bp

Target protein: Fus
Loading control: β-actin

Extended Figure 4

Extended Figure 4e: Ctcf expression in NS cells for WT and KOs (Ddx5 and Fus)

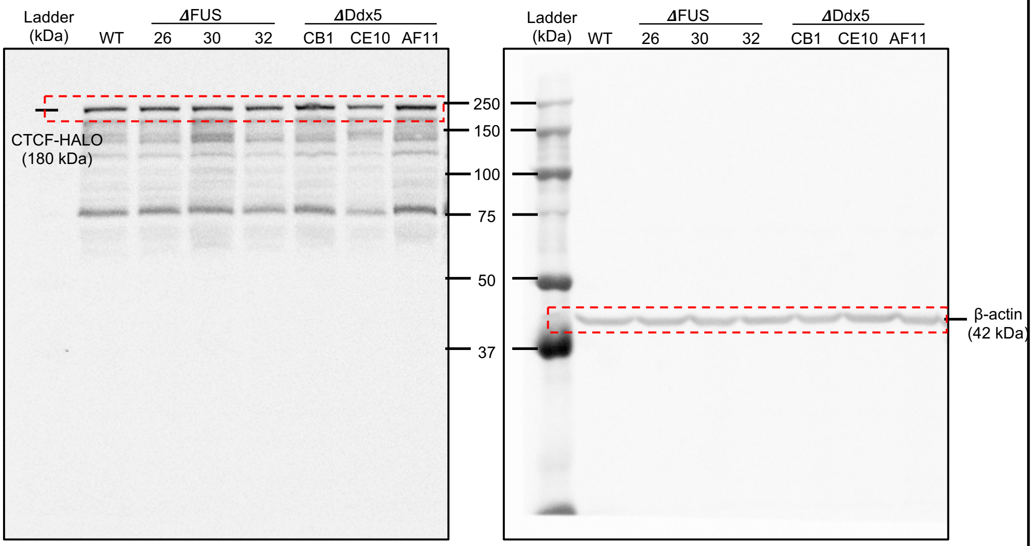

Target protein: CTCF
Loading control: β-actin

Extended Figure 6

Extended figure 6e : Agarose gel image for genotyping of Pantr1-KO in CTCF-HALO ES cells

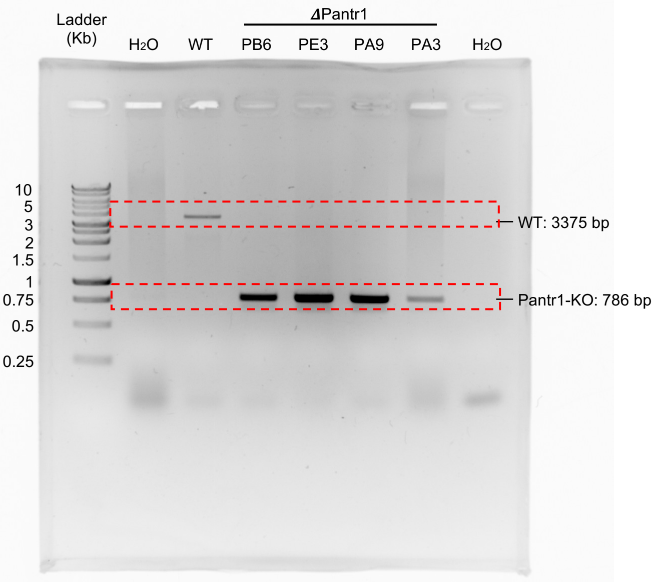

WT: 3375 bp
Pantr1 gene deletion: 786 bp
H_2O: Negative control

Extended Figure 7

Extended figure 7b : Agarose gel image for genotyping of Neat-KO in CTCF-HALO ES cells

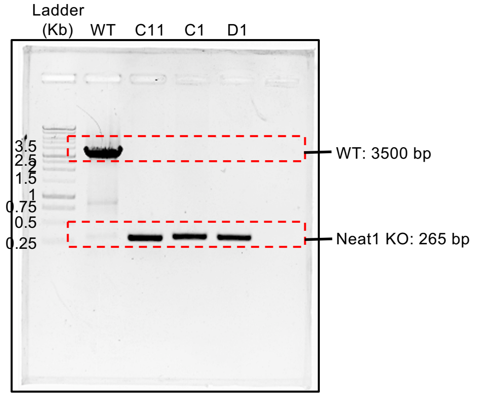

WT: 3500 bp
Neat1-KO : 265 bp
